# Differential microRNA profiles of intramuscular and secreted extracellular vesicles in human tissue-engineered muscle

**DOI:** 10.3389/fphys.2022.937899

**Published:** 2022-08-25

**Authors:** Christopher G Vann, Xin Zhang, Alastair Khodabukus, Melissa C. Orenduff, Yu-Hsiu Chen, David L. Corcoran, George A. Truskey, Nenad Bursac, Virginia B. Kraus

**Affiliations:** ^1^ Duke Molecular Physiology Institute, Duke University School of Medicine, Duke University, Durham, NC, United States; ^2^ Department of Orthopaedic Surgery, Duke University School of Medicine, Duke University, Durham, NC, United States; ^3^ Department of Biomedical Engineering, Pratt School of Engineering, Duke University, Durham, NC, United States; ^4^ Department of Genetics, University of North Carolina School of Medicine, University of North Carolina, Chapel Hill, NC, United States; ^5^ Department of Medicine, Duke University School of Medicine, Duke University, Durham, NC, United States

**Keywords:** extracellular vescicles, microRNA, skeletal muscle, engineered tissue, miRNA sequencing

## Abstract

Exercise affects the expression of microRNAs (miR/s) and muscle-derived extracellular vesicles (EVs). To evaluate sarcoplasmic and secreted miR expression in human skeletal muscle in response to exercise-mimetic contractile activity, we utilized a three-dimensional tissue-engineered model of human skeletal muscle (“myobundles”). Myobundles were subjected to three culture conditions: no electrical stimulation (CTL), chronic low frequency stimulation (CLFS), or intermittent high frequency stimulation (IHFS) for 7 days. RNA was isolated from myobundles and from extracellular vesicles (EVs) secreted by myobundles into culture media; miR abundance was analyzed by miRNA-sequencing. We used edgeR and a within-sample design to evaluate differential miR expression and Pearson correlation to evaluate correlations between myobundle and EV populations within treatments with statistical significance set at *p* < 0.05. Numerous miRs were differentially expressed between myobundles and EVs; 116 miRs were differentially expressed within CTL, 3 within CLFS, and 2 within IHFS. Additionally, 25 miRs were significantly correlated (18 in CTL, 5 in CLFS, 2 in IHFS) between myobundles and EVs. Electrical stimulation resulted in differential expression of 8 miRs in myobundles and only 1 miR in EVs. Several KEGG pathways, known to play a role in regulation of skeletal muscle, were enriched, with differentially overrepresented miRs between myobundle and EV populations identified using miEAA. Together, these results demonstrate that *in vitro* exercise-mimetic contractile activity of human engineered muscle affects both their expression of miRs and number of secreted EVs. These results also identify novel miRs of interest for future studies of the role of exercise in organ-organ interactions *in vivo*.

## Introduction

Skeletal muscle is the largest organ system in the human body. It is responsible for locomotion and known to play critical roles in whole-body glucose metabolism and energy homeostasis. Skeletal muscle also contributes to homeostatic adaptation in peripheral organs (Plomgaard et al., 2012; Guo et al., 2017). Importantly, emerging research in muscle-derived extracellular vesicles (EVs), which carry proteins, lipids, mRNAs and microRNAs (miRs), has provided further insight into mechanisms of crosstalk between muscle and other tissues (Guescini et al., 2010; Forterre et al., 2014; Yoon et al., 2014; Rome et al., 2019).

Epigenetic changes can generally be described as the alterations in gene expression profiles not attributed to changes in DNA sequence (Peschansky and Wahlestedt, 2014). There are three main types of epigenetic mechanisms: i) DNA methylation; ii) histone modification; and iii) non-coding RNA (ncRNA)-associated gene silencing (Lacal and Ventura, 2018; Jacques et al., 2019). Small regulatory RNAs (sRNA) are a class of ncRNA consisting of short interfering RNA (siRNA) and, of importance to this study, miRs. miRs are ∼15–25 nucleotides in length and are generally associated with gene silencing (O'Brien et al., 2018) via post-translational modification and RNA degradation. Canonically, miRs are synthesized through the microprocessor complex consisting of DGCR8, Drosha, and the enzyme ribonuclease III (Denli et al., 2004; O'Brien et al., 2018). All cells export various cellular cargo, including miRs, through EVs–which are synthesized via budding of the plasma membrane or through maturation of endosomes by which intraluminal vesicles accumulate, forming multivesicular bodies, which are then released through exocytosis (De Jong et al., 2014; Rome et al., 2019; Valentino et al., 2021).

The release of EVs by exercising muscle is one means by which exercise affects other organs and tissues (Nederveen et al., 2020; Zhang et al., 2021). Importantly, the number of muscle-derived EVs (specifically small EVs 100–130 nm in size, frequently referred as exosomes in early studies) increases following various modes of exercise (Frühbeis et al., 2015; Whitham et al., 2018; Denham and Spencer, 2020; Vechetti et al., 2021b). Additionally, expression of muscle-derived and circulating miRs are altered following acute and chronic exercise (Backes et al., 2014; Xu et al., 2015; Masi et al., 2016; Ogasawara et al., 2016). To date, studies that have sought to quantify miRs per EV have demonstrated very low molecule counts at ∼1-2 miRs per EV (Chevillet et al., 2014; Helwa et al., 2017), however the concentration of EVs in the circulation is very high, estimated at 10^10^ per mL (Johnsen et al., 2019), suggesting that EVs could be a possible means of gene silencing, which requires a concentration of ∼1,000 copies of a miR per cell (Mukherji et al., 2011). Further, recent work has estimated ∼5% of circulating tetraspanin-positive EVs are muscle-derived *in vivo* (Estrada et al., 2022).

The purpose of the current study was to evaluate the differential expression of miRs, identified and quantified using microRNA sequencing (miRNAseq), in both three-dimensional tissue-engineered models of human skeletal muscle (“myobundles”) (Madden et al., 2015) and their secreted EVs following chronic low frequency and intermittent high frequency electrical-stimulation (e-stim) treatment as a method of simulating exercise. E-stim is commonly used as an exercise mimetic for *in vitro* studies evaluating the effects of exercise on cellular physiology (Nikolić et al., 2012; Tarum et al., 2017). These *in vitro* skeletal muscle culture models (monolayer and engineered 3D tissues) provide a means of studying skeletal muscle development, function, and plasticity (Madden et al., 2015; Rao et al., 2018; Khodabukus, 2021), whilst also affording the capability to investigate the biological processes regulated by miRs in skeletal muscle (Cheng et al., 2016; Rhim et al., 2020). Furthermore, these systems can also be used to evaluate crosstalk between muscle and other cell types via co-culture and/or treating other cell types in isolation with EVs collected from muscle culture systems. To date, numerous studies have demonstrated effects of e-stim on metabolism, force production, and fiber phenotype (Nikolić et al., 2012; Khodabukus et al., 2015; Khodabukus et al., 2019; Chen et al., 2021) in 2D and 3D muscle culture models. However, the effects of e-stim on the expression of miRs within 3D-engineered skeletal muscle and muscle-derived EVs remain understudied. Furthermore, we sought to elucidate the correlation between intracellular and EV miRs to identify miRs that may be preferentially enriched in either muscle and EVs.

## Methods

**FIGURE 1:**
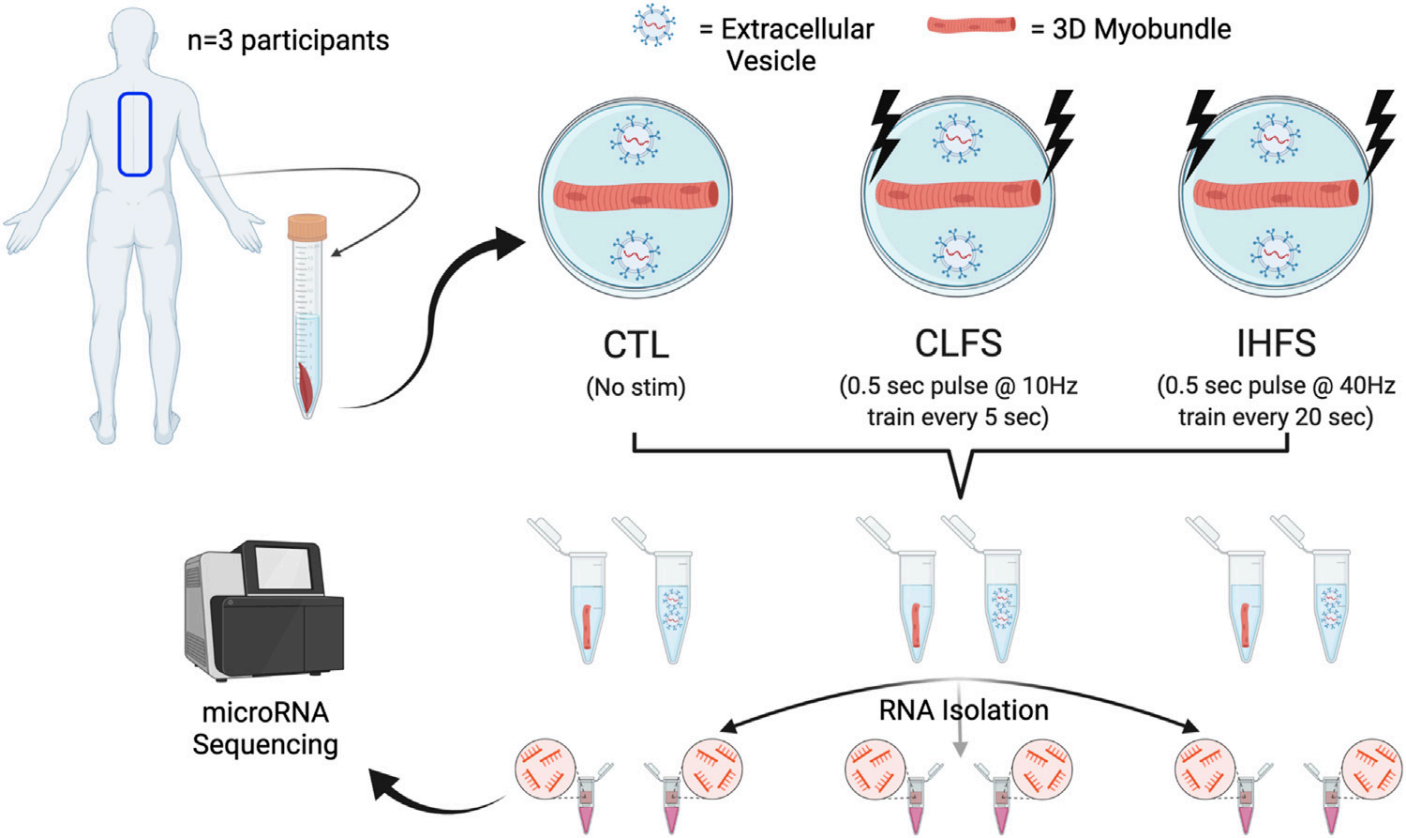
Study design. Abbreviations: CTL, control; CLFS, chronic low frequency stimulation; IHFS, intermittent high frequency stimulation.

Human skeletal muscle samples were obtained from three donors (12 years old female, 18 years old female, and 16 years old male) with informed consent under Duke University Institutional Review Board (IRB) approved protocols (Pro00048509 and Pro00012628). Tissue samples were derived from paraspinal muscle and collected via surgical waste. An overview of the study design can be found in Figure 1 (generated using BioRender.com). Briefly, paraspinal muscle tissue was collected and subsequently minced and expanded prior to 3D muscle myobundle formation. Following myobundle formation, engineered tissues were subjected to one of three conditions; i) Control (CTL); ii) chronic low frequency stimulation (CLFS); or iii) intermittent high frequency stimulation (IHFS). Following treatment, culture media were collected and underwent EV isolation. Total RNA was then isolated from myobundles and EVs prior to submitting for miRNAseq. The methodologies used herein are described in further detail below.

### Myoblast isolation and formation of engineered myobundle

Following tissue procurement), myoblasts were isolated, grown, and subsequently used to engineer three-dimensional engineered muscle tissues (myobundles) as previously described (Madden et al., 2015; Khodabukus et al., 2019; Khodabukus et al., 2020). Briefly, muscle samples were minced and digested with 0.05% trypsin at 37 ° C for 30 min. Isolated cells were then centrifuged and resuspended in growth media (GM) consisting of low-glucose DMEM, 10% fetal bovine serum (FBS), supplemented with SkGM bulletkit without gentamycin and insulin (Lonza Group LTD.; Basel, Switzerland) and subsequently pre-plated for 2 h to decrease fibroblast numbers. Following pre-plating, cells were seeded onto Matrigel coated flasks (BD Biosciences; Franklin Lakes, NJ, United States) and expanded via passaging once 70% confluence was reached. Cells were detached and used to fabricate myobundles at passage 5.

Three-dimensional engineered muscle myobundles were generated within polydimethylsiloxane (PDMS) molds containing two semi-cylindrical wells (7 mm long × 2 mm diameter), cast from 3D-machined Teflon masters as described previously (Madden et al., 2015; Rao et al., 2018). PDMS molds were then coated with 0.2% (w/v) Pluronic^™^ F-127 (Invitrogen; Waltham, MA, United States) for 1 h at room temperature to prevent hydrogel adhesion. Laser-cut Cerex^®^ frames (9 × 9 mm^2^, 1 mm wide rim) were positioned around the wells and served to anchor myobundle ends and facilitate handling and implementation. Briefly, a cell solution (7.5 × 10^5^ cells in 17.2 µL media per bundle +0.5 µL 80 μg/ml aprotinin in water + 2 µL of 50U/mL thrombin in 0.1% BSA in PBS) and a gelling solution (11 µL media +10 µL Matrigel +10 µL of 20 mg/ml Fibrinogen in DMEM) were prepared on ice in separate vials for up to six myobundles per vial. Excessive fibrinolysis was reduced via the inclusion of aprotinin (Khodabukus and Baar, 2009). Gelling solution was added to the cell solution and each myobundle was individually pipetted within the PDMS mold and onto each frame. The cell/hydrogel mixture was then injected into the PDMS wells and subsequently polymerized at 37°C for 30 min. After formation, myobundles were dynamically cultured in a rocker and fed with GM supplemented with 1.5 mg/ml 6-aminocaproic acid (ACA; MilliporeSigma; Burlington, MA, United States) for a period of 4-days. Media were then switched to serum-free differentiation media consisting of low-glucose DMEM, 1% N2-supplement (ThermoFisher, Waltham, MA, United States), 100U/mL penicillin (MilliporeSigma), and 2 mg/ml ACA. Myobundles were cultured in DM for a period of 7-days prior to being subjected to e-stim with media being changed daily.

### Electrical stimulation protocol

Three myobundles were generated from each participant tissue sample; myobundles were subjected to either a CTL condition, which received no electrical stimulation, or one of two electrical stimulation protocols (CLFS and IHFS), which began after 7 days of differentiation. Thus, each treatment condition had *n* = 3 myobundles. Myobundles subjected to CLFS received continuous electrical stimulation consisting of a 0.5s 10Hz train followed by a 4.5s rest. The IHFS protocol consisted of a 0.5s 40Hz train delivered every 20 s for 1h, followed by a 7 h rest period. E-stim protocols were conducted for a period of 7-days. Electrical impulses delivered to the myobundles were bipolar at an amplitude of 70 mA with a duration of 2 ms and were delivered using a D330 Multistim System (Digimeter Ltd.; Hertfordshire, United Kingdom) and programmed using a custom-made pulse generating Labview program (NI; Austin, TX, United States).

### EV isolation

The serum-free media in the engineered muscle myobundle culture were collected and replaced with fresh media daily. EVs were isolated from the serum-free media supernatants collected on day 6 following cultures in the absence or presence of electrical stimulation. Briefly, a standard volume of 1 ml of supernatant was used for each corresponding myobundle. Supernatants were completely thawed on ice and subsequently centrifuged at 2,000 g for 10 min at 4 ° C to remove debris. Following debris removal, EVs in the supernatant were separated using ExoQuick-TC (System Biosciences; Palo Alto, CA, United States) in accordance with the manufacturer’s specifications. The estimated size and number, surface marker CD9 (BD Biosciences; Cat ID: 743048), bilayer structure (PKH; Sigma-Aldrich; Cat ID: MIDI26-1 KT), and mitochondria cargo (MitoTracker Green for total mitochondria and MitoTracker Deep Red for functional respiring mitochondria; Thermo Fisher Scientific; Cat IDs: M7514 and M22426 respectively) of EVs were determined via high resolution flow cytometry (Supplementary Figure S1) as previously described (Zhang et al., 2020). All isolated EVs were subjected to RNA extraction described below.

### RNA extraction and sequencing

RNA isolation was performed on the muscle myobundles and EVs prior to submitting samples for miRNAseq at the Duke University Center for Genomic and Computational Biology. RNA was extracted from myobundles using the Aurum^™^ Total RNA Mini Kit (Bio-Rad; Hercules, CA, United States) in accordance with manufacturers specification. Extraction of RNA from EVs was performed using ExoQuick^®^ Exosome RNA Column Purification Kit (System Bioscience; Palo Alto, CA, United States) in accordance with the manufacturer’s specifications. RNA was subsequently dissolved in 20 μL RNAse-free water and underwent quantification via the Qubit^™^ RNA HS Assay Kit (Thermo Fisher Scientific; Waltham, MA, United States) according to the manufacturer’s specifications.

Library preparation of myobundle and EV RNA samples was completed using the NextSeq 500 Mid-Output Library Kit (Illumina Inc.; San Diego, CA, United States). Following library preparation, sequencing was completed on the Illumina NextSeq500 (Illumina; San Diego, CA, United States) yielding 75bp paired-end reads at a depth of 16 M.

### Data processing, normalization, and statistical analysis

The UMI-tools (Smith et al., 2017) algorithm was used to process miRNAseq data, parsing the Illumina adapters and extracting the unique molecular identifier (UMI) sequence for each read. Reads were then mapped to GRCh38/hg38 using the Bowtie alignment tool (Langmead et al., 2009). Reads were kept for use in subsequent analyses if they mapped to no more than 13 genomic locations. miR counts were compiled using custom scripts that compared the mapped read coordinates to the miRbase miR database (Kozomara and Griffiths-Jones, 2014). Deduplication of reads was then completed using UMI-tools, based on the mapping coordinate and the UMI identified from the read. Reads that matched the coordinates of the known mature miR were kept if they perfectly matched the coordinates of the miR seed whilst not varying more than 2 nt on the 3’ end of the mature miR.

Following the processing of data, a total of 991 miRs were identified in the raw, non-normalized data set. These raw data were then filtered and normalized using the BioConductor package edgeR (Robinson et al., 2010; McCarthy et al., 2012; Chen et al., 2016). Briefly, raw data (991 miRs) were read into R (R Core team, 2020) and subsequently filtered to remove low expressors using a counts per million (CPM) threshold of 0.5 (corresponding to ∼10 counts) whilst additionally requiring more than one sample to meet this threshold to be included in the filtered data set analyses. Following filtering, normalization factors were calculated using the trimmed mean of M-values method, and dispersion was estimated. We used a generalized linear model (GLM) quasi-negative binomial regression (QLF-test) to elucidate differentially expressed miRs while accounting for within sample comparisons with an *a-priori* significance threshold of *p* < 0.05. Importantly, QLF-tests were performed between populations (EV vs. MB) within treatment conditions (CTL, CLFS, IHFS), and within each population between each treatment condition (i.e., EV CLFS vs. CTL; MB CLFS vs. CTL). Critically, we chose to normalize the myobundle and EV data separately for the between treatment within population (myobundle or EV) analyses and perform normalization again on the total data set for the within treatment, between population analyses. Because of the small sample size and the discovery nature of this investigation, we opted to forego use of adjusted *p-*values to identify significant miRs. Following differential expression analysis, miRs meeting the significance threshold were evaluated for correlation with treatment using JMP Pro (JMP, Version 16, SAS Institute Inc., Cary, NC, 1989–2022) using TMM normalized counts. Additionally, we evaluated the overlap of differentially expressed miRs and normalization outputs using Venn diagram analysis via Venny (Oliveros, 2015). A repeated measures one-way ANOVA was used to evaluate differences between populations for miRs identified as being enriched in either the myobundle or EV populations. In the case of significance, we performed paired samples t-tests between populations, within each treatment, to identify differences in raw counts between populations.

## Results

### Differential miR expression within treatment

**FIGURE 2:**
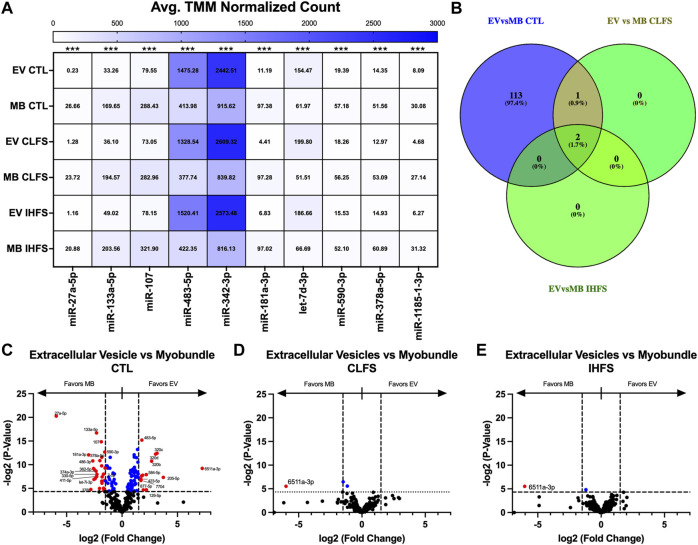
Differential miR Expression Between Population and Within Treatment Condition. Abbreviations: EV, extracellular vesicles; MB, myobundle; CTL, control; CLFS, chronic low frequency stimulation; IHFS, intermittent high frequency stimulation. Legend: data for Panel **(A)** is presented as mean normalized count. Panel **(B)** represents overlap of miRs for analyses completed. Panel **(C–E)** are volcano plots with blue data points depicting *p* < 0.05 and red data points depicting *p* < 0.05 and logFC of >1.5 or < -1.5.

Given that exercise could change miRNA expression and secretion, we evaluated the differential expression pattern of miRs within each treatment condition in both the myobundle and EVs. These analyses yielded 152 differentially expressed miRs overall (*p* < 0.05, Supplementary Table S1). Seven of the top ten miRs (miR-27a-5p, -133a-5p, -107, -181a-3p, -590-3p, -37a-5p, and -1185-1-3p) were enriched in the myobundle (*p* < 0.001; Figure 2A), whereas three (miR-483-5p, -342-3p, and let-7d-3p) were enriched in EVs (*p* < 0.001; Figure 2A).

Exercise has the potential to affect muscle and EV miRs differently. We therefore, evaluated for differences in miR expression within the myobundles compared with EVs to identify miRs that could be differentially expressed in tissue vs. secreted vesicles in response to the exercise-mimetic treatments. Comparing myobundle vs. EV populations, we identified 116 miRs differentially expressed in the CTL treatment group (*p* < 0.05, Figure 2C), 3 differentially expressed miRs (miR-543, -487b-3p, and -6511a-3p) in the CLFS treatment group (*p* < 0.05; Figure 2D), and 2 differentially expressed miRs (miR-6511a-3p and -543) in the IHFS treatment group (*p* < 0.05; Figure 2E). Notably, in our analysis of overlap between treatments groups, only miR-6511a-3p and -543 were significantly differentially expressed (different in myobundle vs. EV) in all 3 treatment conditions and only miR-487b-3p (enriched in myobundle relative to EVs) was differentially expressed in both the CTL and CLFS groups (Figure 2B). Overall, these data demonstrate that e-stim upregulates select miRs that largely favor the myobundle and thus may play a role in tissue regulatory processes.

Given the possibility that EVs transduce signals directly from muscle, we also identified miRs whose expression in EVs was significantly correlated with expression in myobundles (Table 1; Supplementary Figure S2). In the CTL treatment condition, we identified 18 miRs that were significantly correlated between myobundle and EV populations (*p* < 0.05); 15 of these miRs were strongly positively correlated (Pearson R > 0.9, R^2^ > 0.99) whereas 3 miRs were strongly negatively correlated (Pearson R < -0.9, R^2^ > 0.99). In the CLFS treatment condition, we identified 5 miRs as significantly correlated (*p* < 0.05), 3 miRs having strongly positively correlated (R > 0.9) and 2 miRs strongly negatively correlated (R < -0.9, R^2^ > 0.99). Following IHFS stimulation, we identified 2 miRs, both were strongly negatively correlated between the myobundle and EVs (R < -0.9, R^2^ > 0.99, *p* < 0.05).

**TABLE 1:** Pearson correlation of miRs between myobundle and EVs.

Mature miR	Pearson R = CTL	R^2^ = CTL	Pearson p = CTL	Pearson R = CLFS	R^2^ = CLFS	Pearson p = CLFS	Pearson R = IHFS	R^2^ = IHFS	Pearson p = IHFS
30c-5p	1.000	1.000	**0.019** ^*^	0.955	0.912	0.192	0.517	0.267	0.654
374b-5p	1.000	1.000	**0.011** ^*^	0.575	0.331	0.610	0.087	0.008	0.945
190a-5p	1.000	1.000	**0.017** ^*^	0.933	0.870	0.235	0.503	0.253	0.664
140-5p	1.000	1.000	**0.005** ^**^	−0.584	0.341	0.603	−0.100	0.010	0.936
296-3p	1.000	1.000	**<0.001** ^***^	0.653	0.426	0.547	0.473	0.224	0.686
485-3p	0.999	0.998	**0.025** ^*^	0.006	<0.001	0.996	0.545	0.297	0.633
497-5p	0.999	0.998	**0.032** ^*^	0.662	0.438	0.540	0.854	0.729	0.348
664a-5p	0.999	0.998	**0.027** ^*^	0.996	0.992	0.055	−0.999	0.998	**0.022** ^*^
337-3p	0.999	0.0998	**0.033** ^*^	0.368	0.135	0.760	0.765	0.585	0.445
7706	0.998	0.996	**0.039** ^*^	0.908	0.824	0.276	0.868	0.753	0.330
27b-3p	0.998	0.996	**0.042** ^*^	0.599	0.359	0.591	0.823	0.677	0.384
181b-5p	0.998	0.996	**0.036** ^*^	0.230	0.053	0.852	0.729	0.531	0.480
126-3p	0.997	0.994	**0.045** ^*^	0.474	0.225	0.686	0.676	0.457	0.527
328-3p	0.997	0.994	**0.046** ^*^	0.826	0.682	0.381	0.384	0.147	0.749
23b-3p	0.766	0.587	0.445	1.000	1.000	**0.006** ^**^	0.930	0.865	0.240
92a-3p	0.556	0.309	0.625	−1.000	1.000	**0.015** ^*^	−0.867	0.752	0.332
125a-5p	0.454	0.206	0.700	1.000	1.000	**0.016** ^*^	0.229	0.052	0.853
107	−0.026	0.001	0.983	−0.998	0.996	**0.045** ^*^	0.266	0.071	0.828
92b-3p	−0.333	0.111	0.784	1.000	1.000	**0.014** ^*^	−0.444	0.197	0.707
197-3p	−0.998	0.996	**0.042** ^*^	−0.408	0.166	0.733	−0.788	0.621	0.422
let-7f-5p	−0.998	0.996	**0.045** ^*^	−0.212	0.045	0.864	−0.753	0.567	0.457
192-5p	−0.998	0.996	**0.039** ^*^	−0.288	0.083	0.814	−0.725	0.526	0.484
206	−0.999	0.998	**0.021** ^*^	−0.885	0.783	0.308	−1.000	1.000	**0.015** ^*^

miR, microRNA; CTL, Control; CLFS, Chronic Low Frequency Stimulation; IHFS, Intermittent High Frequency Stimulation; EV, extracellular Vesicles.

These data are significant miRs identified *via* QLF-test that also had a significant Pearson correlation between the myobundle and EVs. Significant Pearson correlations are in bold font. Legend: ***, *p* < 0.001; **, *p* < 0.01; *, *p* < 0.05.

### Hierarchical clustering and overrepresentation analysis

**FIGURE 3:**
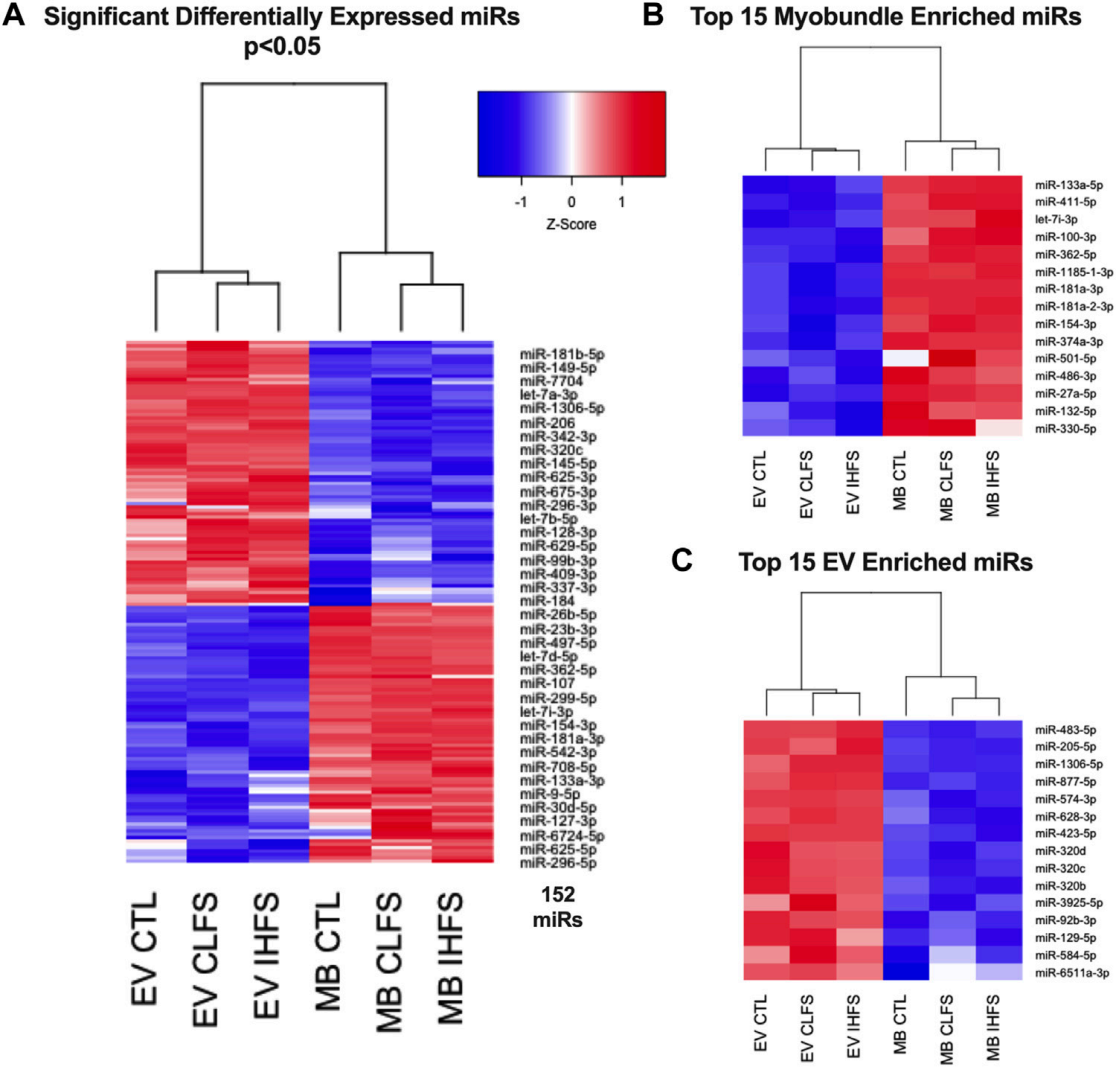
Relationships of Differentially Expressed miRs. Abbreviations: EV, extracellular vesicles; MB, myobundle; CTL, control; CLFS, chronic low frequency stimulation; IHFS, intermittent high frequency stimulation. Legend: data for panels **(A–C)** are presented as mean Z-Score with nodes depicting relationships between population and treatment.

Additionally, hierarchical clustering and over representation analyses were performed on the 152 miRs that met the QLF-test significance threshold. These 152 miR clustered by stimulation condition (CLFS and IHFS) and source (EV and myobundle). To further examine these relationships, we performed clustering of the 15 most highly expressed miRs in myobundle compared with EVs and the 15 most highly expressed in EVs compared with myobundles. Notably, these subgroup analyses also clustered by stimulation condition (CLFS and IHFS) and source (EV and myobundle) (Figures 3B,C). These results show that miR responding to e-stim, clustered together consistent with a similarity of these treatment conditions.

**FIGURE 4:**
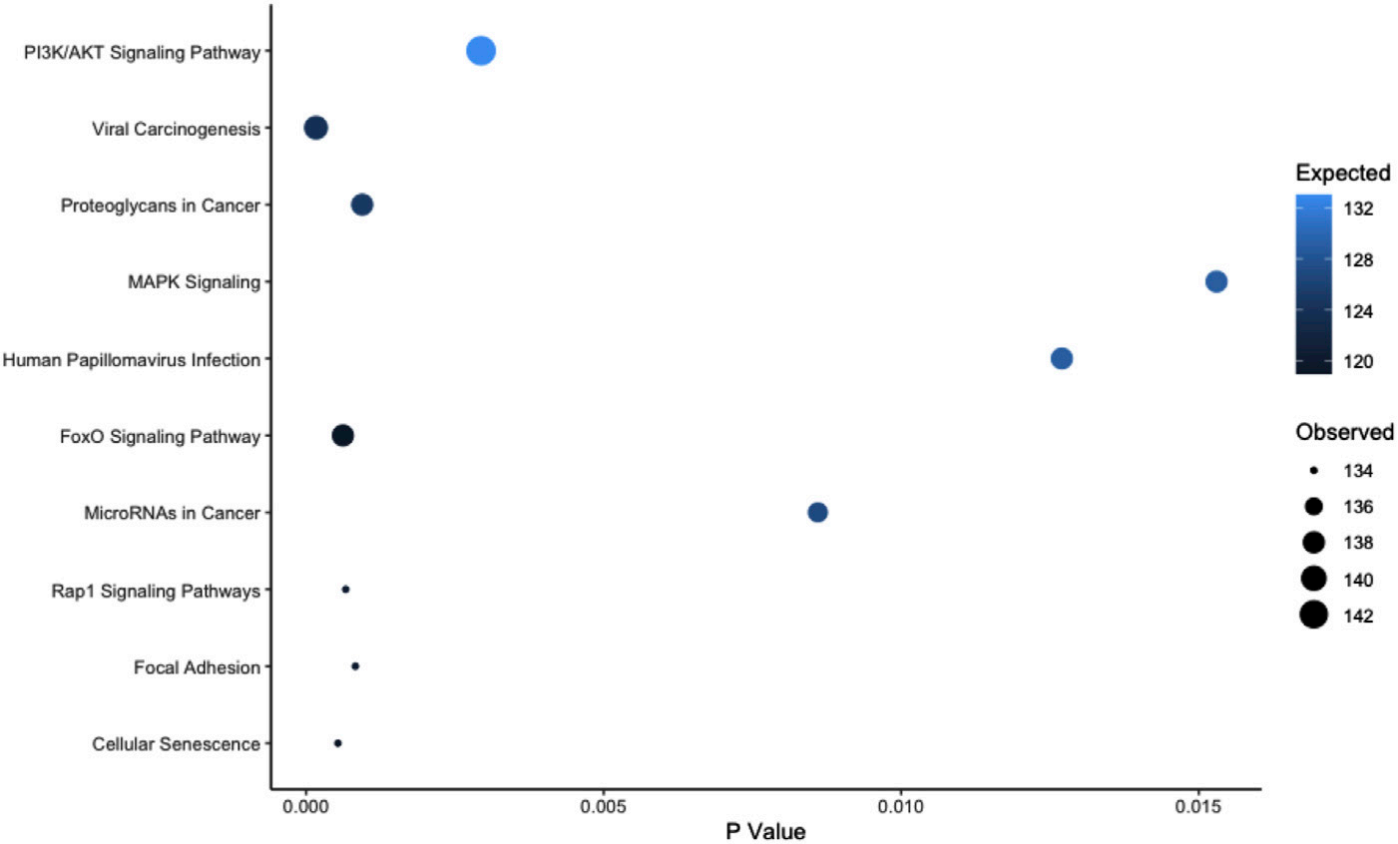
Top 10 Overrepresented KEGG Pathways Based on Observed miRs. Abbreviations: PI3K, phosphoinositide 3-kinase; AKT, protein kinase B; MAPK, mitogen activated protein kinase; FoxO, forkhead box, Rap-1, ras-related protein 1. Legend: pathways are presented by dot size (observed miR count) and color (expected miR count) and plotted in order based on the observed count.

Over representation analysis was performed using the experimentally validated miRNA Enrichment Analysis and Annotation Tool (miEAA) (Kern et al., 2020); top pathways were identified by the number of observed miRs within the pathway. Using the aforementioned 152 miRs and the raw data set prior to filtering (n = 991) as the reference data set, we identified 286 KEGG pathways that were overrepresented in our significant miR set (*p* < 0.05, Supplementary Table S2). The top 10 KEGG pathways can be found in Figure 4; notably, the well documented muscle pathways included in the top 10 were: PI3K/AKT signaling, FoxO signaling, MAPK signaling, and Focal Adhesion pathways (*p* < 0.05). Notably, these miRs were expressed in both the myobundle and EVs, with ∼50% of identified miRs being more highly expressed in the myobundle in all treatment conditions (Supplementary Table S1). Because these miRs were expressed in both populations, it is plausible that pathways identified herein can be regulated locally through muscle derived miRs and systemically via miRs carried by EVs.

### Myobundle differential miR expression between treatments

**FIGURE 5:**
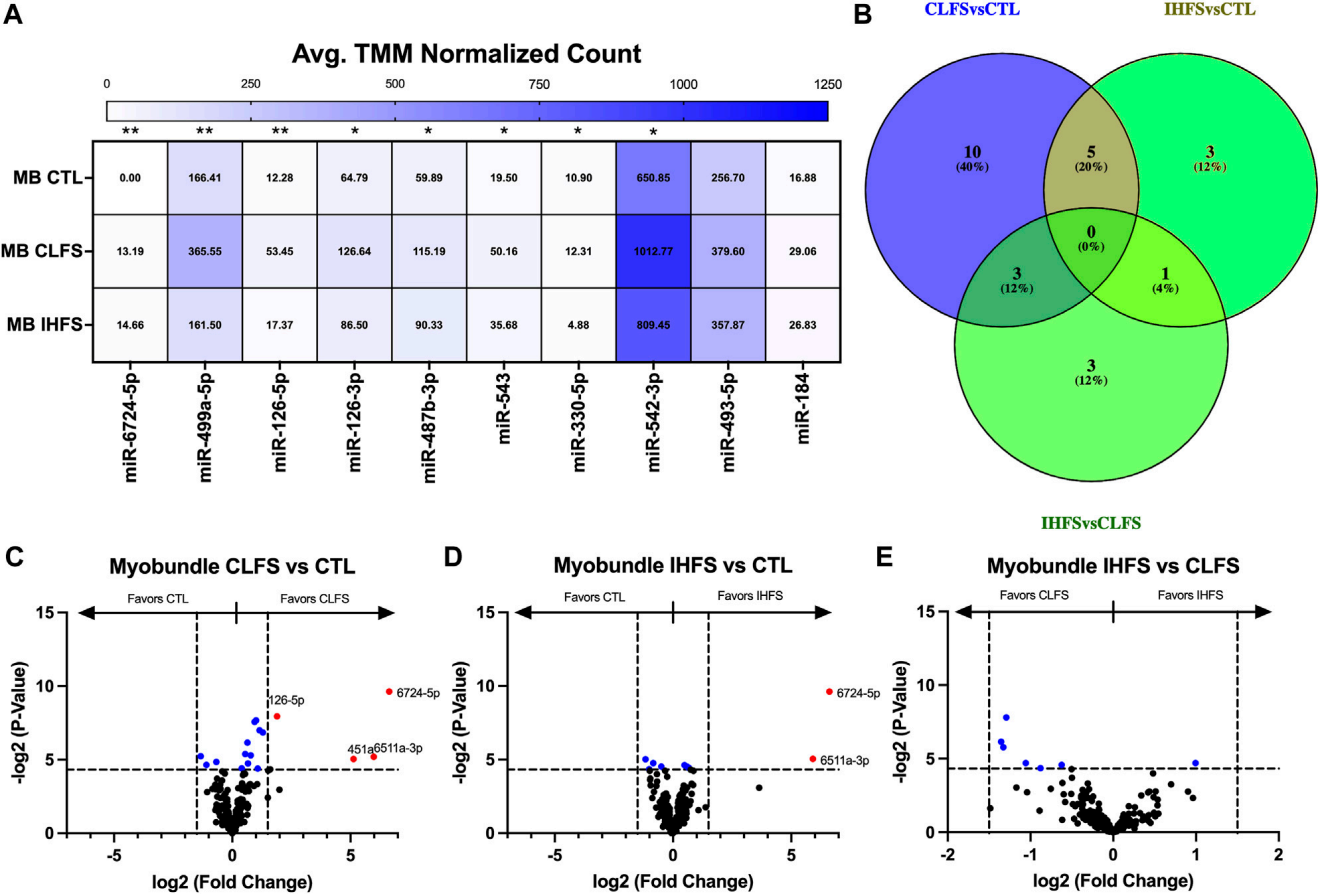
Differential Myobundle miR Expression Between Treatments. Abbreviations: EV, extracellular vesicles; MB, myobundle; CTL, control; CLFS, chronic low frequency stimulation; IHFS, intermittent high frequency stimulation. Legend: data for Panel **(A)** is presented as mean normalized count. Panel **(B)** represents overlap of miRs for analyses completed. Panels **(C–E)** are volcano plots with blue data points depicting *p* < 0.05 and red data points depicting *p* < 0.05 and logFC of >1.5 or < -1.5.

To evaluate the myobundle miR response to exercise-mimetic conditions, we compared CLFS to CTL, IHFS to CTL, and IHFS to CLFS. Overall, we identified 8 miRs (miR-6724-5p, -499a-5p, -126-5p, -126-3p, -487b-3p, -543, -330-5p, and -542-3p) that met the significance threshold (*p* < 0.05; Figure 5A). CLFS compared to CTL yielded 18 differentially expressed miRs (*p* < 0.05; Figure 5C) with 15 miRs enriched in the CLFS treatment compared to 3 enriched in CTL. IHFS compared to CTL yielded 9 differentially expressed miRs (*p* < 0.05; Figure 5D) with 5 miRs enriched in the IHFS treatment compared to 4 miRs enriched in the CTL treatment. IHFS compared to CLFS, yielded 7 differentially expressed miRs (*p* < 0.05; Figure 5E) with 6 miRs enriched in the CLFS treatment.

Although none of these differentially expressed miRs were shared across all treatment conditions (Figure 5B), 5 miRs were differentially expressed in common in the CLFS vs. CTL and IHFS vs. CTL treatments suggesting that these may be exercise responsive miRs.

### EV differential miR expression between treatments

**FIGURE 6:**
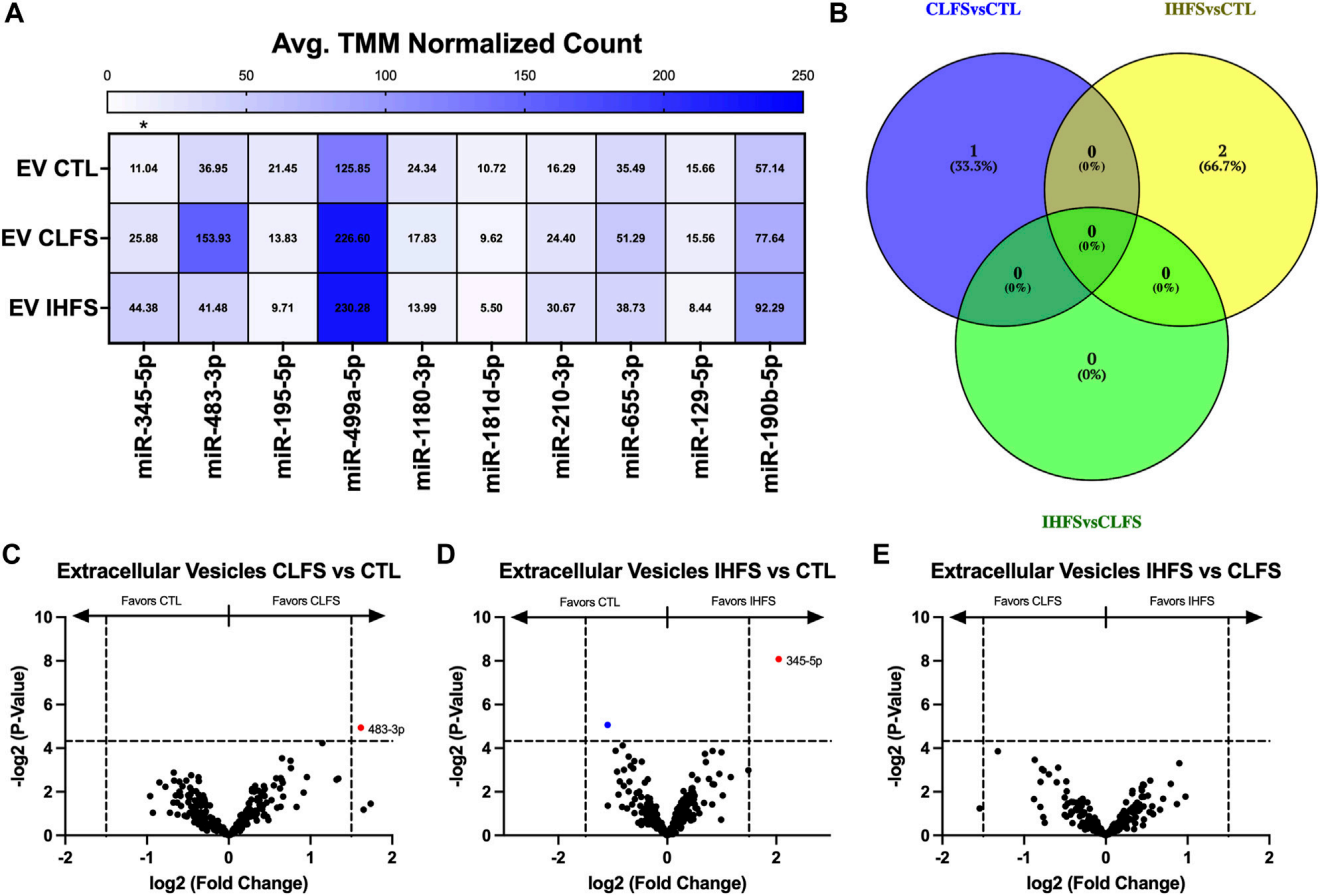
Differential Extracellular Vesicle miR Expression Between Treatments. Abbreviations: EV, extracellular vesicles; MB, myobundle; CTL, control; CLFS, chronic low frequency stimulation; IHFS, intermittent high frequency stimulation. Legend: data for Panel **(A)** is presented as mean normalized count. Panel **(B)** represents overlap of miRs for analyses completed. Panels **(C–E)** are volcano plots with blue data points depicting *p* < 0.05 and red data points depicting *p* < 0.05 and logFC of >1.5 or < -1.5.

To evaluate the EV miR response to exercise-mimetic conditions, we compared all three treatment groups in addition to each pair of conditions (CLFS to CTL, IHFS to CTL, and IHFS to CLFS). Across all treatment groups, we identified miR-345-5p as differentially expressed (*p* = 0.011, Figure 6A). CLFS compared to CTL yielded one differentially expressed miR, miR-483-3p, that was higher in CLFS (*p* = 0.032, Figure 6C). We identified two differentially expressed miRs for IHFS vs. CTL, miR-345-5p (higher in IHFS, *p* = 0.004; Figure 6D) and miR-195-5p (higher in CTL, *p* = 0.030; Figure 6D). There were no differentially expressed miRs identified for IHFS compared to CLFS (*p* > 0.05, Figure 6E). Of interest, there was no overlap identified for these comparisons (Figure 6B). We take this to mean that specific stimulation conditions (simulating exercise *in vivo*) may modulate specific miRs.

### Identification of miRs preferentially expressed in myobundles or EVs

**FIGURE 7:**
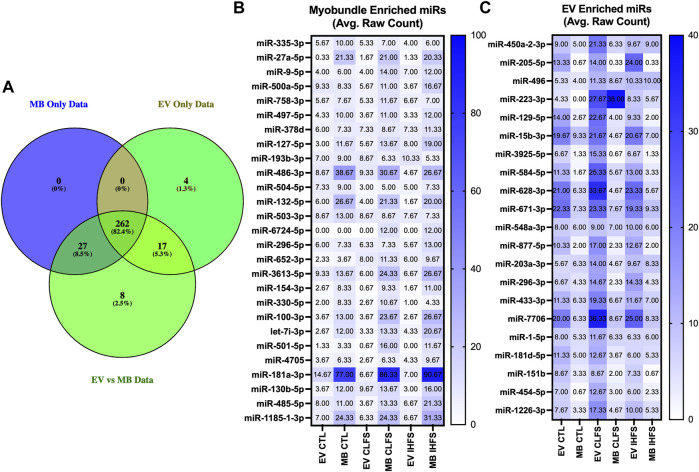
Myobundle and Extracellular Vesicle Enriched miRs. Abbreviations: EV, extracellular vesicles; MB, myobundle; CTL, control; CLFS, chronic low frequency stimulation; IHFS, intermittent high frequency stimulation. Legend: Panel **(A)** represents the overlap of the overall data set used for between population within treatment analyses and the data sets used for the individual myobundle and EV analyses between treatments. Panel **(B)** represents *n* = 27 miRs enriched within the myobundle. Panel c represents *n* = 21 miRs enriched in EVs. Data for panels **(B,C)** are presented as mean raw count.

It should be noted that the default filtering algorithm in the edgeR package seeks to remove low expressors (generally 0.5 counts when expressed as counts per million) if the gene cannot be expressed in all samples for any condition (Chen et al., 2016). This may have unintended consequences of eliminating extreme samples of preferentially expressed miRs (i.e., miRs exclusively expressed in EVs or myobundles). For this reason, we evaluated the previously filtered data sets (filtered to remove low expressors) for overlap to identify miRs whose expression was preferentially enriched in myobundles or EVs (Figure 7A). Overall, 27 miRs were preferentially expressed in myobundles (Figure 7B) while 21 miRs were preferentially expressed in EVs (Figure 7C). Excitingly, this analysis revealed 5 miRs with extreme expression preference (*p* < 0.05) for the myobundle (miRs-27a-5p, -486-3p, -100-3p, -330-5p, and -181-3p; Supplementary Figures S3A–E). Additionally, we identified miR-454-5p as having extreme expression preference for EVs (*p* = 0.039; Supplementary Figure S4A) and 3 additional miRs as trending toward extreme expression preference (*p* < 0.1) for EVs (miRs-887-5p, -7706, and 1226-3p; Supplementary Figures S4B–D).

### Comparison of normalization methods

The processing and normalization of data can impact differential expression results. Following data processing using BOWTIE, we elected to normalize our data using the trimmed mean of M-values (TMM) within the edgeR package. Importantly, we compared this methodology to two other commonly used methods: transcripts per million (TPM) and DESEQ2. While we did not directly compare these outputs to evaluate biological and technical variability, we did perform overlap analyses on miRs identified as differentially expressed from each package using the myobundle data sets comparing CLFS to CTL. Using a *p*-value threshold of *p* < 0.05 for all normalized data sets, there was a notable difference in the quantity of differentially expressed miRs identified by each method: there were 18, 20 and 54 miRs identified as differentially expressed by TMM-edgeR, VST-DESEQ2, and TPM, respectively; 11 miR overlapped among all these data sets, with an additional 6 miR overlap between the TMM-edgeR and TPM data sets (Supplementary Figure S5A). Increasing the threshold stringency for the TPM data set to *p* < 0.01 resulted in a reduction in the number of differentially expressed miRs from 54 to 16; compared to the other two data sets, this yielded an overlap of 7 differentially expressed miRs between all data sets, 10 miRs with TMM-edgeR and 8 miRs with VST-DESEQ2 (Supplementary Figure S5B). These results demonstrate that overall, TPM identifies a larger number of differentially expressed miRs; when constrained to a more stringent *p* < 0.01 threshold, TPM yields a similar number of differentially expressed miRs compared with DESEQ2 and edgeR set to a *p* < 0.05 threshold. This suggests study design, analysis models, and the strengths and shortfalls of different bioinformatic approaches should be considered prior to selection of a bioinformatic approach.

## Discussion

While previous studies have assessed the effects and expression of miRs in tissue-engineered muscle (Rhim et al., 2010; Koning et al., 2012; Cheng et al., 2016), to our knowledge, this study is the first to apply miRNAseq to examine differential miR expression in myobundles subjected to two types of e-stim protocols, and the EVs that these tissues generate. Chief findings include identifying 152 miRs that are differentially expressed between myobundles and EVs with 3 and 2 miRs identified as differentially expressed between myobundles and EVs within the CLFS and IHFS treatment conditions, respectively. Furthermore, within the myobundles, CLFS and IHFS treatment resulted in 18 and 9 differentially expressed miRs compared to CTL, respectively, while within EVs, we only found 1 differentially expressed miR for CLFS and 2 for IHFS compared to CTL. A summary of findings for contrast analyses can be found in Table 2.

**TABLE 2:** Summary of contrast models.

Analysis	Favors myobundle	Favors EV	Citation(s)
EV vs. myobundle CTL	27a-5p; 181a-3p; 378i; 486-3p; 132-5p; let-7i-3p; 362-5p; 127-5p; 374a-3p; 411-5p; 133a-5p; 100-3p; 330-5p; 130b-5p; 1185-1-3p; 107; 1-3p; 378a-5p; 181a-2-3p; 497-5p; let-7f-5p; 7-5p; 23b-3p; 98-5p; 190a-5p; 15a-5p; 299-5p; 590-3p; let-7a-5p; 103a-3p; 15b-5p; let-7d-5p; let-7e-5p; 374a-5p; 31-5p; 374b-5p; 542-3p; 218-5p; 126-3p; 34a-5p; 30e-5p; 708-5p; 99b-5p; 379-5p; 26a-5p; 21-3p; 125b-1-3p; 99a-5p; 26b-5p; 378a-3p; 769-5p; 22-3p; 133a-3p; 30d-5p	6511a-3p; 205-5p; 320c; 320d; 320b; 129-5p; 584-5p; 877-5p; 92b-3p; 483-5p; 1306-5p; 423-5p; 7704; 574-3p; 329-3p; 323a-3p; 342-3p; 184; 628-3p; 576-5p; let-7d-3p; 664a-5p; 485-3p; 192-5p; 382-5p; let-7a-3p; 409-3p; 7706; 432-5p; 93-3p; 629-5p; 665; 543; 92a-3p; 625-3p; 30a-3p; 181b-5p; 136-3p; 1843; 197-3p; 149-5p; 17-5p; 143-3p; 139-5p; 128-3p; 130b-3p; 503-5p; 320a-3p; 493-3p; 941; 323b-3p; 1180-3p; 25-3p; 487b-3p; 151a-3p; 421; 191-5p; 27b-3p; let-7b-5p; 130a-3p; 154-5p; 23a-3p	Drummond et al. (2008), Wong and Tellam (2008), Chen et al. (2009), Nielsen et al. (2010), Sarkar et al. (2010), Dey et al. (2011), Mueller et al. (2011), Ringholm et al. (2011), Lang et al. (2013), Motohashi et al. (2013), Russell et al. (2013), Backes et al. (2014), Chilton et al. (2014), Sato et al. (2014), Cui et al. (2015), Guescini et al. (2015); Kropp et al. (2015); Lozano-Velasco et al. (2015), Wei et al. (2016a), Wei et al. (2016b), Cui et al. (2016), Fyfe et al. (2016), Ogasawara et al. (2016), Narasimhan et al. (2017), Wang et al. (2018); Xu et al. (2018); Huang et al. (2019), Song et al. (2019), Kang et al. (2020), Singh et al. (2020), Yedigaryan and Sampaolesi (2021)
EV vs. myobundle CLFS	543; 487b-3p; 6511a-3p		
EV vs. myobundle IHFS	6511a-3p; 543		
Analysis	Favors Treatment	Favors CTL	
Myobundle CLFSvsCTL	6724-5p; 126-5p; 126-3p; 487b-3p; 499a-5p; 543; 542-3p; 493-5p; 184; 6511a-3p; 451a; 192-5p; 136-5p; 329-3p; 208a-3p	3605-3p; 328-3p; 504-5p	
Myobundle IHFSvsCTL	6724-5p; 6511a-3p; 493-5p; 487b-3p; 184	330-5p; 486-3p; 193a-5p; 335-3p	
Analysis	Favors IHFS	Favors CLFS	
Myobundle IHFSvsCLFS	675-5p	499a-5p; 330-5p; 126-5p; 425-3p	
Analysis	Favors Treatment	Favors CTL	
EV CLFSvsCTL	483-3p		
EV IHFSvsCTL	345-5p	195-5p	
Analysis	Favors IHFS	Favors CLFS	
EV IHFSvsCLFS	No significant miRs identified		

These data represent miRs identified as differentially expressed for all contrast models.

EV, extracellular vesicle; CTL, control; CLFS, chronic low-frequency stimulation; IHFS, intermittent high-frequency stimulation. miRs that are colored blue have been evaluated in skeletal muscle cell culture models and/or *in vivo* with exercise stimulus.

Skeletal muscle exports various myokines and EVs that exert effects on other target tissues (Pedersen and Febbraio, 2008; Pratesi et al., 2013; Rome et al., 2019; Darkwah et al., 2021). In our study, differentially expressed miRs in myobundles vs EVs were miR-543, -487b-3p, and -6511a-3p for CLFS and miR-543 and -6511a-3p for IHFS treatment. Notably, all these miRs were enriched in the myobundles compared with EVs. Previous work has shown that miR-543 plays a role in proliferation of C2C12 cells via targeting Krüppel-like factor 6 (KLF-6), which is a suppressor of multiple tumor cells (Lang et al., 2013; Kang et al., 2020). Interestingly, although miR-487b-3p was not related to any of the pathways identified in this study, miR-6511a-3p was identified in the viral carcinogenesis, proteoglycans in cancer, MAPK signaling, microRNAs in cancer, Rap1 signaling, and focal adhesion pathways, and miR-543 was found in all the top 10 overrepresented pathways. Notably, miRs-6511a-3p and -487b-3p have yet to be investigated in the context of human skeletal muscle.

Using the 152 miRs identified through differential expression analysis of myobundles vs EVs, we identified 286 enriched KEGG pathways in this study. Several of these pathways are known to be important in the regulation of skeletal muscle, including PI3K/AKT signaling, MAPK signaling, and focal adhesion pathways. In addition to being important for regulation of skeletal muscle, the previously mentioned pathways as well as others identified in our analysis have also been identified as critical components in myogenic differentiation of pluripotent stem cells (Fei et al., 2021). Focal adhesion kinase (FAK), which localizes around focal adhesion sites, can be activated through mechanotransduction and through growth factors (Quach et al., 2009; Graham et al., 2015) and has effects that regulate cell growth, differentiation, migration, and survival (Schaller, 2001). Mitogen activated protein kinase (MAPK) pathway is well characterized and known to play a vital role in mechanotransduction. As reviewed by Zhang and Liu, MAPK can be activated through multiple stimuli and plays roles in proliferation, differentiation, development, inflammation, apoptosis, and more (Zhang and Liu, 2002). The PI3K/AKT axis is a well characterized pathway in skeletal muscle. Interestingly, Briata and colleagues found that during myogenesis, this pathway is responsible for myomiR maturation in the presence of KH-type splicing regulatory protein (KSRP) through a switch between two distinct KSRP functions leading to activation of miR maturation through interaction at the terminal loop of select miRs or promoting the decay of myogenin (Briata et al., 2011; Briata et al., 2012). This suggests that select understudied miRs, identified in our study (e.g., miRs-6511a-3p and -487b-3p), potentially regulate pathways that are important throughout the muscle cell cycle.

Within EVs, we identified miR-345-5p as differentially expressed comparing IHFS to CTL. Previously, miR-345-5p was shown to be upregulated in rectus abdominis muscle biopsies of cachectic individuals, which was linked to regulation of the ubiquitin-proteasome pathway (Narasimhan et al., 2017; Yedigaryan and Sampaolesi, 2021). Additionally, comparing CLFS to CTL within EVs, we identified miR-483-3p as differentially expressed, which has previously been linked to inhibition of muscle cell proliferation and differentiation through negative regulation of the PI3K/AKT pathway (Song et al., 2019). In a recent review from Rome and colleagues, miRs-451, -6239, -6240, -6236, -144, -223, -5112, 3062, -142a, -2137, and -720 were noted as having higher expression in muscle-derived EVs than in muscle cells (Rome et al., 2019); among these, only miRs-451 and -223 were included in our analyses. In our data, miRs-451 and -223 were not identified as differentially expressed between myobundles and EVs, however, normalized counts for both miRs were higher in EVs compared to myobundles for CTL and in myobundles compared to EVs for CLFS and IHFS treatments.

In general, known muscle specific miRs (termed myomiRs) changed minimally in our study. For instance, miR-133a-5p (a myomiR related to regulation and function of skeletal muscle) increased minimally (log2FC = 0.228; *p* = 0.314) in myobundles following CLFS, with a similar trend following IHFS (log2FC = 0.389; *p* = 0.180). Additionally, other notable muscle-specific miRs, such as miRs-1, -181a, -23a, -27a, and -206, changed minimally in expression. It is worth noting that many of the prior studies investigating changes in miR expression used RT-qPCR or, in some cases, custom microRNA arrays. Notably, these methods–while very accurate and useful–limit interpretation due to the small quantity of genes that are generally interrogated. Previously, *in vivo* models using mouse muscle (van Rooij et al., 2009; Liu et al., 2011; Chemello et al., 2019; Bjorkman et al., 2020; Vechetti et al., 2021a) and *in vitro* models using transfection in C2C12s and human primary cells (Cheng et al., 2016; Chemello et al., 2019), have reported differential regulation of myomiRs-1, -133a, -206, -208b, and -27a. In some human studies evaluating various forms of training stimuli, miRs-1, -133a and b, -181a, -29 b were upregulated (Russell et al., 2013) while other stimuli resulted in downregulation of these miRs and others such as miRs-378 and -486 (Drummond et al., 2008; Nielsen et al., 2010; Mueller et al., 2011; Ringholm et al., 2011; Fyfe et al., 2016). While minimal changes in these commonly interrogated myomiRs in our study are hard to reconcile with the existing literature, it is possible that the myobundle, made using cells from paraspinal muscles (predominantly type I), respond differently to stimulation compared to the *in vivo* studies generally sampling from the vastus lateralis (∼50% type I and 50% type II fibers). Notably, previous work using three-dimensional culture models derived from the tibialis anterior and soleus muscles in rodents has shown different physiological responses to electrical stimulation (Huang et al., 2006). Additionally, although CTL myobundles did not receive electrical stimulation, these tissues do contract spontaneously at <15% of peak force generation, which may contribute to EV secretion and attenuate differences in miR expression with e-stim.

The use of TMM for analysis of miRNAseq data, as used here, has been shown to be effective at reducing sample variance and generating more accurate downstream analytical results (Tam et al., 2015). Prior to normalization and differential expression analysis, it is generally recommended to remove low expressors through filtering because in general, removal of low expressors increases sensitivity in downstream analyses (i.e., differential expression) and does not meaningfully affect significance of other genes within the data set (Bourgon et al., 2010; Sha et al., 2015). However, this may have the unintended consequence of eliminating informative missingness (highly skewed expression in one group and relatively low or undetectable expression another group). For this reason, we recommend a post-hoc evaluation of raw counts of miR excluded through filtering to evaluate for the occurrence of this circumstance.

We also sought to compare TMM outputs with two other commonly used normalization strategies, VST-DESEQ2 and TPM. We conclude that VST-DESEQ2 and TMM-edgeR yield similar outputs, whereas TPM normalization appears to identify a larger number of differentially expressed targets from the same dataset. However, when the TPM threshold is set at *p* < 0.01, the output results are similar to TMM-edgeR and VST-DESEQ2. It is worthwhile to note that TPM accounts for sequencing depth and gene length but does not account for differences in library composition (Corchete et al., 2020; Zhao et al., 2021). It has been posited that normalizing to sequencing depth alone is insufficient for miRNAseq analysis (Garmire and Subramaniam, 2012; Dillies et al., 2013). Conversely, normalization using TMM-edgeR and VST-DESEQ2 overcomes differences in library size and quantifications through use of scaling factors (Dillies et al., 2013). Taken together, the current information suggests that depending on study design, TMM-edgeR and VST-DESEQ2 are more suited to miRNAseq data because they account for variables such as library size and library composition whereas TPM may not be suitable for miRNAseq data because library composition is not accounted for.

As with many studies utilizing human samples, we were limited by sample size. Contamination of the isolated EV pool by other extracellular particles such as protein and lipids is a known limitation of polymer based precipitation (PBP) isolation methods (Zarovni et al., 2015; Li et al., 2017; Brennan et al., 2020). In the context of miR, PBP has been shown to yield higher recovery of EV derived miRs (Chung et al., 2020). As discussed by Zhang and colleagues (Zhang et al., 2022) there is no established “gold standard” for EV isolation applicable to all subsequent analyses, therefore we chose PBP because of higher EV yields given our specific interest of profiling miRs from the myobundle and myobundle derived EVs. Additionally, miRNAseq is still a relatively new technique with numerous methods available for data processing. Following data processing but prior to filtering out low expressors, 991 miRs met the processing criterion, a low value compared to other work evaluating small RNA sequencing expression in native skeletal muscle. While we used an approach that has been shown to minimize variation and generate accurate differential expression data (Tam et al., 2015), many other approaches and available analysis packages could have been used herein; however, there is no current standard practice for analysis of miRNAseq data.

## Conclusion

While miR expression and secreted EVs have been previously analyzed in skeletal muscle *in vivo*, this is the first study to do this analysis for engineered human skeletal muscle tissues devoid of confounding factors from the systemic environment or the multicellular nature of native muscle tissue. The miRs identified herein have been implicated in numerous pathways related to health, disease, metabolism, and regulation and development of skeletal muscle. For instance, we identified miR-6511a-3p as responsive to both CLFS and IHFS stimulation while also noting its presence in several pathways related to regulation of muscle (i.e., MAPK, Rap1, and focal adhesion signaling) however, miR-6511a-3p has yet to be investigated in human muscle tissue. These data provide novel miR targets for future research to elucidate the effects of exercise on muscle and muscle signals transduced by their EVs and are relevant to identifying mechanisms by which muscle regulates endogenous gene expression and how muscle communicates with other tissue.

## Data Availability

Raw data files were deposited to the National Institutes of Health (NIH) Gene Expression Omnibus (GEO) repository and can be found online (https://www.ncbi.nlm.nih.gov/geo/) Accession ID: GSE203157. All relevant data pertaining to EV isolation methods has been uploaded to the EV-Track knowledge base (EV-Track ID: EV220309) (Van Deun et al., 2017).
