# Supplementary material for: Differential microRNA profiles of intramuscular and secreted extracellular vesicles in human tissue-engineered muscle
Source: Front Physiol. 2022 Aug 25;13:937899. doi: 10.3389/fphys.2022.937899 (PMC9452896; doi:10.3389/fphys.2022.937899)
Supplement: Supplementary file 1 [file Table1.docx]

**Supplementary Table 1**: Differential miR Expression Between Population within Treatment ranked by p-value.

| **Mature miR** | **LogFC EVvsMB**  **CTL** | **LogFC**  **EVvsMB**  **CLFS** | **LogFC**  **EVvsMB**  **IHFS** | ***P*-value** |
| --- | --- | --- | --- | --- |
| miR-27a-5p | -5.930 | 1.864 | 2.030 | 3.75E-08 |
| miR-133a-5p | -2.298 | -0.104 | 0.226 | 1.37E-07 |
| miR-107 | -1.869 | -0.092 | -0.186 | 3.19E-07 |
| miR-483-5p | 1.790 | 0.014 | 0.096 | 3.30E-07 |
| miR-342-3p | 1.402 | 0.233 | 0.262 | 6.00E-07 |
| miR-181a-3p | -3.009 | -1.265 | -0.801 | 8.31E-07 |
| let-7d-3p | 1.313 | 0.637 | 0.187 | 9.25E-07 |
| miR-590-3p | -1.557 | -0.024 | -0.193 | 2.11E-06 |
| miR-378a-5p | -1.830 | -0.232 | -0.167 | 3.90E-06 |
| miR-1185-1-3p | -1.995 | -0.500 | -0.304 | 4.09E-06 |
| miR-30a-3p | 1.095 | 0.006 | 0.084 | 4.74E-06 |
| let-7e-5p | -1.345 | -0.454 | -0.352 | 7.05E-06 |
| miR-362-5p | -2.520 | -0.968 | -1.178 | 8.50E-06 |
| miR-320c | 3.157 | -0.129 | -0.515 | 1.10E-05 |
| miR-320d | 3.029 | -0.089 | -0.570 | 1.38E-05 |
| miR-92a-3p | 1.108 | -0.025 | -0.139 | 1.83E-05 |
| let-7d-5p | -1.403 | -0.211 | -0.340 | 2.10E-05 |
| miR-542-3p | -1.201 | -0.627 | -0.555 | 2.70E-05 |
| miR-100-3p | -2.291 | -0.842 | -1.189 | 2.80E-05 |
| miR-374a-5p | -1.304 | -0.140 | -0.179 | 2.86E-05 |
| miR-31-5p | -1.281 | -0.235 | -0.125 | 3.27E-05 |
| miR-132-5p | -2.563 | -0.300 | -1.011 | 3.53E-05 |
| let-7a-5p | -1.527 | -0.208 | -0.180 | 3.86E-05 |
| miR-197-3p | 0.936 | 0.831 | 0.729 | 4.68E-05 |
| miR-320b | 2.671 | -0.151 | -0.350 | 4.73E-05 |
| miR-22-3p | -0.709 | -0.495 | -0.169 | 5.07E-05 |
| miR-486-3p | -2.636 | 0.431 | 0.065 | 5.19E-05 |
| miR-25-3p | 0.786 | 0.106 | 0.104 | 5.68E-05 |
| miR-149-5p | 0.915 | 0.231 | 0.045 | 5.86E-05 |
| miR-181a-2-3p | -1.826 | -1.180 | -0.966 | 5.93E-05 |
| miR-128-3p | 0.872 | 0.129 | 0.355 | 6.91E-05 |
| miR-126-3p | -1.126 | -0.864 | 0.105 | 7.15E-05 |
| miR-99a-5p | -0.809 | -0.082 | -0.203 | 7.57E-05 |
| miR-323b-3p | 0.808 | 0.391 | 0.783 | 8.99E-05 |
| miR-205-5p | 3.732 | 0.852 | 1.970 | 9.02E-05 |
| miR-1306-5p | 1.766 | 0.317 | 0.468 | 9.23E-05 |
| miR-629-5p | 1.121 | -0.190 | 0.061 | 1.01E-04 |
| miR-1-3p | -1.839 | 0.115 | 0.200 | 1.06E-04 |
| miR-323a-3p | 1.432 | -0.241 | -0.310 | 1.11E-04 |
| miR-379-5p | -0.907 | -0.842 | -0.347 | 1.15E-04 |
| miR-374a-3p | -2.340 | -0.352 | 0.271 | 1.42E-04 |
| miR-30e-5p | -1.065 | 0.293 | 0.421 | 1.45E-04 |
| miR-130b-3p | 0.860 | 0.073 | 0.223 | 1.61E-04 |
| miR-574-3p | 1.479 | 0.387 | 0.279 | 1.69E-04 |
| miR-30c-5p | 0.532 | 0.584 | 0.466 | 1.99E-04 |
| miR-485-3p | 1.244 | -0.089 | 0.083 | 2.12E-04 |
| miR-98-5p | -1.665 | -0.195 | -0.014 | 2.12E-04 |
| let-7f-5p | -1.794 | -0.360 | -0.190 | 2.35E-04 |
| miR-15a-5p | -1.576 | -0.303 | -0.581 | 2.38E-04 |
| miR-34a-5p | -1.095 | 0.116 | 0.175 | 2.42E-04 |
| miR-330-5p | -2.263 | -0.325 | 0.084 | 2.55E-04 |
| miR-7-5p | -1.674 | 0.227 | -0.297 | 2.58E-04 |
| miR-411-5p | -2.328 | -0.602 | 0.027 | 2.64E-04 |
| miR-665 | 1.115 | 0.220 | 0.312 | 2.78E-04 |
| miR-576-5p | 1.329 | 0.215 | -0.130 | 3.66E-04 |
| miR-15b-5p | -1.455 | 0.342 | 0.373 | 4.19E-04 |
| miR-103a-3p | -1.475 | -0.263 | -0.352 | 4.32E-04 |
| let-7a-3p | 1.219 | 0.006 | -0.058 | 4.73E-04 |
| miR-191-5p | 0.616 | 0.196 | 0.188 | 4.73E-04 |
| miR-127-3p | -0.662 | -0.690 | -0.305 | 4.85E-04 |
| miR-151a-3p | 0.729 | -0.022 | -0.113 | 4.94E-04 |
| miR-7706 | 1.178 | 0.375 | 0.171 | 5.07E-04 |
| miR-184 | 1.375 | -0.340 | -0.189 | 5.98E-04 |
| miR-409-3p | 1.178 | -0.468 | -0.117 | 7.24E-04 |
| miR-320a-3p | 0.826 | 0.433 | 0.176 | 8.69E-04 |
| miR-382-5p | 1.223 | -0.533 | -0.109 | 8.75E-04 |
| let-7i-3p | -2.526 | 0.064 | 0.154 | 8.96E-04 |
| miR-130a-3p | 0.529 | 0.253 | 0.366 | 9.21E-04 |
| miR-27b-3p | 0.579 | 0.120 | 0.158 | 9.44E-04 |
| miR-423-5p | 1.722 | -0.076 | 0.118 | 9.52E-04 |
| miR-374b-5p | -1.278 | -0.089 | -0.269 | 1.06E-03 |
| miR-628-3p | 1.337 | 0.803 | 0.650 | 1.11E-03 |
| miR-181b-5p | 1.067 | -0.069 | -0.447 | 1.13E-03 |
| miR-6724-5p | 0.055 | -6.923 | -6.853 | 1.19E-03 |
| miR-584-5p | 2.194 | -0.798 | -0.522 | 1.22E-03 |
| miR-23a-3p | 0.492 | 0.173 | 0.111 | 1.24E-03 |
| miR-22-5p | -0.356 | -0.453 | -0.659 | 1.36E-03 |
| miR-92b-3p | 1.858 | -0.345 | -0.428 | 1.42E-03 |
| miR-136-3p | 0.989 | -0.749 | -0.144 | 1.45E-03 |
| miR-93-3p | 1.144 | 0.338 | -0.046 | 1.50E-03 |
| miR-17-5p | 0.906 | 0.563 | -0.105 | 1.51E-03 |
| miR-484 | 0.758 | 0.371 | 0.626 | 1.60E-03 |
| miR-192-5p | 1.228 | -0.316 | -0.112 | 1.70E-03 |
| miR-421 | 0.690 | 0.002 | 0.142 | 1.78E-03 |
| miR-497-5p | -1.811 | -0.162 | 0.025 | 1.82E-03 |
| miR-154-3p | -2.063 | -1.944 | -0.654 | 1.92E-03 |
| miR-26a-5p | -0.903 | 0.128 | -0.113 | 1.93E-03 |
| miR-328-3p | 0.531 | 1.059 | 0.795 | 1.96E-03 |
| miR-708-5p | -0.932 | 0.248 | -0.274 | 2.02E-03 |
| miR-503-5p | 0.849 | -0.067 | 0.091 | 2.04E-03 |
| miR-675-3p | 0.478 | 0.627 | 0.702 | 2.18E-03 |
| miR-195-5p | -0.591 | -0.394 | -1.056 | 2.25E-03 |
| miR-941 | 0.808 | 0.208 | -0.570 | 2.39E-03 |
| miR-501-5p | -1.698 | -3.123 | -4.891 | 2.64E-03 |
| miR-23b-3p | -1.669 | -0.269 | -0.372 | 3.11E-03 |
| miR-7704 | 1.715 | 0.181 | -0.909 | 3.64E-03 |
| miR-133a-3p | -0.692 | 0.187 | 0.363 | 3.82E-03 |
| miR-139-5p | 0.873 | 0.167 | 0.317 | 3.88E-03 |
| miR-877-5p | 1.926 | 0.302 | 0.403 | 3.93E-03 |
| miR-625-3p | 1.106 | 0.155 | 0.366 | 4.26E-03 |
| miR-127-5p | -2.418 | 0.608 | 1.044 | 4.26E-03 |
| miR-625-5p | -1.274 | 0.037 | -1.825 | 4.43E-03 |
| miR-664a-5p | 1.245 | -0.130 | -0.144 | 5.01E-03 |
| miR-15b-3p | 0.805 | 0.886 | 0.649 | 5.30E-03 |
| miR-299-5p | -1.565 | -0.425 | 0.018 | 6.50E-03 |
| miR-3613-5p | -1.047 | -1.491 | -0.876 | 6.98E-03 |
| miR-6511a-3p | 7.240 | -5.990 | -6.034 | 7.14E-03 |
| miR-1843 | 0.968 | -0.253 | -0.030 | 7.69E-03 |
| miR-26b-5p | -0.751 | 0.251 | 0.154 | 8.02E-03 |
| miR-432-5p | 1.172 | -0.756 | -0.393 | 8.42E-03 |
| let-7b-5p | 0.544 | 0.019 | -0.020 | 9.19E-03 |
| miR-206 | 0.642 | 0.068 | 0.278 | 9.38E-03 |
| miR-24-3p | 0.288 | 0.133 | 0.253 | 9.54E-03 |
| miR-130b-5p | -2.031 | 0.930 | -0.474 | 9.95E-03 |
| miR-598-3p | 0.404 | 0.433 | 0.432 | 1.01E-02 |
| miR-3925-5p | 1.954 | 1.881 | 0.113 | 1.01E-02 |
| miR-337-3p | 0.595 | -0.336 | 0.461 | 1.04E-02 |
| miR-671-5p | -0.817 | -0.106 | -0.129 | 1.07E-02 |
| miR-190a-5p | -1.639 | 0.108 | -0.240 | 1.12E-02 |
| miR-454-3p | -1.359 | -0.192 | -0.159 | 1.18E-02 |
| miR-329-3p | 1.450 | -1.176 | -0.766 | 1.33E-02 |
| miR-889-3p | -0.411 | -0.457 | -0.173 | 1.67E-02 |
| miR-99b-5p | -0.909 | -0.082 | 0.099 | 1.72E-02 |
| miR-145-5p | 1.083 | -0.314 | 0.304 | 1.73E-02 |
| miR-125a-5p | -0.396 | -0.126 | -0.075 | 1.77E-02 |
| miR-377-3p | -0.161 | -0.644 | -0.312 | 1.78E-02 |
| miR-99b-3p | 0.506 | 0.153 | 0.564 | 1.79E-02 |
| miR-376b-3p | -0.641 | -0.213 | -0.235 | 1.94E-02 |
| miR-140-5p | 0.499 | 0.429 | 0.328 | 2.07E-02 |
| miR-493-3p | 0.811 | -0.299 | -0.026 | 2.23E-02 |
| miR-30d-5p | -0.562 | 0.151 | 0.311 | 2.30E-02 |
| miR-296-5p | -0.576 | -0.218 | -0.725 | 2.33E-02 |
| miR-193a-5p | 0.493 | 0.259 | 0.272 | 2.45E-02 |
| miR-491-5p | -0.581 | -0.346 | -0.441 | 2.47E-02 |
| miR-34c-5p | -0.467 | -0.339 | -0.311 | 2.47E-02 |
| miR-9-5p | -1.056 | -1.342 | 0.146 | 2.69E-02 |
| miR-301a-3p | 0.786 | 0.232 | -0.307 | 2.81E-02 |
| miR-129-5p | 2.202 | -0.548 | -0.299 | 2.87E-02 |
| miR-628-5p | -0.504 | -0.243 | -0.227 | 2.95E-02 |
| miR-412-5p | -1.545 | -0.206 | 1.017 | 3.01E-02 |
| miR-376c-3p | -0.168 | -0.449 | -0.304 | 3.14E-02 |
| miR-1307-3p | -0.855 | 0.019 | 0.158 | 3.33E-02 |
| miR-769-5p | -0.710 | 0.077 | 0.198 | 3.55E-02 |
| miR-1271-5p | 0.399 | 0.174 | 0.659 | 3.63E-02 |
| miR-204-5p | 0.578 | 0.022 | -0.005 | 3.73E-02 |
| miR-1287-5p | 0.579 | 0.334 | 0.486 | 3.75E-02 |
| miR-296-3p | 0.010 | 2.418 | 1.720 | 3.77E-02 |
| miR-125b-1-3p | -0.827 | 0.121 | 0.401 | 4.00E-02 |
| miR-500a-5p | -0.560 | -0.935 | -1.763 | 4.63E-02 |
| miR-378a-3p | -0.722 | 0.333 | 0.206 | 4.71E-02 |
| miR-1185-5p | -0.998 | -0.180 | -0.125 | 4.75E-02 |
| miR-543 | 1.108 | -1.484 | -1.201 | 4.88E-02 |
| miR-340-5p | -0.494 | -0.019 | 0.093 | 5.03E-02 |
| miR-485-5p | -0.515 | -1.649 | -1.116 | 5.47E-02 |
| miR-378i | -2.812 | 2.889 | 0.242 | 5.65E-02 |
| miR-361-3p | -0.683 | -0.355 | 0.225 | 6.54E-02 |
| miR-331-3p | -0.769 | -0.453 | -1.436 | 6.95E-02 |
| miR-409-5p | -0.552 | -0.419 | 0.101 | 7.13E-02 |
| miR-361-5p | 0.414 | -0.054 | -0.236 | 7.36E-02 |
| let-7i-5p | -0.288 | -0.203 | -0.073 | 7.83E-02 |
| miR-143-3p | 0.891 | -0.772 | -0.409 | 7.86E-02 |
| miR-154-5p | 0.529 | -0.184 | -0.269 | 7.90E-02 |
| miR-3615 | 0.355 | 0.482 | 0.196 | 7.92E-02 |
| miR-218-5p | -1.164 | 0.388 | 0.513 | 7.97E-02 |
| miR-93-5p | 0.139 | 0.366 | 0.316 | 8.01E-02 |
| miR-299-3p | 0.285 | -1.530 | -0.931 | 8.16E-02 |
| miR-21-3p | -0.855 | 0.895 | 0.828 | 8.69E-02 |
| miR-146b-3p | -1.104 | -0.046 | 0.287 | 9.08E-02 |
| miR-425-3p | 0.199 | -0.043 | 0.899 | 9.30E-02 |
| miR-505-3p | 0.328 | 0.198 | 0.560 | 9.55E-02 |
| miR-214-3p | -0.340 | -0.020 | -0.834 | 9.62E-02 |
| miR-3605-3p | -0.136 | 1.283 | 0.944 | 9.86E-02 |
| miR-487b-3p | 0.736 | -1.155 | -0.545 | 1.02E-01 |
| miR-758-3p | -0.968 | -0.622 | 0.825 | 1.04E-01 |
| miR-182-5p | 0.107 | 1.285 | 0.869 | 1.05E-01 |
| miR-193b-5p | 0.971 | -0.218 | -0.344 | 1.06E-01 |
| miR-671-3p | 1.435 | -0.418 | -0.505 | 1.08E-01 |
| miR-499a-5p | -0.307 | -0.367 | 0.980 | 1.13E-01 |
| miR-450a-2-3p | 0.492 | 0.633 | -0.558 | 1.16E-01 |
| miR-1180-3p | 0.802 | -0.406 | -0.874 | 1.31E-01 |
| miR-214-5p | -0.489 | -0.177 | -1.009 | 1.32E-01 |
| miR-21-5p | -0.162 | -0.160 | -0.149 | 1.34E-01 |
| miR-10b-3p | 0.769 | 0.324 | 0.193 | 1.40E-01 |
| miR-210-3p | -0.956 | 0.321 | 1.241 | 1.42E-01 |
| miR-433-3p | 0.610 | 0.463 | -0.081 | 1.43E-01 |
| miR-654-3p | 0.716 | -0.311 | -0.174 | 1.49E-01 |
| miR-335-3p | -1.120 | 0.172 | 0.413 | 1.53E-01 |
| miR-30c-2-3p | -0.434 | -0.799 | -0.755 | 1.56E-01 |
| miR-208a-3p | -0.906 | -0.613 | 0.777 | 1.63E-01 |
| miR-423-3p | 0.469 | -0.122 | 0.057 | 1.68E-01 |
| miR-874-3p | 0.151 | 0.713 | 0.993 | 1.72E-01 |
| miR-450a-5p | -0.867 | -0.561 | -0.387 | 1.73E-01 |
| miR-28-3p | 0.125 | 0.240 | 0.182 | 1.78E-01 |
| miR-190b-5p | -0.490 | -0.280 | 0.411 | 1.80E-01 |
| miR-122-5p | -1.774 | 2.962 | -0.093 | 1.86E-01 |
| miR-148a-3p | 1.458 | -0.346 | -0.384 | 1.87E-01 |
| miR-139-3p | 0.353 | 0.089 | 0.087 | 1.91E-01 |
| miR-4705 | -0.774 | -1.138 | -0.558 | 1.92E-01 |
| miR-382-3p | 1.047 | -0.757 | -0.286 | 1.99E-01 |
| miR-501-3p | 0.593 | -0.283 | -0.309 | 2.02E-01 |
| miR-369-3p | -0.301 | -0.365 | -0.095 | 2.05E-01 |
| miR-370-3p | 0.403 | -0.346 | 0.178 | 2.12E-01 |
| miR-30e-3p | 0.109 | 0.286 | 0.178 | 2.27E-01 |
| miR-615-3p | 0.259 | 0.212 | 0.196 | 2.30E-01 |
| miR-500a-3p | -0.223 | -0.039 | -0.137 | 2.33E-01 |
| miR-345-5p | -1.155 | 1.089 | 1.769 | 2.38E-01 |
| miR-455-5p | 0.343 | 0.108 | -0.263 | 2.41E-01 |
| miR-486-5p | 0.202 | 0.369 | 0.342 | 2.69E-01 |
| miR-542-5p | -0.029 | 0.323 | 0.643 | 2.80E-01 |
| miR-106b-3p | 0.524 | -0.621 | -0.645 | 2.83E-01 |
| miR-450b-5p | 0.028 | -0.419 | -0.387 | 2.85E-01 |
| miR-186-5p | 0.094 | 0.206 | 0.252 | 3.00E-01 |
| miR-199b-3p | 0.918 | -0.195 | -0.249 | 3.07E-01 |
| miR-19a-3p | -0.326 | 0.278 | -0.137 | 3.08E-01 |
| miR-199a-3p | 0.918 | -0.195 | -0.249 | 3.08E-01 |
| miR-369-5p | -0.010 | -0.419 | -0.282 | 3.12E-01 |
| miR-324-5p | 0.384 | -0.124 | 0.261 | 3.14E-01 |
| miR-339-5p | -0.185 | -0.559 | 0.189 | 3.21E-01 |
| miR-431-5p | -0.236 | -0.383 | -0.529 | 3.29E-01 |
| miR-504-5p | -0.800 | -0.504 | 0.134 | 3.44E-01 |
| miR-455-3p | -0.091 | -1.925 | -0.881 | 3.45E-01 |
| miR-503-3p | -0.824 | 0.241 | 0.690 | 3.47E-01 |
| miR-425-5p | -0.050 | -0.245 | -0.432 | 3.53E-01 |
| miR-134-5p | 0.691 | -0.685 | -0.280 | 3.55E-01 |
| miR-181a-5p | 0.048 | -0.288 | -0.498 | 3.59E-01 |
| miR-450a-1-3p | -0.424 | -0.119 | -0.063 | 3.61E-01 |
| miR-887-3p | -0.526 | 0.946 | 0.959 | 3.66E-01 |
| miR-136-5p | -0.495 | -0.160 | 0.073 | 3.73E-01 |
| miR-502-3p | -0.360 | 0.142 | 0.062 | 3.79E-01 |
| miR-194-5p | -0.306 | -0.182 | 0.008 | 3.82E-01 |
| miR-378d | -0.572 | -0.126 | -0.247 | 3.85E-01 |
| miR-199b-5p | -1.392 | -0.138 | 0.003 | 3.91E-01 |
| miR-493-5p | 0.442 | -0.362 | -0.277 | 4.01E-01 |
| miR-132-3p | 1.088 | -0.577 | -0.272 | 4.04E-01 |
| miR-487a-3p | 0.689 | -1.863 | -1.139 | 4.06E-01 |
| miR-146b-5p | -0.319 | -0.315 | -0.561 | 4.09E-01 |
| miR-18a-5p | 0.337 | 0.179 | -0.026 | 4.11E-01 |
| miR-1296-5p | 0.375 | -0.005 | 0.254 | 4.18E-01 |
| miR-133b | -0.248 | 0.052 | 0.151 | 4.24E-01 |
| miR-222-3p | 0.495 | -0.259 | -0.400 | 4.27E-01 |
| miR-188-5p | -0.094 | -0.131 | 0.513 | 4.34E-01 |
| miR-125b-5p | -0.349 | 0.105 | 0.177 | 4.37E-01 |
| miR-483-3p | -0.786 | 2.532 | 0.753 | 4.52E-01 |
| miR-185-5p | 0.084 | 0.235 | 0.155 | 4.55E-01 |
| miR-539-3p | 0.464 | -0.699 | -0.436 | 4.59E-01 |
| miR-335-5p | -0.077 | 0.685 | 0.373 | 4.74E-01 |
| miR-532-5p | 0.188 | -0.071 | 0.092 | 4.86E-01 |
| miR-495-3p | 0.296 | -0.851 | -0.565 | 4.93E-01 |
| miR-424-3p | 0.427 | -0.277 | -0.049 | 5.07E-01 |
| miR-4662a-5p | 0.410 | 0.209 | -0.940 | 5.24E-01 |
| miR-193b-3p | -0.599 | 0.522 | 1.206 | 5.28E-01 |
| miR-221-3p | 0.520 | -0.287 | -0.308 | 5.28E-01 |
| miR-3065-5p | 0.521 | 0.090 | -0.024 | 5.37E-01 |
| miR-381-3p | -0.051 | -0.446 | -0.073 | 5.66E-01 |
| miR-744-5p | 0.318 | -0.169 | 0.005 | 5.70E-01 |
| miR-183-5p | 0.309 | -0.101 | 0.668 | 5.93E-01 |
| miR-152-3p | 0.224 | -0.164 | 0.029 | 5.97E-01 |
| miR-140-3p | -0.757 | 0.259 | 1.131 | 5.98E-01 |
| miR-203a-3p | -0.411 | 1.202 | 0.292 | 6.12E-01 |
| miR-451a | 3.217 | -4.307 | -2.421 | 6.18E-01 |
| miR-223-3p | 5.543 | -6.171 | -4.899 | 6.37E-01 |
| miR-532-3p | -0.304 | -0.003 | 0.051 | 6.50E-01 |
| miR-652-3p | -0.959 | -0.160 | 0.203 | 6.52E-01 |
| miR-199a-5p | -0.339 | -0.120 | 0.001 | 6.63E-01 |
| miR-376a-3p | 0.335 | -0.367 | -0.057 | 6.70E-01 |
| miR-495-5p | 0.110 | -0.121 | -0.848 | 6.82E-01 |
| miR-29b-3p | -0.080 | -0.378 | -0.115 | 6.83E-01 |
| miR-146a-5p | 0.806 | -0.584 | 0.637 | 6.84E-01 |
| miR-148b-3p | 0.135 | 0.091 | -0.184 | 7.02E-01 |
| miR-675-5p | -0.171 | 1.066 | 0.035 | 7.03E-01 |
| miR-208b-3p | -0.240 | -0.487 | 0.421 | 7.06E-01 |
| miR-10a-5p | -0.070 | -0.101 | -0.182 | 7.13E-01 |
| miR-196b-5p | 0.102 | -0.579 | -0.147 | 7.21E-01 |
| miR-30b-5p | -0.180 | 0.419 | 0.224 | 7.46E-01 |
| miR-221-5p | -0.535 | 0.256 | 0.408 | 7.54E-01 |
| miR-339-3p | -0.398 | 0.308 | 0.611 | 7.73E-01 |
| miR-2110 | -0.372 | -0.074 | 0.311 | 7.85E-01 |
| miR-424-5p | 0.198 | -0.030 | 0.054 | 8.01E-01 |
| miR-126-5p | -0.112 | -0.520 | 0.506 | 8.07E-01 |
| miR-494-3p | 0.262 | -0.201 | -0.088 | 8.10E-01 |
| miR-10b-5p | -0.255 | -0.085 | -0.116 | 8.18E-01 |
| miR-29c-3p | -0.125 | 0.338 | 0.226 | 8.21E-01 |
| miR-100-5p | 0.231 | -0.360 | -0.400 | 8.32E-01 |
| miR-20a-5p | -0.088 | 0.402 | 0.141 | 8.37E-01 |
| miR-656-3p | 0.427 | -0.996 | -0.786 | 8.40E-01 |
| miR-138-5p | 0.306 | -0.223 | -0.049 | 8.42E-01 |
| miR-95-3p | -0.288 | 0.263 | 0.147 | 8.48E-01 |
| miR-664a-3p | -0.163 | 0.275 | -0.274 | 8.58E-01 |
| miR-365b-3p | -0.147 | 0.096 | -0.061 | 8.61E-01 |
| let-7g-5p | -0.038 | 0.188 | 0.044 | 8.64E-01 |
| miR-1301-3p | 0.357 | -0.099 | -0.295 | 8.65E-01 |
| miR-101-3p | -0.139 | 0.217 | 0.197 | 8.72E-01 |
| miR-137-3p | -0.034 | -0.141 | 0.241 | 8.74E-01 |
| miR-151a-5p | -0.114 | 0.032 | -0.229 | 8.76E-01 |
| miR-1307-5p | -0.573 | 0.492 | 0.654 | 8.78E-01 |
| miR-337-5p | 0.071 | -0.150 | 0.157 | 8.78E-01 |
| miR-27a-3p | 0.085 | -0.113 | 0.133 | 8.91E-01 |
| miR-19b-3p | -0.140 | 0.038 | 0.107 | 9.05E-01 |
| miR-106b-5p | 0.189 | 0.240 | -0.394 | 9.06E-01 |
| miR-142-3p | -0.631 | 0.245 | 1.827 | 9.11E-01 |
| miR-32-5p | 0.206 | -0.261 | -0.156 | 9.18E-01 |
| miR-196a-5p | 0.272 | -0.102 | 0.030 | 9.32E-01 |
| miR-548a-3p | -0.125 | -0.095 | 0.724 | 9.33E-01 |
| miR-29a-3p | 0.079 | 0.018 | 0.067 | 9.38E-01 |
| miR-16-5p | 0.241 | -0.120 | -0.255 | 9.57E-01 |
| miR-30a-5p | -0.166 | 0.134 | 0.119 | 9.63E-01 |
| miR-655-3p | 0.112 | 0.065 | -0.046 | 9.65E-01 |
| miR-1-5p | -0.025 | 0.350 | 0.107 | 9.70E-01 |
| miR-496 | 0.172 | -0.362 | -0.048 | 9.75E-01 |
| miR-365a-3p | -0.033 | -0.018 | -0.075 | 9.79E-01 |
| miR-155-5p | 0.185 | -0.176 | -0.155 | 9.79E-01 |
| miR-33a-5p | 0.152 | -0.141 | -0.319 | 9.82E-01 |
| let-7c-5p | 0.022 | -0.043 | -0.086 | 9.94E-01 |
| miR-660-5p | 0.041 | -0.036 | -0.040 | 9.98E-01 |

Abbreviations: miR: microRNA; CTL, control; CLFS, chronic low-frequency stimulation; IHFS, intermittent high-frequency stimulation; EV, extracellular vesicle; MB, myobundle. These data are the differential expression analysis (QLF-test) between populations with all treatments in the model. 152 miRs were identified as differentially expressed (*p*<0.05).
