# Supplementary material for: Differential microRNA profiles of intramuscular and secreted extracellular vesicles in human tissue-engineered muscle
Source: Front Physiol. 2022 Aug 25;13:937899. doi: 10.3389/fphys.2022.937899 (PMC9452896; doi:10.3389/fphys.2022.937899)
Supplement: Supplementary file 2 [file Table2.docx]

| **Pathway** | ***P*-value** | **Expected** | **Observed** | **Observed miRs in pathway** |
| --- | --- | --- | --- | --- |
| PI3K-Akt signaling pathway | 2.94E-03 | 133 | 143 | 27a-5p; 133a-5p; 107; 483-5p; 342-3p; 590-3p; 378a-5p; 1185-1-3p; 30a-3p; let-7e-5p; 362-5p; 320c; 320d; 92a-3p; let-7d-5p; 542-3p; 100-3p; 374a-5p; 31-5p; 132-5p; let-7a-5p; 197-3p; 320b; 22-3p; 486-3p; 25-3p; 149-5p; 128-3p; 126-3p; 99a-5p; 323b-3p; 205-5p; 1306-5p; 1-3p; 323a-3p; 379-5p; 374a-3p; 30e-5p; 130b-3p; 574-3p; 30c-5p; 485-3p; 98-5p; let-7f-5p; 15a-5p; 34a-5p; 330-5p; 7-5p; 411-5p; 665; 576-5p; 15b-5p; 103a-3p; let-7a-3p; 191-5p; 127-3p; 151a-3p; 7706; 184; 409-3p; 320a-3p; 382-5p; 130a-3p; 27b-3p; 423-5p; 374b-5p; 628-3p; 181b-5p; 584-5p; 23a-3p; 22-5p; 92b-3p; 136-3p; 93-3p; 17-5p; 484; 192-5p; 421; 497-5p; 154-3p; 26a-5p; 328-3p; 708-5p; 503-5p; 675-3p; 195-5p; 941; 501-5p; 23b-3p; 7704; 133a-3p; 139-5p; 877-5p; 625-3p; 127-5p; 625-5p; 664a-5p; 15b-3p; 299-5p; 3613-5p; 26b-5p; 432-5p; let-7b-5p; 206; 24-3p; 130b-5p; 598-3p; 3925-5p; 337-3p; 671-5p; 190a-5p; 454-3p; 329-3p; 889-3p; 99b-5p; 145-5p; 125a-5p; 377-3p; 99b-3p; 376b-3p; 140-5p; 493-3p; 30d-5p; 296-5p; 193a-5p; 491-5p; 34c-5p; 9-5p; 301a-3p; 129-5p; 628-5p; 376c-3p; 1307-3p; 769-5p; 1271-5p; 204-5p; 1287-5p; 296-3p; 125b-1-3p; 500a-5p; 378a-3p; 1185-5p; 543 |
| Viral carcinogenesis | 1.67E-04 | 124 | 139 | 133a-5p; 107; 483-5p; 342-3p; 590-3p; 378a-5p; 1185-1-3p; 30a-3p; let-7e-5p; 362-5p; 320c; 320d; 92a-3p; let-7d-5p; 542-3p; 100-3p; 374a-5p; 31-5p; 132-5p; let-7a-5p; 197-3p; 320b; 22-3p; 486-3p; 25-3p; 149-5p; 128-3p; 126-3p; 99a-5p; 323b-3p; 205-5p; 1306-5p; 629-5p; 1-3p; 323a-3p; 379-5p; 374a-3p; 30e-5p; 130b-3p; 574-3p; 30c-5p; 485-3p; 98-5p; let-7f-5p; 15a-5p; 34a-5p; 330-5p; 7-5p; 411-5p; 665; 576-5p; 15b-5p; 103a-3p; let-7a-3p; 191-5p; 151a-3p; 7706; 184; 409-3p; 320a-3p; 382-5p; 130a-3p; 27b-3p; 423-5p; 374b-5p; 628-3p; 181b-5p; 584-5p; 23a-3p; 22-5p; 92b-3p; 136-3p; 93-3p; 17-5p; 484; 192-5p; 421; 497-5p; 154-3p; 26a-5p; 328-3p; 708-5p; 503-5p; 195-5p; 941; 501-5p; 23b-3p; 7704; 133a-3p; 139-5p; 877-5p; 127-5p; 625-5p; 15b-3p; 299-5p; 3613-5p; 6511a-3p; 26b-5p; 432-5p; let-7b-5p; 206; 24-3p; 130b-5p; 598-3p; 3925-5p; 337-3p; 671-5p; 190a-5p; 454-3p; 329-3p; 99b-5p; 145-5p; 125a-5p; 377-3p; 99b-3p; 376b-3p; 140-5p; 493-3p; 30d-5p; 296-5p; 193a-5p; 491-5p; 34c-5p; 9-5p; 301a-3p; 129-5p; 628-5p; 376c-3p; 1307-3p; 769-5p; 1271-5p; 204-5p; 1287-5p; 296-3p; 125b-1-3p; 500a-5p; 378a-3p; 1185-5p; 543 |
| FoxO signaling pathway | 6.19E-06 | 119 | 138 | 27a-5p; 133a-5p; 107; 483-5p; 342-3p; 590-3p; 378a-5p; 1185-1-3p; 30a-3p; let-7e-5p; 362-5p; 92a-3p; let-7d-5p; 542-3p; 100-3p; 374a-5p; 31-5p; 132-5p; let-7a-5p; 197-3p; 320b; 22-3p; 486-3p; 25-3p; 149-5p; 181a-2-3p; 128-3p; 126-3p; 99a-5p; 323b-3p; 205-5p; 1306-5p; 629-5p; 1-3p; 323a-3p; 379-5p; 374a-3p; 30e-5p; 130b-3p; 574-3p; 30c-5p; 485-3p; 98-5p; let-7f-5p; 15a-5p; 34a-5p; 330-5p; 7-5p; 411-5p; 665; 576-5p; 15b-5p; 103a-3p; let-7a-3p; 191-5p; 127-3p; 151a-3p; 7706; 184; 409-3p; 320a-3p; 382-5p; 130a-3p; 27b-3p; 423-5p; 374b-5p; 628-3p; 181b-5p; 584-5p; 23a-3p; 92b-3p; 136-3p; 93-3p; 17-5p; 484; 192-5p; 421; 497-5p; 154-3p; 26a-5p; 708-5p; 503-5p; 675-3p; 195-5p; 941; 501-5p; 23b-3p; 7704; 133a-3p; 139-5p; 877-5p; 625-3p; 127-5p; 625-5p; 664a-5p; 15b-3p; 299-5p; 3613-5p; 26b-5p; 432-5p; let-7b-5p; 206; 24-3p; 130b-5p; 3925-5p; 337-3p; 671-5p; 190a-5p; 454-3p; 329-3p; 889-3p; 99b-5p; 145-5p; 125a-5p; 377-3p; 376b-3p; 140-5p; 30d-5p; 296-5p; 193a-5p; 491-5p; 34c-5p; 9-5p; 301a-3p; 129-5p; 628-5p; 376c-3p; 1307-3p; 769-5p; 1271-5p; 204-5p; 1287-5p; 296-3p; 125b-1-3p; 500a-5p; 378a-3p; 1185-5p; 543 |
| Proteoglycans in cancer | 9.43E-04 | 125 | 138 | 27a-5p; 133a-5p; 107; 483-5p; 342-3p; 181a-3p; 590-3p; 378a-5p; 1185-1-3p; 30a-3p; let-7e-5p; 362-5p; 320c; 320d; 92a-3p; let-7d-5p; 542-3p; 100-3p; 374a-5p; 31-5p; let-7a-5p; 197-3p; 320b; 22-3p; 486-3p; 25-3p; 149-5p; 181a-2-3p; 128-3p; 126-3p; 99a-5p; 323b-3p; 205-5p; 1306-5p; 1-3p; 323a-3p; 379-5p; 374a-3p; 30e-5p; 130b-3p; 574-3p; 30c-5p; 485-3p; 98-5p; let-7f-5p; 15a-5p; 34a-5p; 330-5p; 7-5p; 411-5p; 665; 576-5p; 15b-5p; 103a-3p; let-7a-3p; 191-5p; 151a-3p; 7706; 184; 409-3p; 320a-3p; 382-5p; let-7i-3p; 130a-3p; 27b-3p; 423-5p; 374b-5p; 181b-5p; 584-5p; 23a-3p; 22-5p; 92b-3p; 136-3p; 93-3p; 17-5p; 484; 192-5p; 421; 497-5p; 154-3p; 26a-5p; 328-3p; 708-5p; 503-5p; 675-3p; 195-5p; 23b-3p; 7704; 133a-3p; 139-5p; 877-5p; 625-3p; 127-5p; 625-5p; 664a-5p; 15b-3p; 299-5p; 6511a-3p; 26b-5p; 432-5p; let-7b-5p; 206; 24-3p; 130b-5p; 3925-5p; 337-3p; 671-5p; 190a-5p; 454-3p; 329-3p; 889-3p; 99b-5p; 145-5p; 125a-5p; 377-3p; 99b-3p; 376b-3p; 140-5p; 493-3p; 30d-5p; 296-5p; 193a-5p; 491-5p; 34c-5p; 9-5p; 301a-3p; 129-5p; 376c-3p; 1307-3p; 769-5p; 1271-5p; 204-5p; 296-3p; 125b-1-3p; 500a-5p; 378a-3p; 1185-5p; 543 |
| Human papillomavirus infection | 1.27E-02 | 129 | 138 | 27a-5p; 133a-5p; 107; 483-5p; 342-3p; 590-3p; 378a-5p; 1185-1-3p; 30a-3p; let-7e-5p; 362-5p; 320c; 320d; 92a-3p; let-7d-5p; 542-3p; 374a-5p; 31-5p; 132-5p; let-7a-5p; 197-3p; 320b; 22-3p; 486-3p; 25-3p; 149-5p; 128-3p; 126-3p; 99a-5p; 323b-3p; 205-5p; 1306-5p; 629-5p; 1-3p; 323a-3p; 379-5p; 374a-3p; 30e-5p; 130b-3p; 574-3p; 30c-5p; 485-3p; 98-5p; let-7f-5p; 15a-5p; 34a-5p; 330-5p; 7-5p; 411-5p; 665; 576-5p; 15b-5p; 103a-3p; let-7a-3p; 191-5p; 151a-3p; 7706; 184; 409-3p; 320a-3p; 382-5p; 130a-3p; 27b-3p; 423-5p; 374b-5p; 628-3p; 181b-5p; 584-5p; 23a-3p; 92b-3p; 136-3p; 93-3p; 17-5p; 484; 192-5p; 421; 497-5p; 154-3p; 26a-5p; 328-3p; 708-5p; 503-5p; 675-3p; 195-5p; 941; 501-5p; 23b-3p; 7704; 133a-3p; 139-5p; 877-5p; 625-3p; 127-5p; 625-5p; 664a-5p; 15b-3p; 299-5p; 3613-5p; 26b-5p; 432-5p; let-7b-5p; 206; 24-3p; 130b-5p; 3925-5p; 671-5p; 454-3p; 329-3p; 889-3p; 99b-5p; 145-5p; 125a-5p; 377-3p; 99b-3p; 376b-3p; 140-5p; 493-3p; 30d-5p; 296-5p; 193a-5p; 491-5p; 34c-5p; 9-5p; 301a-3p; 129-5p; 628-5p; 376c-3p; 1307-3p; 769-5p; 1271-5p; 204-5p; 1287-5p; 296-3p; 125b-1-3p; 500a-5p; 378a-3p; 1185-5p; 543 |
| MAPK signaling pathway | 1.53E-02 | 129 | 138 | 27a-5p; 133a-5p; 107; 483-5p; 342-3p; 590-3p; 378a-5p; 1185-1-3p; 30a-3p; let-7e-5p; 362-5p; 320c; 320d; 92a-3p; let-7d-5p; 542-3p; 100-3p; 374a-5p; 31-5p; let-7a-5p; 197-3p; 320b; 22-3p; 486-3p; 25-3p; 149-5p; 181a-2-3p; 128-3p; 126-3p; 99a-5p; 323b-3p; 205-5p; 1306-5p; 1-3p; 323a-3p; 379-5p; 374a-3p; 30e-5p; 130b-3p; 574-3p; 30c-5p; 485-3p; 98-5p; let-7f-5p; 15a-5p; 34a-5p; 330-5p; 7-5p; 411-5p; 665; 576-5p; 15b-5p; 103a-3p; let-7a-3p; 191-5p; 151a-3p; 7706; 184; 409-3p; 320a-3p; 382-5p; 130a-3p; 27b-3p; 423-5p; 374b-5p; 181b-5p; 584-5p; 23a-3p; 22-5p; 92b-3p; 93-3p; 17-5p; 484; 192-5p; 421; 497-5p; 154-3p; 26a-5p; 328-3p; 708-5p; 503-5p; 675-3p; 195-5p; 941; 501-5p; 23b-3p; 7704; 133a-3p; 139-5p; 877-5p; 625-3p; 127-5p; 625-5p; 664a-5p; 15b-3p; 6511a-3p; 26b-5p; 432-5p; let-7b-5p; 206; 24-3p; 130b-5p; 3925-5p; 337-3p; 671-5p; 190a-5p; 454-3p; 329-3p; 889-3p; 99b-5p; 145-5p; 125a-5p; 377-3p; 99b-3p; 376b-3p; 140-5p; 493-3p; 30d-5p; 296-5p; 193a-5p; 491-5p; 34c-5p; 9-5p; 301a-3p; 129-5p; 628-5p; 376c-3p; 1307-3p; 769-5p; 1271-5p; 204-5p; 1287-5p; 296-3p; 125b-1-3p; 500a-5p; 378a-3p; 1185-5p; 543 |
| MicroRNAs in cancer | 8.60E-03 | 127 | 137 | 27a-5p; 133a-5p; 107; 483-5p; 342-3p; 590-3p; 378a-5p; 1185-1-3p; 30a-3p; let-7e-5p; 362-5p; 320c; 320d; 92a-3p; let-7d-5p; 542-3p; 100-3p; 374a-5p; 31-5p; let-7a-5p; 197-3p; 320b; 22-3p; 486-3p; 25-3p; 149-5p; 181a-2-3p; 128-3p; 126-3p; 99a-5p; 323b-3p; 205-5p; 1306-5p; 629-5p; 1-3p; 323a-3p; 379-5p; 30e-5p; 130b-3p; 574-3p; 30c-5p; 485-3p; 98-5p; let-7f-5p; 15a-5p; 34a-5p; 330-5p; 7-5p; 411-5p; 665; 576-5p; 15b-5p; 103a-3p; let-7a-3p; 191-5p; 151a-3p; 7706; 184; 409-3p; 320a-3p; 382-5p; 130a-3p; 27b-3p; 423-5p; 374b-5p; 628-3p; 181b-5p; 584-5p; 23a-3p; 22-5p; 92b-3p; 136-3p; 93-3p; 17-5p; 484; 192-5p; 421; 497-5p; 154-3p; 26a-5p; 328-3p; 708-5p; 503-5p; 675-3p; 195-5p; 501-5p; 23b-3p; 7704; 133a-3p; 139-5p; 877-5p; 127-5p; 625-5p; 664a-5p; 15b-3p; 299-5p; 3613-5p; 6511a-3p; 26b-5p; 432-5p; let-7b-5p; 206; 24-3p; 130b-5p; 3925-5p; 337-3p; 671-5p; 454-3p; 329-3p; 889-3p; 99b-5p; 145-5p; 125a-5p; 377-3p; 99b-3p; 376b-3p; 140-5p; 30d-5p; 296-5p; 193a-5p; 491-5p; 34c-5p; 9-5p; 301a-3p; 129-5p; 628-5p; 376c-3p; 1307-3p; 769-5p; 1271-5p; 204-5p; 1287-5p; 296-3p; 125b-1-3p; 378a-3p; 1185-5p; 543 |
| Cellular senescence | 5.35E-04 | 119 | 134 | 27a-5p; 133a-5p; 107; 483-5p; 342-3p; 590-3p; 378a-5p; 1185-1-3p; 30a-3p; let-7e-5p; 362-5p; 320c; 92a-3p; let-7d-5p; 542-3p; 100-3p; 374a-5p; 31-5p; 132-5p; let-7a-5p; 197-3p; 320b; 22-3p; 486-3p; 25-3p; 149-5p; 181a-2-3p; 128-3p; 126-3p; 99a-5p; 323b-3p; 205-5p; 1306-5p; 1-3p; 323a-3p; 379-5p; 374a-3p; 30e-5p; 130b-3p; 574-3p; 30c-5p; 485-3p; 98-5p; let-7f-5p; 15a-5p; 34a-5p; 330-5p; 7-5p; 411-5p; 665; 576-5p; 15b-5p; 103a-3p; let-7a-3p; 191-5p; 151a-3p; 7706; 184; 409-3p; 320a-3p; 382-5p; 130a-3p; 27b-3p; 423-5p; 374b-5p; 181b-5p; 584-5p; 23a-3p; 92b-3p; 93-3p; 17-5p; 484; 192-5p; 421; 497-5p; 154-3p; 26a-5p; 708-5p; 503-5p; 195-5p; 941; 23b-3p; 7704; 133a-3p; 139-5p; 877-5p; 625-3p; 127-5p; 625-5p; 15b-3p; 299-5p; 3613-5p; 26b-5p; 432-5p; let-7b-5p; 206; 24-3p; 130b-5p; 598-3p; 3925-5p; 337-3p; 671-5p; 190a-5p; 454-3p; 329-3p; 889-3p; 99b-5p; 145-5p; 125a-5p; 377-3p; 99b-3p; 376b-3p; 140-5p; 493-3p; 30d-5p; 193a-5p; 491-5p; 34c-5p; 9-5p; 301a-3p; 129-5p; 628-5p; 376c-3p; 1307-3p; 769-5p; 1271-5p; 204-5p; 1287-5p; 296-3p; 125b-1-3p; 500a-5p; 378a-3p; 1185-5p; 543 |
| Rap1 signaling pathway | 6.66E-04 | 120 | 134 | 27a-5p; 133a-5p; 107; 483-5p; 342-3p; 590-3p; 378a-5p; 30a-3p; let-7e-5p; 362-5p; 320c; 320d; 92a-3p; let-7d-5p; 542-3p; 374a-5p; 31-5p; let-7a-5p; 197-3p; 320b; 22-3p; 486-3p; 25-3p; 149-5p; 181a-2-3p; 128-3p; 126-3p; 99a-5p; 323b-3p; 205-5p; 1306-5p; 629-5p; 1-3p; 374a-3p; 30e-5p; 130b-3p; 574-3p; 30c-5p; 98-5p; let-7f-5p; 15a-5p; 34a-5p; 330-5p; 7-5p; 411-5p; 665; 576-5p; 15b-5p; 103a-3p; let-7a-3p; 191-5p; 151a-3p; 7706; 184; 409-3p; 320a-3p; 382-5p; let-7i-3p; 130a-3p; 27b-3p; 423-5p; 374b-5p; 181b-5p; 584-5p; 23a-3p; 92b-3p; 93-3p; 17-5p; 484; 192-5p; 421; 497-5p; 154-3p; 26a-5p; 328-3p; 708-5p; 503-5p; 675-3p; 195-5p; 941; 501-5p; 23b-3p; 7704; 133a-3p; 139-5p; 877-5p; 625-3p; 127-5p; 625-5p; 664a-5p; 15b-3p; 6511a-3p; 26b-5p; 432-5p; let-7b-5p; 206; 24-3p; 130b-5p; 598-3p; 3925-5p; 337-3p; 671-5p; 190a-5p; 454-3p; 329-3p; 889-3p; 99b-5p; 145-5p; 125a-5p; 377-3p; 99b-3p; 376b-3p; 140-5p; 493-3p; 30d-5p; 296-5p; 193a-5p; 491-5p; 34c-5p; 9-5p; 301a-3p; 129-5p; 628-5p; 376c-3p; 1307-3p; 769-5p; 1271-5p; 204-5p; 296-3p; 125b-1-3p; 500a-5p; 378a-3p; 1185-5p; 543 |
| Focal adhesion | 8.28E-04 | 120 | 134 | 27a-5p; 133a-5p; 107; 483-5p; 342-3p; 590-3p; 378a-5p; 1185-1-3p; 30a-3p; let-7e-5p; 320c; 320d; 92a-3p; let-7d-5p; 542-3p; 100-3p; 374a-5p; 31-5p; let-7a-5p; 197-3p; 320b; 22-3p; 486-3p; 25-3p; 149-5p; 181a-2-3p; 128-3p; 126-3p; 99a-5p; 323b-3p; 205-5p; 1306-5p; 1-3p; 323a-3p; 379-5p; 374a-3p; 30e-5p; 130b-3p; 574-3p; 30c-5p; 485-3p; 98-5p; let-7f-5p; 15a-5p; 34a-5p; 330-5p; 7-5p; 411-5p; 665; 576-5p; 15b-5p; 103a-3p; let-7a-3p; 191-5p; 7706; 184; 409-3p; 320a-3p; 382-5p; let-7i-3p; 130a-3p; 27b-3p; 423-5p; 374b-5p; 181b-5p; 584-5p; 23a-3p; 22-5p; 92b-3p; 136-3p; 93-3p; 17-5p; 484; 192-5p; 421; 497-5p; 154-3p; 26a-5p; 328-3p; 708-5p; 503-5p; 675-3p; 195-5p; 501-5p; 23b-3p; 133a-3p; 139-5p; 877-5p; 625-3p; 127-5p; 625-5p; 664a-5p; 15b-3p; 299-5p; 6511a-3p; 26b-5p; let-7b-5p; 206; 24-3p; 130b-5p; 3925-5p; 337-3p; 671-5p; 190a-5p; 454-3p; 329-3p; 889-3p; 99b-5p; 145-5p; 125a-5p; 377-3p; 99b-3p; 376b-3p; 140-5p; 493-3p; 30d-5p; 296-5p; 193a-5p; 491-5p; 34c-5p; 9-5p; 301a-3p; 129-5p; 628-5p; 376c-3p; 1307-3p; 769-5p; 1271-5p; 204-5p; 1287-5p; 296-3p; 125b-1-3p; 378a-3p; 543 |
| Human cytomegalovirus infection | 2.59E-03 | 122 | 134 | 27a-5p; 133a-5p; 107; 483-5p; 342-3p; 590-3p; 378a-5p; 1185-1-3p; 30a-3p; let-7e-5p; 362-5p; 320c; 320d; 92a-3p; let-7d-5p; 542-3p; 100-3p; 374a-5p; 31-5p; let-7a-5p; 197-3p; 320b; 22-3p; 486-3p; 25-3p; 149-5p; 181a-2-3p; 128-3p; 126-3p; 99a-5p; 323b-3p; 205-5p; 1306-5p; 1-3p; 323a-3p; 379-5p; 374a-3p; 30e-5p; 130b-3p; 574-3p; 30c-5p; 485-3p; 98-5p; let-7f-5p; 15a-5p; 34a-5p; 330-5p; 7-5p; 411-5p; 665; 576-5p; 15b-5p; 103a-3p; let-7a-3p; 191-5p; 151a-3p; 7706; 184; 409-3p; 320a-3p; 382-5p; 130a-3p; 27b-3p; 423-5p; 374b-5p; 181b-5p; 584-5p; 23a-3p; 92b-3p; 93-3p; 17-5p; 484; 192-5p; 421; 497-5p; 154-3p; 26a-5p; 328-3p; 708-5p; 503-5p; 195-5p; 501-5p; 23b-3p; 7704; 133a-3p; 139-5p; 877-5p; 625-3p; 127-5p; 625-5p; 299-5p; 3613-5p; 26b-5p; 432-5p; let-7b-5p; 206; 24-3p; 130b-5p; 598-3p; 3925-5p; 337-3p; 671-5p; 190a-5p; 454-3p; 329-3p; 889-3p; 99b-5p; 145-5p; 125a-5p; 377-3p; 99b-3p; 376b-3p; 140-5p; 493-3p; 30d-5p; 296-5p; 193a-5p; 491-5p; 34c-5p; 9-5p; 301a-3p; 129-5p; 628-5p; 376c-3p; 1307-3p; 769-5p; 1271-5p; 204-5p; 1287-5p; 296-3p; 125b-1-3p; 500a-5p; 378a-3p; 543 |
| Thyroid hormone signaling pathway | 9.33E-09 | 106 | 133 | 27a-5p; 107; 483-5p; 342-3p; 590-3p; 378a-5p; 1185-1-3p; 30a-3p; let-7e-5p; 320c; 320d; 92a-3p; let-7d-5p; 542-3p; 374a-5p; 31-5p; 132-5p; let-7a-5p; 197-3p; 320b; 22-3p; 486-3p; 25-3p; 149-5p; 181a-2-3p; 128-3p; 126-3p; 99a-5p; 323b-3p; 205-5p; 1306-5p; 1-3p; 323a-3p; 379-5p; 374a-3p; 30e-5p; 130b-3p; 574-3p; 30c-5p; 485-3p; 98-5p; let-7f-5p; 15a-5p; 34a-5p; 330-5p; 7-5p; 411-5p; 665; 15b-5p; 103a-3p; let-7a-3p; 191-5p; 151a-3p; 7706; 184; 409-3p; 320a-3p; 382-5p; let-7i-3p; 130a-3p; 27b-3p; 423-5p; 374b-5p; 628-3p; 181b-5p; 584-5p; 23a-3p; 22-5p; 92b-3p; 136-3p; 93-3p; 17-5p; 484; 192-5p; 421; 497-5p; 154-3p; 26a-5p; 328-3p; 708-5p; 503-5p; 195-5p; 941; 501-5p; 23b-3p; 133a-3p; 139-5p; 877-5p; 625-3p; 127-5p; 625-5p; 15b-3p; 299-5p; 6511a-3p; 26b-5p; 432-5p; let-7b-5p; 206; 24-3p; 130b-5p; 3925-5p; 671-5p; 190a-5p; 454-3p; 329-3p; 889-3p; 99b-5p; 145-5p; 125a-5p; 377-3p; 99b-3p; 140-5p; 493-3p; 30d-5p; 296-5p; 193a-5p; 491-5p; 34c-5p; 9-5p; 301a-3p; 129-5p; 376c-3p; 1307-3p; 769-5p; 1271-5p; 204-5p; 1287-5p; 296-3p; 125b-1-3p; 500a-5p; 378a-3p; 1185-5p; 543 |
| Hepatocellular carcinoma | 4.32E-04 | 118 | 133 | 27a-5p; 133a-5p; 107; 483-5p; 342-3p; 590-3p; 378a-5p; 1185-1-3p; 30a-3p; let-7e-5p; 362-5p; 320c; 320d; 92a-3p; let-7d-5p; 542-3p; 100-3p; 374a-5p; 31-5p; 132-5p; let-7a-5p; 197-3p; 320b; 22-3p; 486-3p; 25-3p; 149-5p; 181a-2-3p; 128-3p; 126-3p; 99a-5p; 323b-3p; 205-5p; 1-3p; 323a-3p; 379-5p; 374a-3p; 30e-5p; 130b-3p; 574-3p; 30c-5p; 485-3p; 98-5p; let-7f-5p; 15a-5p; 34a-5p; 330-5p; 7-5p; 411-5p; 665; 576-5p; 15b-5p; 103a-3p; let-7a-3p; 191-5p; 7706; 184; 409-3p; 320a-3p; 382-5p; let-7i-3p; 130a-3p; 27b-3p; 423-5p; 374b-5p; 628-3p; 181b-5p; 584-5p; 23a-3p; 92b-3p; 136-3p; 93-3p; 17-5p; 484; 192-5p; 421; 497-5p; 154-3p; 26a-5p; 328-3p; 708-5p; 503-5p; 675-3p; 195-5p; 501-5p; 23b-3p; 7704; 133a-3p; 139-5p; 877-5p; 625-3p; 127-5p; 625-5p; 15b-3p; 299-5p; 3613-5p; 26b-5p; let-7b-5p; 206; 24-3p; 130b-5p; 3925-5p; 671-5p; 454-3p; 329-3p; 889-3p; 99b-5p; 145-5p; 125a-5p; 377-3p; 99b-3p; 376b-3p; 140-5p; 493-3p; 30d-5p; 193a-5p; 491-5p; 34c-5p; 9-5p; 301a-3p; 129-5p; 628-5p; 376c-3p; 1307-3p; 769-5p; 1271-5p; 204-5p; 1287-5p; 296-3p; 125b-1-3p; 500a-5p; 378a-3p; 543 |
| Shigellosis | 2.63E-02 | 125 | 133 | 27a-5p; 133a-5p; 107; 483-5p; 342-3p; 590-3p; 378a-5p; 1185-1-3p; 30a-3p; let-7e-5p; 362-5p; 320c; 320d; 92a-3p; let-7d-5p; 542-3p; 100-3p; 374a-5p; 31-5p; 132-5p; let-7a-5p; 197-3p; 320b; 22-3p; 486-3p; 25-3p; 149-5p; 181a-2-3p; 128-3p; 126-3p; 99a-5p; 205-5p; 1306-5p; 629-5p; 1-3p; 323a-3p; 379-5p; 374a-3p; 30e-5p; 130b-3p; 574-3p; 30c-5p; 485-3p; 98-5p; let-7f-5p; 15a-5p; 34a-5p; 330-5p; 7-5p; 411-5p; 665; 576-5p; 15b-5p; 103a-3p; let-7a-3p; 127-3p; 151a-3p; 184; 409-3p; 320a-3p; 382-5p; let-7i-3p; 130a-3p; 27b-3p; 423-5p; 374b-5p; 181b-5p; 584-5p; 23a-3p; 92b-3p; 93-3p; 17-5p; 484; 192-5p; 421; 497-5p; 154-3p; 26a-5p; 328-3p; 708-5p; 503-5p; 195-5p; 941; 501-5p; 23b-3p; 133a-3p; 139-5p; 877-5p; 625-3p; 127-5p; 625-5p; 664a-5p; 299-5p; 3613-5p; 6511a-3p; 26b-5p; 432-5p; let-7b-5p; 206; 24-3p; 130b-5p; 3925-5p; 337-3p; 671-5p; 454-3p; 329-3p; 99b-5p; 145-5p; 125a-5p; 377-3p; 99b-3p; 376b-3p; 140-5p; 493-3p; 30d-5p; 193a-5p; 491-5p; 34c-5p; 9-5p; 301a-3p; 129-5p; 628-5p; 376c-3p; 1307-3p; 769-5p; 1271-5p; 204-5p; 1287-5p; 296-3p; 125b-1-3p; 500a-5p; 378a-3p; 543 |
| Endocytosis | 2.84E-02 | 125 | 133 | 27a-5p; 133a-5p; 107; 483-5p; 342-3p; 590-3p; 378a-5p; 1185-1-3p; 30a-3p; let-7e-5p; 320c; 320d; 92a-3p; let-7d-5p; 542-3p; 374a-5p; 31-5p; 132-5p; let-7a-5p; 197-3p; 320b; 22-3p; 486-3p; 25-3p; 149-5p; 181a-2-3p; 128-3p; 126-3p; 99a-5p; 205-5p; 1306-5p; 629-5p; 1-3p; 323a-3p; 379-5p; 374a-3p; 30e-5p; 130b-3p; 574-3p; 30c-5p; 485-3p; 98-5p; let-7f-5p; 15a-5p; 34a-5p; 330-5p; 7-5p; 411-5p; 665; 576-5p; 15b-5p; 103a-3p; let-7a-3p; 191-5p; 127-3p; 409-3p; 320a-3p; 382-5p; 130a-3p; 27b-3p; 423-5p; 374b-5p; 181b-5p; 6724-5p; 584-5p; 23a-3p; 22-5p; 92b-3p; 136-3p; 93-3p; 17-5p; 484; 192-5p; 421; 497-5p; 26a-5p; 328-3p; 708-5p; 503-5p; 675-3p; 195-5p; 941; 23b-3p; 7704; 133a-3p; 139-5p; 877-5p; 625-3p; 127-5p; 625-5p; 15b-3p; 299-5p; 6511a-3p; 26b-5p; 432-5p; let-7b-5p; 206; 24-3p; 130b-5p; 598-3p; 3925-5p; 337-3p; 190a-5p; 454-3p; 329-3p; 99b-5p; 145-5p; 125a-5p; 377-3p; 99b-3p; 376b-3p; 140-5p; 493-3p; 30d-5p; 296-5p; 491-5p; 34c-5p; 9-5p; 301a-3p; 129-5p; 628-5p; 376c-3p; 1307-3p; 769-5p; 1271-5p; 204-5p; 1287-5p; 296-3p; 125b-1-3p; 500a-5p; 378a-3p; 1185-5p; 543 |
| Hippo signaling pathway | 5.46E-05 | 114 | 132 | 27a-5p; 107; 342-3p; 590-3p; 378a-5p; 1185-1-3p; 30a-3p; let-7e-5p; 362-5p; 320c; 320d; 92a-3p; let-7d-5p; 542-3p; 100-3p; 374a-5p; 31-5p; 132-5p; let-7a-5p; 197-3p; 320b; 22-3p; 486-3p; 25-3p; 149-5p; 181a-2-3p; 128-3p; 126-3p; 99a-5p; 323b-3p; 205-5p; 629-5p; 1-3p; 323a-3p; 379-5p; 374a-3p; 30e-5p; 130b-3p; 574-3p; 30c-5p; 485-3p; 98-5p; let-7f-5p; 15a-5p; 34a-5p; 330-5p; 7-5p; 411-5p; 665; 576-5p; 15b-5p; 103a-3p; let-7a-3p; 191-5p; 151a-3p; 7706; 184; 409-3p; 320a-3p; 382-5p; let-7i-3p; 130a-3p; 27b-3p; 423-5p; 374b-5p; 181b-5p; 584-5p; 23a-3p; 22-5p; 92b-3p; 136-3p; 93-3p; 17-5p; 484; 192-5p; 421; 497-5p; 154-3p; 26a-5p; 328-3p; 708-5p; 503-5p; 675-3p; 195-5p; 941; 501-5p; 23b-3p; 133a-3p; 139-5p; 877-5p; 127-5p; 625-5p; 15b-3p; 6511a-3p; 26b-5p; let-7b-5p; 206; 24-3p; 130b-5p; 598-3p; 3925-5p; 337-3p; 671-5p; 454-3p; 329-3p; 889-3p; 99b-5p; 145-5p; 125a-5p; 377-3p; 99b-3p; 376b-3p; 140-5p; 493-3p; 30d-5p; 296-5p; 193a-5p; 491-5p; 34c-5p; 9-5p; 301a-3p; 129-5p; 376c-3p; 1307-3p; 769-5p; 1271-5p; 204-5p; 296-3p; 125b-1-3p; 500a-5p; 378a-3p; 1185-5p |
| Transcriptional misregulation in cancer | 2.51E-03 | 119 | 132 | 133a-5p; 107; 483-5p; 342-3p; 590-3p; 378a-5p; 1185-1-3p; 30a-3p; let-7e-5p; 362-5p; 320c; 320d; 92a-3p; let-7d-5p; 542-3p; 100-3p; 374a-5p; 31-5p; 132-5p; let-7a-5p; 197-3p; 320b; 22-3p; 486-3p; 25-3p; 149-5p; 181a-2-3p; 128-3p; 126-3p; 99a-5p; 205-5p; 1306-5p; 629-5p; 1-3p; 323a-3p; 379-5p; 374a-3p; 30e-5p; 130b-3p; 574-3p; 30c-5p; 485-3p; 98-5p; let-7f-5p; 15a-5p; 34a-5p; 330-5p; 7-5p; 665; 576-5p; 15b-5p; 103a-3p; let-7a-3p; 191-5p; 127-3p; 184; 409-3p; 320a-3p; 382-5p; 130a-3p; 27b-3p; 423-5p; 374b-5p; 628-3p; 181b-5p; 23a-3p; 22-5p; 92b-3p; 136-3p; 93-3p; 17-5p; 484; 192-5p; 421; 497-5p; 26a-5p; 328-3p; 708-5p; 503-5p; 675-3p; 195-5p; 941; 501-5p; 23b-3p; 7704; 133a-3p; 139-5p; 877-5p; 127-5p; 625-5p; 664a-5p; 15b-3p; 299-5p; 3613-5p; 6511a-3p; 26b-5p; 432-5p; let-7b-5p; 206; 24-3p; 130b-5p; 3925-5p; 337-3p; 671-5p; 190a-5p; 454-3p; 329-3p; 889-3p; 99b-5p; 145-5p; 125a-5p; 377-3p; 140-5p; 30d-5p; 296-5p; 193a-5p; 491-5p; 34c-5p; 9-5p; 301a-3p; 129-5p; 376c-3p; 769-5p; 1271-5p; 204-5p; 1287-5p; 296-3p; 125b-1-3p; 500a-5p; 378a-3p; 1185-5p; 543 |
| Prostate cancer | 9.74E-06 | 110 | 131 | 27a-5p; 133a-5p; 107; 483-5p; 342-3p; 590-3p; 378a-5p; 1185-1-3p; 30a-3p; let-7e-5p; 362-5p; 92a-3p; let-7d-5p; 542-3p; 100-3p; 374a-5p; 31-5p; 132-5p; let-7a-5p; 197-3p; 320b; 22-3p; 486-3p; 25-3p; 149-5p; 128-3p; 126-3p; 99a-5p; 323b-3p; 205-5p; 629-5p; 1-3p; 323a-3p; 374a-3p; 30e-5p; 130b-3p; 574-3p; 30c-5p; 485-3p; 98-5p; let-7f-5p; 15a-5p; 34a-5p; 330-5p; 7-5p; 411-5p; 665; 576-5p; 15b-5p; 103a-3p; let-7a-3p; 191-5p; 151a-3p; 7706; 184; 409-3p; 320a-3p; 382-5p; 130a-3p; 27b-3p; 423-5p; 374b-5p; 628-3p; 181b-5p; 584-5p; 23a-3p; 22-5p; 92b-3p; 93-3p; 17-5p; 484; 192-5p; 421; 497-5p; 154-3p; 26a-5p; 708-5p; 503-5p; 675-3p; 195-5p; 941; 501-5p; 23b-3p; 7704; 133a-3p; 139-5p; 877-5p; 625-3p; 127-5p; 625-5p; 15b-3p; 299-5p; 26b-5p; 432-5p; let-7b-5p; 206; 24-3p; 130b-5p; 3925-5p; 671-5p; 190a-5p; 454-3p; 329-3p; 889-3p; 99b-5p; 145-5p; 125a-5p; 377-3p; 99b-3p; 376b-3p; 140-5p; 30d-5p; 296-5p; 193a-5p; 491-5p; 34c-5p; 9-5p; 301a-3p; 129-5p; 628-5p; 376c-3p; 1307-3p; 769-5p; 1271-5p; 204-5p; 1287-5p; 296-3p; 125b-1-3p; 378a-3p; 1185-5p; 543 |
| mTOR signaling pathway | 4.88E-05 | 113 | 131 | 27a-5p; 133a-5p; 107; 483-5p; 342-3p; 590-3p; 378a-5p; 1185-1-3p; 30a-3p; let-7e-5p; 362-5p; 320c; 320d; 92a-3p; let-7d-5p; 542-3p; 100-3p; 374a-5p; 31-5p; 132-5p; let-7a-5p; 197-3p; 320b; 22-3p; 486-3p; 25-3p; 149-5p; 181a-2-3p; 128-3p; 126-3p; 99a-5p; 323b-3p; 205-5p; 1-3p; 374a-3p; 30e-5p; 130b-3p; 30c-5p; 98-5p; let-7f-5p; 15a-5p; 34a-5p; 330-5p; 7-5p; 411-5p; 665; 576-5p; 15b-5p; 103a-3p; let-7a-3p; 191-5p; 151a-3p; 184; 409-3p; 320a-3p; 382-5p; 130a-3p; 27b-3p; 423-5p; 374b-5p; 628-3p; 181b-5p; 6724-5p; 584-5p; 23a-3p; 92b-3p; 136-3p; 93-3p; 17-5p; 484; 192-5p; 421; 497-5p; 154-3p; 26a-5p; 708-5p; 503-5p; 675-3p; 195-5p; 501-5p; 23b-3p; 133a-3p; 139-5p; 877-5p; 625-3p; 625-5p; 15b-3p; 299-5p; 3613-5p; 26b-5p; 432-5p; let-7b-5p; 206; 24-3p; 130b-5p; 598-3p; 3925-5p; 337-3p; 671-5p; 190a-5p; 454-3p; 329-3p; 889-3p; 99b-5p; 145-5p; 125a-5p; 377-3p; 99b-3p; 376b-3p; 140-5p; 493-3p; 30d-5p; 296-5p; 193a-5p; 491-5p; 34c-5p; 9-5p; 301a-3p; 129-5p; 628-5p; 376c-3p; 1307-3p; 769-5p; 1271-5p; 204-5p; 1287-5p; 296-3p; 125b-1-3p; 500a-5p; 378a-3p; 543 |
| Ras signaling pathway | 6.24E-03 | 120 | 131 | 27a-5p; 133a-5p; 107; 483-5p; 342-3p; 590-3p; 378a-5p; 1185-1-3p; 30a-3p; let-7e-5p; 362-5p; 320c; 320d; 92a-3p; let-7d-5p; 542-3p; 100-3p; 374a-5p; 31-5p; let-7a-5p; 197-3p; 320b; 22-3p; 486-3p; 25-3p; 149-5p; 128-3p; 126-3p; 99a-5p; 205-5p; 629-5p; 1-3p; 374a-3p; 30e-5p; 130b-3p; 574-3p; 30c-5p; 485-3p; 98-5p; let-7f-5p; 15a-5p; 34a-5p; 330-5p; 7-5p; 411-5p; 665; 576-5p; 15b-5p; 103a-3p; let-7a-3p; 151a-3p; 184; 409-3p; 320a-3p; 382-5p; 130a-3p; 27b-3p; 423-5p; 374b-5p; 181b-5p; 584-5p; 23a-3p; 92b-3p; 136-3p; 93-3p; 17-5p; 484; 192-5p; 421; 497-5p; 154-3p; 26a-5p; 328-3p; 708-5p; 503-5p; 675-3p; 195-5p; 501-5p; 23b-3p; 7704; 133a-3p; 139-5p; 877-5p; 625-3p; 127-5p; 625-5p; 664a-5p; 15b-3p; 299-5p; 26b-5p; 432-5p; let-7b-5p; 206; 24-3p; 130b-5p; 598-3p; 3925-5p; 337-3p; 671-5p; 190a-5p; 454-3p; 329-3p; 889-3p; 99b-5p; 145-5p; 125a-5p; 377-3p; 376b-3p; 140-5p; 493-3p; 30d-5p; 296-5p; 193a-5p; 491-5p; 34c-5p; 9-5p; 301a-3p; 129-5p; 628-5p; 376c-3p; 1307-3p; 769-5p; 1271-5p; 204-5p; 1287-5p; 296-3p; 125b-1-3p; 500a-5p; 378a-3p; 1185-5p; 543 |
| Epstein-Barr virus infection | 8.90E-03 | 120 | 131 | 27a-5p; 133a-5p; 107; 483-5p; 342-3p; 590-3p; 378a-5p; 1185-1-3p; 30a-3p; let-7e-5p; 362-5p; 320c; 320d; 92a-3p; let-7d-5p; 542-3p; 100-3p; 374a-5p; 31-5p; let-7a-5p; 197-3p; 320b; 22-3p; 486-3p; 25-3p; 149-5p; 181a-2-3p; 128-3p; 126-3p; 99a-5p; 323b-3p; 205-5p; 1306-5p; 1-3p; 323a-3p; 379-5p; 374a-3p; 30e-5p; 130b-3p; 574-3p; 30c-5p; 98-5p; let-7f-5p; 15a-5p; 34a-5p; 330-5p; 7-5p; 411-5p; 665; 576-5p; 15b-5p; 103a-3p; let-7a-3p; 191-5p; 151a-3p; 7706; 184; 409-3p; 320a-3p; 382-5p; 130a-3p; 27b-3p; 423-5p; 374b-5p; 181b-5p; 6724-5p; 23a-3p; 22-5p; 92b-3p; 93-3p; 17-5p; 484; 192-5p; 421; 497-5p; 26a-5p; 328-3p; 708-5p; 503-5p; 195-5p; 501-5p; 23b-3p; 7704; 133a-3p; 139-5p; 877-5p; 625-3p; 127-5p; 625-5p; 15b-3p; 299-5p; 3613-5p; 6511a-3p; 26b-5p; 432-5p; let-7b-5p; 206; 24-3p; 130b-5p; 3925-5p; 337-3p; 671-5p; 454-3p; 329-3p; 145-5p; 125a-5p; 377-3p; 99b-3p; 376b-3p; 140-5p; 493-3p; 30d-5p; 296-5p; 193a-5p; 491-5p; 34c-5p; 9-5p; 301a-3p; 129-5p; 628-5p; 376c-3p; 1307-3p; 769-5p; 1271-5p; 204-5p; 1287-5p; 296-3p; 125b-1-3p; 500a-5p; 378a-3p; 1185-5p |
| Human T-cell leukemia virus 1 infection | 3.79E-02 | 123 | 131 | 27a-5p; 107; 483-5p; 342-3p; 590-3p; 378a-5p; 1185-1-3p; 30a-3p; let-7e-5p; 362-5p; 320c; 92a-3p; let-7d-5p; 542-3p; 100-3p; 374a-5p; 31-5p; let-7a-5p; 197-3p; 320b; 22-3p; 486-3p; 25-3p; 149-5p; 181a-2-3p; 128-3p; 126-3p; 99a-5p; 323b-3p; 205-5p; 1306-5p; 1-3p; 323a-3p; 379-5p; 374a-3p; 30e-5p; 130b-3p; 574-3p; 30c-5p; 485-3p; 98-5p; let-7f-5p; 15a-5p; 34a-5p; 330-5p; 7-5p; 665; 576-5p; 15b-5p; 103a-3p; let-7a-3p; 191-5p; 151a-3p; 7706; 184; 409-3p; 320a-3p; 382-5p; 130a-3p; 27b-3p; 423-5p; 374b-5p; 628-3p; 181b-5p; 6724-5p; 584-5p; 23a-3p; 22-5p; 92b-3p; 136-3p; 93-3p; 17-5p; 484; 192-5p; 421; 497-5p; 154-3p; 26a-5p; 708-5p; 503-5p; 195-5p; 941; 23b-3p; 7704; 133a-3p; 139-5p; 877-5p; 625-3p; 127-5p; 625-5p; 15b-3p; 299-5p; 3613-5p; 6511a-3p; 26b-5p; 432-5p; let-7b-5p; 206; 24-3p; 130b-5p; 3925-5p; 337-3p; 671-5p; 454-3p; 329-3p; 889-3p; 99b-5p; 145-5p; 125a-5p; 377-3p; 376b-3p; 140-5p; 493-3p; 30d-5p; 193a-5p; 491-5p; 34c-5p; 9-5p; 301a-3p; 129-5p; 628-5p; 376c-3p; 1307-3p; 769-5p; 1271-5p; 204-5p; 1287-5p; 296-3p; 125b-1-3p; 378a-3p; 543 |
| Hepatitis C | 1.56E-04 | 113 | 130 | 27a-5p; 133a-5p; 107; 483-5p; 342-3p; 590-3p; 378a-5p; 1185-1-3p; 30a-3p; let-7e-5p; 362-5p; 320c; 92a-3p; let-7d-5p; 542-3p; 100-3p; 374a-5p; 31-5p; 132-5p; let-7a-5p; 197-3p; 320b; 22-3p; 486-3p; 25-3p; 149-5p; 128-3p; 126-3p; 99a-5p; 323b-3p; 205-5p; 1306-5p; 629-5p; 1-3p; 323a-3p; 374a-3p; 30e-5p; 130b-3p; 574-3p; 30c-5p; 485-3p; 98-5p; let-7f-5p; 15a-5p; 34a-5p; 330-5p; 7-5p; 411-5p; 665; 576-5p; 15b-5p; 103a-3p; let-7a-3p; 191-5p; 151a-3p; 7706; 184; 409-3p; 320a-3p; 382-5p; 130a-3p; 27b-3p; 423-5p; 374b-5p; 181b-5p; 584-5p; 23a-3p; 22-5p; 92b-3p; 93-3p; 17-5p; 484; 192-5p; 421; 497-5p; 154-3p; 26a-5p; 328-3p; 708-5p; 503-5p; 195-5p; 941; 501-5p; 23b-3p; 7704; 133a-3p; 139-5p; 877-5p; 625-3p; 625-5p; 299-5p; 3613-5p; 26b-5p; let-7b-5p; 206; 24-3p; 130b-5p; 598-3p; 3925-5p; 337-3p; 671-5p; 454-3p; 329-3p; 889-3p; 145-5p; 125a-5p; 377-3p; 99b-3p; 376b-3p; 140-5p; 493-3p; 30d-5p; 296-5p; 193a-5p; 491-5p; 34c-5p; 9-5p; 301a-3p; 129-5p; 376c-3p; 1307-3p; 769-5p; 1271-5p; 204-5p; 296-3p; 125b-1-3p; 500a-5p; 378a-3p; 1185-5p; 543 |
| Human immunodeficiency virus 1 infection | 1.32E-03 | 116 | 130 | 27a-5p; 107; 483-5p; 342-3p; 590-3p; 378a-5p; 1185-1-3p; 30a-3p; let-7e-5p; 362-5p; 320c; 320d; 92a-3p; let-7d-5p; 542-3p; 100-3p; 374a-5p; 31-5p; let-7a-5p; 197-3p; 320b; 22-3p; 486-3p; 25-3p; 149-5p; 181a-2-3p; 128-3p; 126-3p; 99a-5p; 323b-3p; 205-5p; 1306-5p; 1-3p; 323a-3p; 379-5p; 374a-3p; 30e-5p; 130b-3p; 574-3p; 30c-5p; 485-3p; 98-5p; let-7f-5p; 15a-5p; 34a-5p; 330-5p; 7-5p; 411-5p; 665; 576-5p; 15b-5p; 103a-3p; let-7a-3p; 151a-3p; 184; 409-3p; 320a-3p; 382-5p; 130a-3p; 27b-3p; 423-5p; 374b-5p; 181b-5p; 6724-5p; 584-5p; 23a-3p; 92b-3p; 93-3p; 17-5p; 484; 192-5p; 421; 497-5p; 154-3p; 26a-5p; 328-3p; 708-5p; 503-5p; 195-5p; 941; 501-5p; 23b-3p; 133a-3p; 139-5p; 877-5p; 625-3p; 127-5p; 625-5p; 15b-3p; 6511a-3p; 26b-5p; 432-5p; let-7b-5p; 206; 24-3p; 130b-5p; 598-3p; 3925-5p; 337-3p; 671-5p; 190a-5p; 454-3p; 329-3p; 889-3p; 99b-5p; 145-5p; 125a-5p; 377-3p; 376b-3p; 493-3p; 30d-5p; 296-5p; 193a-5p; 491-5p; 34c-5p; 9-5p; 301a-3p; 129-5p; 628-5p; 376c-3p; 1307-3p; 769-5p; 1271-5p; 204-5p; 1287-5p; 296-3p; 125b-1-3p; 500a-5p; 378a-3p; 543 |
| Breast cancer | 4.58E-03 | 118 | 130 | 27a-5p; 133a-5p; 107; 483-5p; 342-3p; 590-3p; 378a-5p; 1185-1-3p; 30a-3p; let-7e-5p; 362-5p; 320c; 92a-3p; let-7d-5p; 542-3p; 100-3p; 374a-5p; 31-5p; let-7a-5p; 197-3p; 320b; 22-3p; 486-3p; 25-3p; 149-5p; 128-3p; 126-3p; 99a-5p; 323b-3p; 205-5p; 1-3p; 323a-3p; 379-5p; 374a-3p; 30e-5p; 130b-3p; 574-3p; 30c-5p; 485-3p; 98-5p; let-7f-5p; 15a-5p; 34a-5p; 330-5p; 7-5p; 411-5p; 665; 576-5p; 15b-5p; 103a-3p; let-7a-3p; 191-5p; 7706; 184; 409-3p; 320a-3p; 382-5p; 130a-3p; 27b-3p; 423-5p; 374b-5p; 628-3p; 181b-5p; 584-5p; 23a-3p; 22-5p; 92b-3p; 136-3p; 93-3p; 17-5p; 484; 192-5p; 421; 497-5p; 154-3p; 26a-5p; 328-3p; 708-5p; 503-5p; 675-3p; 195-5p; 501-5p; 23b-3p; 7704; 133a-3p; 139-5p; 877-5p; 625-3p; 625-5p; 15b-3p; 299-5p; 3613-5p; 26b-5p; let-7b-5p; 206; 24-3p; 130b-5p; 3925-5p; 671-5p; 190a-5p; 454-3p; 329-3p; 889-3p; 99b-5p; 145-5p; 125a-5p; 377-3p; 99b-3p; 376b-3p; 140-5p; 493-3p; 30d-5p; 296-5p; 193a-5p; 491-5p; 34c-5p; 9-5p; 301a-3p; 129-5p; 376c-3p; 1307-3p; 769-5p; 1271-5p; 204-5p; 1287-5p; 296-3p; 125b-1-3p; 500a-5p; 378a-3p; 543 |
| Kaposi sarcoma-associated herpesvirus infection | 7.18E-03 | 119 | 130 | 27a-5p; 107; 483-5p; 342-3p; 590-3p; 378a-5p; 1185-1-3p; 30a-3p; let-7e-5p; 362-5p; 320c; 92a-3p; let-7d-5p; 542-3p; 100-3p; 374a-5p; 31-5p; let-7a-5p; 197-3p; 320b; 22-3p; 486-3p; 25-3p; 149-5p; 181a-2-3p; 128-3p; 126-3p; 99a-5p; 323b-3p; 205-5p; 1306-5p; 1-3p; 323a-3p; 374a-3p; 30e-5p; 130b-3p; 574-3p; 30c-5p; 98-5p; let-7f-5p; 15a-5p; 34a-5p; 330-5p; 7-5p; 411-5p; 665; 576-5p; 15b-5p; 103a-3p; let-7a-3p; 191-5p; 127-3p; 151a-3p; 7706; 184; 409-3p; 320a-3p; 382-5p; 130a-3p; 27b-3p; 423-5p; 374b-5p; 628-3p; 181b-5p; 584-5p; 23a-3p; 22-5p; 92b-3p; 93-3p; 17-5p; 484; 192-5p; 421; 497-5p; 154-3p; 26a-5p; 708-5p; 503-5p; 195-5p; 501-5p; 23b-3p; 7704; 133a-3p; 139-5p; 877-5p; 625-3p; 127-5p; 625-5p; 299-5p; 3613-5p; 26b-5p; let-7b-5p; 206; 24-3p; 130b-5p; 598-3p; 3925-5p; 337-3p; 671-5p; 190a-5p; 454-3p; 329-3p; 889-3p; 99b-5p; 145-5p; 125a-5p; 377-3p; 99b-3p; 376b-3p; 140-5p; 493-3p; 30d-5p; 296-5p; 193a-5p; 491-5p; 34c-5p; 9-5p; 301a-3p; 129-5p; 628-5p; 1307-3p; 769-5p; 1271-5p; 204-5p; 1287-5p; 296-3p; 125b-1-3p; 500a-5p; 378a-3p; 543 |
| Regulation of actin cytoskeleton | 8.55E-03 | 119 | 130 | 27a-5p; 133a-5p; 107; 483-5p; 342-3p; 590-3p; 378a-5p; 30a-3p; let-7e-5p; 320c; 320d; 92a-3p; let-7d-5p; 542-3p; 100-3p; 374a-5p; 31-5p; let-7a-5p; 197-3p; 320b; 22-3p; 486-3p; 25-3p; 149-5p; 181a-2-3p; 128-3p; 126-3p; 99a-5p; 323b-3p; 205-5p; 1306-5p; 1-3p; 323a-3p; 379-5p; 374a-3p; 30e-5p; 130b-3p; 574-3p; 30c-5p; 98-5p; let-7f-5p; 15a-5p; 34a-5p; 330-5p; 7-5p; 411-5p; 665; 576-5p; 15b-5p; 103a-3p; let-7a-3p; 191-5p; 7706; 184; 409-3p; 320a-3p; let-7i-3p; 130a-3p; 27b-3p; 423-5p; 374b-5p; 181b-5p; 584-5p; 23a-3p; 22-5p; 92b-3p; 136-3p; 93-3p; 17-5p; 484; 192-5p; 497-5p; 154-3p; 26a-5p; 328-3p; 503-5p; 675-3p; 195-5p; 941; 501-5p; 23b-3p; 7704; 133a-3p; 139-5p; 877-5p; 625-3p; 127-5p; 625-5p; 15b-3p; 6511a-3p; 26b-5p; 432-5p; let-7b-5p; 206; 24-3p; 130b-5p; 598-3p; 3925-5p; 671-5p; 454-3p; 329-3p; 889-3p; 99b-5p; 145-5p; 125a-5p; 377-3p; 99b-3p; 376b-3p; 140-5p; 493-3p; 30d-5p; 296-5p; 193a-5p; 491-5p; 34c-5p; 9-5p; 301a-3p; 129-5p; 628-5p; 1307-3p; 769-5p; 1271-5p; 204-5p; 1287-5p; 296-3p; 125b-1-3p; 500a-5p; 378a-3p; 1185-5p; 543 |
| cAMP signaling pathway | 5.09E-04 | 113 | 129 | 27a-5p; 133a-5p; 107; 483-5p; 342-3p; 590-3p; 378a-5p; 1185-1-3p; 30a-3p; let-7e-5p; 320c; 320d; 92a-3p; let-7d-5p; 542-3p; 100-3p; 374a-5p; 31-5p; let-7a-5p; 197-3p; 320b; 22-3p; 486-3p; 25-3p; 149-5p; 181a-2-3p; 128-3p; 126-3p; 99a-5p; 323b-3p; 205-5p; 1306-5p; 1-3p; 323a-3p; 379-5p; 374a-3p; 30e-5p; 130b-3p; 574-3p; 30c-5p; 98-5p; let-7f-5p; 15a-5p; 34a-5p; 330-5p; 7-5p; 411-5p; 665; 576-5p; 15b-5p; 103a-3p; let-7a-3p; 191-5p; 7706; 184; 409-3p; 320a-3p; 382-5p; 130a-3p; 27b-3p; 423-5p; 374b-5p; 628-3p; 181b-5p; 584-5p; 23a-3p; 22-5p; 92b-3p; 93-3p; 17-5p; 484; 192-5p; 421; 497-5p; 154-3p; 26a-5p; 328-3p; 708-5p; 503-5p; 195-5p; 941; 501-5p; 23b-3p; 133a-3p; 139-5p; 877-5p; 625-3p; 127-5p; 625-5p; 664a-5p; 6511a-3p; 26b-5p; 432-5p; let-7b-5p; 206; 24-3p; 130b-5p; 598-3p; 3925-5p; 337-3p; 671-5p; 190a-5p; 454-3p; 329-3p; 889-3p; 99b-5p; 145-5p; 125a-5p; 377-3p; 376b-3p; 140-5p; 493-3p; 30d-5p; 193a-5p; 491-5p; 34c-5p; 9-5p; 301a-3p; 129-5p; 376c-3p; 1307-3p; 769-5p; 1271-5p; 204-5p; 296-3p; 500a-5p; 378a-3p; 1185-5p; 543 |
| Pathogenic Escherichia coli infection | 3.35E-03 | 116 | 129 | 27a-5p; 133a-5p; 107; 483-5p; 342-3p; 590-3p; 378a-5p; 30a-3p; let-7e-5p; 362-5p; 320c; 320d; 92a-3p; let-7d-5p; 542-3p; 100-3p; 374a-5p; 31-5p; let-7a-5p; 197-3p; 320b; 22-3p; 486-3p; 25-3p; 149-5p; 181a-2-3p; 128-3p; 126-3p; 323b-3p; 205-5p; 1306-5p; 1-3p; 323a-3p; 379-5p; 374a-3p; 30e-5p; 130b-3p; 574-3p; 30c-5p; 485-3p; 98-5p; let-7f-5p; 15a-5p; 34a-5p; 330-5p; 7-5p; 411-5p; 665; 576-5p; 15b-5p; 103a-3p; let-7a-3p; 191-5p; 151a-3p; 7706; 184; 320a-3p; 382-5p; let-7i-3p; 130a-3p; 27b-3p; 423-5p; 374b-5p; 181b-5p; 584-5p; 23a-3p; 22-5p; 92b-3p; 93-3p; 17-5p; 484; 192-5p; 421; 497-5p; 154-3p; 26a-5p; 328-3p; 708-5p; 503-5p; 195-5p; 501-5p; 23b-3p; 7704; 133a-3p; 139-5p; 877-5p; 625-3p; 127-5p; 625-5p; 15b-3p; 3613-5p; 6511a-3p; 26b-5p; 432-5p; let-7b-5p; 24-3p; 130b-5p; 598-3p; 3925-5p; 337-3p; 671-5p; 454-3p; 329-3p; 99b-5p; 145-5p; 125a-5p; 377-3p; 99b-3p; 376b-3p; 140-5p; 493-3p; 30d-5p; 296-5p; 491-5p; 34c-5p; 9-5p; 301a-3p; 129-5p; 628-5p; 376c-3p; 1307-3p; 769-5p; 1271-5p; 204-5p; 1287-5p; 296-3p; 500a-5p; 378a-3p; 543 |
| Influenza A | 1.02E-08 | 99 | 128 | 27a-5p; 107; 483-5p; 342-3p; 590-3p; 378a-5p; 1185-1-3p; 30a-3p; let-7e-5p; 362-5p; 320c; 92a-3p; let-7d-5p; 542-3p; 100-3p; 374a-5p; 132-5p; let-7a-5p; 197-3p; 320b; 22-3p; 486-3p; 25-3p; 149-5p; 181a-2-3p; 128-3p; 126-3p; 99a-5p; 205-5p; 1306-5p; 1-3p; 323a-3p; 379-5p; 374a-3p; 30e-5p; 130b-3p; 574-3p; 30c-5p; 485-3p; 98-5p; let-7f-5p; 15a-5p; 34a-5p; 330-5p; 7-5p; 411-5p; 665; 576-5p; 15b-5p; 103a-3p; 191-5p; 151a-3p; 184; 409-3p; 320a-3p; 382-5p; let-7i-3p; 130a-3p; 27b-3p; 423-5p; 374b-5p; 628-3p; 181b-5p; 584-5p; 23a-3p; 22-5p; 92b-3p; 93-3p; 17-5p; 484; 192-5p; 421; 497-5p; 154-3p; 26a-5p; 708-5p; 503-5p; 195-5p; 501-5p; 23b-3p; 7704; 133a-3p; 139-5p; 877-5p; 625-3p; 127-5p; 625-5p; 664a-5p; 15b-3p; 3613-5p; 6511a-3p; 26b-5p; 432-5p; let-7b-5p; 206; 24-3p; 130b-5p; 3925-5p; 337-3p; 671-5p; 454-3p; 329-3p; 889-3p; 99b-5p; 145-5p; 125a-5p; 99b-3p; 376b-3p; 140-5p; 493-3p; 30d-5p; 296-5p; 193a-5p; 491-5p; 34c-5p; 9-5p; 301a-3p; 129-5p; 628-5p; 376c-3p; 1307-3p; 769-5p; 1271-5p; 204-5p; 296-3p; 500a-5p; 378a-3p; 543 |
| Apelin signaling pathway | 1.12E-07 | 102 | 128 | 27a-5p; 107; 483-5p; 342-3p; 590-3p; 378a-5p; 1185-1-3p; 30a-3p; let-7e-5p; 362-5p; 320c; 320d; 92a-3p; let-7d-5p; 542-3p; 100-3p; 374a-5p; 31-5p; let-7a-5p; 197-3p; 320b; 22-3p; 486-3p; 25-3p; 149-5p; 181a-2-3p; 128-3p; 126-3p; 99a-5p; 323b-3p; 205-5p; 1-3p; 323a-3p; 379-5p; 374a-3p; 30e-5p; 130b-3p; 574-3p; 30c-5p; 485-3p; 98-5p; let-7f-5p; 15a-5p; 34a-5p; 330-5p; 7-5p; 665; 15b-5p; 103a-3p; let-7a-3p; 191-5p; 127-3p; 7706; 184; 409-3p; 320a-3p; 382-5p; 130a-3p; 27b-3p; 423-5p; 374b-5p; 181b-5p; 584-5p; 23a-3p; 92b-3p; 136-3p; 93-3p; 17-5p; 484; 192-5p; 421; 497-5p; 154-3p; 26a-5p; 708-5p; 503-5p; 675-3p; 195-5p; 501-5p; 23b-3p; 7704; 133a-3p; 139-5p; 877-5p; 625-3p; 127-5p; 625-5p; 299-5p; 3613-5p; 26b-5p; 432-5p; let-7b-5p; 206; 24-3p; 598-3p; 3925-5p; 337-3p; 671-5p; 190a-5p; 454-3p; 329-3p; 889-3p; 99b-5p; 145-5p; 125a-5p; 377-3p; 376b-3p; 140-5p; 493-3p; 30d-5p; 296-5p; 193a-5p; 491-5p; 34c-5p; 9-5p; 301a-3p; 129-5p; 376c-3p; 1307-3p; 769-5p; 1271-5p; 204-5p; 1287-5p; 296-3p; 500a-5p; 378a-3p; 1185-5p; 543 |
| Hepatitis B | 1.36E-03 | 114 | 128 | 27a-5p; 133a-5p; 107; 483-5p; 342-3p; 590-3p; 378a-5p; 1185-1-3p; 30a-3p; let-7e-5p; 362-5p; 320c; 92a-3p; let-7d-5p; 542-3p; 100-3p; 374a-5p; 31-5p; let-7a-5p; 197-3p; 320b; 22-3p; 486-3p; 25-3p; 149-5p; 181a-2-3p; 128-3p; 126-3p; 99a-5p; 323b-3p; 205-5p; 1306-5p; 1-3p; 323a-3p; 379-5p; 374a-3p; 30e-5p; 130b-3p; 574-3p; 30c-5p; 485-3p; 98-5p; let-7f-5p; 15a-5p; 34a-5p; 330-5p; 7-5p; 411-5p; 665; 576-5p; 15b-5p; 103a-3p; let-7a-3p; 191-5p; 151a-3p; 184; 409-3p; 320a-3p; 382-5p; 130a-3p; 27b-3p; 423-5p; 374b-5p; 628-3p; 181b-5p; 584-5p; 23a-3p; 22-5p; 92b-3p; 93-3p; 17-5p; 484; 192-5p; 421; 497-5p; 154-3p; 26a-5p; 328-3p; 708-5p; 503-5p; 195-5p; 941; 501-5p; 23b-3p; 7704; 133a-3p; 139-5p; 877-5p; 625-3p; 664a-5p; 299-5p; 6511a-3p; 26b-5p; let-7b-5p; 206; 24-3p; 130b-5p; 3925-5p; 337-3p; 671-5p; 454-3p; 329-3p; 889-3p; 145-5p; 125a-5p; 377-3p; 376b-3p; 140-5p; 493-3p; 30d-5p; 296-5p; 193a-5p; 491-5p; 34c-5p; 9-5p; 301a-3p; 129-5p; 628-5p; 376c-3p; 1307-3p; 769-5p; 204-5p; 296-3p; 125b-1-3p; 500a-5p; 378a-3p; 1185-5p; 543 |
| Oxytocin signaling pathway | 1.26E-04 | 108 | 126 | 27a-5p; 133a-5p; 107; 483-5p; 342-3p; 590-3p; 378a-5p; 30a-3p; let-7e-5p; 320c; 320d; 92a-3p; let-7d-5p; 542-3p; 100-3p; 374a-5p; 31-5p; let-7a-5p; 197-3p; 320b; 22-3p; 486-3p; 25-3p; 149-5p; 181a-2-3p; 128-3p; 126-3p; 99a-5p; 323b-3p; 205-5p; 1306-5p; 1-3p; 323a-3p; 374a-3p; 30e-5p; 130b-3p; 574-3p; 30c-5p; 98-5p; let-7f-5p; 15a-5p; 34a-5p; 330-5p; 7-5p; 665; 576-5p; 15b-5p; 103a-3p; let-7a-3p; 191-5p; 127-3p; 151a-3p; 7706; 184; 409-3p; 320a-3p; 382-5p; let-7i-3p; 130a-3p; 27b-3p; 423-5p; 374b-5p; 181b-5p; 584-5p; 23a-3p; 22-5p; 92b-3p; 136-3p; 93-3p; 17-5p; 484; 192-5p; 421; 497-5p; 154-3p; 26a-5p; 708-5p; 503-5p; 675-3p; 195-5p; 501-5p; 23b-3p; 7704; 133a-3p; 139-5p; 877-5p; 625-3p; 127-5p; 625-5p; 664a-5p; 299-5p; 3613-5p; 26b-5p; 432-5p; let-7b-5p; 206; 24-3p; 130b-5p; 598-3p; 3925-5p; 337-3p; 671-5p; 190a-5p; 454-3p; 329-3p; 889-3p; 99b-5p; 145-5p; 125a-5p; 99b-3p; 376b-3p; 493-3p; 30d-5p; 491-5p; 34c-5p; 9-5p; 301a-3p; 129-5p; 1307-3p; 769-5p; 1271-5p; 204-5p; 296-3p; 500a-5p; 378a-3p; 543 |
| Axon guidance | 3.72E-04 | 109 | 126 | 133a-5p; 107; 483-5p; 342-3p; 590-3p; 378a-5p; 1185-1-3p; 30a-3p; let-7e-5p; 362-5p; 320c; 320d; 92a-3p; let-7d-5p; 542-3p; 100-3p; 374a-5p; 31-5p; let-7a-5p; 197-3p; 320b; 22-3p; 486-3p; 25-3p; 149-5p; 181a-2-3p; 128-3p; 126-3p; 323b-3p; 205-5p; 1306-5p; 629-5p; 1-3p; 323a-3p; 379-5p; 374a-3p; 30e-5p; 130b-3p; 574-3p; 30c-5p; 98-5p; let-7f-5p; 15a-5p; 34a-5p; 330-5p; 7-5p; 411-5p; 665; 576-5p; 15b-5p; 103a-3p; let-7a-3p; 191-5p; 127-3p; 151a-3p; 184; 409-3p; 320a-3p; 130a-3p; 27b-3p; 423-5p; 374b-5p; 181b-5p; 584-5p; 23a-3p; 22-5p; 92b-3p; 93-3p; 17-5p; 484; 192-5p; 421; 497-5p; 154-3p; 26a-5p; 708-5p; 503-5p; 195-5p; 501-5p; 23b-3p; 133a-3p; 139-5p; 877-5p; 625-3p; 625-5p; 664a-5p; 299-5p; 3613-5p; 6511a-3p; 26b-5p; 432-5p; let-7b-5p; 206; 24-3p; 130b-5p; 3925-5p; 337-3p; 671-5p; 454-3p; 329-3p; 889-3p; 145-5p; 125a-5p; 377-3p; 99b-3p; 376b-3p; 493-3p; 30d-5p; 193a-5p; 491-5p; 34c-5p; 9-5p; 301a-3p; 129-5p; 628-5p; 1307-3p; 769-5p; 1271-5p; 204-5p; 1287-5p; 296-3p; 125b-1-3p; 500a-5p; 378a-3p; 1185-5p; 543 |
| Gastric cancer | 4.20E-03 | 113 | 126 | 27a-5p; 133a-5p; 107; 483-5p; 342-3p; 590-3p; 378a-5p; 1185-1-3p; 30a-3p; let-7e-5p; 362-5p; 320c; 92a-3p; let-7d-5p; 542-3p; 100-3p; 374a-5p; 31-5p; let-7a-5p; 197-3p; 320b; 22-3p; 486-3p; 25-3p; 149-5p; 128-3p; 126-3p; 99a-5p; 323b-3p; 205-5p; 1-3p; 323a-3p; 379-5p; 374a-3p; 30e-5p; 130b-3p; 574-3p; 30c-5p; 485-3p; 98-5p; let-7f-5p; 15a-5p; 34a-5p; 330-5p; 7-5p; 411-5p; 665; 576-5p; 15b-5p; 103a-3p; let-7a-3p; 191-5p; 151a-3p; 7706; 184; 409-3p; 320a-3p; 130a-3p; 27b-3p; 423-5p; 374b-5p; 628-3p; 181b-5p; 584-5p; 23a-3p; 22-5p; 92b-3p; 136-3p; 93-3p; 17-5p; 484; 192-5p; 421; 497-5p; 154-3p; 26a-5p; 328-3p; 708-5p; 503-5p; 675-3p; 195-5p; 501-5p; 23b-3p; 7704; 133a-3p; 139-5p; 877-5p; 625-3p; 625-5p; 299-5p; 26b-5p; let-7b-5p; 206; 24-3p; 130b-5p; 3925-5p; 671-5p; 454-3p; 329-3p; 889-3p; 99b-5p; 145-5p; 125a-5p; 377-3p; 99b-3p; 376b-3p; 140-5p; 493-3p; 30d-5p; 193a-5p; 491-5p; 34c-5p; 9-5p; 301a-3p; 129-5p; 376c-3p; 1307-3p; 769-5p; 1271-5p; 204-5p; 296-3p; 125b-1-3p; 500a-5p; 378a-3p; 1185-5p; 543 |
| Chemokine signaling pathway | 2.72E-05 | 104 | 125 | 27a-5p; 133a-5p; 107; 483-5p; 342-3p; 590-3p; 378a-5p; 1185-1-3p; 30a-3p; let-7e-5p; 320c; 320d; 92a-3p; let-7d-5p; 542-3p; 100-3p; 374a-5p; 31-5p; let-7a-5p; 197-3p; 320b; 22-3p; 486-3p; 25-3p; 149-5p; 128-3p; 126-3p; 99a-5p; 205-5p; 1306-5p; 629-5p; 1-3p; 323a-3p; 379-5p; 374a-3p; 30e-5p; 130b-3p; 574-3p; 30c-5p; 98-5p; let-7f-5p; 15a-5p; 34a-5p; 330-5p; 7-5p; 411-5p; 665; 576-5p; 15b-5p; 103a-3p; let-7a-3p; 191-5p; 127-3p; 151a-3p; 7706; 184; 409-3p; 320a-3p; 130a-3p; 27b-3p; 423-5p; 374b-5p; 628-3p; 181b-5p; 584-5p; 23a-3p; 92b-3p; 93-3p; 17-5p; 484; 192-5p; 421; 497-5p; 154-3p; 26a-5p; 708-5p; 503-5p; 195-5p; 941; 501-5p; 23b-3p; 133a-3p; 139-5p; 877-5p; 625-3p; 127-5p; 625-5p; 6511a-3p; 26b-5p; let-7b-5p; 206; 24-3p; 130b-5p; 3925-5p; 337-3p; 671-5p; 454-3p; 329-3p; 889-3p; 145-5p; 125a-5p; 377-3p; 99b-3p; 376b-3p; 140-5p; 493-3p; 30d-5p; 296-5p; 193a-5p; 491-5p; 34c-5p; 9-5p; 301a-3p; 129-5p; 628-5p; 376c-3p; 1307-3p; 769-5p; 1271-5p; 204-5p; 1287-5p; 296-3p; 500a-5p; 378a-3p; 543 |
| Cell cycle | 8.59E-03 | 113 | 125 | 107; 342-3p; 590-3p; 378a-5p; 1185-1-3p; 30a-3p; let-7e-5p; 362-5p; 92a-3p; let-7d-5p; 542-3p; 100-3p; 374a-5p; 31-5p; 132-5p; let-7a-5p; 197-3p; 320b; 22-3p; 486-3p; 25-3p; 149-5p; 128-3p; 99a-5p; 323b-3p; 205-5p; 1306-5p; 629-5p; 1-3p; 323a-3p; 374a-3p; 30e-5p; 130b-3p; 574-3p; 30c-5p; 485-3p; 98-5p; let-7f-5p; 15a-5p; 34a-5p; 330-5p; 7-5p; 411-5p; 665; 576-5p; 15b-5p; 103a-3p; let-7a-3p; 191-5p; 151a-3p; 7706; 184; 409-3p; 320a-3p; 382-5p; 130a-3p; 27b-3p; 423-5p; 374b-5p; 628-3p; 181b-5p; 584-5p; 23a-3p; 22-5p; 92b-3p; 93-3p; 17-5p; 484; 192-5p; 421; 497-5p; 26a-5p; 328-3p; 708-5p; 503-5p; 195-5p; 941; 501-5p; 23b-3p; 7704; 133a-3p; 877-5p; 127-5p; 625-5p; 15b-3p; 299-5p; 3613-5p; 26b-5p; 432-5p; let-7b-5p; 206; 24-3p; 130b-5p; 598-3p; 3925-5p; 671-5p; 454-3p; 329-3p; 99b-5p; 145-5p; 125a-5p; 377-3p; 99b-3p; 376b-3p; 140-5p; 493-3p; 30d-5p; 296-5p; 193a-5p; 491-5p; 34c-5p; 9-5p; 301a-3p; 129-5p; 376c-3p; 1307-3p; 769-5p; 1271-5p; 204-5p; 296-3p; 125b-1-3p; 500a-5p; 378a-3p; 1185-5p; 543 |
| Spliceosome | 6.46E-07 | 99 | 124 | 27a-5p; 107; 483-5p; 342-3p; let-7d-3p; 590-3p; 378a-5p; 1185-1-3p; 30a-3p; let-7e-5p; 362-5p; 320c; 320d; 92a-3p; let-7d-5p; 542-3p; 100-3p; 374a-5p; 31-5p; let-7a-5p; 197-3p; 320b; 22-3p; 486-3p; 25-3p; 149-5p; 128-3p; 126-3p; 99a-5p; 205-5p; 1306-5p; 1-3p; 323a-3p; 379-5p; 374a-3p; 30e-5p; 130b-3p; 30c-5p; 485-3p; 98-5p; let-7f-5p; 15a-5p; 34a-5p; 330-5p; 7-5p; 411-5p; 665; 15b-5p; 103a-3p; let-7a-3p; 191-5p; 409-3p; 320a-3p; 382-5p; 130a-3p; 27b-3p; 423-5p; 374b-5p; 628-3p; 181b-5p; 6724-5p; 584-5p; 23a-3p; 22-5p; 92b-3p; 136-3p; 93-3p; 17-5p; 484; 192-5p; 421; 497-5p; 26a-5p; 328-3p; 708-5p; 503-5p; 675-3p; 195-5p; 941; 501-5p; 23b-3p; 133a-3p; 877-5p; 625-3p; 127-5p; 625-5p; 664a-5p; 299-5p; 3613-5p; 6511a-3p; 26b-5p; 432-5p; let-7b-5p; 206; 24-3p; 130b-5p; 337-3p; 671-5p; 190a-5p; 454-3p; 329-3p; 889-3p; 99b-5p; 145-5p; 125a-5p; 377-3p; 376b-3p; 140-5p; 493-3p; 30d-5p; 296-5p; 193a-5p; 34c-5p; 9-5p; 301a-3p; 129-5p; 376c-3p; 1307-3p; 769-5p; 1287-5p; 296-3p; 500a-5p; 378a-3p; 543 |
| AMPK signaling pathway | 8.28E-05 | 105 | 124 | 27a-5p; 107; 342-3p; 590-3p; 378a-5p; 1185-1-3p; let-7e-5p; 362-5p; 320c; 320d; 92a-3p; let-7d-5p; 542-3p; 100-3p; 374a-5p; 31-5p; 132-5p; let-7a-5p; 197-3p; 320b; 22-3p; 486-3p; 25-3p; 149-5p; 128-3p; 126-3p; 99a-5p; 323b-3p; 205-5p; 629-5p; 1-3p; 374a-3p; 30e-5p; 130b-3p; 30c-5p; 485-3p; 98-5p; let-7f-5p; 15a-5p; 34a-5p; 330-5p; 7-5p; 665; 576-5p; 15b-5p; 103a-3p; let-7a-3p; 191-5p; 7706; 184; 409-3p; 320a-3p; 382-5p; 130a-3p; 27b-3p; 423-5p; 374b-5p; 181b-5p; 584-5p; 23a-3p; 92b-3p; 136-3p; 93-3p; 17-5p; 484; 192-5p; 421; 497-5p; 154-3p; 26a-5p; 328-3p; 708-5p; 503-5p; 675-3p; 195-5p; 941; 501-5p; 23b-3p; 133a-3p; 139-5p; 877-5p; 625-3p; 127-5p; 625-5p; 15b-3p; 299-5p; 3613-5p; 26b-5p; 432-5p; let-7b-5p; 206; 24-3p; 130b-5p; 3925-5p; 671-5p; 190a-5p; 454-3p; 329-3p; 889-3p; 99b-5p; 145-5p; 125a-5p; 377-3p; 99b-3p; 140-5p; 30d-5p; 296-5p; 193a-5p; 491-5p; 34c-5p; 9-5p; 301a-3p; 129-5p; 628-5p; 376c-3p; 1307-3p; 1271-5p; 204-5p; 296-3p; 125b-1-3p; 500a-5p; 378a-3p; 1185-5p; 543 |
| Neurotrophin signaling pathway | 5.12E-04 | 107 | 124 | 27a-5p; 133a-5p; 107; 483-5p; 342-3p; 181a-3p; 590-3p; 378a-5p; 1185-1-3p; 30a-3p; let-7e-5p; 320c; 320d; 92a-3p; let-7d-5p; 542-3p; 100-3p; 374a-5p; 31-5p; let-7a-5p; 197-3p; 320b; 22-3p; 486-3p; 25-3p; 149-5p; 181a-2-3p; 128-3p; 126-3p; 99a-5p; 205-5p; 1306-5p; 1-3p; 323a-3p; 374a-3p; 30e-5p; 130b-3p; 574-3p; 30c-5p; 485-3p; 98-5p; let-7f-5p; 15a-5p; 34a-5p; 330-5p; 7-5p; 411-5p; 665; 576-5p; 15b-5p; 103a-3p; let-7a-3p; 191-5p; 151a-3p; 184; 409-3p; 320a-3p; 382-5p; 130a-3p; 27b-3p; 423-5p; 374b-5p; 181b-5p; 584-5p; 23a-3p; 92b-3p; 93-3p; 17-5p; 484; 192-5p; 421; 497-5p; 154-3p; 26a-5p; 328-3p; 708-5p; 503-5p; 195-5p; 501-5p; 23b-3p; 133a-3p; 139-5p; 877-5p; 625-3p; 127-5p; 625-5p; 26b-5p; let-7b-5p; 206; 24-3p; 130b-5p; 598-3p; 3925-5p; 337-3p; 671-5p; 190a-5p; 454-3p; 329-3p; 889-3p; 145-5p; 125a-5p; 377-3p; 99b-3p; 376b-3p; 493-3p; 30d-5p; 296-5p; 193a-5p; 491-5p; 34c-5p; 9-5p; 301a-3p; 129-5p; 628-5p; 376c-3p; 1307-3p; 769-5p; 1271-5p; 204-5p; 296-3p; 125b-1-3p; 378a-3p; 1185-5p; 543 |
| Apoptosis | 6.27E-04 | 108 | 124 | 27a-5p; 107; 483-5p; 342-3p; 590-3p; 378a-5p; 1185-1-3p; 30a-3p; let-7e-5p; 362-5p; 320c; 320d; 92a-3p; let-7d-5p; 542-3p; 374a-5p; 31-5p; let-7a-5p; 197-3p; 320b; 22-3p; 486-3p; 25-3p; 149-5p; 181a-2-3p; 128-3p; 126-3p; 99a-5p; 323b-3p; 205-5p; 1306-5p; 629-5p; 1-3p; 323a-3p; 379-5p; 374a-3p; 30e-5p; 130b-3p; 30c-5p; 485-3p; 98-5p; let-7f-5p; 15a-5p; 34a-5p; 330-5p; 7-5p; 411-5p; 665; 15b-5p; 103a-3p; 151a-3p; 184; 409-3p; 320a-3p; 382-5p; let-7i-3p; 130a-3p; 27b-3p; 423-5p; 374b-5p; 181b-5p; 584-5p; 23a-3p; 22-5p; 92b-3p; 136-3p; 93-3p; 17-5p; 484; 192-5p; 421; 497-5p; 154-3p; 26a-5p; 708-5p; 503-5p; 195-5p; 23b-3p; 133a-3p; 139-5p; 877-5p; 625-3p; 127-5p; 625-5p; 664a-5p; 15b-3p; 3613-5p; 6511a-3p; 26b-5p; 432-5p; let-7b-5p; 206; 24-3p; 130b-5p; 3925-5p; 671-5p; 454-3p; 329-3p; 889-3p; 99b-5p; 145-5p; 125a-5p; 377-3p; 99b-3p; 376b-3p; 140-5p; 493-3p; 30d-5p; 296-5p; 193a-5p; 491-5p; 34c-5p; 9-5p; 301a-3p; 129-5p; 376c-3p; 1307-3p; 769-5p; 1271-5p; 204-5p; 296-3p; 125b-1-3p; 378a-3p; 543 |
| Tight junction | 9.32E-04 | 108 | 124 | 133a-5p; 107; 483-5p; 342-3p; 590-3p; 378a-5p; 30a-3p; let-7e-5p; 320c; 92a-3p; let-7d-5p; 542-3p; 374a-5p; 31-5p; let-7a-5p; 197-3p; 22-3p; 486-3p; 25-3p; 149-5p; 128-3p; 126-3p; 99a-5p; 323b-3p; 205-5p; 1306-5p; 629-5p; 1-3p; 323a-3p; 374a-3p; 30e-5p; 130b-3p; 574-3p; 30c-5p; 98-5p; let-7f-5p; 15a-5p; 34a-5p; 330-5p; 7-5p; 411-5p; 665; 576-5p; 15b-5p; 103a-3p; let-7a-3p; 191-5p; 127-3p; 7706; 184; 409-3p; 320a-3p; 382-5p; let-7i-3p; 130a-3p; 27b-3p; 423-5p; 374b-5p; 181b-5p; 584-5p; 23a-3p; 22-5p; 92b-3p; 93-3p; 17-5p; 484; 192-5p; 421; 497-5p; 154-3p; 26a-5p; 328-3p; 708-5p; 503-5p; 675-3p; 195-5p; 941; 23b-3p; 133a-3p; 139-5p; 877-5p; 625-3p; 127-5p; 625-5p; 15b-3p; 3613-5p; 6511a-3p; 26b-5p; 432-5p; let-7b-5p; 206; 24-3p; 130b-5p; 3925-5p; 337-3p; 671-5p; 454-3p; 329-3p; 889-3p; 99b-5p; 145-5p; 125a-5p; 377-3p; 99b-3p; 376b-3p; 493-3p; 30d-5p; 296-5p; 193a-5p; 491-5p; 34c-5p; 9-5p; 301a-3p; 129-5p; 376c-3p; 1307-3p; 769-5p; 1271-5p; 204-5p; 296-3p; 125b-1-3p; 500a-5p; 378a-3p; 1185-5p |
| Glioma | 5.00E-05 | 103 | 123 | 27a-5p; 133a-5p; 107; 483-5p; 342-3p; 590-3p; 378a-5p; 1185-1-3p; 30a-3p; let-7e-5p; 92a-3p; let-7d-5p; 542-3p; 100-3p; 374a-5p; 31-5p; let-7a-5p; 197-3p; 320b; 22-3p; 486-3p; 25-3p; 149-5p; 181a-2-3p; 128-3p; 126-3p; 99a-5p; 323b-3p; 205-5p; 1-3p; 323a-3p; 374a-3p; 30e-5p; 130b-3p; 574-3p; 30c-5p; 98-5p; let-7f-5p; 15a-5p; 34a-5p; 330-5p; 7-5p; 411-5p; 665; 576-5p; 15b-5p; 103a-3p; let-7a-3p; 191-5p; 7706; 184; 409-3p; 320a-3p; 382-5p; 130a-3p; 27b-3p; 423-5p; 374b-5p; 181b-5p; 584-5p; 23a-3p; 92b-3p; 93-3p; 17-5p; 484; 192-5p; 421; 497-5p; 154-3p; 26a-5p; 708-5p; 503-5p; 675-3p; 195-5p; 23b-3p; 7704; 133a-3p; 139-5p; 877-5p; 625-3p; 127-5p; 625-5p; 15b-3p; 299-5p; 3613-5p; 26b-5p; 432-5p; let-7b-5p; 206; 24-3p; 130b-5p; 598-3p; 3925-5p; 671-5p; 190a-5p; 454-3p; 329-3p; 889-3p; 99b-5p; 145-5p; 125a-5p; 377-3p; 376b-3p; 140-5p; 493-3p; 30d-5p; 193a-5p; 491-5p; 34c-5p; 9-5p; 301a-3p; 129-5p; 628-5p; 376c-3p; 1307-3p; 769-5p; 1271-5p; 204-5p; 1287-5p; 296-3p; 125b-1-3p; 378a-3p; 543 |
| cGMP-PKG signaling pathway | 5.00E-05 | 103 | 123 | 27a-5p; 133a-5p; 107; 483-5p; 342-3p; let-7d-3p; 590-3p; 378a-5p; 1185-1-3p; 30a-3p; let-7e-5p; 320c; 320d; 92a-3p; let-7d-5p; 542-3p; 100-3p; 374a-5p; 31-5p; let-7a-5p; 197-3p; 320b; 22-3p; 486-3p; 25-3p; 149-5p; 181a-2-3p; 128-3p; 126-3p; 99a-5p; 323b-3p; 205-5p; 1-3p; 323a-3p; 374a-3p; 30e-5p; 130b-3p; 30c-5p; 98-5p; let-7f-5p; 15a-5p; 34a-5p; 330-5p; 7-5p; 411-5p; 665; 576-5p; 15b-5p; 103a-3p; let-7a-3p; 191-5p; 127-3p; 184; 409-3p; 320a-3p; 382-5p; 130a-3p; 27b-3p; 423-5p; 374b-5p; 181b-5p; 584-5p; 23a-3p; 22-5p; 92b-3p; 136-3p; 93-3p; 17-5p; 484; 192-5p; 421; 497-5p; 154-3p; 26a-5p; 708-5p; 503-5p; 195-5p; 941; 501-5p; 23b-3p; 7704; 133a-3p; 139-5p; 877-5p; 625-3p; 127-5p; 625-5p; 664a-5p; 6511a-3p; 26b-5p; 432-5p; let-7b-5p; 206; 24-3p; 130b-5p; 598-3p; 3925-5p; 337-3p; 671-5p; 190a-5p; 454-3p; 329-3p; 889-3p; 99b-5p; 145-5p; 125a-5p; 377-3p; 493-3p; 30d-5p; 34c-5p; 9-5p; 301a-3p; 129-5p; 628-5p; 1307-3p; 769-5p; 1271-5p; 204-5p; 296-3p; 500a-5p; 378a-3p; 1185-5p; 543 |
| Insulin signaling pathway | 7.04E-05 | 103 | 123 | 27a-5p; 107; 483-5p; 342-3p; 590-3p; 378a-5p; 1185-1-3p; 30a-3p; let-7e-5p; 362-5p; 320c; 320d; 92a-3p; let-7d-5p; 542-3p; 100-3p; 374a-5p; 132-5p; let-7a-5p; 197-3p; 320b; 22-3p; 486-3p; 25-3p; 149-5p; 128-3p; 126-3p; 99a-5p; 205-5p; 1306-5p; 1-3p; 323a-3p; 374a-3p; 30e-5p; 130b-3p; 30c-5p; 485-3p; 98-5p; let-7f-5p; 15a-5p; 34a-5p; 330-5p; 7-5p; 411-5p; 665; 576-5p; 15b-5p; 103a-3p; let-7a-3p; 191-5p; 151a-3p; 184; 409-3p; 320a-3p; 382-5p; 130a-3p; 27b-3p; 423-5p; 374b-5p; 181b-5p; 584-5p; 23a-3p; 92b-3p; 136-3p; 93-3p; 17-5p; 484; 192-5p; 421; 497-5p; 154-3p; 26a-5p; 708-5p; 503-5p; 675-3p; 195-5p; 941; 501-5p; 23b-3p; 133a-3p; 139-5p; 877-5p; 625-3p; 127-5p; 625-5p; 15b-3p; 3613-5p; 26b-5p; 432-5p; let-7b-5p; 206; 24-3p; 130b-5p; 598-3p; 3925-5p; 671-5p; 190a-5p; 454-3p; 329-3p; 889-3p; 99b-5p; 145-5p; 125a-5p; 377-3p; 99b-3p; 376b-3p; 493-3p; 30d-5p; 193a-5p; 34c-5p; 9-5p; 301a-3p; 129-5p; 628-5p; 376c-3p; 1307-3p; 769-5p; 1271-5p; 204-5p; 296-3p; 500a-5p; 378a-3p; 543 |
| Protein processing in endoplasmic reticulum | 2.27E-03 | 109 | 123 | 107; 483-5p; 342-3p; 590-3p; 378a-5p; 1185-1-3p; 30a-3p; let-7e-5p; 362-5p; 320c; 320d; 92a-3p; let-7d-5p; 100-3p; 374a-5p; 31-5p; 132-5p; let-7a-5p; 197-3p; 320b; 22-3p; 486-3p; 25-3p; 149-5p; 181a-2-3p; 128-3p; 126-3p; 99a-5p; 323b-3p; 205-5p; 1306-5p; 1-3p; 323a-3p; 379-5p; 374a-3p; 30e-5p; 130b-3p; 30c-5p; 98-5p; let-7f-5p; 15a-5p; 34a-5p; 330-5p; 7-5p; 411-5p; 665; 576-5p; 15b-5p; 103a-3p; let-7a-3p; 191-5p; 127-3p; 184; 409-3p; 320a-3p; 382-5p; 130a-3p; 27b-3p; 423-5p; 374b-5p; 181b-5p; 584-5p; 23a-3p; 22-5p; 92b-3p; 136-3p; 93-3p; 17-5p; 484; 192-5p; 421; 497-5p; 26a-5p; 328-3p; 708-5p; 503-5p; 195-5p; 941; 501-5p; 23b-3p; 7704; 133a-3p; 139-5p; 877-5p; 625-3p; 127-5p; 625-5p; 664a-5p; 299-5p; 6511a-3p; 26b-5p; 432-5p; let-7b-5p; 206; 24-3p; 130b-5p; 671-5p; 454-3p; 329-3p; 889-3p; 125a-5p; 377-3p; 376b-3p; 140-5p; 493-3p; 30d-5p; 34c-5p; 9-5p; 301a-3p; 129-5p; 628-5p; 376c-3p; 1307-3p; 769-5p; 1271-5p; 204-5p; 1287-5p; 296-3p; 125b-1-3p; 500a-5p; 378a-3p; 1185-5p; 543 |
| Signaling pathways regulating pluripotency of stem cells | 6.47E-03 | 110 | 123 | 27a-5p; 107; 483-5p; 342-3p; 181a-3p; 590-3p; 378a-5p; 1185-1-3p; 30a-3p; let-7e-5p; 320c; 92a-3p; let-7d-5p; 542-3p; 100-3p; 374a-5p; 31-5p; let-7a-5p; 197-3p; 320b; 22-3p; 486-3p; 25-3p; 149-5p; 181a-2-3p; 128-3p; 126-3p; 99a-5p; 205-5p; 1-3p; 323a-3p; 379-5p; 374a-3p; 30e-5p; 130b-3p; 574-3p; 30c-5p; 485-3p; 98-5p; let-7f-5p; 15a-5p; 34a-5p; 330-5p; 7-5p; 411-5p; 665; 576-5p; 15b-5p; 103a-3p; let-7a-3p; 7706; 184; 409-3p; 320a-3p; 130a-3p; 27b-3p; 423-5p; 374b-5p; 181b-5p; 584-5p; 23a-3p; 92b-3p; 136-3p; 93-3p; 17-5p; 484; 192-5p; 421; 497-5p; 154-3p; 26a-5p; 708-5p; 503-5p; 675-3p; 195-5p; 501-5p; 23b-3p; 133a-3p; 139-5p; 877-5p; 625-3p; 625-5p; 664a-5p; 15b-3p; 3613-5p; 26b-5p; 432-5p; let-7b-5p; 206; 24-3p; 130b-5p; 3925-5p; 337-3p; 671-5p; 190a-5p; 454-3p; 329-3p; 889-3p; 99b-5p; 145-5p; 125a-5p; 377-3p; 99b-3p; 376b-3p; 140-5p; 493-3p; 30d-5p; 296-5p; 193a-5p; 491-5p; 34c-5p; 9-5p; 301a-3p; 129-5p; 376c-3p; 1307-3p; 769-5p; 1271-5p; 204-5p; 296-3p; 125b-1-3p; 378a-3p; 543 |
| Colorectal cancer | 2.73E-04 | 104 | 122 | 27a-5p; 133a-5p; 107; 483-5p; 342-3p; 590-3p; 378a-5p; 1185-1-3p; 30a-3p; let-7e-5p; 362-5p; 320c; 92a-3p; let-7d-5p; 542-3p; 374a-5p; 31-5p; let-7a-5p; 197-3p; 320b; 22-3p; 486-3p; 25-3p; 149-5p; 128-3p; 126-3p; 99a-5p; 323b-3p; 205-5p; 1306-5p; 1-3p; 323a-3p; 379-5p; 374a-3p; 30e-5p; 130b-3p; 574-3p; 30c-5p; 485-3p; 98-5p; let-7f-5p; 15a-5p; 34a-5p; 330-5p; 7-5p; 411-5p; 665; 576-5p; 15b-5p; 103a-3p; let-7a-3p; 191-5p; 7706; 184; 409-3p; 320a-3p; 382-5p; 130a-3p; 27b-3p; 423-5p; 374b-5p; 181b-5p; 584-5p; 23a-3p; 22-5p; 92b-3p; 93-3p; 17-5p; 484; 192-5p; 421; 497-5p; 154-3p; 26a-5p; 708-5p; 503-5p; 195-5p; 501-5p; 23b-3p; 7704; 133a-3p; 139-5p; 877-5p; 625-3p; 625-5p; 15b-3p; 299-5p; 6511a-3p; 26b-5p; let-7b-5p; 206; 24-3p; 130b-5p; 3925-5p; 671-5p; 454-3p; 329-3p; 889-3p; 99b-5p; 145-5p; 125a-5p; 377-3p; 99b-3p; 140-5p; 30d-5p; 296-5p; 193a-5p; 491-5p; 34c-5p; 9-5p; 301a-3p; 129-5p; 376c-3p; 1307-3p; 769-5p; 1271-5p; 204-5p; 296-3p; 125b-1-3p; 500a-5p; 378a-3p; 543 |
| Autophagy - animal | 1.46E-02 | 111 | 122 | 27a-5p; 133a-5p; 107; 483-5p; 342-3p; 181a-3p; 590-3p; 1185-1-3p; 30a-3p; let-7e-5p; 362-5p; 320c; 320d; 92a-3p; let-7d-5p; 542-3p; 374a-5p; let-7a-5p; 197-3p; 320b; 22-3p; 486-3p; 25-3p; 149-5p; 181a-2-3p; 128-3p; 126-3p; 99a-5p; 205-5p; 629-5p; 1-3p; 374a-3p; 30e-5p; 130b-3p; 30c-5p; 98-5p; let-7f-5p; 15a-5p; 34a-5p; 330-5p; 7-5p; 411-5p; 665; 576-5p; 15b-5p; 103a-3p; let-7a-3p; 191-5p; 151a-3p; 184; 409-3p; 320a-3p; 382-5p; 130a-3p; 27b-3p; 423-5p; 374b-5p; 181b-5p; 584-5p; 23a-3p; 92b-3p; 93-3p; 17-5p; 484; 192-5p; 421; 497-5p; 154-3p; 26a-5p; 328-3p; 708-5p; 503-5p; 675-3p; 195-5p; 23b-3p; 133a-3p; 139-5p; 877-5p; 625-3p; 127-5p; 625-5p; 15b-3p; 299-5p; 3613-5p; 26b-5p; 432-5p; let-7b-5p; 206; 24-3p; 130b-5p; 3925-5p; 671-5p; 454-3p; 329-3p; 889-3p; 99b-5p; 145-5p; 125a-5p; 377-3p; 376b-3p; 140-5p; 493-3p; 30d-5p; 193a-5p; 491-5p; 34c-5p; 9-5p; 301a-3p; 129-5p; 628-5p; 376c-3p; 1307-3p; 769-5p; 1271-5p; 204-5p; 1287-5p; 296-3p; 125b-1-3p; 500a-5p; 378a-3p; 1185-5p; 543 |
| Estrogen signaling pathway | 7.46E-06 | 98 | 121 | 27a-5p; 133a-5p; 107; 483-5p; 342-3p; 590-3p; 378a-5p; 1185-1-3p; 30a-3p; let-7e-5p; 362-5p; 320c; 320d; 92a-3p; let-7d-5p; 542-3p; 374a-5p; 31-5p; let-7a-5p; 197-3p; 320b; 22-3p; 486-3p; 25-3p; 149-5p; 128-3p; 126-3p; 99a-5p; 323b-3p; 205-5p; 1-3p; 379-5p; 374a-3p; 30e-5p; 130b-3p; 574-3p; 30c-5p; 98-5p; let-7f-5p; 15a-5p; 34a-5p; 330-5p; 7-5p; 411-5p; 665; 576-5p; 15b-5p; 103a-3p; let-7a-3p; 151a-3p; 184; 409-3p; 320a-3p; 382-5p; 130a-3p; 27b-3p; 423-5p; 374b-5p; 181b-5p; 584-5p; 23a-3p; 92b-3p; 93-3p; 17-5p; 484; 192-5p; 421; 497-5p; 154-3p; 26a-5p; 328-3p; 708-5p; 503-5p; 195-5p; 941; 501-5p; 23b-3p; 133a-3p; 139-5p; 877-5p; 625-3p; 625-5p; 6511a-3p; 26b-5p; 432-5p; let-7b-5p; 206; 24-3p; 130b-5p; 598-3p; 3925-5p; 337-3p; 671-5p; 190a-5p; 454-3p; 329-3p; 889-3p; 99b-5p; 145-5p; 125a-5p; 99b-3p; 376b-3p; 140-5p; 493-3p; 30d-5p; 296-5p; 193a-5p; 491-5p; 34c-5p; 9-5p; 301a-3p; 129-5p; 376c-3p; 1307-3p; 769-5p; 204-5p; 296-3p; 500a-5p; 378a-3p; 1185-5p; 543 |
| Pancreatic cancer | 3.54E-05 | 100 | 121 | 27a-5p; 133a-5p; 107; 483-5p; 342-3p; 590-3p; 378a-5p; 1185-1-3p; 30a-3p; let-7e-5p; 362-5p; 92a-3p; let-7d-5p; 542-3p; 100-3p; 374a-5p; 31-5p; let-7a-5p; 197-3p; 320b; 22-3p; 486-3p; 25-3p; 149-5p; 128-3p; 126-3p; 99a-5p; 323b-3p; 205-5p; 1-3p; 323a-3p; 379-5p; 374a-3p; 30e-5p; 130b-3p; 574-3p; 30c-5p; 98-5p; let-7f-5p; 15a-5p; 34a-5p; 330-5p; 7-5p; 665; 576-5p; 15b-5p; 103a-3p; let-7a-3p; 191-5p; 151a-3p; 7706; 184; 409-3p; 320a-3p; 130a-3p; 27b-3p; 423-5p; 374b-5p; 181b-5p; 584-5p; 23a-3p; 22-5p; 92b-3p; 93-3p; 17-5p; 484; 192-5p; 421; 497-5p; 154-3p; 26a-5p; 328-3p; 708-5p; 503-5p; 195-5p; 501-5p; 23b-3p; 7704; 133a-3p; 139-5p; 877-5p; 625-3p; 625-5p; 299-5p; 3613-5p; 26b-5p; let-7b-5p; 206; 24-3p; 130b-5p; 3925-5p; 337-3p; 671-5p; 454-3p; 329-3p; 889-3p; 99b-5p; 145-5p; 125a-5p; 377-3p; 376b-3p; 140-5p; 493-3p; 30d-5p; 296-5p; 193a-5p; 491-5p; 34c-5p; 9-5p; 301a-3p; 129-5p; 376c-3p; 1307-3p; 769-5p; 1271-5p; 204-5p; 296-3p; 125b-1-3p; 500a-5p; 378a-3p; 543 |
| Fluid shear stress and atherosclerosis | 6.27E-05 | 101 | 121 | 27a-5p; 133a-5p; 107; 483-5p; 342-3p; 590-3p; 1185-1-3p; 30a-3p; let-7e-5p; 362-5p; 320c; 92a-3p; let-7d-5p; 542-3p; 100-3p; 374a-5p; 31-5p; 132-5p; let-7a-5p; 197-3p; 22-3p; 486-3p; 25-3p; 149-5p; 181a-2-3p; 128-3p; 126-3p; 99a-5p; 323b-3p; 205-5p; 1-3p; 379-5p; 374a-3p; 30e-5p; 130b-3p; 574-3p; 30c-5p; 98-5p; let-7f-5p; 15a-5p; 34a-5p; 330-5p; 7-5p; 411-5p; 665; 576-5p; 15b-5p; 103a-3p; let-7a-3p; 191-5p; 151a-3p; 184; 409-3p; 320a-3p; 382-5p; let-7i-3p; 130a-3p; 27b-3p; 423-5p; 374b-5p; 181b-5p; 23a-3p; 22-5p; 92b-3p; 93-3p; 17-5p; 484; 192-5p; 421; 497-5p; 154-3p; 26a-5p; 328-3p; 708-5p; 503-5p; 675-3p; 195-5p; 501-5p; 23b-3p; 133a-3p; 139-5p; 877-5p; 625-3p; 127-5p; 625-5p; 3613-5p; 26b-5p; let-7b-5p; 206; 24-3p; 130b-5p; 598-3p; 3925-5p; 190a-5p; 454-3p; 329-3p; 889-3p; 99b-5p; 145-5p; 125a-5p; 377-3p; 99b-3p; 140-5p; 493-3p; 30d-5p; 296-5p; 193a-5p; 491-5p; 34c-5p; 9-5p; 301a-3p; 129-5p; 376c-3p; 1307-3p; 769-5p; 204-5p; 296-3p; 125b-1-3p; 500a-5p; 378a-3p; 543 |
| Measles | 2.63E-03 | 107 | 121 | 27a-5p; 107; 483-5p; 342-3p; 590-3p; 378a-5p; 1185-1-3p; 30a-3p; let-7e-5p; 362-5p; 320c; 320d; 92a-3p; let-7d-5p; 542-3p; 374a-5p; let-7a-5p; 197-3p; 320b; 22-3p; 25-3p; 149-5p; 128-3p; 126-3p; 99a-5p; 323b-3p; 205-5p; 1306-5p; 1-3p; 323a-3p; 374a-3p; 30e-5p; 130b-3p; 30c-5p; 485-3p; 98-5p; let-7f-5p; 15a-5p; 34a-5p; 330-5p; 7-5p; 411-5p; 665; 15b-5p; 103a-3p; let-7a-3p; 191-5p; 151a-3p; 7706; 184; 409-3p; 320a-3p; 382-5p; 130a-3p; 27b-3p; 423-5p; 374b-5p; 181b-5p; 6724-5p; 23a-3p; 92b-3p; 93-3p; 17-5p; 484; 192-5p; 421; 497-5p; 26a-5p; 328-3p; 708-5p; 503-5p; 195-5p; 941; 501-5p; 23b-3p; 7704; 133a-3p; 139-5p; 877-5p; 625-3p; 625-5p; 664a-5p; 15b-3p; 3613-5p; 6511a-3p; 26b-5p; 432-5p; let-7b-5p; 206; 24-3p; 130b-5p; 3925-5p; 337-3p; 454-3p; 329-3p; 145-5p; 125a-5p; 377-3p; 99b-3p; 376b-3p; 140-5p; 30d-5p; 296-5p; 193a-5p; 491-5p; 34c-5p; 9-5p; 301a-3p; 129-5p; 628-5p; 376c-3p; 1307-3p; 769-5p; 1271-5p; 204-5p; 296-3p; 125b-1-3p; 500a-5p; 378a-3p; 1185-5p; 543 |
| JAK-STAT signaling pathway | 2.87E-03 | 107 | 121 | 27a-5p; 133a-5p; 107; 483-5p; 342-3p; 590-3p; 378a-5p; 30a-3p; let-7e-5p; 320c; 320d; 92a-3p; let-7d-5p; 542-3p; 100-3p; 374a-5p; 31-5p; let-7a-5p; 197-3p; 320b; 22-3p; 486-3p; 25-3p; 149-5p; 128-3p; 126-3p; 99a-5p; 323b-3p; 205-5p; 1306-5p; 1-3p; 323a-3p; 379-5p; 374a-3p; 30e-5p; 130b-3p; 574-3p; 30c-5p; 485-3p; 98-5p; let-7f-5p; 15a-5p; 34a-5p; 330-5p; 7-5p; 411-5p; 665; 576-5p; 15b-5p; 103a-3p; let-7a-3p; 191-5p; 151a-3p; 7706; 184; 409-3p; 320a-3p; 382-5p; 130a-3p; 27b-3p; 423-5p; 374b-5p; 628-3p; 181b-5p; 584-5p; 23a-3p; 92b-3p; 93-3p; 17-5p; 484; 192-5p; 497-5p; 26a-5p; 708-5p; 503-5p; 195-5p; 501-5p; 23b-3p; 7704; 133a-3p; 139-5p; 877-5p; 625-3p; 625-5p; 15b-3p; 299-5p; 6511a-3p; 26b-5p; 432-5p; let-7b-5p; 206; 24-3p; 130b-5p; 337-3p; 190a-5p; 454-3p; 329-3p; 99b-5p; 145-5p; 125a-5p; 377-3p; 376b-3p; 140-5p; 493-3p; 30d-5p; 193a-5p; 491-5p; 34c-5p; 9-5p; 301a-3p; 129-5p; 628-5p; 376c-3p; 1307-3p; 769-5p; 1271-5p; 204-5p; 296-3p; 500a-5p; 378a-3p; 543 |
| Adherens junction | 6.79E-07 | 94 | 120 | 27a-5p; 133a-5p; 107; 483-5p; 342-3p; 590-3p; 378a-5p; 1185-1-3p; 30a-3p; let-7e-5p; 320c; 92a-3p; let-7d-5p; 542-3p; 100-3p; 374a-5p; 31-5p; let-7a-5p; 197-3p; 320b; 22-3p; 486-3p; 25-3p; 149-5p; 128-3p; 99a-5p; 205-5p; 629-5p; 1-3p; 323a-3p; 379-5p; 374a-3p; 30e-5p; 130b-3p; 574-3p; 30c-5p; 485-3p; 98-5p; let-7f-5p; 15a-5p; 34a-5p; 7-5p; 411-5p; 576-5p; 15b-5p; 103a-3p; let-7a-3p; 151a-3p; 7706; 409-3p; 320a-3p; let-7i-3p; 130a-3p; 27b-3p; 423-5p; 374b-5p; 628-3p; 181b-5p; 584-5p; 23a-3p; 22-5p; 92b-3p; 136-3p; 93-3p; 17-5p; 484; 192-5p; 421; 497-5p; 154-3p; 26a-5p; 328-3p; 708-5p; 503-5p; 675-3p; 195-5p; 23b-3p; 133a-3p; 139-5p; 877-5p; 625-3p; 127-5p; 625-5p; 15b-3p; 6511a-3p; 26b-5p; let-7b-5p; 206; 24-3p; 130b-5p; 3925-5p; 337-3p; 190a-5p; 454-3p; 329-3p; 99b-5p; 145-5p; 125a-5p; 377-3p; 99b-3p; 140-5p; 493-3p; 30d-5p; 296-5p; 193a-5p; 491-5p; 34c-5p; 9-5p; 301a-3p; 129-5p; 376c-3p; 1307-3p; 769-5p; 1271-5p; 204-5p; 296-3p; 125b-1-3p; 378a-3p; 1185-5p; 543 |
| ErbB signaling pathway | 1.01E-05 | 97 | 120 | 27a-5p; 133a-5p; 107; 483-5p; 342-3p; 590-3p; 378a-5p; 1185-1-3p; 30a-3p; let-7e-5p; 362-5p; 320c; 320d; 92a-3p; let-7d-5p; 542-3p; 374a-5p; 31-5p; let-7a-5p; 197-3p; 320b; 22-3p; 486-3p; 25-3p; 149-5p; 128-3p; 126-3p; 99a-5p; 323b-3p; 205-5p; 1306-5p; 1-3p; 323a-3p; 379-5p; 374a-3p; 30e-5p; 130b-3p; 574-3p; 30c-5p; 98-5p; let-7f-5p; 15a-5p; 34a-5p; 330-5p; 7-5p; 411-5p; 665; 576-5p; 15b-5p; 103a-3p; let-7a-3p; 191-5p; 184; 409-3p; 320a-3p; 130a-3p; 27b-3p; 423-5p; 374b-5p; 181b-5p; 584-5p; 23a-3p; 22-5p; 92b-3p; 93-3p; 17-5p; 484; 192-5p; 421; 497-5p; 154-3p; 26a-5p; 708-5p; 503-5p; 195-5p; 501-5p; 23b-3p; 7704; 133a-3p; 139-5p; 877-5p; 625-3p; 127-5p; 625-5p; 299-5p; 26b-5p; let-7b-5p; 206; 24-3p; 130b-5p; 3925-5p; 671-5p; 454-3p; 329-3p; 889-3p; 99b-5p; 145-5p; 125a-5p; 377-3p; 99b-3p; 376b-3p; 493-3p; 30d-5p; 193a-5p; 491-5p; 34c-5p; 9-5p; 301a-3p; 129-5p; 376c-3p; 1307-3p; 769-5p; 1271-5p; 204-5p; 296-3p; 125b-1-3p; 500a-5p; 378a-3p; 1185-5p; 543 |
| Tuberculosis | 6.57E-05 | 100 | 120 | 27a-5p; 133a-5p; 107; 483-5p; 590-3p; 378a-5p; 1185-1-3p; 30a-3p; let-7e-5p; 362-5p; 320c; 92a-3p; let-7d-5p; 542-3p; 374a-5p; 31-5p; let-7a-5p; 197-3p; 320b; 22-3p; 486-3p; 25-3p; 149-5p; 181a-2-3p; 128-3p; 126-3p; 99a-5p; 205-5p; 1306-5p; 629-5p; 1-3p; 374a-3p; 30e-5p; 130b-3p; 574-3p; 30c-5p; 485-3p; 98-5p; let-7f-5p; 15a-5p; 34a-5p; 330-5p; 7-5p; 665; 576-5p; 15b-5p; 103a-3p; let-7a-3p; 191-5p; 184; 409-3p; 320a-3p; 382-5p; 130a-3p; 27b-3p; 423-5p; 374b-5p; 628-3p; 181b-5p; 584-5p; 23a-3p; 22-5p; 92b-3p; 93-3p; 17-5p; 484; 192-5p; 421; 497-5p; 154-3p; 26a-5p; 328-3p; 708-5p; 503-5p; 195-5p; 501-5p; 23b-3p; 7704; 133a-3p; 139-5p; 877-5p; 625-3p; 127-5p; 625-5p; 664a-5p; 6511a-3p; 26b-5p; let-7b-5p; 206; 24-3p; 130b-5p; 598-3p; 3925-5p; 190a-5p; 454-3p; 329-3p; 145-5p; 125a-5p; 377-3p; 99b-3p; 376b-3p; 140-5p; 493-3p; 30d-5p; 296-5p; 193a-5p; 491-5p; 34c-5p; 9-5p; 301a-3p; 129-5p; 628-5p; 376c-3p; 1307-3p; 769-5p; 1271-5p; 204-5p; 500a-5p; 378a-3p; 543 |
| Melanoma | 1.42E-04 | 101 | 120 | 27a-5p; 133a-5p; 107; 483-5p; 342-3p; 590-3p; 378a-5p; 1185-1-3p; 30a-3p; let-7e-5p; 92a-3p; let-7d-5p; 542-3p; 100-3p; 374a-5p; 31-5p; let-7a-5p; 197-3p; 320b; 22-3p; 486-3p; 25-3p; 149-5p; 128-3p; 126-3p; 99a-5p; 323b-3p; 205-5p; 1-3p; 374a-3p; 30e-5p; 130b-3p; 574-3p; 30c-5p; 98-5p; let-7f-5p; 15a-5p; 34a-5p; 330-5p; 7-5p; 665; 576-5p; 15b-5p; 103a-3p; let-7a-3p; 191-5p; 7706; 184; 409-3p; 320a-3p; 382-5p; 130a-3p; 27b-3p; 423-5p; 374b-5p; 181b-5p; 584-5p; 23a-3p; 92b-3p; 93-3p; 17-5p; 484; 192-5p; 421; 497-5p; 154-3p; 26a-5p; 328-3p; 708-5p; 503-5p; 675-3p; 195-5p; 23b-3p; 7704; 133a-3p; 139-5p; 877-5p; 625-3p; 127-5p; 625-5p; 15b-3p; 299-5p; 3613-5p; 26b-5p; 432-5p; let-7b-5p; 206; 24-3p; 130b-5p; 3925-5p; 671-5p; 190a-5p; 454-3p; 329-3p; 889-3p; 99b-5p; 145-5p; 125a-5p; 377-3p; 376b-3p; 140-5p; 30d-5p; 296-5p; 193a-5p; 491-5p; 34c-5p; 9-5p; 301a-3p; 129-5p; 628-5p; 376c-3p; 1307-3p; 769-5p; 1271-5p; 204-5p; 1287-5p; 296-3p; 125b-1-3p; 378a-3p; 543 |
| Yersinia infection | 1.71E-03 | 105 | 120 | 27a-5p; 133a-5p; 107; 483-5p; 342-3p; 590-3p; 378a-5p; 1185-1-3p; 30a-3p; let-7e-5p; 320c; 320d; 92a-3p; let-7d-5p; 542-3p; 100-3p; 374a-5p; 31-5p; let-7a-5p; 197-3p; 320b; 22-3p; 25-3p; 149-5p; 181a-2-3p; 128-3p; 126-3p; 99a-5p; 323b-3p; 205-5p; 1306-5p; 1-3p; 323a-3p; 379-5p; 374a-3p; 30e-5p; 130b-3p; 574-3p; 30c-5p; 98-5p; let-7f-5p; 15a-5p; 34a-5p; 330-5p; 7-5p; 411-5p; 665; 576-5p; 15b-5p; 103a-3p; let-7a-3p; 191-5p; 127-3p; 151a-3p; 7706; 184; 409-3p; 320a-3p; let-7i-3p; 130a-3p; 27b-3p; 423-5p; 374b-5p; 181b-5p; 584-5p; 23a-3p; 92b-3p; 93-3p; 17-5p; 484; 192-5p; 421; 497-5p; 154-3p; 26a-5p; 328-3p; 708-5p; 503-5p; 195-5p; 501-5p; 23b-3p; 133a-3p; 139-5p; 877-5p; 625-3p; 127-5p; 6511a-3p; 26b-5p; let-7b-5p; 206; 24-3p; 130b-5p; 3925-5p; 337-3p; 671-5p; 454-3p; 329-3p; 889-3p; 99b-5p; 145-5p; 125a-5p; 377-3p; 99b-3p; 493-3p; 30d-5p; 193a-5p; 491-5p; 34c-5p; 9-5p; 301a-3p; 129-5p; 628-5p; 376c-3p; 1307-3p; 769-5p; 204-5p; 296-3p; 125b-1-3p; 378a-3p; 543 |
| HIF-1 signaling pathway | 4.15E-03 | 106 | 120 | 27a-5p; 133a-5p; 107; 483-5p; 342-3p; 590-3p; 378a-5p; 1185-1-3p; 30a-3p; let-7e-5p; 362-5p; 320c; 92a-3p; let-7d-5p; 542-3p; 374a-5p; let-7a-5p; 197-3p; 320b; 22-3p; 486-3p; 25-3p; 149-5p; 181a-2-3p; 128-3p; 126-3p; 99a-5p; 205-5p; 1-3p; 323a-3p; 379-5p; 374a-3p; 30e-5p; 130b-3p; 574-3p; 30c-5p; 98-5p; let-7f-5p; 15a-5p; 34a-5p; 330-5p; 7-5p; 665; 576-5p; 15b-5p; 103a-3p; let-7a-3p; 191-5p; 151a-3p; 184; 409-3p; 320a-3p; 130a-3p; 27b-3p; 423-5p; 374b-5p; 628-3p; 181b-5p; 584-5p; 23a-3p; 22-5p; 92b-3p; 93-3p; 17-5p; 484; 192-5p; 421; 497-5p; 154-3p; 26a-5p; 708-5p; 503-5p; 675-3p; 195-5p; 501-5p; 23b-3p; 7704; 133a-3p; 139-5p; 877-5p; 625-3p; 625-5p; 15b-3p; 299-5p; 26b-5p; 432-5p; let-7b-5p; 206; 24-3p; 130b-5p; 337-3p; 190a-5p; 454-3p; 329-3p; 889-3p; 99b-5p; 145-5p; 125a-5p; 377-3p; 140-5p; 493-3p; 30d-5p; 296-5p; 193a-5p; 491-5p; 34c-5p; 9-5p; 301a-3p; 129-5p; 376c-3p; 1307-3p; 769-5p; 1271-5p; 204-5p; 296-3p; 125b-1-3p; 500a-5p; 378a-3p; 1185-5p; 543 |
| Wnt signaling pathway | 5.80E-03 | 107 | 120 | 27a-5p; 133a-5p; 107; 483-5p; 342-3p; 590-3p; 378a-5p; 1185-1-3p; 30a-3p; let-7e-5p; 320c; 92a-3p; let-7d-5p; 542-3p; 374a-5p; 31-5p; let-7a-5p; 197-3p; 320b; 22-3p; 486-3p; 25-3p; 149-5p; 181a-2-3p; 128-3p; 126-3p; 323b-3p; 205-5p; 629-5p; 1-3p; 323a-3p; 374a-3p; 30e-5p; 130b-3p; 574-3p; 30c-5p; 485-3p; 98-5p; let-7f-5p; 15a-5p; 34a-5p; 330-5p; 7-5p; 411-5p; 665; 576-5p; 15b-5p; 103a-3p; let-7a-3p; 191-5p; 127-3p; 7706; 184; 409-3p; 320a-3p; 382-5p; 130a-3p; 27b-3p; 423-5p; 374b-5p; 628-3p; 181b-5p; 23a-3p; 92b-3p; 136-3p; 93-3p; 17-5p; 484; 192-5p; 421; 497-5p; 154-3p; 26a-5p; 328-3p; 708-5p; 503-5p; 195-5p; 501-5p; 23b-3p; 139-5p; 877-5p; 625-3p; 127-5p; 625-5p; 15b-3p; 26b-5p; let-7b-5p; 206; 24-3p; 130b-5p; 337-3p; 671-5p; 190a-5p; 454-3p; 329-3p; 145-5p; 125a-5p; 377-3p; 99b-3p; 376b-3p; 140-5p; 493-3p; 30d-5p; 193a-5p; 491-5p; 34c-5p; 9-5p; 301a-3p; 129-5p; 376c-3p; 769-5p; 1271-5p; 204-5p; 1287-5p; 296-3p; 125b-1-3p; 500a-5p; 378a-3p; 1185-5p; 543 |
| Chagas disease American trypanosomiasis | 1.22E-09 | 86 | 119 | 27a-5p; 107; 483-5p; 342-3p; 590-3p; 378a-5p; 1185-1-3p; 30a-3p; let-7e-5p; 362-5p; 320c; 320d; 92a-3p; let-7d-5p; 542-3p; 100-3p; 374a-5p; 31-5p; let-7a-5p; 197-3p; 320b; 22-3p; 486-3p; 25-3p; 149-5p; 181a-2-3p; 128-3p; 126-3p; 99a-5p; 323b-3p; 205-5p; 1306-5p; 629-5p; 1-3p; 323a-3p; 379-5p; 374a-3p; 30e-5p; 130b-3p; 574-3p; 30c-5p; 485-3p; 98-5p; let-7f-5p; 15a-5p; 34a-5p; 330-5p; 7-5p; 665; 576-5p; 15b-5p; 103a-3p; let-7a-3p; 151a-3p; 184; 409-3p; 320a-3p; 130a-3p; 27b-3p; 423-5p; 374b-5p; 181b-5p; 6724-5p; 584-5p; 23a-3p; 92b-3p; 93-3p; 17-5p; 484; 192-5p; 421; 497-5p; 154-3p; 26a-5p; 328-3p; 708-5p; 503-5p; 195-5p; 941; 501-5p; 23b-3p; 139-5p; 877-5p; 625-3p; 127-5p; 625-5p; 6511a-3p; 26b-5p; 432-5p; let-7b-5p; 206; 24-3p; 130b-5p; 671-5p; 454-3p; 329-3p; 889-3p; 145-5p; 125a-5p; 376b-3p; 140-5p; 30d-5p; 296-5p; 193a-5p; 491-5p; 34c-5p; 9-5p; 301a-3p; 129-5p; 628-5p; 376c-3p; 1307-3p; 769-5p; 204-5p; 296-3p; 500a-5p; 378a-3p; 1185-5p; 543 |
| Longevity regulating pathway | 2.69E-06 | 95 | 119 | 27a-5p; 107; 342-3p; 590-3p; 378a-5p; 1185-1-3p; 30a-3p; let-7e-5p; 362-5p; 320c; 320d; 92a-3p; let-7d-5p; 542-3p; 100-3p; 374a-5p; 31-5p; 132-5p; let-7a-5p; 197-3p; 320b; 22-3p; 25-3p; 149-5p; 181a-2-3p; 128-3p; 126-3p; 99a-5p; 323b-3p; 205-5p; 1-3p; 323a-3p; 374a-3p; 30e-5p; 130b-3p; 30c-5p; 485-3p; 98-5p; let-7f-5p; 15a-5p; 34a-5p; 330-5p; 7-5p; 665; 576-5p; 15b-5p; 103a-3p; let-7a-3p; 191-5p; 184; 409-3p; 320a-3p; 130a-3p; 27b-3p; 423-5p; 374b-5p; 181b-5p; 584-5p; 23a-3p; 92b-3p; 93-3p; 17-5p; 484; 192-5p; 421; 497-5p; 154-3p; 26a-5p; 708-5p; 503-5p; 675-3p; 195-5p; 941; 501-5p; 23b-3p; 133a-3p; 139-5p; 877-5p; 625-3p; 127-5p; 625-5p; 15b-3p; 299-5p; 3613-5p; 26b-5p; 432-5p; let-7b-5p; 206; 24-3p; 130b-5p; 3925-5p; 337-3p; 671-5p; 190a-5p; 454-3p; 329-3p; 99b-5p; 145-5p; 125a-5p; 377-3p; 140-5p; 30d-5p; 193a-5p; 491-5p; 34c-5p; 9-5p; 301a-3p; 129-5p; 628-5p; 376c-3p; 769-5p; 1271-5p; 204-5p; 1287-5p; 296-3p; 125b-1-3p; 500a-5p; 378a-3p; 543 |
| Alcoholism | 1.82E-04 | 100 | 119 | 133a-5p; 107; 483-5p; 342-3p; 590-3p; 378a-5p; 1185-1-3p; 30a-3p; let-7e-5p; 320c; 320d; 92a-3p; let-7d-5p; 542-3p; 100-3p; 374a-5p; 31-5p; let-7a-5p; 197-3p; 320b; 22-3p; 486-3p; 25-3p; 149-5p; 181a-2-3p; 128-3p; 126-3p; 99a-5p; 323b-3p; 205-5p; 629-5p; 1-3p; 323a-3p; 374a-3p; 30e-5p; 130b-3p; 30c-5p; 98-5p; let-7f-5p; 15a-5p; 34a-5p; 330-5p; 7-5p; 411-5p; 665; 576-5p; 15b-5p; 103a-3p; let-7a-3p; 191-5p; 127-3p; 320a-3p; 382-5p; 130a-3p; 27b-3p; 423-5p; 374b-5p; 181b-5p; 584-5p; 23a-3p; 22-5p; 92b-3p; 93-3p; 17-5p; 484; 192-5p; 421; 497-5p; 154-3p; 26a-5p; 328-3p; 195-5p; 501-5p; 23b-3p; 133a-3p; 139-5p; 877-5p; 625-5p; 299-5p; 3613-5p; 26b-5p; 432-5p; let-7b-5p; 206; 24-3p; 130b-5p; 598-3p; 3925-5p; 671-5p; 190a-5p; 454-3p; 329-3p; 889-3p; 99b-5p; 145-5p; 125a-5p; 377-3p; 99b-3p; 376b-3p; 140-5p; 493-3p; 30d-5p; 296-5p; 491-5p; 34c-5p; 9-5p; 301a-3p; 129-5p; 628-5p; 376c-3p; 1307-3p; 769-5p; 204-5p; 1287-5p; 296-3p; 500a-5p; 378a-3p; 1185-5p; 543 |
| Small cell lung cancer | 5.60E-04 | 102 | 119 | 27a-5p; 107; 483-5p; 342-3p; 590-3p; 378a-5p; 1185-1-3p; 30a-3p; let-7e-5p; 362-5p; 320c; 320d; 92a-3p; let-7d-5p; 542-3p; 100-3p; 374a-5p; 31-5p; let-7a-5p; 197-3p; 320b; 22-3p; 486-3p; 25-3p; 149-5p; 181a-2-3p; 128-3p; 126-3p; 99a-5p; 323b-3p; 205-5p; 1306-5p; 1-3p; 323a-3p; 379-5p; 374a-3p; 30e-5p; 130b-3p; 574-3p; 30c-5p; 98-5p; let-7f-5p; 15a-5p; 34a-5p; 330-5p; 7-5p; 665; 576-5p; 15b-5p; 103a-3p; let-7a-3p; 191-5p; 151a-3p; 7706; 184; 409-3p; 320a-3p; 382-5p; 130a-3p; 423-5p; 374b-5p; 181b-5p; 584-5p; 23a-3p; 92b-3p; 136-3p; 93-3p; 17-5p; 484; 192-5p; 421; 497-5p; 26a-5p; 708-5p; 503-5p; 195-5p; 941; 23b-3p; 7704; 133a-3p; 139-5p; 877-5p; 625-3p; 625-5p; 15b-3p; 299-5p; 3613-5p; 26b-5p; let-7b-5p; 206; 24-3p; 130b-5p; 3925-5p; 454-3p; 329-3p; 889-3p; 145-5p; 125a-5p; 377-3p; 376b-3p; 140-5p; 30d-5p; 193a-5p; 491-5p; 34c-5p; 9-5p; 301a-3p; 129-5p; 376c-3p; 1307-3p; 769-5p; 1271-5p; 204-5p; 1287-5p; 296-3p; 125b-1-3p; 378a-3p; 1185-5p; 543 |
| Thermogenesis | 1.39E-02 | 108 | 119 | 107; 483-5p; 342-3p; 590-3p; 378a-5p; 1185-1-3p; 30a-3p; let-7e-5p; 362-5p; 320c; 320d; 92a-3p; let-7d-5p; 542-3p; 374a-5p; 31-5p; let-7a-5p; 197-3p; 320b; 22-3p; 25-3p; 149-5p; 181a-2-3p; 128-3p; 126-3p; 99a-5p; 323b-3p; 205-5p; 1306-5p; 629-5p; 1-3p; 379-5p; 30e-5p; 130b-3p; 30c-5p; 485-3p; 98-5p; let-7f-5p; 15a-5p; 34a-5p; 330-5p; 7-5p; 411-5p; 665; 576-5p; 15b-5p; 103a-3p; let-7a-3p; 191-5p; 409-3p; 320a-3p; let-7i-3p; 130a-3p; 27b-3p; 423-5p; 374b-5p; 628-3p; 181b-5p; 23a-3p; 92b-3p; 93-3p; 17-5p; 484; 192-5p; 421; 497-5p; 154-3p; 26a-5p; 328-3p; 708-5p; 503-5p; 675-3p; 195-5p; 501-5p; 23b-3p; 133a-3p; 139-5p; 877-5p; 127-5p; 664a-5p; 3613-5p; 26b-5p; 432-5p; let-7b-5p; 206; 24-3p; 130b-5p; 3925-5p; 671-5p; 454-3p; 329-3p; 889-3p; 99b-5p; 145-5p; 125a-5p; 377-3p; 99b-3p; 376b-3p; 140-5p; 493-3p; 30d-5p; 296-5p; 193a-5p; 491-5p; 9-5p; 301a-3p; 129-5p; 628-5p; 376c-3p; 1307-3p; 769-5p; 204-5p; 1287-5p; 296-3p; 125b-1-3p; 500a-5p; 378a-3p; 1185-5p; 543 |
| AGE-RAGE signaling pathway in diabetic complications | 8.85E-05 | 98 | 118 | 27a-5p; 107; 483-5p; 342-3p; 590-3p; 378a-5p; 1185-1-3p; 30a-3p; let-7e-5p; 92a-3p; let-7d-5p; 542-3p; 100-3p; 374a-5p; 31-5p; 132-5p; let-7a-5p; 197-3p; 320b; 22-3p; 486-3p; 25-3p; 149-5p; 181a-2-3p; 128-3p; 126-3p; 99a-5p; 323b-3p; 205-5p; 1306-5p; 1-3p; 323a-3p; 379-5p; 374a-3p; 30e-5p; 130b-3p; 574-3p; 30c-5p; 98-5p; let-7f-5p; 15a-5p; 34a-5p; 330-5p; 7-5p; 665; 576-5p; 15b-5p; 103a-3p; let-7a-3p; 191-5p; 7706; 184; 409-3p; 320a-3p; 382-5p; 130a-3p; 27b-3p; 423-5p; 374b-5p; 181b-5p; 584-5p; 23a-3p; 22-5p; 92b-3p; 93-3p; 17-5p; 484; 192-5p; 421; 497-5p; 154-3p; 26a-5p; 328-3p; 708-5p; 503-5p; 195-5p; 941; 501-5p; 23b-3p; 133a-3p; 139-5p; 877-5p; 625-3p; 127-5p; 625-5p; 26b-5p; let-7b-5p; 206; 24-3p; 130b-5p; 3925-5p; 337-3p; 671-5p; 454-3p; 329-3p; 99b-5p; 145-5p; 125a-5p; 377-3p; 140-5p; 30d-5p; 296-5p; 193a-5p; 491-5p; 34c-5p; 9-5p; 301a-3p; 129-5p; 376c-3p; 1307-3p; 769-5p; 1271-5p; 204-5p; 296-3p; 500a-5p; 378a-3p; 1185-5p; 543 |
| Chronic myeloid leukemia | 1.13E-03 | 102 | 118 | 27a-5p; 107; 483-5p; 342-3p; 590-3p; 378a-5p; 1185-1-3p; 30a-3p; let-7e-5p; 320c; 320d; 92a-3p; let-7d-5p; 542-3p; 100-3p; 374a-5p; 31-5p; let-7a-5p; 197-3p; 320b; 22-3p; 486-3p; 25-3p; 149-5p; 128-3p; 126-3p; 99a-5p; 323b-3p; 205-5p; 1306-5p; 1-3p; 323a-3p; 379-5p; 374a-3p; 30e-5p; 130b-3p; 574-3p; 30c-5p; 98-5p; let-7f-5p; 15a-5p; 34a-5p; 330-5p; 7-5p; 411-5p; 665; 576-5p; 15b-5p; 103a-3p; let-7a-3p; 191-5p; 151a-3p; 7706; 184; 409-3p; 320a-3p; 130a-3p; 27b-3p; 423-5p; 374b-5p; 181b-5p; 584-5p; 23a-3p; 92b-3p; 93-3p; 17-5p; 484; 192-5p; 421; 497-5p; 154-3p; 26a-5p; 708-5p; 503-5p; 195-5p; 23b-3p; 7704; 133a-3p; 139-5p; 877-5p; 625-3p; 127-5p; 625-5p; 299-5p; 3613-5p; 26b-5p; 432-5p; let-7b-5p; 206; 24-3p; 130b-5p; 3925-5p; 671-5p; 454-3p; 329-3p; 889-3p; 145-5p; 125a-5p; 377-3p; 376b-3p; 140-5p; 30d-5p; 193a-5p; 491-5p; 34c-5p; 9-5p; 301a-3p; 129-5p; 376c-3p; 1307-3p; 769-5p; 1271-5p; 204-5p; 296-3p; 125b-1-3p; 378a-3p; 1185-5p; 543 |
| Cytokine-cytokine receptor interaction | 3.08E-03 | 102 | 117 | 107; 483-5p; 342-3p; 590-3p; 378a-5p; 1185-1-3p; 30a-3p; let-7e-5p; 362-5p; 92a-3p; let-7d-5p; 542-3p; 100-3p; 374a-5p; 31-5p; let-7a-5p; 197-3p; 22-3p; 486-3p; 25-3p; 149-5p; 181a-2-3p; 128-3p; 126-3p; 205-5p; 1306-5p; 629-5p; 1-3p; 323a-3p; 379-5p; 374a-3p; 30e-5p; 130b-3p; 574-3p; 30c-5p; 485-3p; 98-5p; let-7f-5p; 15a-5p; 34a-5p; 330-5p; 7-5p; 411-5p; 665; 576-5p; 15b-5p; 103a-3p; let-7a-3p; 191-5p; 151a-3p; 184; 409-3p; 320a-3p; 130a-3p; 27b-3p; 423-5p; 374b-5p; 628-3p; 181b-5p; 6724-5p; 584-5p; 23a-3p; 92b-3p; 93-3p; 17-5p; 484; 192-5p; 497-5p; 26a-5p; 708-5p; 503-5p; 675-3p; 195-5p; 501-5p; 23b-3p; 7704; 133a-3p; 139-5p; 877-5p; 127-5p; 625-5p; 15b-3p; 299-5p; 3613-5p; 6511a-3p; 26b-5p; 432-5p; let-7b-5p; 206; 24-3p; 130b-5p; 3925-5p; 337-3p; 671-5p; 190a-5p; 454-3p; 329-3p; 145-5p; 125a-5p; 377-3p; 376b-3p; 140-5p; 493-3p; 30d-5p; 296-5p; 34c-5p; 9-5p; 301a-3p; 129-5p; 376c-3p; 1307-3p; 769-5p; 1271-5p; 204-5p; 296-3p; 125b-1-3p; 378a-3p |
| p53 signaling pathway | 1.51E-02 | 106 | 117 | 107; 342-3p; 590-3p; 378a-5p; 1185-1-3p; 30a-3p; let-7e-5p; 362-5p; 320c; 320d; 92a-3p; let-7d-5p; 542-3p; 374a-5p; 31-5p; let-7a-5p; 197-3p; 320b; 22-3p; 486-3p; 25-3p; 149-5p; 128-3p; 126-3p; 323b-3p; 205-5p; 1306-5p; 629-5p; 1-3p; 323a-3p; 379-5p; 374a-3p; 30e-5p; 130b-3p; 30c-5p; 98-5p; let-7f-5p; 15a-5p; 34a-5p; 330-5p; 7-5p; 665; 576-5p; 15b-5p; 103a-3p; let-7a-3p; 191-5p; 151a-3p; 7706; 184; 409-3p; 320a-3p; 382-5p; 130a-3p; 27b-3p; 423-5p; 374b-5p; 181b-5p; 584-5p; 23a-3p; 22-5p; 92b-3p; 93-3p; 17-5p; 484; 192-5p; 421; 497-5p; 26a-5p; 708-5p; 503-5p; 675-3p; 195-5p; 501-5p; 23b-3p; 7704; 133a-3p; 139-5p; 877-5p; 127-5p; 625-5p; 664a-5p; 15b-3p; 299-5p; 3613-5p; 26b-5p; 432-5p; let-7b-5p; 206; 24-3p; 130b-5p; 337-3p; 190a-5p; 454-3p; 329-3p; 99b-5p; 145-5p; 125a-5p; 377-3p; 376b-3p; 30d-5p; 296-5p; 193a-5p; 491-5p; 34c-5p; 9-5p; 301a-3p; 129-5p; 376c-3p; 1307-3p; 1271-5p; 204-5p; 1287-5p; 296-3p; 125b-1-3p; 378a-3p; 543 |
| Central carbon metabolism in cancer | 1.08E-06 | 90 | 116 | 27a-5p; 133a-5p; 107; 483-5p; 590-3p; 378a-5p; 30a-3p; let-7e-5p; 320c; 92a-3p; let-7d-5p; 542-3p; 374a-5p; 31-5p; 132-5p; let-7a-5p; 197-3p; 320b; 22-3p; 486-3p; 25-3p; 149-5p; 181a-2-3p; 128-3p; 126-3p; 99a-5p; 205-5p; 1306-5p; 1-3p; 323a-3p; 374a-3p; 30e-5p; 130b-3p; 574-3p; 30c-5p; 485-3p; 98-5p; let-7f-5p; 15a-5p; 34a-5p; 330-5p; 7-5p; 665; 576-5p; 15b-5p; 103a-3p; 191-5p; 151a-3p; 184; 409-3p; 320a-3p; 382-5p; 130a-3p; 27b-3p; 423-5p; 374b-5p; 181b-5p; 6724-5p; 584-5p; 23a-3p; 22-5p; 92b-3p; 93-3p; 17-5p; 484; 192-5p; 421; 497-5p; 154-3p; 26a-5p; 328-3p; 708-5p; 503-5p; 195-5p; 23b-3p; 133a-3p; 139-5p; 877-5p; 625-3p; 625-5p; 299-5p; 26b-5p; let-7b-5p; 206; 24-3p; 130b-5p; 598-3p; 3925-5p; 671-5p; 190a-5p; 454-3p; 329-3p; 889-3p; 99b-5p; 145-5p; 125a-5p; 377-3p; 140-5p; 493-3p; 30d-5p; 296-5p; 193a-5p; 491-5p; 34c-5p; 9-5p; 301a-3p; 129-5p; 628-5p; 1307-3p; 769-5p; 204-5p; 1287-5p; 296-3p; 125b-1-3p; 378a-3p; 543 |
| TNF signaling pathway | 1.23E-06 | 90 | 116 | 27a-5p; 133a-5p; 107; 483-5p; 342-3p; 590-3p; 378a-5p; 1185-1-3p; let-7e-5p; 362-5p; 320c; 92a-3p; let-7d-5p; 542-3p; 100-3p; 374a-5p; 31-5p; let-7a-5p; 197-3p; 320b; 22-3p; 486-3p; 25-3p; 149-5p; 181a-2-3p; 128-3p; 126-3p; 99a-5p; 323b-3p; 205-5p; 629-5p; 1-3p; 379-5p; 374a-3p; 30e-5p; 130b-3p; 30c-5p; 485-3p; 98-5p; let-7f-5p; 15a-5p; 34a-5p; 330-5p; 7-5p; 411-5p; 665; 576-5p; 15b-5p; 103a-3p; let-7a-3p; 191-5p; 151a-3p; 184; 409-3p; 320a-3p; 382-5p; 130a-3p; 27b-3p; 423-5p; 374b-5p; 181b-5p; 584-5p; 23a-3p; 22-5p; 92b-3p; 136-3p; 93-3p; 17-5p; 484; 192-5p; 421; 497-5p; 154-3p; 26a-5p; 708-5p; 503-5p; 195-5p; 23b-3p; 133a-3p; 139-5p; 877-5p; 625-3p; 127-5p; 625-5p; 664a-5p; 6511a-3p; 26b-5p; 432-5p; let-7b-5p; 206; 24-3p; 130b-5p; 3925-5p; 454-3p; 329-3p; 889-3p; 145-5p; 125a-5p; 376b-3p; 493-3p; 30d-5p; 296-5p; 193a-5p; 491-5p; 34c-5p; 9-5p; 301a-3p; 129-5p; 376c-3p; 1307-3p; 769-5p; 204-5p; 296-3p; 125b-1-3p; 378a-3p; 543 |
| Insulin resistance | 7.53E-05 | 96 | 116 | 27a-5p; 107; 483-5p; 342-3p; 590-3p; 378a-5p; 1185-1-3p; let-7e-5p; 362-5p; 320c; 320d; 92a-3p; 542-3p; 100-3p; 374a-5p; 31-5p; 132-5p; let-7a-5p; 197-3p; 320b; 22-3p; 486-3p; 25-3p; 149-5p; 128-3p; 126-3p; 99a-5p; 323b-3p; 205-5p; 1-3p; 323a-3p; 374a-3p; 30e-5p; 130b-3p; 30c-5p; 485-3p; 98-5p; let-7f-5p; 15a-5p; 34a-5p; 330-5p; 7-5p; 665; 576-5p; 15b-5p; 103a-3p; let-7a-3p; 191-5p; 151a-3p; 184; 409-3p; 320a-3p; 382-5p; 130a-3p; 27b-3p; 423-5p; 374b-5p; 181b-5p; 23a-3p; 92b-3p; 136-3p; 93-3p; 17-5p; 484; 192-5p; 421; 497-5p; 154-3p; 26a-5p; 708-5p; 503-5p; 675-3p; 195-5p; 941; 501-5p; 23b-3p; 139-5p; 877-5p; 625-3p; 625-5p; 15b-3p; 3613-5p; 6511a-3p; 26b-5p; 432-5p; let-7b-5p; 206; 24-3p; 130b-5p; 3925-5p; 337-3p; 671-5p; 454-3p; 329-3p; 99b-5p; 145-5p; 125a-5p; 377-3p; 99b-3p; 493-3p; 30d-5p; 193a-5p; 9-5p; 301a-3p; 129-5p; 628-5p; 376c-3p; 1307-3p; 769-5p; 1271-5p; 204-5p; 1287-5p; 296-3p; 500a-5p; 378a-3p; 543 |
| Growth hormone synthesis, secretion and action | 2.69E-04 | 97 | 116 | 27a-5p; 107; 483-5p; 342-3p; 590-3p; 378a-5p; 1185-1-3p; 30a-3p; let-7e-5p; 320c; 320d; 92a-3p; let-7d-5p; 542-3p; 374a-5p; let-7a-5p; 197-3p; 320b; 22-3p; 486-3p; 25-3p; 149-5p; 181a-2-3p; 128-3p; 126-3p; 99a-5p; 323b-3p; 205-5p; 1306-5p; 1-3p; 323a-3p; 379-5p; 374a-3p; 30e-5p; 130b-3p; 574-3p; 30c-5p; 98-5p; let-7f-5p; 15a-5p; 34a-5p; 330-5p; 7-5p; 411-5p; 665; 576-5p; 15b-5p; 103a-3p; let-7a-3p; 191-5p; 184; 409-3p; 320a-3p; 130a-3p; 27b-3p; 423-5p; 374b-5p; 628-3p; 181b-5p; 584-5p; 23a-3p; 92b-3p; 93-3p; 17-5p; 484; 192-5p; 421; 497-5p; 154-3p; 26a-5p; 708-5p; 503-5p; 195-5p; 501-5p; 23b-3p; 133a-3p; 139-5p; 877-5p; 625-3p; 127-5p; 625-5p; 26b-5p; 432-5p; let-7b-5p; 206; 24-3p; 130b-5p; 3925-5p; 337-3p; 671-5p; 190a-5p; 454-3p; 329-3p; 889-3p; 99b-5p; 145-5p; 125a-5p; 377-3p; 99b-3p; 376b-3p; 140-5p; 30d-5p; 193a-5p; 34c-5p; 9-5p; 301a-3p; 129-5p; 628-5p; 376c-3p; 1307-3p; 769-5p; 204-5p; 296-3p; 500a-5p; 378a-3p; 543 |
| Dopaminergic synapse | 4.06E-06 | 91 | 115 | 27a-5p; 107; 342-3p; 590-3p; 1185-1-3p; 30a-3p; let-7e-5p; 320c; 320d; 92a-3p; let-7d-5p; 542-3p; 100-3p; 374a-5p; 31-5p; let-7a-5p; 197-3p; 320b; 22-3p; 486-3p; 25-3p; 149-5p; 181a-2-3p; 128-3p; 126-3p; 99a-5p; 323b-3p; 205-5p; 1-3p; 323a-3p; 374a-3p; 30e-5p; 130b-3p; 30c-5p; 98-5p; let-7f-5p; 15a-5p; 34a-5p; 330-5p; 7-5p; 411-5p; 665; 576-5p; 15b-5p; 103a-3p; let-7a-3p; 151a-3p; 184; 409-3p; 320a-3p; 382-5p; 130a-3p; 27b-3p; 423-5p; 374b-5p; 181b-5p; 584-5p; 23a-3p; 92b-3p; 93-3p; 17-5p; 484; 192-5p; 421; 497-5p; 26a-5p; 708-5p; 503-5p; 195-5p; 941; 501-5p; 23b-3p; 133a-3p; 139-5p; 877-5p; 625-3p; 625-5p; 664a-5p; 15b-3p; 26b-5p; 432-5p; let-7b-5p; 206; 24-3p; 130b-5p; 598-3p; 3925-5p; 671-5p; 190a-5p; 454-3p; 329-3p; 889-3p; 99b-5p; 145-5p; 125a-5p; 377-3p; 99b-3p; 376b-3p; 493-3p; 30d-5p; 296-5p; 491-5p; 34c-5p; 9-5p; 301a-3p; 129-5p; 628-5p; 1307-3p; 769-5p; 204-5p; 1287-5p; 500a-5p; 378a-3p; 1185-5p; 543 |
| TGF-beta signaling pathway | 1.09E-05 | 92 | 115 | 27a-5p; 133a-5p; 107; 483-5p; 342-3p; 590-3p; 378a-5p; 1185-1-3p; 30a-3p; let-7e-5p; 362-5p; 320c; 92a-3p; let-7d-5p; 542-3p; 100-3p; 374a-5p; 31-5p; 132-5p; let-7a-5p; 197-3p; 320b; 22-3p; 486-3p; 25-3p; 149-5p; 128-3p; 126-3p; 205-5p; 629-5p; 1-3p; 323a-3p; 379-5p; 374a-3p; 30e-5p; 130b-3p; 574-3p; 30c-5p; 98-5p; let-7f-5p; 15a-5p; 34a-5p; 330-5p; 7-5p; 411-5p; 665; 576-5p; 15b-5p; 103a-3p; let-7a-3p; 127-3p; 184; 409-3p; 320a-3p; 382-5p; 130a-3p; 27b-3p; 423-5p; 374b-5p; 628-3p; 181b-5p; 584-5p; 23a-3p; 92b-3p; 93-3p; 17-5p; 484; 192-5p; 421; 497-5p; 154-3p; 26a-5p; 708-5p; 503-5p; 675-3p; 195-5p; 941; 501-5p; 23b-3p; 133a-3p; 877-5p; 625-5p; 26b-5p; 432-5p; let-7b-5p; 206; 24-3p; 130b-5p; 3925-5p; 337-3p; 671-5p; 454-3p; 329-3p; 889-3p; 99b-5p; 145-5p; 125a-5p; 377-3p; 140-5p; 493-3p; 30d-5p; 296-5p; 491-5p; 34c-5p; 9-5p; 301a-3p; 129-5p; 376c-3p; 1307-3p; 769-5p; 204-5p; 125b-1-3p; 500a-5p; 378a-3p; 543 |
| Non-alcoholic fatty liver disease NAFLD | 1.38E-05 | 92 | 115 | 27a-5p; 107; 483-5p; 342-3p; 590-3p; 378a-5p; 1185-1-3p; 30a-3p; let-7e-5p; 362-5p; 320c; 92a-3p; let-7d-5p; 542-3p; 100-3p; 374a-5p; let-7a-5p; 197-3p; 320b; 22-3p; 486-3p; 25-3p; 149-5p; 128-3p; 126-3p; 99a-5p; 205-5p; 1306-5p; 1-3p; 379-5p; 374a-3p; 30e-5p; 130b-3p; 574-3p; 30c-5p; 485-3p; 98-5p; let-7f-5p; 15a-5p; 34a-5p; 330-5p; 7-5p; 411-5p; 665; 576-5p; 15b-5p; 103a-3p; let-7a-3p; 191-5p; 127-3p; 151a-3p; 184; 409-3p; 320a-3p; 382-5p; 130a-3p; 27b-3p; 423-5p; 374b-5p; 628-3p; 181b-5p; 23a-3p; 92b-3p; 93-3p; 17-5p; 484; 192-5p; 421; 497-5p; 154-3p; 26a-5p; 328-3p; 708-5p; 503-5p; 675-3p; 195-5p; 501-5p; 23b-3p; 133a-3p; 139-5p; 877-5p; 625-3p; 127-5p; 3613-5p; 26b-5p; 432-5p; let-7b-5p; 206; 24-3p; 130b-5p; 671-5p; 454-3p; 329-3p; 145-5p; 125a-5p; 377-3p; 99b-3p; 376b-3p; 140-5p; 30d-5p; 296-5p; 193a-5p; 491-5p; 34c-5p; 9-5p; 301a-3p; 129-5p; 628-5p; 1307-3p; 769-5p; 204-5p; 296-3p; 500a-5p; 378a-3p; 543 |
| Non-small cell lung cancer | 4.10E-04 | 97 | 115 | 27a-5p; 133a-5p; 107; 483-5p; 342-3p; 378a-5p; 1185-1-3p; 30a-3p; let-7e-5p; 92a-3p; let-7d-5p; 542-3p; 100-3p; 374a-5p; 31-5p; let-7a-5p; 197-3p; 320b; 22-3p; 486-3p; 25-3p; 149-5p; 128-3p; 126-3p; 99a-5p; 323b-3p; 205-5p; 1-3p; 323a-3p; 374a-3p; 30e-5p; 130b-3p; 574-3p; 30c-5p; 98-5p; let-7f-5p; 15a-5p; 34a-5p; 330-5p; 7-5p; 411-5p; 665; 576-5p; 15b-5p; 103a-3p; let-7a-3p; 191-5p; 7706; 184; 409-3p; 320a-3p; 130a-3p; 27b-3p; 423-5p; 374b-5p; 181b-5p; 584-5p; 23a-3p; 22-5p; 92b-3p; 93-3p; 17-5p; 484; 192-5p; 421; 497-5p; 154-3p; 26a-5p; 708-5p; 503-5p; 195-5p; 501-5p; 23b-3p; 7704; 133a-3p; 139-5p; 877-5p; 625-3p; 625-5p; 664a-5p; 299-5p; 3613-5p; 26b-5p; let-7b-5p; 206; 24-3p; 130b-5p; 3925-5p; 337-3p; 671-5p; 454-3p; 329-3p; 889-3p; 145-5p; 125a-5p; 377-3p; 376b-3p; 140-5p; 30d-5p; 193a-5p; 491-5p; 34c-5p; 9-5p; 301a-3p; 129-5p; 376c-3p; 1307-3p; 769-5p; 1271-5p; 204-5p; 296-3p; 125b-1-3p; 500a-5p; 378a-3p; 543 |
| Toxoplasmosis | 6.07E-04 | 98 | 115 | 27a-5p; 107; 483-5p; 342-3p; 590-3p; 378a-5p; 1185-1-3p; 30a-3p; let-7e-5p; 362-5p; 320c; 320d; 92a-3p; let-7d-5p; 542-3p; 374a-5p; let-7a-5p; 197-3p; 320b; 22-3p; 25-3p; 149-5p; 181a-2-3p; 128-3p; 126-3p; 99a-5p; 205-5p; 1306-5p; 1-3p; 323a-3p; 374a-3p; 30e-5p; 130b-3p; 574-3p; 30c-5p; 98-5p; let-7f-5p; 15a-5p; 34a-5p; 330-5p; 7-5p; 411-5p; 665; 576-5p; 15b-5p; 103a-3p; let-7a-3p; 127-3p; 151a-3p; 184; 409-3p; 320a-3p; 382-5p; 130a-3p; 27b-3p; 423-5p; 374b-5p; 181b-5p; 584-5p; 23a-3p; 22-5p; 92b-3p; 93-3p; 17-5p; 484; 192-5p; 421; 497-5p; 154-3p; 26a-5p; 328-3p; 708-5p; 503-5p; 195-5p; 941; 501-5p; 23b-3p; 7704; 133a-3p; 139-5p; 877-5p; 625-3p; 625-5p; 15b-3p; 299-5p; 6511a-3p; 26b-5p; let-7b-5p; 206; 24-3p; 130b-5p; 337-3p; 454-3p; 329-3p; 889-3p; 145-5p; 125a-5p; 377-3p; 376b-3p; 140-5p; 30d-5p; 296-5p; 491-5p; 34c-5p; 9-5p; 301a-3p; 129-5p; 628-5p; 376c-3p; 1307-3p; 769-5p; 204-5p; 296-3p; 500a-5p; 378a-3p |
| Oocyte meiosis | 2.21E-03 | 100 | 115 | 107; 483-5p; 342-3p; 590-3p; 378a-5p; 30a-3p; let-7e-5p; 362-5p; 320c; 320d; 92a-3p; let-7d-5p; 542-3p; 100-3p; 374a-5p; 31-5p; 132-5p; let-7a-5p; 197-3p; 320b; 22-3p; 486-3p; 25-3p; 149-5p; 181a-2-3p; 128-3p; 126-3p; 99a-5p; 205-5p; 1-3p; 323a-3p; 30e-5p; 130b-3p; 30c-5p; 485-3p; 98-5p; let-7f-5p; 15a-5p; 34a-5p; 330-5p; 7-5p; 411-5p; 665; 576-5p; 15b-5p; 103a-3p; 191-5p; 151a-3p; 409-3p; 320a-3p; 382-5p; 130a-3p; 27b-3p; 423-5p; 181b-5p; 584-5p; 23a-3p; 22-5p; 92b-3p; 93-3p; 17-5p; 484; 192-5p; 421; 497-5p; 154-3p; 26a-5p; 328-3p; 503-5p; 675-3p; 195-5p; 941; 23b-3p; 133a-3p; 139-5p; 877-5p; 625-5p; 15b-3p; 3613-5p; 26b-5p; 432-5p; let-7b-5p; 206; 24-3p; 130b-5p; 598-3p; 3925-5p; 671-5p; 190a-5p; 454-3p; 329-3p; 889-3p; 99b-5p; 145-5p; 125a-5p; 377-3p; 99b-3p; 376b-3p; 140-5p; 493-3p; 30d-5p; 296-5p; 193a-5p; 491-5p; 34c-5p; 9-5p; 301a-3p; 129-5p; 376c-3p; 1307-3p; 769-5p; 1271-5p; 204-5p; 125b-1-3p; 378a-3p |
| Endometrial cancer | 1.59E-05 | 91 | 114 | 27a-5p; 133a-5p; 107; 483-5p; 590-3p; 378a-5p; 1185-1-3p; 30a-3p; let-7e-5p; 320c; 92a-3p; let-7d-5p; 542-3p; 100-3p; 374a-5p; 31-5p; let-7a-5p; 197-3p; 320b; 22-3p; 486-3p; 25-3p; 149-5p; 128-3p; 126-3p; 99a-5p; 323b-3p; 205-5p; 1-3p; 323a-3p; 374a-3p; 30e-5p; 130b-3p; 574-3p; 30c-5p; 485-3p; 98-5p; let-7f-5p; 15a-5p; 34a-5p; 330-5p; 7-5p; 411-5p; 665; 576-5p; 15b-5p; 103a-3p; let-7a-3p; 191-5p; 7706; 184; 409-3p; 320a-3p; 382-5p; 130a-3p; 27b-3p; 423-5p; 374b-5p; 181b-5p; 584-5p; 23a-3p; 22-5p; 92b-3p; 93-3p; 17-5p; 484; 192-5p; 421; 497-5p; 154-3p; 26a-5p; 708-5p; 503-5p; 195-5p; 501-5p; 23b-3p; 7704; 133a-3p; 139-5p; 877-5p; 625-3p; 625-5p; 299-5p; 26b-5p; let-7b-5p; 206; 24-3p; 130b-5p; 3925-5p; 671-5p; 454-3p; 329-3p; 889-3p; 145-5p; 125a-5p; 377-3p; 99b-3p; 30d-5p; 193a-5p; 491-5p; 34c-5p; 9-5p; 301a-3p; 129-5p; 376c-3p; 1307-3p; 769-5p; 1271-5p; 204-5p; 1287-5p; 296-3p; 125b-1-3p; 378a-3p; 543 |
| C-type lectin receptor signaling pathway | 2.54E-05 | 92 | 114 | 27a-5p; 133a-5p; 107; 483-5p; 342-3p; 590-3p; 378a-5p; 1185-1-3p; 30a-3p; let-7e-5p; 362-5p; 320c; 92a-3p; let-7d-5p; 542-3p; 374a-5p; 31-5p; let-7a-5p; 197-3p; 320b; 22-3p; 486-3p; 25-3p; 149-5p; 181a-2-3p; 128-3p; 126-3p; 99a-5p; 205-5p; 1306-5p; 1-3p; 374a-3p; 30e-5p; 130b-3p; 30c-5p; 98-5p; let-7f-5p; 15a-5p; 34a-5p; 330-5p; 7-5p; 665; 15b-5p; 103a-3p; let-7a-3p; 151a-3p; 184; 409-3p; 320a-3p; 382-5p; 130a-3p; 27b-3p; 423-5p; 374b-5p; 181b-5p; 584-5p; 23a-3p; 92b-3p; 17-5p; 484; 192-5p; 421; 497-5p; 154-3p; 26a-5p; 708-5p; 503-5p; 195-5p; 501-5p; 23b-3p; 133a-3p; 139-5p; 877-5p; 625-3p; 127-5p; 625-5p; 6511a-3p; 26b-5p; 432-5p; let-7b-5p; 206; 24-3p; 130b-5p; 598-3p; 3925-5p; 337-3p; 671-5p; 190a-5p; 454-3p; 329-3p; 145-5p; 125a-5p; 377-3p; 99b-3p; 376b-3p; 140-5p; 493-3p; 30d-5p; 296-5p; 193a-5p; 34c-5p; 9-5p; 301a-3p; 129-5p; 376c-3p; 1307-3p; 769-5p; 1271-5p; 204-5p; 296-3p; 500a-5p; 378a-3p; 1185-5p; 543 |
| Osteoclast differentiation | 3.76E-04 | 96 | 114 | 27a-5p; 107; 483-5p; 342-3p; 590-3p; 378a-5p; 1185-1-3p; 30a-3p; let-7e-5p; 362-5p; 92a-3p; let-7d-5p; 542-3p; 374a-5p; 31-5p; let-7a-5p; 197-3p; 320b; 22-3p; 486-3p; 25-3p; 149-5p; 181a-2-3p; 128-3p; 126-3p; 99a-5p; 323b-3p; 205-5p; 1306-5p; 1-3p; 379-5p; 374a-3p; 30e-5p; 130b-3p; 574-3p; 30c-5p; 98-5p; let-7f-5p; 15a-5p; 34a-5p; 330-5p; 7-5p; 411-5p; 665; 576-5p; 15b-5p; 103a-3p; let-7a-3p; 191-5p; 151a-3p; 184; 409-3p; 320a-3p; 130a-3p; 27b-3p; 423-5p; 374b-5p; 181b-5p; 6724-5p; 584-5p; 23a-3p; 92b-3p; 93-3p; 17-5p; 484; 192-5p; 421; 497-5p; 154-3p; 26a-5p; 708-5p; 503-5p; 195-5p; 501-5p; 23b-3p; 7704; 133a-3p; 139-5p; 877-5p; 625-3p; 625-5p; 6511a-3p; 26b-5p; let-7b-5p; 206; 24-3p; 130b-5p; 3925-5p; 454-3p; 329-3p; 889-3p; 145-5p; 125a-5p; 377-3p; 99b-3p; 140-5p; 493-3p; 30d-5p; 296-5p; 193a-5p; 491-5p; 34c-5p; 9-5p; 301a-3p; 129-5p; 376c-3p; 1307-3p; 769-5p; 204-5p; 296-3p; 125b-1-3p; 500a-5p; 378a-3p; 543 |
| Calcium signaling pathway | 6.77E-04 | 97 | 114 | 27a-5p; 133a-5p; 107; 483-5p; 342-3p; 590-3p; 378a-5p; 1185-1-3p; 30a-3p; let-7e-5p; 320c; 92a-3p; let-7d-5p; 542-3p; 100-3p; 374a-5p; 31-5p; let-7a-5p; 197-3p; 320b; 22-3p; 486-3p; 25-3p; 149-5p; 181a-2-3p; 128-3p; 126-3p; 99a-5p; 323b-3p; 205-5p; 1-3p; 323a-3p; 30e-5p; 130b-3p; 574-3p; 30c-5p; 98-5p; let-7f-5p; 15a-5p; 34a-5p; 330-5p; 7-5p; 411-5p; 665; 576-5p; 15b-5p; 103a-3p; let-7a-3p; 184; 409-3p; 320a-3p; 382-5p; 130a-3p; 27b-3p; 423-5p; 374b-5p; 181b-5p; 23a-3p; 22-5p; 92b-3p; 136-3p; 17-5p; 484; 192-5p; 421; 497-5p; 26a-5p; 328-3p; 503-5p; 675-3p; 195-5p; 941; 501-5p; 23b-3p; 7704; 133a-3p; 139-5p; 877-5p; 127-5p; 625-5p; 664a-5p; 15b-3p; 26b-5p; 432-5p; let-7b-5p; 206; 24-3p; 130b-5p; 598-3p; 3925-5p; 671-5p; 190a-5p; 454-3p; 329-3p; 145-5p; 125a-5p; 377-3p; 376b-3p; 140-5p; 493-3p; 30d-5p; 193a-5p; 491-5p; 34c-5p; 9-5p; 301a-3p; 129-5p; 628-5p; 1307-3p; 204-5p; 296-3p; 500a-5p; 1185-5p; 543 |
| Adrenergic signaling in cardiomyocytes | 1.87E-03 | 98 | 114 | 27a-5p; 107; 483-5p; 342-3p; 590-3p; 378a-5p; 1185-1-3p; let-7e-5p; 320c; 320d; 92a-3p; let-7d-5p; 542-3p; 100-3p; 374a-5p; 31-5p; let-7a-5p; 197-3p; 320b; 22-3p; 486-3p; 25-3p; 149-5p; 181a-2-3p; 128-3p; 126-3p; 99a-5p; 323b-3p; 205-5p; 1-3p; 323a-3p; 374a-3p; 30e-5p; 130b-3p; 30c-5p; 485-3p; 98-5p; let-7f-5p; 15a-5p; 34a-5p; 330-5p; 7-5p; 411-5p; 665; 576-5p; 15b-5p; 103a-3p; let-7a-3p; 127-3p; 184; 409-3p; 320a-3p; 382-5p; 130a-3p; 27b-3p; 423-5p; 374b-5p; 181b-5p; 584-5p; 23a-3p; 92b-3p; 136-3p; 93-3p; 17-5p; 484; 192-5p; 421; 497-5p; 154-3p; 26a-5p; 708-5p; 503-5p; 195-5p; 941; 501-5p; 23b-3p; 133a-3p; 139-5p; 877-5p; 625-3p; 127-5p; 625-5p; 299-5p; 26b-5p; 432-5p; let-7b-5p; 206; 24-3p; 130b-5p; 598-3p; 3925-5p; 671-5p; 190a-5p; 454-3p; 329-3p; 889-3p; 99b-5p; 145-5p; 125a-5p; 376b-3p; 493-3p; 30d-5p; 34c-5p; 9-5p; 301a-3p; 129-5p; 376c-3p; 1307-3p; 769-5p; 204-5p; 296-3p; 500a-5p; 378a-3p; 1185-5p |
| Sphingolipid signaling pathway | 6.59E-03 | 101 | 114 | 27a-5p; 133a-5p; 107; 483-5p; 342-3p; 590-3p; 378a-5p; 30a-3p; let-7e-5p; 362-5p; 320c; 320d; 92a-3p; let-7d-5p; 542-3p; 374a-5p; 31-5p; let-7a-5p; 197-3p; 320b; 22-3p; 486-3p; 25-3p; 149-5p; 181a-2-3p; 128-3p; 126-3p; 99a-5p; 205-5p; 1-3p; 374a-3p; 30e-5p; 130b-3p; 574-3p; 30c-5p; 98-5p; let-7f-5p; 15a-5p; 34a-5p; 330-5p; 7-5p; 411-5p; 665; 576-5p; 15b-5p; 103a-3p; let-7a-3p; 184; 409-3p; 320a-3p; 382-5p; 130a-3p; 27b-3p; 423-5p; 374b-5p; 181b-5p; 584-5p; 23a-3p; 92b-3p; 93-3p; 17-5p; 484; 192-5p; 421; 497-5p; 154-3p; 26a-5p; 708-5p; 503-5p; 195-5p; 941; 501-5p; 23b-3p; 133a-3p; 139-5p; 877-5p; 625-3p; 625-5p; 664a-5p; 299-5p; 26b-5p; 432-5p; let-7b-5p; 206; 24-3p; 130b-5p; 3925-5p; 671-5p; 190a-5p; 454-3p; 329-3p; 889-3p; 145-5p; 125a-5p; 377-3p; 30d-5p; 193a-5p; 491-5p; 34c-5p; 9-5p; 301a-3p; 129-5p; 376c-3p; 1307-3p; 769-5p; 1271-5p; 204-5p; 1287-5p; 296-3p; 125b-1-3p; 500a-5p; 378a-3p; 1185-5p; 543 |
| Huntington disease | 3.65E-02 | 104 | 114 | 107; 483-5p; 342-3p; 590-3p; 378a-5p; 1185-1-3p; 30a-3p; let-7e-5p; 362-5p; 320c; 92a-3p; let-7d-5p; 542-3p; 374a-5p; 31-5p; let-7a-5p; 197-3p; 320b; 22-3p; 486-3p; 25-3p; 149-5p; 128-3p; 126-3p; 99a-5p; 323b-3p; 205-5p; 1306-5p; 629-5p; 1-3p; 379-5p; 30e-5p; 130b-3p; 574-3p; 30c-5p; 485-3p; 98-5p; let-7f-5p; 15a-5p; 34a-5p; 7-5p; 411-5p; 665; 576-5p; 15b-5p; 103a-3p; let-7a-3p; 127-3p; 409-3p; 320a-3p; 382-5p; 130a-3p; 27b-3p; 423-5p; 374b-5p; 628-3p; 181b-5p; 584-5p; 23a-3p; 92b-3p; 93-3p; 17-5p; 484; 192-5p; 421; 497-5p; 26a-5p; 328-3p; 708-5p; 503-5p; 675-3p; 195-5p; 501-5p; 23b-3p; 7704; 133a-3p; 139-5p; 877-5p; 625-5p; 299-5p; 26b-5p; 432-5p; let-7b-5p; 206; 24-3p; 130b-5p; 337-3p; 671-5p; 454-3p; 329-3p; 889-3p; 99b-5p; 145-5p; 125a-5p; 377-3p; 99b-3p; 376b-3p; 140-5p; 30d-5p; 296-5p; 491-5p; 34c-5p; 9-5p; 301a-3p; 129-5p; 769-5p; 204-5p; 1287-5p; 296-3p; 125b-1-3p; 500a-5p; 378a-3p; 1185-5p; 543 |
| Parathyroid hormone synthesis, secretion and action | 4.08E-05 | 91 | 113 | 27a-5p; 133a-5p; 107; 483-5p; 342-3p; 590-3p; 378a-5p; 1185-1-3p; 30a-3p; let-7e-5p; 362-5p; 320c; 320d; 92a-3p; let-7d-5p; 542-3p; 374a-5p; 31-5p; let-7a-5p; 197-3p; 320b; 22-3p; 486-3p; 25-3p; 149-5p; 128-3p; 126-3p; 99a-5p; 323b-3p; 205-5p; 1306-5p; 1-3p; 30e-5p; 130b-3p; 574-3p; 30c-5p; 98-5p; let-7f-5p; 15a-5p; 34a-5p; 330-5p; 7-5p; 665; 576-5p; 15b-5p; 103a-3p; let-7a-3p; 191-5p; 127-3p; 184; 320a-3p; 130a-3p; 27b-3p; 423-5p; 374b-5p; 628-3p; 181b-5p; 584-5p; 23a-3p; 92b-3p; 93-3p; 17-5p; 484; 192-5p; 497-5p; 154-3p; 26a-5p; 328-3p; 708-5p; 503-5p; 195-5p; 501-5p; 23b-3p; 7704; 133a-3p; 139-5p; 877-5p; 625-5p; 664a-5p; 299-5p; 26b-5p; 432-5p; let-7b-5p; 206; 24-3p; 130b-5p; 671-5p; 454-3p; 329-3p; 889-3p; 99b-5p; 145-5p; 125a-5p; 377-3p; 99b-3p; 376b-3p; 140-5p; 30d-5p; 296-5p; 491-5p; 34c-5p; 9-5p; 301a-3p; 129-5p; 376c-3p; 1307-3p; 769-5p; 204-5p; 296-3p; 125b-1-3p; 500a-5p; 378a-3p; 543 |
| NOD-like receptor signaling pathway | 1.21E-02 | 101 | 113 | 27a-5p; 133a-5p; 107; 483-5p; 342-3p; 590-3p; 378a-5p; 1185-1-3p; 30a-3p; let-7e-5p; 362-5p; 320c; 92a-3p; let-7d-5p; 100-3p; 374a-5p; 31-5p; let-7a-5p; 197-3p; 320b; 486-3p; 25-3p; 149-5p; 181a-2-3p; 128-3p; 126-3p; 205-5p; 1306-5p; 629-5p; 1-3p; 323a-3p; 379-5p; 374a-3p; 30e-5p; 130b-3p; 30c-5p; 98-5p; let-7f-5p; 15a-5p; 34a-5p; 7-5p; 411-5p; 665; 576-5p; 15b-5p; 103a-3p; 151a-3p; 184; 409-3p; 320a-3p; 382-5p; 130a-3p; 27b-3p; 423-5p; 374b-5p; 181b-5p; 584-5p; 23a-3p; 92b-3p; 93-3p; 17-5p; 484; 192-5p; 421; 497-5p; 154-3p; 26a-5p; 708-5p; 503-5p; 195-5p; 501-5p; 23b-3p; 7704; 133a-3p; 139-5p; 877-5p; 15b-3p; 299-5p; 6511a-3p; 26b-5p; let-7b-5p; 206; 24-3p; 130b-5p; 3925-5p; 337-3p; 454-3p; 329-3p; 889-3p; 99b-5p; 145-5p; 125a-5p; 377-3p; 376b-3p; 140-5p; 30d-5p; 296-5p; 193a-5p; 491-5p; 34c-5p; 9-5p; 301a-3p; 129-5p; 628-5p; 376c-3p; 1307-3p; 769-5p; 1271-5p; 204-5p; 296-3p; 500a-5p; 378a-3p; 543 |
| Glucagon signaling pathway | 6.96E-07 | 85 | 112 | 27a-5p; 107; 483-5p; 342-3p; 590-3p; 378a-5p; 1185-1-3p; 30a-3p; let-7e-5p; 320c; 92a-3p; 542-3p; 100-3p; 374a-5p; 31-5p; 132-5p; let-7a-5p; 197-3p; 320b; 22-3p; 486-3p; 25-3p; 149-5p; 181a-2-3p; 128-3p; 126-3p; 99a-5p; 323b-3p; 205-5p; 1-3p; 374a-3p; 30e-5p; 130b-3p; 574-3p; 30c-5p; 485-3p; 98-5p; let-7f-5p; 15a-5p; 34a-5p; 330-5p; 7-5p; 665; 576-5p; 15b-5p; 103a-3p; let-7a-3p; 191-5p; 184; 409-3p; 320a-3p; 382-5p; 130a-3p; 27b-3p; 423-5p; 374b-5p; 628-3p; 181b-5p; 23a-3p; 92b-3p; 136-3p; 17-5p; 484; 192-5p; 421; 497-5p; 154-3p; 26a-5p; 328-3p; 708-5p; 503-5p; 675-3p; 195-5p; 941; 501-5p; 23b-3p; 133a-3p; 139-5p; 625-3p; 127-5p; 625-5p; 3613-5p; 26b-5p; 432-5p; let-7b-5p; 206; 24-3p; 130b-5p; 598-3p; 3925-5p; 190a-5p; 454-3p; 329-3p; 145-5p; 125a-5p; 376b-3p; 140-5p; 493-3p; 30d-5p; 491-5p; 34c-5p; 9-5p; 301a-3p; 129-5p; 1307-3p; 769-5p; 1271-5p; 204-5p; 296-3p; 500a-5p; 378a-3p; 543 |
| Bladder cancer | 1.29E-05 | 89 | 112 | 27a-5p; 133a-5p; 107; 483-5p; 342-3p; 590-3p; 378a-5p; 1185-1-3p; 30a-3p; let-7e-5p; 320c; 92a-3p; let-7d-5p; 542-3p; 100-3p; 374a-5p; 31-5p; let-7a-5p; 197-3p; 320b; 22-3p; 486-3p; 25-3p; 149-5p; 128-3p; 126-3p; 99a-5p; 323b-3p; 205-5p; 1-3p; 323a-3p; 30e-5p; 130b-3p; 574-3p; 30c-5p; 485-3p; 98-5p; let-7f-5p; 15a-5p; 34a-5p; 330-5p; 7-5p; 665; 576-5p; 15b-5p; 103a-3p; let-7a-3p; 191-5p; 7706; 184; 320a-3p; 130a-3p; 27b-3p; 423-5p; 374b-5p; 181b-5p; 584-5p; 23a-3p; 22-5p; 92b-3p; 93-3p; 17-5p; 484; 192-5p; 421; 497-5p; 154-3p; 26a-5p; 708-5p; 503-5p; 675-3p; 195-5p; 23b-3p; 7704; 133a-3p; 139-5p; 877-5p; 127-5p; 625-5p; 299-5p; 26b-5p; 432-5p; let-7b-5p; 206; 24-3p; 3925-5p; 671-5p; 454-3p; 329-3p; 889-3p; 145-5p; 125a-5p; 377-3p; 376b-3p; 140-5p; 30d-5p; 296-5p; 193a-5p; 491-5p; 34c-5p; 9-5p; 301a-3p; 129-5p; 376c-3p; 1307-3p; 769-5p; 1271-5p; 204-5p; 296-3p; 125b-1-3p; 378a-3p; 543 |
| Th17 cell differentiation | 5.77E-05 | 91 | 112 | 107; 483-5p; 342-3p; 590-3p; 378a-5p; 1185-1-3p; 30a-3p; let-7e-5p; 362-5p; 320c; 92a-3p; let-7d-5p; 374a-5p; 31-5p; let-7a-5p; 197-3p; 320b; 22-3p; 486-3p; 25-3p; 149-5p; 181a-2-3p; 128-3p; 126-3p; 99a-5p; 323b-3p; 205-5p; 1-3p; 323a-3p; 379-5p; 374a-3p; 30e-5p; 130b-3p; 574-3p; 30c-5p; 485-3p; 98-5p; let-7f-5p; 15a-5p; 34a-5p; 7-5p; 665; 576-5p; 15b-5p; 103a-3p; let-7a-3p; 151a-3p; 184; 409-3p; 320a-3p; 130a-3p; 27b-3p; 423-5p; 374b-5p; 181b-5p; 6724-5p; 584-5p; 23a-3p; 22-5p; 92b-3p; 93-3p; 17-5p; 484; 192-5p; 421; 497-5p; 154-3p; 26a-5p; 708-5p; 503-5p; 195-5p; 501-5p; 23b-3p; 7704; 139-5p; 877-5p; 625-3p; 127-5p; 625-5p; 299-5p; 3613-5p; 26b-5p; let-7b-5p; 206; 24-3p; 130b-5p; 337-3p; 190a-5p; 454-3p; 329-3p; 889-3p; 99b-5p; 145-5p; 125a-5p; 377-3p; 140-5p; 493-3p; 30d-5p; 193a-5p; 491-5p; 34c-5p; 9-5p; 301a-3p; 129-5p; 376c-3p; 1307-3p; 769-5p; 204-5p; 296-3p; 500a-5p; 378a-3p; 543 |
| Relaxin signaling pathway | 5.14E-04 | 94 | 112 | 27a-5p; 133a-5p; 107; 483-5p; 342-3p; 590-3p; 1185-1-3p; 30a-3p; let-7e-5p; 320c; 320d; 92a-3p; let-7d-5p; 542-3p; 100-3p; 374a-5p; 31-5p; let-7a-5p; 197-3p; 320b; 22-3p; 486-3p; 25-3p; 149-5p; 181a-2-3p; 128-3p; 126-3p; 99a-5p; 323b-3p; 205-5p; 1-3p; 323a-3p; 379-5p; 374a-3p; 30e-5p; 130b-3p; 574-3p; 30c-5p; 98-5p; let-7f-5p; 15a-5p; 34a-5p; 330-5p; 7-5p; 411-5p; 665; 576-5p; 15b-5p; 103a-3p; let-7a-3p; 127-3p; 184; 409-3p; 320a-3p; 130a-3p; 27b-3p; 423-5p; 374b-5p; 181b-5p; 584-5p; 23a-3p; 92b-3p; 93-3p; 17-5p; 484; 192-5p; 421; 497-5p; 154-3p; 26a-5p; 708-5p; 503-5p; 195-5p; 23b-3p; 133a-3p; 139-5p; 877-5p; 625-3p; 625-5p; 26b-5p; 432-5p; let-7b-5p; 206; 24-3p; 130b-5p; 3925-5p; 671-5p; 454-3p; 329-3p; 889-3p; 145-5p; 125a-5p; 376b-3p; 140-5p; 493-3p; 30d-5p; 296-5p; 193a-5p; 491-5p; 34c-5p; 9-5p; 301a-3p; 129-5p; 376c-3p; 1307-3p; 769-5p; 204-5p; 1287-5p; 296-3p; 125b-1-3p; 378a-3p; 543 |
| Phospholipase D signaling pathway | 2.84E-04 | 92 | 111 | 27a-5p; 133a-5p; 107; 483-5p; 342-3p; 378a-5p; 30a-3p; let-7e-5p; 320c; 320d; 92a-3p; let-7d-5p; 542-3p; 100-3p; 374a-5p; 31-5p; let-7a-5p; 197-3p; 320b; 22-3p; 486-3p; 25-3p; 149-5p; 128-3p; 126-3p; 99a-5p; 205-5p; 1306-5p; 1-3p; 374a-3p; 30e-5p; 130b-3p; 574-3p; 30c-5p; 98-5p; let-7f-5p; 15a-5p; 34a-5p; 330-5p; 7-5p; 411-5p; 665; 576-5p; 15b-5p; 103a-3p; 191-5p; 127-3p; 151a-3p; 184; 409-3p; 320a-3p; 130a-3p; 27b-3p; 423-5p; 374b-5p; 181b-5p; 584-5p; 23a-3p; 92b-3p; 93-3p; 17-5p; 484; 192-5p; 421; 497-5p; 154-3p; 26a-5p; 708-5p; 503-5p; 195-5p; 23b-3p; 133a-3p; 139-5p; 877-5p; 625-3p; 127-5p; 625-5p; 26b-5p; 432-5p; let-7b-5p; 206; 24-3p; 130b-5p; 598-3p; 3925-5p; 671-5p; 454-3p; 329-3p; 889-3p; 99b-5p; 145-5p; 125a-5p; 99b-3p; 376b-3p; 140-5p; 30d-5p; 193a-5p; 491-5p; 34c-5p; 9-5p; 301a-3p; 129-5p; 628-5p; 376c-3p; 1307-3p; 769-5p; 204-5p; 296-3p; 378a-3p; 1185-5p; 543 |
| T cell receptor signaling pathway | 1.72E-03 | 95 | 111 | 27a-5p; 133a-5p; 107; 483-5p; 342-3p; 590-3p; 378a-5p; 1185-1-3p; 30a-3p; let-7e-5p; 320c; 92a-3p; let-7d-5p; 542-3p; 374a-5p; 31-5p; let-7a-5p; 197-3p; 320b; 22-3p; 486-3p; 25-3p; 149-5p; 181a-2-3p; 128-3p; 126-3p; 99a-5p; 323b-3p; 205-5p; 1306-5p; 1-3p; 374a-3p; 30e-5p; 130b-3p; 30c-5p; 98-5p; let-7f-5p; 15a-5p; 34a-5p; 330-5p; 7-5p; 411-5p; 665; 15b-5p; 103a-3p; let-7a-3p; 151a-3p; 7706; 184; 409-3p; 320a-3p; 130a-3p; 27b-3p; 423-5p; 374b-5p; 181b-5p; 6724-5p; 584-5p; 23a-3p; 92b-3p; 93-3p; 17-5p; 484; 192-5p; 421; 497-5p; 154-3p; 26a-5p; 708-5p; 503-5p; 195-5p; 501-5p; 23b-3p; 133a-3p; 139-5p; 877-5p; 625-3p; 625-5p; 6511a-3p; 26b-5p; let-7b-5p; 206; 24-3p; 130b-5p; 3925-5p; 337-3p; 671-5p; 454-3p; 329-3p; 889-3p; 145-5p; 125a-5p; 377-3p; 99b-3p; 493-3p; 30d-5p; 193a-5p; 34c-5p; 9-5p; 301a-3p; 129-5p; 376c-3p; 1307-3p; 769-5p; 1271-5p; 204-5p; 296-3p; 125b-1-3p; 378a-3p; 1185-5p; 543 |
| Neuroactive ligand-receptor interaction | 3.15E-03 | 96 | 111 | 133a-5p; 107; 483-5p; 342-3p; 590-3p; 378a-5p; 1185-1-3p; 30a-3p; let-7e-5p; 320c; 320d; 92a-3p; let-7d-5p; 542-3p; 100-3p; 374a-5p; 31-5p; let-7a-5p; 197-3p; 320b; 22-3p; 25-3p; 149-5p; 128-3p; 126-3p; 99a-5p; 205-5p; 1306-5p; 629-5p; 1-3p; 323a-3p; 379-5p; 374a-3p; 130b-3p; 30c-5p; 98-5p; let-7f-5p; 15a-5p; 34a-5p; 330-5p; 7-5p; 411-5p; 665; 15b-5p; 103a-3p; let-7a-3p; 151a-3p; 184; 320a-3p; 382-5p; 130a-3p; 27b-3p; 423-5p; 374b-5p; 181b-5p; 584-5p; 23a-3p; 22-5p; 92b-3p; 136-3p; 93-3p; 17-5p; 484; 192-5p; 421; 497-5p; 26a-5p; 708-5p; 503-5p; 675-3p; 195-5p; 501-5p; 23b-3p; 7704; 133a-3p; 139-5p; 877-5p; 127-5p; 625-5p; 664a-5p; 15b-3p; 6511a-3p; 26b-5p; 432-5p; let-7b-5p; 206; 24-3p; 130b-5p; 598-3p; 3925-5p; 337-3p; 671-5p; 190a-5p; 454-3p; 329-3p; 889-3p; 125a-5p; 377-3p; 34c-5p; 9-5p; 301a-3p; 129-5p; 376c-3p; 1307-3p; 769-5p; 1271-5p; 204-5p; 296-3p; 125b-1-3p; 500a-5p; 1185-5p |
| Alzheimer disease | 6.55E-03 | 97 | 111 | 107; 483-5p; 590-3p; 378a-5p; 1185-1-3p; let-7e-5p; 362-5p; 320c; 92a-3p; let-7d-5p; 374a-5p; 31-5p; let-7a-5p; 197-3p; 320b; 22-3p; 486-3p; 25-3p; 149-5p; 181a-2-3p; 128-3p; 126-3p; 99a-5p; 205-5p; 1306-5p; 629-5p; 1-3p; 379-5p; 30e-5p; 130b-3p; 30c-5p; 98-5p; let-7f-5p; 15a-5p; 34a-5p; 330-5p; 7-5p; 411-5p; 665; 15b-5p; 103a-3p; let-7a-3p; 191-5p; 409-3p; 320a-3p; 382-5p; 130a-3p; 27b-3p; 423-5p; 374b-5p; 628-3p; 181b-5p; 584-5p; 23a-3p; 92b-3p; 93-3p; 17-5p; 484; 192-5p; 421; 497-5p; 154-3p; 26a-5p; 328-3p; 708-5p; 503-5p; 675-3p; 195-5p; 501-5p; 23b-3p; 133a-3p; 877-5p; 127-5p; 625-5p; 664a-5p; 26b-5p; let-7b-5p; 206; 24-3p; 130b-5p; 598-3p; 3925-5p; 671-5p; 190a-5p; 454-3p; 329-3p; 889-3p; 145-5p; 125a-5p; 377-3p; 99b-3p; 376b-3p; 140-5p; 493-3p; 30d-5p; 296-5p; 491-5p; 34c-5p; 9-5p; 301a-3p; 129-5p; 376c-3p; 1307-3p; 769-5p; 204-5p; 1287-5p; 296-3p; 500a-5p; 378a-3p; 1185-5p; 543 |
| Prolactin signaling pathway | 4.17E-05 | 88 | 110 | 27a-5p; 107; 483-5p; 342-3p; 590-3p; 378a-5p; 1185-1-3p; 30a-3p; let-7e-5p; 92a-3p; let-7d-5p; 542-3p; 100-3p; 374a-5p; 31-5p; let-7a-5p; 197-3p; 320b; 22-3p; 486-3p; 25-3p; 149-5p; 181a-2-3p; 128-3p; 126-3p; 99a-5p; 323b-3p; 205-5p; 1-3p; 323a-3p; 374a-3p; 30e-5p; 130b-3p; 30c-5p; 98-5p; let-7f-5p; 15a-5p; 34a-5p; 330-5p; 7-5p; 411-5p; 665; 576-5p; 15b-5p; 103a-3p; let-7a-3p; 191-5p; 7706; 184; 409-3p; 320a-3p; 382-5p; 130a-3p; 27b-3p; 423-5p; 374b-5p; 181b-5p; 584-5p; 23a-3p; 92b-3p; 93-3p; 17-5p; 484; 192-5p; 421; 497-5p; 154-3p; 26a-5p; 708-5p; 503-5p; 195-5p; 501-5p; 23b-3p; 139-5p; 877-5p; 625-3p; 625-5p; 26b-5p; 432-5p; let-7b-5p; 206; 24-3p; 130b-5p; 3925-5p; 337-3p; 671-5p; 454-3p; 329-3p; 889-3p; 145-5p; 125a-5p; 377-3p; 99b-3p; 376b-3p; 140-5p; 30d-5p; 193a-5p; 34c-5p; 9-5p; 301a-3p; 129-5p; 376c-3p; 1307-3p; 769-5p; 1271-5p; 204-5p; 296-3p; 500a-5p; 378a-3p; 543 |
| Ubiquitin mediated proteolysis | 2.24E-03 | 94 | 110 | 107; 483-5p; 342-3p; 590-3p; 378a-5p; 1185-1-3p; let-7e-5p; 92a-3p; let-7d-5p; 542-3p; 374a-5p; 31-5p; let-7a-5p; 197-3p; 22-3p; 25-3p; 149-5p; 181a-2-3p; 128-3p; 99a-5p; 205-5p; 1306-5p; 1-3p; 323a-3p; 379-5p; 374a-3p; 30e-5p; 130b-3p; 574-3p; 30c-5p; 98-5p; let-7f-5p; 15a-5p; 34a-5p; 330-5p; 7-5p; 411-5p; 665; 15b-5p; 103a-3p; let-7a-3p; 151a-3p; 409-3p; 320a-3p; 382-5p; 130a-3p; 27b-3p; 423-5p; 374b-5p; 181b-5p; 584-5p; 23a-3p; 22-5p; 92b-3p; 136-3p; 93-3p; 17-5p; 484; 192-5p; 421; 497-5p; 26a-5p; 328-3p; 503-5p; 675-3p; 195-5p; 941; 501-5p; 23b-3p; 7704; 133a-3p; 877-5p; 127-5p; 625-5p; 664a-5p; 15b-3p; 299-5p; 3613-5p; 26b-5p; 432-5p; let-7b-5p; 24-3p; 130b-5p; 671-5p; 454-3p; 329-3p; 889-3p; 145-5p; 125a-5p; 377-3p; 99b-3p; 376b-3p; 140-5p; 493-3p; 30d-5p; 491-5p; 34c-5p; 9-5p; 301a-3p; 129-5p; 628-5p; 376c-3p; 1307-3p; 769-5p; 1271-5p; 204-5p; 296-3p; 500a-5p; 378a-3p; 543 |
| Leukocyte transendothelial migration | 4.03E-03 | 95 | 110 | 27a-5p; 133a-5p; 107; 483-5p; 342-3p; 590-3p; 378a-5p; 1185-1-3p; 30a-3p; let-7e-5p; 320c; 320d; 92a-3p; let-7d-5p; 542-3p; 374a-5p; 31-5p; let-7a-5p; 197-3p; 320b; 486-3p; 25-3p; 149-5p; 181a-2-3p; 128-3p; 126-3p; 99a-5p; 205-5p; 1306-5p; 1-3p; 379-5p; 374a-3p; 30e-5p; 130b-3p; 574-3p; 30c-5p; 98-5p; let-7f-5p; 15a-5p; 34a-5p; 330-5p; 7-5p; 665; 576-5p; 15b-5p; 103a-3p; let-7a-3p; 7706; 184; 409-3p; 320a-3p; let-7i-3p; 27b-3p; 423-5p; 374b-5p; 181b-5p; 584-5p; 23a-3p; 22-5p; 92b-3p; 93-3p; 17-5p; 484; 192-5p; 421; 497-5p; 26a-5p; 328-3p; 708-5p; 503-5p; 195-5p; 23b-3p; 7704; 133a-3p; 139-5p; 877-5p; 625-3p; 127-5p; 625-5p; 3613-5p; 6511a-3p; 26b-5p; let-7b-5p; 24-3p; 130b-5p; 3925-5p; 337-3p; 671-5p; 454-3p; 329-3p; 99b-5p; 145-5p; 125a-5p; 377-3p; 99b-3p; 493-3p; 30d-5p; 193a-5p; 491-5p; 34c-5p; 9-5p; 129-5p; 376c-3p; 1307-3p; 769-5p; 204-5p; 1287-5p; 296-3p; 378a-3p; 543 |
| NF-kappa B signaling pathway | 1.66E-05 | 86 | 109 | 107; 483-5p; 342-3p; 590-3p; 378a-5p; 30a-3p; let-7e-5p; 362-5p; 92a-3p; let-7d-5p; 542-3p; 100-3p; 374a-5p; 31-5p; let-7a-5p; 197-3p; 320b; 22-3p; 486-3p; 25-3p; 149-5p; 181a-2-3p; 128-3p; 126-3p; 205-5p; 1306-5p; 629-5p; 1-3p; 379-5p; 30e-5p; 130b-3p; 30c-5p; 98-5p; let-7f-5p; 15a-5p; 34a-5p; 330-5p; 7-5p; 411-5p; 665; 15b-5p; 103a-3p; 151a-3p; 7706; 184; 409-3p; 320a-3p; 130a-3p; 27b-3p; 423-5p; 181b-5p; 584-5p; 23a-3p; 22-5p; 92b-3p; 136-3p; 93-3p; 17-5p; 484; 192-5p; 421; 497-5p; 26a-5p; 328-3p; 708-5p; 503-5p; 195-5p; 23b-3p; 133a-3p; 139-5p; 877-5p; 625-3p; 625-5p; 15b-3p; 3613-5p; 6511a-3p; 26b-5p; 432-5p; let-7b-5p; 206; 24-3p; 130b-5p; 3925-5p; 337-3p; 454-3p; 329-3p; 889-3p; 145-5p; 125a-5p; 377-3p; 99b-3p; 30d-5p; 193a-5p; 491-5p; 34c-5p; 9-5p; 301a-3p; 129-5p; 628-5p; 376c-3p; 1307-3p; 769-5p; 1271-5p; 204-5p; 296-3p; 500a-5p; 378a-3p; 1185-5p; 543 |
| PD-L1 expression and PD-1 checkpoint pathway in cancer | 5.19E-05 | 86 | 108 | 27a-5p; 133a-5p; 107; 483-5p; 342-3p; 590-3p; 378a-5p; 1185-1-3p; 30a-3p; let-7e-5p; 362-5p; 92a-3p; 542-3p; 374a-5p; let-7a-5p; 197-3p; 320b; 22-3p; 486-3p; 25-3p; 149-5p; 181a-2-3p; 128-3p; 126-3p; 99a-5p; 323b-3p; 205-5p; 1-3p; 323a-3p; 374a-3p; 30e-5p; 130b-3p; 574-3p; 30c-5p; 98-5p; 15a-5p; 34a-5p; 330-5p; 7-5p; 411-5p; 665; 15b-5p; 103a-3p; 151a-3p; 7706; 184; 409-3p; 320a-3p; 382-5p; 130a-3p; 27b-3p; 374b-5p; 181b-5p; 6724-5p; 584-5p; 23a-3p; 92b-3p; 93-3p; 17-5p; 484; 192-5p; 497-5p; 154-3p; 26a-5p; 708-5p; 503-5p; 195-5p; 501-5p; 23b-3p; 133a-3p; 139-5p; 877-5p; 625-3p; 299-5p; 26b-5p; let-7b-5p; 206; 24-3p; 130b-5p; 3925-5p; 337-3p; 671-5p; 454-3p; 329-3p; 889-3p; 99b-5p; 145-5p; 125a-5p; 377-3p; 140-5p; 493-3p; 30d-5p; 193a-5p; 491-5p; 34c-5p; 9-5p; 301a-3p; 129-5p; 1307-3p; 769-5p; 1271-5p; 204-5p; 1287-5p; 296-3p; 500a-5p; 378a-3p; 1185-5p; 543 |
| Fc gamma R-mediated phagocytosis | 5.11E-04 | 90 | 108 | 27a-5p; 107; 483-5p; 342-3p; 590-3p; 378a-5p; 30a-3p; let-7e-5p; 362-5p; 320c; 320d; 92a-3p; let-7d-5p; 542-3p; 374a-5p; 31-5p; let-7a-5p; 197-3p; 320b; 22-3p; 486-3p; 25-3p; 149-5p; 128-3p; 126-3p; 99a-5p; 205-5p; 1306-5p; 1-3p; 374a-3p; 30e-5p; 130b-3p; 574-3p; 30c-5p; 98-5p; let-7f-5p; 15a-5p; 34a-5p; 330-5p; 7-5p; 411-5p; 665; 576-5p; 15b-5p; 103a-3p; 7706; 184; 409-3p; 320a-3p; 130a-3p; 27b-3p; 423-5p; 374b-5p; 181b-5p; 584-5p; 23a-3p; 92b-3p; 136-3p; 93-3p; 17-5p; 484; 192-5p; 497-5p; 154-3p; 26a-5p; 708-5p; 503-5p; 195-5p; 23b-3p; 133a-3p; 139-5p; 877-5p; 625-3p; 127-5p; 625-5p; 3613-5p; 6511a-3p; 26b-5p; 432-5p; let-7b-5p; 206; 24-3p; 130b-5p; 671-5p; 454-3p; 329-3p; 889-3p; 99b-5p; 145-5p; 125a-5p; 377-3p; 99b-3p; 493-3p; 30d-5p; 193a-5p; 491-5p; 34c-5p; 9-5p; 301a-3p; 129-5p; 628-5p; 376c-3p; 1307-3p; 769-5p; 204-5p; 296-3p; 500a-5p; 378a-3p |
| Necroptosis | 1.79E-02 | 96 | 108 | 133a-5p; 107; 483-5p; 342-3p; 590-3p; 378a-5p; let-7e-5p; 362-5p; 320c; 92a-3p; let-7d-5p; 374a-5p; 31-5p; let-7a-5p; 197-3p; 320b; 22-3p; 486-3p; 25-3p; 149-5p; 181a-2-3p; 128-3p; 126-3p; 99a-5p; 205-5p; 1306-5p; 629-5p; 1-3p; 323a-3p; 379-5p; 374a-3p; 30e-5p; 130b-3p; 30c-5p; 485-3p; 98-5p; let-7f-5p; 15a-5p; 34a-5p; 7-5p; 411-5p; 665; 15b-5p; 103a-3p; 191-5p; 184; 409-3p; 320a-3p; 130a-3p; 27b-3p; 423-5p; 181b-5p; 584-5p; 23a-3p; 92b-3p; 93-3p; 17-5p; 484; 192-5p; 421; 497-5p; 26a-5p; 328-3p; 708-5p; 503-5p; 195-5p; 941; 501-5p; 23b-3p; 7704; 133a-3p; 139-5p; 877-5p; 625-5p; 15b-3p; 299-5p; 26b-5p; 432-5p; let-7b-5p; 206; 24-3p; 130b-5p; 3925-5p; 337-3p; 671-5p; 454-3p; 329-3p; 889-3p; 99b-5p; 145-5p; 125a-5p; 376b-3p; 140-5p; 30d-5p; 296-5p; 34c-5p; 9-5p; 301a-3p; 129-5p; 376c-3p; 1307-3p; 769-5p; 1271-5p; 204-5p; 1287-5p; 296-3p; 500a-5p; 378a-3p |
| Toll-like receptor signaling pathway | 9.65E-08 | 78 | 107 | 27a-5p; 107; 483-5p; 342-3p; 590-3p; 378a-5p; 1185-1-3p; let-7e-5p; 362-5p; 92a-3p; let-7d-5p; 542-3p; 100-3p; 374a-5p; let-7a-5p; 197-3p; 320b; 22-3p; 25-3p; 149-5p; 181a-2-3p; 128-3p; 126-3p; 99a-5p; 323b-3p; 205-5p; 1306-5p; 1-3p; 374a-3p; 30e-5p; 130b-3p; 574-3p; 30c-5p; 98-5p; let-7f-5p; 15a-5p; 34a-5p; 330-5p; 7-5p; 411-5p; 665; 576-5p; 15b-5p; 103a-3p; 151a-3p; 184; 409-3p; 320a-3p; 130a-3p; 27b-3p; 423-5p; 374b-5p; 181b-5p; 584-5p; 23a-3p; 92b-3p; 93-3p; 17-5p; 484; 192-5p; 421; 497-5p; 154-3p; 26a-5p; 328-3p; 708-5p; 503-5p; 195-5p; 501-5p; 23b-3p; 7704; 139-5p; 877-5p; 625-3p; 127-5p; 299-5p; 6511a-3p; 26b-5p; let-7b-5p; 206; 24-3p; 130b-5p; 454-3p; 329-3p; 889-3p; 145-5p; 125a-5p; 376b-3p; 140-5p; 493-3p; 30d-5p; 296-5p; 193a-5p; 491-5p; 34c-5p; 9-5p; 301a-3p; 129-5p; 628-5p; 1307-3p; 769-5p; 204-5p; 296-3p; 125b-1-3p; 500a-5p; 378a-3p; 543 |
| Cholinergic synapse | 2.32E-05 | 84 | 107 | 27a-5p; 107; 483-5p; 342-3p; 590-3p; 1185-1-3p; 30a-3p; let-7e-5p; 320c; 320d; 92a-3p; let-7d-5p; 542-3p; 100-3p; 374a-5p; let-7a-5p; 197-3p; 320b; 22-3p; 486-3p; 25-3p; 149-5p; 181a-2-3p; 128-3p; 126-3p; 99a-5p; 323b-3p; 205-5p; 1-3p; 374a-3p; 30e-5p; 130b-3p; 30c-5p; 98-5p; let-7f-5p; 15a-5p; 34a-5p; 330-5p; 7-5p; 665; 576-5p; 15b-5p; 103a-3p; let-7a-3p; 127-3p; 151a-3p; 184; 409-3p; 320a-3p; 130a-3p; 27b-3p; 423-5p; 374b-5p; 181b-5p; 584-5p; 23a-3p; 92b-3p; 93-3p; 17-5p; 484; 192-5p; 497-5p; 154-3p; 26a-5p; 708-5p; 503-5p; 675-3p; 195-5p; 501-5p; 23b-3p; 133a-3p; 139-5p; 877-5p; 625-3p; 625-5p; 15b-3p; 26b-5p; let-7b-5p; 206; 24-3p; 130b-5p; 3925-5p; 671-5p; 190a-5p; 454-3p; 329-3p; 889-3p; 145-5p; 125a-5p; 377-3p; 493-3p; 30d-5p; 296-5p; 193a-5p; 34c-5p; 9-5p; 301a-3p; 129-5p; 376c-3p; 1307-3p; 769-5p; 204-5p; 1287-5p; 296-3p; 500a-5p; 378a-3p; 543 |
| Progesterone-mediated oocyte maturation | 2.01E-03 | 91 | 107 | 27a-5p; 107; 483-5p; 342-3p; 590-3p; 378a-5p; 30a-3p; let-7e-5p; 362-5p; 320c; 320d; 92a-3p; let-7d-5p; 542-3p; 374a-5p; 31-5p; let-7a-5p; 197-3p; 320b; 22-3p; 486-3p; 25-3p; 149-5p; 181a-2-3p; 128-3p; 126-3p; 99a-5p; 205-5p; 1-3p; 374a-3p; 30e-5p; 130b-3p; 30c-5p; 98-5p; let-7f-5p; 15a-5p; 34a-5p; 330-5p; 7-5p; 665; 576-5p; 15b-5p; 103a-3p; 191-5p; 184; 409-3p; 320a-3p; 130a-3p; 27b-3p; 374b-5p; 181b-5p; 584-5p; 23a-3p; 92b-3p; 93-3p; 17-5p; 484; 192-5p; 421; 497-5p; 154-3p; 26a-5p; 708-5p; 503-5p; 675-3p; 195-5p; 501-5p; 23b-3p; 133a-3p; 139-5p; 877-5p; 625-3p; 625-5p; 15b-3p; 26b-5p; 432-5p; let-7b-5p; 206; 24-3p; 130b-5p; 671-5p; 190a-5p; 454-3p; 329-3p; 889-3p; 99b-5p; 145-5p; 125a-5p; 377-3p; 376b-3p; 140-5p; 30d-5p; 296-5p; 193a-5p; 34c-5p; 9-5p; 301a-3p; 129-5p; 376c-3p; 1307-3p; 769-5p; 1271-5p; 204-5p; 296-3p; 125b-1-3p; 378a-3p; 543 |
| Phagosome | 9.29E-03 | 94 | 107 | 27a-5p; 107; 342-3p; 590-3p; 378a-5p; 1185-1-3p; 30a-3p; let-7e-5p; 320c; 92a-3p; let-7d-5p; 542-3p; 374a-5p; 31-5p; let-7a-5p; 197-3p; 320b; 22-3p; 486-3p; 25-3p; 149-5p; 181a-2-3p; 128-3p; 323b-3p; 205-5p; 1306-5p; 1-3p; 323a-3p; 374a-3p; 30e-5p; 130b-3p; 574-3p; 30c-5p; 98-5p; let-7f-5p; 15a-5p; 34a-5p; 330-5p; 7-5p; 665; 576-5p; 15b-5p; 103a-3p; 320a-3p; 382-5p; let-7i-3p; 130a-3p; 27b-3p; 423-5p; 374b-5p; 181b-5p; 23a-3p; 22-5p; 92b-3p; 136-3p; 93-3p; 17-5p; 484; 192-5p; 421; 497-5p; 26a-5p; 328-3p; 708-5p; 503-5p; 675-3p; 195-5p; 501-5p; 23b-3p; 7704; 133a-3p; 877-5p; 127-5p; 625-5p; 664a-5p; 299-5p; 26b-5p; 432-5p; let-7b-5p; 206; 24-3p; 130b-5p; 3925-5p; 671-5p; 454-3p; 329-3p; 889-3p; 99b-5p; 145-5p; 125a-5p; 377-3p; 99b-3p; 493-3p; 30d-5p; 296-5p; 491-5p; 34c-5p; 9-5p; 301a-3p; 129-5p; 628-5p; 1307-3p; 769-5p; 204-5p; 296-3p; 378a-3p; 543 |
| Renal cell carcinoma | 1.08E-02 | 94 | 107 | 27a-5p; 107; 483-5p; 342-3p; 378a-5p; 30a-3p; let-7e-5p; 320c; 320d; 92a-3p; let-7d-5p; 542-3p; 374a-5p; 31-5p; let-7a-5p; 197-3p; 320b; 22-3p; 486-3p; 25-3p; 149-5p; 128-3p; 126-3p; 99a-5p; 205-5p; 1306-5p; 1-3p; 374a-3p; 30e-5p; 130b-3p; 574-3p; 30c-5p; 98-5p; let-7f-5p; 15a-5p; 34a-5p; 330-5p; 7-5p; 411-5p; 665; 576-5p; 15b-5p; 103a-3p; 191-5p; 151a-3p; 184; 409-3p; 320a-3p; 130a-3p; 27b-3p; 423-5p; 374b-5p; 628-3p; 181b-5p; 584-5p; 23a-3p; 92b-3p; 93-3p; 17-5p; 484; 192-5p; 421; 497-5p; 154-3p; 26a-5p; 708-5p; 503-5p; 195-5p; 23b-3p; 7704; 133a-3p; 139-5p; 877-5p; 625-3p; 127-5p; 625-5p; 299-5p; 26b-5p; let-7b-5p; 206; 24-3p; 130b-5p; 3925-5p; 337-3p; 671-5p; 454-3p; 329-3p; 889-3p; 145-5p; 125a-5p; 377-3p; 140-5p; 30d-5p; 296-5p; 193a-5p; 34c-5p; 9-5p; 301a-3p; 129-5p; 376c-3p; 1307-3p; 769-5p; 1271-5p; 204-5p; 296-3p; 378a-3p; 543 |
| B cell receptor signaling pathway | 4.07E-05 | 84 | 106 | 27a-5p; 107; 483-5p; 342-3p; 590-3p; 1185-1-3p; 30a-3p; let-7e-5p; 92a-3p; let-7d-5p; 542-3p; 374a-5p; 31-5p; let-7a-5p; 197-3p; 320b; 22-3p; 486-3p; 25-3p; 149-5p; 181a-2-3p; 128-3p; 126-3p; 99a-5p; 323b-3p; 205-5p; 1306-5p; 1-3p; 374a-3p; 30e-5p; 130b-3p; 574-3p; 30c-5p; 98-5p; let-7f-5p; 15a-5p; 34a-5p; 330-5p; 7-5p; 411-5p; 665; 576-5p; 15b-5p; 103a-3p; let-7a-3p; 151a-3p; 7706; 184; 409-3p; 320a-3p; 130a-3p; 27b-3p; 423-5p; 374b-5p; 181b-5p; 584-5p; 23a-3p; 92b-3p; 93-3p; 17-5p; 484; 192-5p; 497-5p; 154-3p; 26a-5p; 708-5p; 503-5p; 195-5p; 501-5p; 23b-3p; 139-5p; 877-5p; 625-3p; 625-5p; 6511a-3p; 26b-5p; let-7b-5p; 206; 24-3p; 130b-5p; 3925-5p; 337-3p; 671-5p; 454-3p; 329-3p; 889-3p; 145-5p; 125a-5p; 377-3p; 99b-3p; 493-3p; 30d-5p; 193a-5p; 34c-5p; 9-5p; 301a-3p; 129-5p; 628-5p; 376c-3p; 1307-3p; 769-5p; 1271-5p; 204-5p; 296-3p; 378a-3p; 543 |
| Choline metabolism in cancer | 2.51E-04 | 86 | 106 | 27a-5p; 133a-5p; 107; 483-5p; 342-3p; 590-3p; 378a-5p; 30a-3p; let-7e-5p; 362-5p; 92a-3p; let-7d-5p; 542-3p; 374a-5p; 31-5p; let-7a-5p; 197-3p; 320b; 22-3p; 486-3p; 25-3p; 149-5p; 128-3p; 126-3p; 99a-5p; 323b-3p; 1306-5p; 1-3p; 374a-3p; 30e-5p; 130b-3p; 574-3p; 30c-5p; 98-5p; let-7f-5p; 15a-5p; 34a-5p; 330-5p; 7-5p; 411-5p; 665; 576-5p; 15b-5p; 103a-3p; 151a-3p; 184; 409-3p; 320a-3p; 130a-3p; 27b-3p; 423-5p; 374b-5p; 181b-5p; 584-5p; 23a-3p; 92b-3p; 93-3p; 17-5p; 484; 192-5p; 421; 497-5p; 154-3p; 26a-5p; 708-5p; 503-5p; 195-5p; 23b-3p; 133a-3p; 139-5p; 877-5p; 625-3p; 127-5p; 625-5p; 299-5p; 26b-5p; let-7b-5p; 206; 24-3p; 130b-5p; 3925-5p; 671-5p; 454-3p; 329-3p; 889-3p; 99b-5p; 145-5p; 125a-5p; 140-5p; 30d-5p; 296-5p; 193a-5p; 491-5p; 34c-5p; 9-5p; 301a-3p; 129-5p; 628-5p; 376c-3p; 1307-3p; 769-5p; 204-5p; 296-3p; 500a-5p; 378a-3p; 543 |
| Melanogenesis | 1.82E-03 | 90 | 106 | 27a-5p; 107; 483-5p; 590-3p; 378a-5p; 1185-1-3p; 30a-3p; let-7e-5p; 320c; 320d; 92a-3p; let-7d-5p; 542-3p; 374a-5p; 31-5p; let-7a-5p; 197-3p; 320b; 22-3p; 486-3p; 25-3p; 149-5p; 128-3p; 126-3p; 99a-5p; 205-5p; 1-3p; 379-5p; 30e-5p; 130b-3p; 574-3p; 30c-5p; 485-3p; 98-5p; let-7f-5p; 15a-5p; 34a-5p; 330-5p; 7-5p; 665; 576-5p; 15b-5p; 103a-3p; let-7a-3p; 184; 409-3p; 320a-3p; 382-5p; 130a-3p; 27b-3p; 423-5p; 374b-5p; 628-3p; 181b-5p; 584-5p; 23a-3p; 92b-3p; 136-3p; 93-3p; 17-5p; 484; 192-5p; 421; 497-5p; 154-3p; 26a-5p; 195-5p; 501-5p; 23b-3p; 139-5p; 877-5p; 625-5p; 26b-5p; 432-5p; let-7b-5p; 206; 24-3p; 130b-5p; 598-3p; 3925-5p; 671-5p; 190a-5p; 454-3p; 329-3p; 889-3p; 145-5p; 125a-5p; 377-3p; 99b-3p; 376b-3p; 140-5p; 493-3p; 30d-5p; 491-5p; 34c-5p; 9-5p; 301a-3p; 129-5p; 376c-3p; 1307-3p; 769-5p; 204-5p; 296-3p; 500a-5p; 378a-3p; 543 |
| GnRH signaling pathway | 7.71E-06 | 81 | 105 | 27a-5p; 133a-5p; 107; 483-5p; 342-3p; 590-3p; 1185-1-3p; 30a-3p; let-7e-5p; 92a-3p; let-7d-5p; 542-3p; 374a-5p; 31-5p; let-7a-5p; 197-3p; 320b; 22-3p; 486-3p; 25-3p; 149-5p; 181a-2-3p; 128-3p; 126-3p; 99a-5p; 205-5p; 1-3p; 323a-3p; 30e-5p; 130b-3p; 574-3p; 30c-5p; 98-5p; let-7f-5p; 15a-5p; 34a-5p; 330-5p; 7-5p; 411-5p; 665; 15b-5p; 103a-3p; 191-5p; 184; 320a-3p; 382-5p; 130a-3p; 27b-3p; 423-5p; 374b-5p; 181b-5p; 584-5p; 92b-3p; 93-3p; 17-5p; 484; 192-5p; 421; 497-5p; 154-3p; 26a-5p; 708-5p; 195-5p; 501-5p; 23b-3p; 133a-3p; 139-5p; 877-5p; 625-3p; 625-5p; 26b-5p; 432-5p; let-7b-5p; 206; 24-3p; 130b-5p; 598-3p; 3925-5p; 671-5p; 190a-5p; 454-3p; 329-3p; 889-3p; 99b-5p; 145-5p; 125a-5p; 377-3p; 376b-3p; 493-3p; 30d-5p; 491-5p; 34c-5p; 9-5p; 301a-3p; 129-5p; 376c-3p; 1307-3p; 769-5p; 1271-5p; 204-5p; 296-3p; 125b-1-3p; 500a-5p; 378a-3p; 543 |
| Acute myeloid leukemia | 1.11E-05 | 81 | 105 | 27a-5p; 107; 483-5p; 342-3p; 378a-5p; 1185-1-3p; 30a-3p; let-7e-5p; 362-5p; 92a-3p; let-7d-5p; 542-3p; 374a-5p; let-7a-5p; 197-3p; 320b; 22-3p; 486-3p; 25-3p; 149-5p; 128-3p; 126-3p; 99a-5p; 323b-3p; 1306-5p; 1-3p; 323a-3p; 374a-3p; 30e-5p; 130b-3p; 30c-5p; 485-3p; 98-5p; let-7f-5p; 15a-5p; 34a-5p; 330-5p; 7-5p; 411-5p; 665; 15b-5p; 103a-3p; let-7a-3p; 191-5p; 151a-3p; 7706; 184; 409-3p; 320a-3p; 130a-3p; 27b-3p; 423-5p; 374b-5p; 181b-5p; 584-5p; 23a-3p; 92b-3p; 93-3p; 17-5p; 484; 192-5p; 421; 497-5p; 154-3p; 26a-5p; 708-5p; 503-5p; 195-5p; 23b-3p; 133a-3p; 139-5p; 877-5p; 625-3p; 625-5p; 6511a-3p; 26b-5p; let-7b-5p; 206; 24-3p; 130b-5p; 3925-5p; 337-3p; 671-5p; 454-3p; 329-3p; 889-3p; 99b-5p; 145-5p; 125a-5p; 377-3p; 30d-5p; 193a-5p; 34c-5p; 9-5p; 301a-3p; 129-5p; 376c-3p; 1307-3p; 769-5p; 1271-5p; 204-5p; 296-3p; 500a-5p; 378a-3p; 543 |
| Retrograde endocannabinoid signaling | 2.02E-04 | 85 | 105 | 107; 483-5p; 590-3p; 378a-5p; 1185-1-3p; 30a-3p; let-7e-5p; 320c; 320d; 92a-3p; let-7d-5p; 542-3p; 100-3p; 374a-5p; let-7a-5p; 197-3p; 320b; 22-3p; 25-3p; 149-5p; 181a-2-3p; 128-3p; 99a-5p; 205-5p; 1-3p; 379-5p; 374a-3p; 30e-5p; 130b-3p; 30c-5p; 485-3p; 98-5p; let-7f-5p; 15a-5p; 34a-5p; 7-5p; 411-5p; 665; 15b-5p; 103a-3p; let-7a-3p; 127-3p; 151a-3p; 184; 320a-3p; 130a-3p; 27b-3p; 423-5p; 374b-5p; 181b-5p; 584-5p; 23a-3p; 92b-3p; 136-3p; 17-5p; 484; 192-5p; 421; 497-5p; 154-3p; 26a-5p; 328-3p; 675-3p; 195-5p; 501-5p; 23b-3p; 133a-3p; 877-5p; 127-5p; 625-5p; 15b-3p; 6511a-3p; 26b-5p; let-7b-5p; 206; 24-3p; 130b-5p; 3925-5p; 671-5p; 454-3p; 329-3p; 889-3p; 99b-5p; 145-5p; 125a-5p; 377-3p; 376b-3p; 140-5p; 493-3p; 30d-5p; 296-5p; 491-5p; 34c-5p; 9-5p; 301a-3p; 129-5p; 1307-3p; 769-5p; 1271-5p; 204-5p; 1287-5p; 296-3p; 500a-5p; 378a-3p; 543 |
| Natural killer cell mediated cytotoxicity | 1.38E-03 | 88 | 105 | 27a-5p; 107; 483-5p; 342-3p; 378a-5p; 30a-3p; let-7e-5p; 92a-3p; let-7d-5p; 542-3p; 374a-5p; 31-5p; let-7a-5p; 197-3p; 320b; 22-3p; 486-3p; 25-3p; 149-5p; 181a-2-3p; 128-3p; 126-3p; 323b-3p; 1306-5p; 1-3p; 323a-3p; 374a-3p; 30e-5p; 130b-3p; 574-3p; 30c-5p; 485-3p; 98-5p; let-7f-5p; 15a-5p; 34a-5p; 330-5p; 7-5p; 411-5p; 665; 576-5p; 15b-5p; 103a-3p; 191-5p; 151a-3p; 7706; 184; 409-3p; 320a-3p; 382-5p; 130a-3p; 27b-3p; 423-5p; 374b-5p; 181b-5p; 584-5p; 23a-3p; 92b-3p; 93-3p; 17-5p; 484; 421; 497-5p; 154-3p; 26a-5p; 503-5p; 195-5p; 501-5p; 23b-3p; 7704; 139-5p; 877-5p; 6511a-3p; 26b-5p; let-7b-5p; 206; 24-3p; 130b-5p; 3925-5p; 671-5p; 454-3p; 329-3p; 889-3p; 145-5p; 125a-5p; 377-3p; 99b-3p; 376b-3p; 30d-5p; 193a-5p; 491-5p; 34c-5p; 9-5p; 301a-3p; 129-5p; 628-5p; 376c-3p; 1307-3p; 769-5p; 1271-5p; 204-5p; 296-3p; 500a-5p; 378a-3p; 543 |
| Longevity regulating pathway - multiple species | 4.01E-04 | 85 | 104 | 27a-5p; 107; 342-3p; 378a-5p; 1185-1-3p; 30a-3p; let-7e-5p; 362-5p; 92a-3p; let-7d-5p; 542-3p; 100-3p; 31-5p; 132-5p; let-7a-5p; 197-3p; 22-3p; 25-3p; 149-5p; 128-3p; 126-3p; 99a-5p; 205-5p; 1-3p; 374a-3p; 30e-5p; 130b-3p; 30c-5p; 98-5p; let-7f-5p; 15a-5p; 34a-5p; 330-5p; 7-5p; 665; 576-5p; 15b-5p; 103a-3p; 191-5p; 184; 409-3p; 320a-3p; 130a-3p; 27b-3p; 374b-5p; 181b-5p; 23a-3p; 92b-3p; 17-5p; 484; 192-5p; 421; 497-5p; 154-3p; 26a-5p; 328-3p; 708-5p; 503-5p; 675-3p; 195-5p; 941; 23b-3p; 133a-3p; 139-5p; 877-5p; 625-3p; 127-5p; 625-5p; 15b-3p; 299-5p; 3613-5p; 6511a-3p; 26b-5p; 432-5p; let-7b-5p; 206; 24-3p; 130b-5p; 3925-5p; 671-5p; 190a-5p; 454-3p; 329-3p; 99b-5p; 145-5p; 125a-5p; 377-3p; 140-5p; 30d-5p; 193a-5p; 34c-5p; 9-5p; 301a-3p; 129-5p; 628-5p; 376c-3p; 1271-5p; 204-5p; 1287-5p; 296-3p; 125b-1-3p; 500a-5p; 378a-3p; 543 |
| Bacterial invasion of epithelial cells | 3.43E-05 | 80 | 103 | 133a-5p; 107; 483-5p; 342-3p; 590-3p; 378a-5p; 30a-3p; let-7e-5p; 320c; 320d; 92a-3p; let-7d-5p; 542-3p; 374a-5p; 31-5p; let-7a-5p; 197-3p; 320b; 22-3p; 25-3p; 149-5p; 128-3p; 126-3p; 205-5p; 1306-5p; 1-3p; 323a-3p; 379-5p; 374a-3p; 30e-5p; 130b-3p; 574-3p; 30c-5p; 485-3p; 98-5p; let-7f-5p; 15a-5p; 34a-5p; 330-5p; 7-5p; 411-5p; 665; 576-5p; 15b-5p; 103a-3p; 151a-3p; 409-3p; 320a-3p; 382-5p; let-7i-3p; 130a-3p; 27b-3p; 423-5p; 374b-5p; 181b-5p; 23a-3p; 92b-3p; 93-3p; 17-5p; 484; 192-5p; 421; 497-5p; 26a-5p; 503-5p; 195-5p; 23b-3p; 133a-3p; 139-5p; 877-5p; 625-3p; 127-5p; 625-5p; 26b-5p; let-7b-5p; 206; 24-3p; 130b-5p; 3925-5p; 671-5p; 454-3p; 329-3p; 99b-5p; 145-5p; 125a-5p; 377-3p; 99b-3p; 376b-3p; 140-5p; 493-3p; 30d-5p; 193a-5p; 491-5p; 34c-5p; 9-5p; 301a-3p; 129-5p; 1307-3p; 769-5p; 204-5p; 296-3p; 378a-3p; 543 |
| Vascular smooth muscle contraction | 5.12E-03 | 88 | 103 | 133a-5p; 107; 483-5p; 342-3p; 590-3p; 378a-5p; 30a-3p; let-7e-5p; 320c; 92a-3p; let-7d-5p; 100-3p; 374a-5p; 31-5p; let-7a-5p; 197-3p; 320b; 22-3p; 486-3p; 25-3p; 149-5p; 181a-2-3p; 128-3p; 126-3p; 99a-5p; 205-5p; 1306-5p; 1-3p; 323a-3p; 379-5p; 30e-5p; 130b-3p; 30c-5p; 98-5p; let-7f-5p; 15a-5p; 34a-5p; 7-5p; 665; 15b-5p; 103a-3p; 191-5p; 184; 320a-3p; 382-5p; 130a-3p; 27b-3p; 423-5p; 374b-5p; 181b-5p; 584-5p; 92b-3p; 136-3p; 93-3p; 17-5p; 484; 192-5p; 421; 497-5p; 154-3p; 26a-5p; 195-5p; 501-5p; 23b-3p; 133a-3p; 139-5p; 877-5p; 625-3p; 127-5p; 625-5p; 664a-5p; 26b-5p; 432-5p; let-7b-5p; 206; 24-3p; 130b-5p; 598-3p; 3925-5p; 190a-5p; 454-3p; 329-3p; 889-3p; 99b-5p; 145-5p; 125a-5p; 377-3p; 376b-3p; 493-3p; 30d-5p; 34c-5p; 9-5p; 301a-3p; 129-5p; 376c-3p; 1307-3p; 769-5p; 1271-5p; 204-5p; 296-3p; 500a-5p; 378a-3p; 1185-5p |
| Mitophagy - animal | 1.66E-05 | 78 | 102 | 107; 483-5p; 342-3p; 590-3p; 378a-5p; 1185-1-3p; 30a-3p; let-7e-5p; 320c; 320d; 92a-3p; let-7d-5p; 100-3p; 374a-5p; 31-5p; let-7a-5p; 197-3p; 320b; 22-3p; 486-3p; 25-3p; 149-5p; 128-3p; 126-3p; 99a-5p; 205-5p; 1-3p; 30e-5p; 130b-3p; 30c-5p; 98-5p; let-7f-5p; 15a-5p; 34a-5p; 330-5p; 7-5p; 411-5p; 665; 576-5p; 15b-5p; 103a-3p; 151a-3p; 7706; 184; 320a-3p; 382-5p; 130a-3p; 27b-3p; 423-5p; 374b-5p; 181b-5p; 23a-3p; 92b-3p; 17-5p; 484; 192-5p; 421; 497-5p; 26a-5p; 708-5p; 195-5p; 23b-3p; 133a-3p; 139-5p; 877-5p; 625-3p; 625-5p; 299-5p; 6511a-3p; 26b-5p; let-7b-5p; 206; 24-3p; 130b-5p; 3925-5p; 337-3p; 671-5p; 190a-5p; 454-3p; 329-3p; 889-3p; 99b-5p; 145-5p; 125a-5p; 377-3p; 376b-3p; 493-3p; 30d-5p; 296-5p; 193a-5p; 491-5p; 34c-5p; 9-5p; 301a-3p; 129-5p; 1307-3p; 204-5p; 1287-5p; 125b-1-3p; 500a-5p; 1185-5p; 543 |
| IL-17 signaling pathway | 2.37E-05 | 79 | 102 | 133a-5p; 107; 483-5p; 342-3p; 590-3p; 1185-1-3p; let-7e-5p; 362-5p; 92a-3p; let-7d-5p; 542-3p; 100-3p; 374a-5p; 31-5p; 132-5p; let-7a-5p; 197-3p; 320b; 486-3p; 25-3p; 149-5p; 181a-2-3p; 128-3p; 126-3p; 99a-5p; 323b-3p; 205-5p; 1-3p; 374a-3p; 30e-5p; 130b-3p; 30c-5p; 98-5p; let-7f-5p; 15a-5p; 34a-5p; 7-5p; 411-5p; 665; 15b-5p; 103a-3p; let-7a-3p; 191-5p; 127-3p; 151a-3p; 409-3p; 320a-3p; 382-5p; 130a-3p; 27b-3p; 423-5p; 374b-5p; 181b-5p; 584-5p; 23a-3p; 22-5p; 92b-3p; 93-3p; 17-5p; 484; 192-5p; 421; 497-5p; 154-3p; 26a-5p; 708-5p; 503-5p; 195-5p; 501-5p; 23b-3p; 139-5p; 877-5p; 625-5p; 26b-5p; let-7b-5p; 206; 24-3p; 130b-5p; 3925-5p; 454-3p; 329-3p; 889-3p; 145-5p; 125a-5p; 377-3p; 99b-3p; 376b-3p; 140-5p; 30d-5p; 296-5p; 491-5p; 34c-5p; 9-5p; 301a-3p; 129-5p; 1307-3p; 769-5p; 204-5p; 296-3p; 500a-5p; 378a-3p; 543 |
| Serotonergic synapse | 5.10E-05 | 79 | 101 | 107; 483-5p; 342-3p; 590-3p; 378a-5p; 30a-3p; let-7e-5p; 320c; 320d; 92a-3p; let-7d-5p; 542-3p; 100-3p; 374a-5p; let-7a-5p; 197-3p; 320b; 22-3p; 486-3p; 25-3p; 128-3p; 126-3p; 205-5p; 1-3p; 374a-3p; 30e-5p; 130b-3p; 30c-5p; 98-5p; let-7f-5p; 15a-5p; 34a-5p; 330-5p; 7-5p; 411-5p; 665; 15b-5p; 103a-3p; 191-5p; 151a-3p; 184; 320a-3p; 382-5p; 130a-3p; 27b-3p; 423-5p; 181b-5p; 584-5p; 23a-3p; 22-5p; 92b-3p; 93-3p; 17-5p; 484; 192-5p; 421; 497-5p; 154-3p; 26a-5p; 328-3p; 195-5p; 501-5p; 23b-3p; 133a-3p; 139-5p; 877-5p; 625-5p; 15b-3p; 26b-5p; 432-5p; let-7b-5p; 206; 24-3p; 130b-5p; 3925-5p; 671-5p; 454-3p; 329-3p; 889-3p; 99b-5p; 145-5p; 125a-5p; 377-3p; 376b-3p; 493-3p; 30d-5p; 296-5p; 491-5p; 34c-5p; 9-5p; 301a-3p; 129-5p; 628-5p; 1307-3p; 769-5p; 1271-5p; 204-5p; 1287-5p; 500a-5p; 378a-3p; 543 |
| Fc epsilon RI signaling pathway | 1.22E-04 | 80 | 101 | 27a-5p; 107; 483-5p; 342-3p; 590-3p; 1185-1-3p; 30a-3p; let-7e-5p; 92a-3p; let-7d-5p; 542-3p; 374a-5p; let-7a-5p; 197-3p; 320b; 22-3p; 486-3p; 25-3p; 149-5p; 181a-2-3p; 128-3p; 126-3p; 99a-5p; 205-5p; 1-3p; 374a-3p; 30e-5p; 130b-3p; 574-3p; 30c-5p; 98-5p; let-7f-5p; 15a-5p; 34a-5p; 330-5p; 7-5p; 411-5p; 665; 576-5p; 15b-5p; 103a-3p; 7706; 184; 409-3p; 320a-3p; 130a-3p; 27b-3p; 423-5p; 374b-5p; 181b-5p; 584-5p; 23a-3p; 92b-3p; 93-3p; 17-5p; 484; 192-5p; 421; 497-5p; 154-3p; 26a-5p; 708-5p; 503-5p; 195-5p; 23b-3p; 139-5p; 877-5p; 625-3p; 625-5p; 664a-5p; 6511a-3p; 26b-5p; let-7b-5p; 206; 24-3p; 130b-5p; 3925-5p; 671-5p; 454-3p; 329-3p; 889-3p; 99b-5p; 145-5p; 125a-5p; 99b-3p; 493-3p; 30d-5p; 193a-5p; 34c-5p; 9-5p; 301a-3p; 129-5p; 628-5p; 376c-3p; 1307-3p; 769-5p; 204-5p; 296-3p; 125b-1-3p; 378a-3p; 543 |
| Long-term depression | 8.23E-07 | 73 | 100 | 107; 483-5p; 342-3p; 590-3p; 378a-5p; 30a-3p; let-7e-5p; 320c; 320d; 92a-3p; let-7d-5p; 31-5p; let-7a-5p; 197-3p; 320b; 486-3p; 25-3p; 149-5p; 128-3p; 126-3p; 99a-5p; 205-5p; 1-3p; 30e-5p; 130b-3p; 30c-5p; 98-5p; let-7f-5p; 15a-5p; 34a-5p; 330-5p; 7-5p; 665; 576-5p; 15b-5p; 103a-3p; 191-5p; 184; 320a-3p; 130a-3p; 27b-3p; 423-5p; 181b-5p; 584-5p; 23a-3p; 92b-3p; 93-3p; 17-5p; 484; 192-5p; 421; 497-5p; 154-3p; 26a-5p; 503-5p; 675-3p; 195-5p; 941; 501-5p; 23b-3p; 133a-3p; 139-5p; 877-5p; 625-3p; 625-5p; 15b-3p; 26b-5p; 432-5p; let-7b-5p; 206; 24-3p; 130b-5p; 3925-5p; 671-5p; 190a-5p; 454-3p; 329-3p; 889-3p; 99b-5p; 145-5p; 125a-5p; 377-3p; 376b-3p; 140-5p; 30d-5p; 34c-5p; 9-5p; 301a-3p; 129-5p; 628-5p; 376c-3p; 1307-3p; 769-5p; 1271-5p; 204-5p; 296-3p; 125b-1-3p; 500a-5p; 378a-3p; 543 |
| Gap junction | 1.42E-04 | 78 | 99 | 27a-5p; 133a-5p; 107; 483-5p; 342-3p; 590-3p; 30a-3p; let-7e-5p; 320c; 320d; 92a-3p; let-7d-5p; 374a-5p; 31-5p; let-7a-5p; 197-3p; 320b; 22-3p; 486-3p; 25-3p; 128-3p; 126-3p; 99a-5p; 205-5p; 1-3p; 30e-5p; 130b-3p; 574-3p; 30c-5p; 98-5p; let-7f-5p; 15a-5p; 34a-5p; 330-5p; 7-5p; 411-5p; 665; 15b-5p; 103a-3p; 184; 320a-3p; 382-5p; 130a-3p; 27b-3p; 423-5p; 374b-5p; 181b-5p; 584-5p; 23a-3p; 92b-3p; 93-3p; 17-5p; 484; 421; 497-5p; 154-3p; 26a-5p; 708-5p; 195-5p; 501-5p; 23b-3p; 7704; 133a-3p; 139-5p; 877-5p; 625-3p; 625-5p; 26b-5p; 432-5p; let-7b-5p; 206; 24-3p; 130b-5p; 3925-5p; 671-5p; 190a-5p; 454-3p; 329-3p; 889-3p; 145-5p; 125a-5p; 376b-3p; 140-5p; 30d-5p; 491-5p; 34c-5p; 9-5p; 301a-3p; 129-5p; 628-5p; 376c-3p; 1307-3p; 769-5p; 1271-5p; 204-5p; 296-3p; 500a-5p; 378a-3p; 543 |
| Phosphatidylinositol signaling system | 7.68E-04 | 81 | 99 | 107; 342-3p; 590-3p; 378a-5p; 30a-3p; let-7e-5p; 362-5p; 92a-3p; let-7d-5p; 542-3p; 374a-5p; let-7a-5p; 197-3p; 22-3p; 486-3p; 25-3p; 149-5p; 128-3p; 126-3p; 99a-5p; 205-5p; 1306-5p; 1-3p; 374a-3p; 30e-5p; 130b-3p; 30c-5p; 98-5p; let-7f-5p; 15a-5p; 34a-5p; 330-5p; 7-5p; 665; 15b-5p; 103a-3p; 191-5p; 184; 409-3p; 320a-3p; 382-5p; 130a-3p; 27b-3p; 423-5p; 374b-5p; 181b-5p; 23a-3p; 92b-3p; 136-3p; 93-3p; 17-5p; 484; 192-5p; 421; 497-5p; 26a-5p; 328-3p; 503-5p; 195-5p; 941; 501-5p; 23b-3p; 7704; 133a-3p; 139-5p; 877-5p; 127-5p; 625-5p; 26b-5p; let-7b-5p; 206; 24-3p; 130b-5p; 598-3p; 3925-5p; 671-5p; 190a-5p; 454-3p; 329-3p; 889-3p; 99b-5p; 145-5p; 125a-5p; 377-3p; 493-3p; 30d-5p; 193a-5p; 34c-5p; 9-5p; 301a-3p; 129-5p; 1307-3p; 204-5p; 1287-5p; 296-3p; 500a-5p; 378a-3p; 1185-5p; 543 |
| Ribosome | 3.15E-03 | 83 | 99 | 27a-5p; 107; 342-3p; 590-3p; 378a-5p; 1185-1-3p; 30a-3p; let-7e-5p; 92a-3p; let-7d-5p; 542-3p; 100-3p; 374a-5p; 31-5p; let-7a-5p; 197-3p; 320b; 22-3p; 486-3p; 25-3p; 149-5p; 128-3p; 99a-5p; 1306-5p; 379-5p; 374a-3p; 30e-5p; 130b-3p; 30c-5p; 485-3p; 98-5p; let-7f-5p; 15a-5p; 34a-5p; 330-5p; 7-5p; 411-5p; 665; 576-5p; 15b-5p; 103a-3p; let-7a-3p; 191-5p; 151a-3p; 409-3p; 320a-3p; 382-5p; 130a-3p; 27b-3p; 423-5p; 374b-5p; 181b-5p; 584-5p; 23a-3p; 22-5p; 92b-3p; 93-3p; 17-5p; 484; 192-5p; 421; 497-5p; 26a-5p; 328-3p; 503-5p; 195-5p; 941; 501-5p; 23b-3p; 139-5p; 877-5p; 625-3p; 127-5p; 664a-5p; 26b-5p; 432-5p; let-7b-5p; 24-3p; 130b-5p; 671-5p; 454-3p; 99b-5p; 125a-5p; 99b-3p; 140-5p; 493-3p; 30d-5p; 193a-5p; 491-5p; 301a-3p; 129-5p; 1307-3p; 769-5p; 1271-5p; 204-5p; 296-3p; 500a-5p; 378a-3p; 543 |
| Salmonella infection | 2.59E-05 | 75 | 98 | 107; 483-5p; 342-3p; 590-3p; 378a-5p; let-7e-5p; 320c; 320d; 92a-3p; let-7d-5p; 542-3p; 100-3p; 374a-5p; 31-5p; let-7a-5p; 197-3p; 320b; 22-3p; 25-3p; 149-5p; 181a-2-3p; 128-3p; 126-3p; 323b-3p; 205-5p; 1-3p; 323a-3p; 379-5p; 374a-3p; 30e-5p; 130b-3p; 574-3p; 30c-5p; 485-3p; 98-5p; let-7f-5p; 15a-5p; 34a-5p; 7-5p; 411-5p; 665; 576-5p; 15b-5p; 103a-3p; 191-5p; 184; 409-3p; 320a-3p; 382-5p; let-7i-3p; 130a-3p; 27b-3p; 423-5p; 374b-5p; 181b-5p; 584-5p; 23a-3p; 92b-3p; 93-3p; 17-5p; 484; 192-5p; 421; 154-3p; 26a-5p; 195-5p; 941; 23b-3p; 133a-3p; 139-5p; 877-5p; 625-3p; 127-5p; 26b-5p; let-7b-5p; 24-3p; 3925-5p; 337-3p; 454-3p; 329-3p; 99b-5p; 145-5p; 125a-5p; 377-3p; 99b-3p; 493-3p; 30d-5p; 491-5p; 34c-5p; 9-5p; 301a-3p; 129-5p; 1307-3p; 769-5p; 1271-5p; 204-5p; 378a-3p; 543 |
| Hedgehog signaling pathway | 4.93E-05 | 75 | 97 | 107; 483-5p; 342-3p; 590-3p; 378a-5p; 1185-1-3p; let-7e-5p; 92a-3p; let-7d-5p; 542-3p; 374a-5p; 31-5p; let-7a-5p; 197-3p; 22-3p; 149-5p; 181a-2-3p; 128-3p; 126-3p; 99a-5p; 323b-3p; 205-5p; 629-5p; 1-3p; 379-5p; 30e-5p; 130b-3p; 30c-5p; 485-3p; 98-5p; let-7f-5p; 15a-5p; 34a-5p; 330-5p; 7-5p; 411-5p; 665; 15b-5p; 103a-3p; let-7a-3p; 191-5p; 127-3p; 7706; 184; 409-3p; 320a-3p; 382-5p; 130a-3p; 27b-3p; 423-5p; 374b-5p; 181b-5p; 23a-3p; 93-3p; 17-5p; 484; 192-5p; 421; 497-5p; 26a-5p; 708-5p; 503-5p; 675-3p; 195-5p; 501-5p; 23b-3p; 139-5p; 877-5p; 625-5p; 3613-5p; 26b-5p; 432-5p; let-7b-5p; 206; 24-3p; 130b-5p; 454-3p; 329-3p; 889-3p; 125a-5p; 377-3p; 99b-3p; 140-5p; 493-3p; 30d-5p; 491-5p; 34c-5p; 9-5p; 301a-3p; 129-5p; 376c-3p; 1307-3p; 769-5p; 1271-5p; 204-5p; 500a-5p; 378a-3p |
| Glutamatergic synapse | 1.64E-03 | 80 | 97 | 107; 483-5p; 342-3p; 590-3p; 1185-1-3p; 30a-3p; let-7e-5p; 320c; 320d; 92a-3p; let-7d-5p; 100-3p; 374a-5p; 31-5p; let-7a-5p; 197-3p; 320b; 22-3p; 25-3p; 149-5p; 181a-2-3p; 128-3p; 126-3p; 99a-5p; 205-5p; 1306-5p; 1-3p; 374a-3p; 30e-5p; 130b-3p; 30c-5p; 485-3p; 98-5p; let-7f-5p; 15a-5p; 34a-5p; 7-5p; 411-5p; 665; 15b-5p; 103a-3p; 127-3p; 151a-3p; 184; 409-3p; 320a-3p; 130a-3p; 27b-3p; 423-5p; 374b-5p; 181b-5p; 584-5p; 23a-3p; 92b-3p; 93-3p; 17-5p; 484; 192-5p; 421; 497-5p; 154-3p; 26a-5p; 195-5p; 501-5p; 23b-3p; 133a-3p; 877-5p; 625-5p; 26b-5p; 432-5p; let-7b-5p; 24-3p; 130b-5p; 3925-5p; 454-3p; 329-3p; 889-3p; 99b-5p; 145-5p; 125a-5p; 376b-3p; 493-3p; 30d-5p; 296-5p; 491-5p; 34c-5p; 9-5p; 301a-3p; 129-5p; 1307-3p; 769-5p; 204-5p; 1287-5p; 296-3p; 500a-5p; 378a-3p; 1185-5p |
| Long-term potentiation | 7.01E-06 | 71 | 96 | 483-5p; 342-3p; 590-3p; 378a-5p; 30a-3p; let-7e-5p; 320c; 320d; 92a-3p; 100-3p; 374a-5p; let-7a-5p; 197-3p; 320b; 22-3p; 486-3p; 25-3p; 149-5p; 181a-2-3p; 128-3p; 126-3p; 99a-5p; 1-3p; 323a-3p; 30e-5p; 130b-3p; 574-3p; 30c-5p; 98-5p; let-7f-5p; 15a-5p; 34a-5p; 330-5p; 7-5p; 411-5p; 15b-5p; 103a-3p; 191-5p; 184; 409-3p; 320a-3p; 382-5p; 130a-3p; 27b-3p; 423-5p; 374b-5p; 628-3p; 181b-5p; 584-5p; 92b-3p; 93-3p; 17-5p; 484; 192-5p; 497-5p; 154-3p; 26a-5p; 503-5p; 195-5p; 501-5p; 23b-3p; 133a-3p; 139-5p; 877-5p; 625-5p; 26b-5p; let-7b-5p; 206; 24-3p; 130b-5p; 598-3p; 3925-5p; 337-3p; 671-5p; 190a-5p; 454-3p; 329-3p; 889-3p; 99b-5p; 145-5p; 125a-5p; 140-5p; 493-3p; 30d-5p; 34c-5p; 9-5p; 301a-3p; 129-5p; 1307-3p; 769-5p; 204-5p; 296-3p; 500a-5p; 378a-3p; 1185-5p; 543 |
| Th1 and Th2 cell differentiation | 2.98E-05 | 73 | 96 | 133a-5p; 107; 483-5p; 342-3p; 590-3p; 30a-3p; 320c; 320d; 92a-3p; let-7d-5p; let-7a-5p; 197-3p; 320b; 486-3p; 25-3p; 149-5p; 181a-2-3p; 128-3p; 126-3p; 323b-3p; 205-5p; 1-3p; 30e-5p; 130b-3p; 30c-5p; 485-3p; 98-5p; let-7f-5p; 15a-5p; 34a-5p; 7-5p; 665; 576-5p; 15b-5p; 103a-3p; 191-5p; 151a-3p; 184; 409-3p; 320a-3p; 130a-3p; 27b-3p; 423-5p; 181b-5p; 6724-5p; 584-5p; 23a-3p; 22-5p; 92b-3p; 136-3p; 93-3p; 17-5p; 484; 192-5p; 421; 497-5p; 154-3p; 26a-5p; 708-5p; 503-5p; 195-5p; 501-5p; 23b-3p; 7704; 133a-3p; 139-5p; 877-5p; 625-5p; 6511a-3p; 26b-5p; let-7b-5p; 206; 24-3p; 130b-5p; 337-3p; 454-3p; 329-3p; 145-5p; 125a-5p; 140-5p; 493-3p; 30d-5p; 296-5p; 491-5p; 34c-5p; 9-5p; 301a-3p; 129-5p; 1307-3p; 769-5p; 1271-5p; 204-5p; 296-3p; 500a-5p; 378a-3p; 543 |
| Systemic lupus erythematosus | 3.76E-05 | 73 | 96 | 133a-5p; 483-5p; 342-3p; let-7d-3p; 590-3p; 378a-5p; 30a-3p; let-7e-5p; 320c; 92a-3p; let-7d-5p; 100-3p; 374a-5p; 31-5p; let-7a-5p; 197-3p; 320b; 22-3p; 25-3p; 149-5p; 128-3p; 126-3p; 1306-5p; 629-5p; 1-3p; 374a-3p; 130b-3p; 98-5p; let-7f-5p; 15a-5p; 34a-5p; 330-5p; 411-5p; 665; 576-5p; 15b-5p; 103a-3p; 409-3p; 320a-3p; 382-5p; 130a-3p; 27b-3p; 423-5p; 374b-5p; 181b-5p; 584-5p; 23a-3p; 22-5p; 92b-3p; 93-3p; 17-5p; 484; 192-5p; 421; 497-5p; 26a-5p; 328-3p; 503-5p; 195-5p; 501-5p; 23b-3p; 7704; 133a-3p; 877-5p; 127-5p; 625-5p; 299-5p; 3613-5p; 6511a-3p; 26b-5p; let-7b-5p; 206; 24-3p; 130b-5p; 598-3p; 3925-5p; 337-3p; 671-5p; 454-3p; 329-3p; 99b-5p; 145-5p; 125a-5p; 99b-3p; 376b-3p; 9-5p; 301a-3p; 129-5p; 1307-3p; 769-5p; 204-5p; 1287-5p; 296-3p; 500a-5p; 378a-3p; 1185-5p |
| Parkinson disease | 1.29E-03 | 78 | 95 | 107; 483-5p; 590-3p; 378a-5p; 30a-3p; let-7e-5p; 320c; 320d; 92a-3p; let-7d-5p; 374a-5p; 31-5p; let-7a-5p; 197-3p; 320b; 22-3p; 486-3p; 25-3p; 149-5p; 128-3p; 99a-5p; 205-5p; 1306-5p; 629-5p; 1-3p; 379-5p; 30e-5p; 130b-3p; 30c-5p; 98-5p; let-7f-5p; 15a-5p; 34a-5p; 7-5p; 411-5p; 665; 576-5p; 15b-5p; 103a-3p; 409-3p; 320a-3p; 382-5p; 130a-3p; 27b-3p; 423-5p; 374b-5p; 628-3p; 181b-5p; 23a-3p; 92b-3p; 93-3p; 17-5p; 484; 192-5p; 421; 497-5p; 26a-5p; 328-3p; 708-5p; 503-5p; 675-3p; 195-5p; 23b-3p; 133a-3p; 139-5p; 877-5p; 625-5p; 26b-5p; let-7b-5p; 206; 24-3p; 130b-5p; 671-5p; 454-3p; 329-3p; 889-3p; 99b-5p; 145-5p; 125a-5p; 377-3p; 99b-3p; 376b-3p; 140-5p; 30d-5p; 491-5p; 34c-5p; 9-5p; 301a-3p; 129-5p; 769-5p; 204-5p; 1287-5p; 296-3p; 1185-5p; 543 |
| Circadian entrainment | 1.51E-02 | 82 | 95 | 107; 483-5p; 590-3p; 1185-1-3p; let-7e-5p; 320c; 320d; 92a-3p; let-7d-5p; 542-3p; 100-3p; let-7a-5p; 197-3p; 320b; 22-3p; 486-3p; 25-3p; 181a-2-3p; 128-3p; 126-3p; 99a-5p; 323b-3p; 205-5p; 1-3p; 374a-3p; 30e-5p; 130b-3p; 30c-5p; 485-3p; 98-5p; let-7f-5p; 15a-5p; 34a-5p; 7-5p; 411-5p; 665; 576-5p; 15b-5p; 103a-3p; let-7a-3p; 151a-3p; 184; 320a-3p; 382-5p; 130a-3p; 27b-3p; 423-5p; 181b-5p; 584-5p; 23a-3p; 92b-3p; 17-5p; 484; 192-5p; 497-5p; 154-3p; 708-5p; 195-5p; 501-5p; 23b-3p; 133a-3p; 139-5p; 877-5p; 625-3p; 625-5p; 26b-5p; 432-5p; let-7b-5p; 206; 24-3p; 130b-5p; 598-3p; 3925-5p; 190a-5p; 454-3p; 329-3p; 889-3p; 377-3p; 376b-3p; 493-3p; 30d-5p; 296-5p; 34c-5p; 9-5p; 301a-3p; 129-5p; 376c-3p; 1307-3p; 769-5p; 204-5p; 1287-5p; 500a-5p; 378a-3p; 1185-5p; 543 |
| Lysosome | 3.62E-04 | 75 | 94 | 107; 342-3p; 590-3p; 378a-5p; 30a-3p; let-7e-5p; 320c; 320d; 92a-3p; let-7d-5p; 542-3p; 374a-5p; 31-5p; let-7a-5p; 197-3p; 320b; 486-3p; 25-3p; 149-5p; 128-3p; 205-5p; 1306-5p; 1-3p; 323a-3p; 30e-5p; 130b-3p; 574-3p; 30c-5p; 485-3p; 98-5p; let-7f-5p; 15a-5p; 34a-5p; 330-5p; 7-5p; 411-5p; 665; 15b-5p; 103a-3p; 191-5p; 184; 320a-3p; 382-5p; 130a-3p; 27b-3p; 423-5p; 374b-5p; 181b-5p; 23a-3p; 22-5p; 92b-3p; 17-5p; 484; 192-5p; 421; 497-5p; 26a-5p; 503-5p; 195-5p; 941; 501-5p; 23b-3p; 877-5p; 127-5p; 625-5p; 664a-5p; 26b-5p; let-7b-5p; 24-3p; 671-5p; 454-3p; 329-3p; 889-3p; 145-5p; 125a-5p; 377-3p; 99b-3p; 376b-3p; 140-5p; 30d-5p; 491-5p; 34c-5p; 9-5p; 301a-3p; 129-5p; 1307-3p; 769-5p; 1271-5p; 204-5p; 1287-5p; 296-3p; 378a-3p; 1185-5p; 543 |
| Thyroid cancer | 7.14E-04 | 76 | 94 | 483-5p; 342-3p; 378a-5p; 1185-1-3p; 30a-3p; let-7e-5p; 92a-3p; let-7d-5p; 542-3p; 100-3p; 374a-5p; let-7a-5p; 197-3p; 320b; 22-3p; 486-3p; 25-3p; 128-3p; 126-3p; 99a-5p; 323b-3p; 1-3p; 323a-3p; 30e-5p; 130b-3p; 574-3p; 30c-5p; 485-3p; 98-5p; let-7f-5p; 15a-5p; 34a-5p; 330-5p; 7-5p; 665; 576-5p; 15b-5p; let-7a-3p; 191-5p; 7706; 184; 320a-3p; 130a-3p; 27b-3p; 423-5p; 374b-5p; 181b-5p; 584-5p; 23a-3p; 92b-3p; 93-3p; 17-5p; 484; 192-5p; 421; 497-5p; 154-3p; 26a-5p; 708-5p; 503-5p; 195-5p; 23b-3p; 7704; 133a-3p; 139-5p; 877-5p; 625-5p; 299-5p; 26b-5p; let-7b-5p; 206; 24-3p; 3925-5p; 671-5p; 454-3p; 329-3p; 889-3p; 145-5p; 125a-5p; 377-3p; 30d-5p; 491-5p; 34c-5p; 9-5p; 301a-3p; 129-5p; 1307-3p; 769-5p; 1271-5p; 204-5p; 296-3p; 125b-1-3p; 378a-3p; 543 |
| Antigen processing and presentation | 3.04E-06 | 67 | 93 | 342-3p; 590-3p; 378a-5p; 1185-1-3p; 30a-3p; let-7e-5p; 362-5p; 92a-3p; let-7d-5p; 374a-5p; 31-5p; 197-3p; 22-3p; 486-3p; 25-3p; 149-5p; 128-3p; 205-5p; 1306-5p; 629-5p; 1-3p; 323a-3p; 379-5p; 374a-3p; 30e-5p; 130b-3p; 30c-5p; 485-3p; 15a-5p; 34a-5p; 330-5p; 7-5p; 411-5p; 665; 576-5p; 15b-5p; 103a-3p; let-7a-3p; 151a-3p; 409-3p; 320a-3p; 130a-3p; 27b-3p; 423-5p; 374b-5p; 181b-5p; 584-5p; 23a-3p; 22-5p; 92b-3p; 136-3p; 93-3p; 17-5p; 484; 192-5p; 421; 497-5p; 26a-5p; 328-3p; 503-5p; 195-5p; 941; 501-5p; 7704; 139-5p; 877-5p; 625-3p; 625-5p; 664a-5p; 6511a-3p; 26b-5p; let-7b-5p; 24-3p; 130b-5p; 454-3p; 329-3p; 889-3p; 125a-5p; 99b-3p; 493-3p; 30d-5p; 491-5p; 34c-5p; 9-5p; 301a-3p; 129-5p; 628-5p; 769-5p; 1271-5p; 204-5p; 296-3p; 500a-5p; 378a-3p |
| Aldosterone synthesis and secretion | 2.08E-04 | 73 | 93 | 107; 342-3p; let-7d-3p; 590-3p; 1185-1-3p; 30a-3p; 92a-3p; let-7d-5p; 542-3p; 374a-5p; 31-5p; let-7a-5p; 197-3p; 320b; 22-3p; 486-3p; 25-3p; 149-5p; 181a-2-3p; 128-3p; 99a-5p; 323b-3p; 205-5p; 1-3p; 323a-3p; 30e-5p; 130b-3p; 30c-5p; 98-5p; let-7f-5p; 15a-5p; 34a-5p; 7-5p; 411-5p; 665; 576-5p; 15b-5p; 103a-3p; let-7a-3p; 127-3p; 184; 320a-3p; 382-5p; 130a-3p; 27b-3p; 423-5p; 374b-5p; 181b-5p; 23a-3p; 92b-3p; 93-3p; 17-5p; 484; 192-5p; 26a-5p; 195-5p; 941; 501-5p; 23b-3p; 7704; 133a-3p; 877-5p; 625-5p; 664a-5p; 299-5p; 26b-5p; 432-5p; let-7b-5p; 206; 24-3p; 130b-5p; 598-3p; 3925-5p; 671-5p; 190a-5p; 454-3p; 329-3p; 145-5p; 125a-5p; 377-3p; 376b-3p; 140-5p; 493-3p; 30d-5p; 34c-5p; 9-5p; 301a-3p; 129-5p; 376c-3p; 1307-3p; 204-5p; 500a-5p; 378a-3p |
| Inflammatory mediator regulation of TRP channels | 4.66E-04 | 74 | 93 | 107; 342-3p; 590-3p; let-7e-5p; 92a-3p; let-7d-5p; 542-3p; 100-3p; 374a-5p; 31-5p; let-7a-5p; 197-3p; 320b; 22-3p; 486-3p; 25-3p; 149-5p; 181a-2-3p; 128-3p; 126-3p; 99a-5p; 205-5p; 1-3p; 323a-3p; 379-5p; 30e-5p; 130b-3p; 30c-5p; 98-5p; let-7f-5p; 15a-5p; 34a-5p; 7-5p; 411-5p; 665; 576-5p; 15b-5p; 103a-3p; 184; 320a-3p; 382-5p; 130a-3p; 27b-3p; 423-5p; 374b-5p; 181b-5p; 23a-3p; 92b-3p; 17-5p; 484; 192-5p; 421; 497-5p; 26a-5p; 708-5p; 503-5p; 195-5p; 501-5p; 23b-3p; 133a-3p; 139-5p; 877-5p; 625-3p; 625-5p; 6511a-3p; 26b-5p; 432-5p; let-7b-5p; 206; 24-3p; 130b-5p; 598-3p; 3925-5p; 337-3p; 190a-5p; 454-3p; 329-3p; 99b-5p; 145-5p; 125a-5p; 376b-3p; 493-3p; 30d-5p; 193a-5p; 34c-5p; 9-5p; 301a-3p; 129-5p; 376c-3p; 769-5p; 204-5p; 296-3p; 500a-5p |
| Hypertrophic cardiomyopathy HCM | 2.22E-03 | 77 | 93 | 107; 483-5p; 342-3p; 590-3p; 378a-5p; 30a-3p; let-7e-5p; 92a-3p; let-7d-5p; 31-5p; let-7a-5p; 197-3p; 486-3p; 25-3p; 149-5p; 128-3p; 99a-5p; 205-5p; 629-5p; 1-3p; 379-5p; 30e-5p; 130b-3p; 574-3p; 30c-5p; 98-5p; let-7f-5p; 15a-5p; 34a-5p; 330-5p; 7-5p; 665; 576-5p; 15b-5p; 103a-3p; let-7a-3p; 409-3p; 320a-3p; let-7i-3p; 130a-3p; 423-5p; 23a-3p; 92b-3p; 136-3p; 93-3p; 17-5p; 484; 192-5p; 421; 497-5p; 154-3p; 26a-5p; 675-3p; 195-5p; 501-5p; 23b-3p; 133a-3p; 139-5p; 877-5p; 127-5p; 625-5p; 299-5p; 3613-5p; 26b-5p; 432-5p; let-7b-5p; 24-3p; 130b-5p; 3925-5p; 671-5p; 190a-5p; 454-3p; 329-3p; 99b-5p; 145-5p; 125a-5p; 99b-3p; 376b-3p; 493-3p; 30d-5p; 34c-5p; 9-5p; 301a-3p; 129-5p; 1307-3p; 769-5p; 204-5p; 1287-5p; 296-3p; 125b-1-3p; 500a-5p; 378a-3p; 1185-5p |
| Viral myocarditis | 1.47E-05 | 68 | 92 | 107; 342-3p; 590-3p; 378a-5p; 30a-3p; let-7e-5p; 362-5p; 320c; 92a-3p; let-7d-5p; 542-3p; 374a-5p; 31-5p; let-7a-5p; 197-3p; 22-3p; 25-3p; 149-5p; 128-3p; 323b-3p; 1306-5p; 629-5p; 1-3p; 374a-3p; 30e-5p; 574-3p; 30c-5p; 98-5p; let-7f-5p; 15a-5p; 34a-5p; 330-5p; 7-5p; 411-5p; 665; 576-5p; 15b-5p; 103a-3p; let-7a-3p; 7706; 409-3p; 320a-3p; 382-5p; let-7i-3p; 27b-3p; 423-5p; 374b-5p; 584-5p; 22-5p; 92b-3p; 93-3p; 17-5p; 484; 192-5p; 421; 497-5p; 26a-5p; 708-5p; 503-5p; 195-5p; 7704; 133a-3p; 139-5p; 877-5p; 127-5p; 625-5p; 26b-5p; let-7b-5p; 206; 24-3p; 130b-5p; 3925-5p; 329-3p; 99b-5p; 145-5p; 125a-5p; 99b-3p; 376b-3p; 493-3p; 30d-5p; 296-5p; 491-5p; 34c-5p; 9-5p; 129-5p; 628-5p; 1307-3p; 1271-5p; 204-5p; 296-3p; 378a-3p; 543 |
| VEGF signaling pathway | 1.05E-04 | 71 | 92 | 27a-5p; 107; 483-5p; 342-3p; 590-3p; 30a-3p; let-7e-5p; 92a-3p; let-7d-5p; 542-3p; 374a-5p; 31-5p; let-7a-5p; 197-3p; 320b; 22-3p; 486-3p; 25-3p; 149-5p; 181a-2-3p; 128-3p; 126-3p; 99a-5p; 205-5p; 1-3p; 379-5p; 374a-3p; 30e-5p; 130b-3p; 574-3p; 30c-5p; 98-5p; let-7f-5p; 15a-5p; 34a-5p; 330-5p; 7-5p; 665; 576-5p; 15b-5p; 103a-3p; 184; 409-3p; 320a-3p; 130a-3p; 27b-3p; 374b-5p; 181b-5p; 584-5p; 23a-3p; 92b-3p; 93-3p; 17-5p; 484; 192-5p; 497-5p; 154-3p; 26a-5p; 708-5p; 503-5p; 195-5p; 23b-3p; 133a-3p; 139-5p; 877-5p; 625-3p; 26b-5p; let-7b-5p; 206; 24-3p; 130b-5p; 3925-5p; 671-5p; 454-3p; 329-3p; 889-3p; 99b-5p; 145-5p; 125a-5p; 140-5p; 30d-5p; 296-5p; 193a-5p; 34c-5p; 301a-3p; 129-5p; 1307-3p; 769-5p; 204-5p; 296-3p; 378a-3p; 543 |
| Morphine addiction | 7.23E-04 | 74 | 92 | 107; 483-5p; let-7d-3p; 590-3p; 1185-1-3p; 30a-3p; let-7e-5p; 320c; 320d; 92a-3p; let-7d-5p; 542-3p; 100-3p; 374a-5p; let-7a-5p; 320b; 22-3p; 149-5p; 128-3p; 99a-5p; 205-5p; 1306-5p; 1-3p; 374a-3p; 30e-5p; 130b-3p; 30c-5p; 98-5p; let-7f-5p; 15a-5p; 330-5p; 7-5p; 411-5p; 665; 15b-5p; 103a-3p; let-7a-3p; 127-3p; 151a-3p; 184; 409-3p; 320a-3p; 382-5p; 27b-3p; 423-5p; 374b-5p; 181b-5p; 584-5p; 23a-3p; 22-5p; 92b-3p; 136-3p; 17-5p; 484; 192-5p; 421; 497-5p; 26a-5p; 195-5p; 941; 23b-3p; 133a-3p; 139-5p; 877-5p; 625-5p; 664a-5p; 15b-3p; 6511a-3p; 26b-5p; 432-5p; let-7b-5p; 24-3p; 130b-5p; 3925-5p; 337-3p; 329-3p; 889-3p; 377-3p; 376b-3p; 493-3p; 30d-5p; 296-5p; 491-5p; 34c-5p; 129-5p; 1307-3p; 769-5p; 1271-5p; 204-5p; 1287-5p; 500a-5p; 378a-3p |
| Dilated cardiomyopathy DCM | 8.10E-03 | 77 | 91 | 107; 483-5p; 342-3p; 590-3p; 378a-5p; 30a-3p; let-7e-5p; 92a-3p; let-7d-5p; 31-5p; let-7a-5p; 197-3p; 320b; 22-3p; 486-3p; 25-3p; 149-5p; 128-3p; 99a-5p; 205-5p; 629-5p; 1-3p; 30e-5p; 130b-3p; 574-3p; 30c-5p; 98-5p; let-7f-5p; 15a-5p; 34a-5p; 330-5p; 7-5p; 665; 576-5p; 15b-5p; 103a-3p; let-7a-3p; 409-3p; 320a-3p; let-7i-3p; 130a-3p; 423-5p; 181b-5p; 23a-3p; 92b-3p; 136-3p; 93-3p; 17-5p; 484; 192-5p; 421; 497-5p; 154-3p; 26a-5p; 195-5p; 501-5p; 23b-3p; 133a-3p; 139-5p; 877-5p; 127-5p; 625-5p; 299-5p; 26b-5p; 432-5p; let-7b-5p; 24-3p; 130b-5p; 3925-5p; 671-5p; 190a-5p; 329-3p; 99b-5p; 145-5p; 125a-5p; 99b-3p; 376b-3p; 493-3p; 30d-5p; 34c-5p; 9-5p; 129-5p; 1307-3p; 769-5p; 204-5p; 1287-5p; 296-3p; 125b-1-3p; 500a-5p; 378a-3p; 1185-5p |
| Pertussis | 5.33E-03 | 75 | 90 | 133a-5p; 107; 483-5p; 342-3p; 590-3p; 378a-5p; 30a-3p; let-7e-5p; 320c; 320d; 92a-3p; let-7d-5p; 100-3p; 374a-5p; 31-5p; let-7a-5p; 197-3p; 320b; 486-3p; 25-3p; 149-5p; 181a-2-3p; 128-3p; 323b-3p; 205-5p; 1306-5p; 1-3p; 30e-5p; 130b-3p; 30c-5p; 98-5p; let-7f-5p; 15a-5p; 34a-5p; 330-5p; 7-5p; 665; 15b-5p; 103a-3p; 191-5p; 320a-3p; 382-5p; 130a-3p; 27b-3p; 423-5p; 374b-5p; 181b-5p; 584-5p; 23a-3p; 92b-3p; 93-3p; 17-5p; 484; 192-5p; 421; 154-3p; 26a-5p; 328-3p; 708-5p; 23b-3p; 139-5p; 877-5p; 127-5p; 625-5p; 6511a-3p; 26b-5p; let-7b-5p; 206; 24-3p; 598-3p; 3925-5p; 337-3p; 190a-5p; 454-3p; 329-3p; 145-5p; 125a-5p; 493-3p; 30d-5p; 491-5p; 9-5p; 301a-3p; 129-5p; 628-5p; 1307-3p; 769-5p; 204-5p; 296-3p; 378a-3p; 543 |
| mRNA surveillance pathway | 1.84E-02 | 78 | 90 | 107; 483-5p; 590-3p; 378a-5p; 1185-1-3p; 30a-3p; let-7e-5p; 320c; 320d; 92a-3p; let-7d-5p; 100-3p; 31-5p; let-7a-5p; 197-3p; 320b; 486-3p; 25-3p; 149-5p; 128-3p; 99a-5p; 205-5p; 1306-5p; 1-3p; 323a-3p; 379-5p; 30e-5p; 130b-3p; 30c-5p; 98-5p; let-7f-5p; 15a-5p; 34a-5p; 7-5p; 665; 576-5p; 15b-5p; 103a-3p; let-7a-3p; 409-3p; 320a-3p; let-7i-3p; 27b-3p; 423-5p; 181b-5p; 584-5p; 23a-3p; 92b-3p; 93-3p; 17-5p; 484; 192-5p; 421; 497-5p; 26a-5p; 708-5p; 503-5p; 195-5p; 941; 23b-3p; 877-5p; 625-5p; 26b-5p; let-7b-5p; 206; 24-3p; 130b-5p; 3925-5p; 337-3p; 671-5p; 190a-5p; 329-3p; 889-3p; 99b-5p; 125a-5p; 377-3p; 99b-3p; 493-3p; 30d-5p; 193a-5p; 491-5p; 34c-5p; 129-5p; 628-5p; 1307-3p; 769-5p; 1271-5p; 204-5p; 378a-3p; 1185-5p |
| GnRH secretion | 2.50E-06 | 63 | 89 | 27a-5p; 107; 483-5p; 590-3p; 378a-5p; 30a-3p; 362-5p; 92a-3p; 542-3p; let-7a-5p; 197-3p; 320b; 22-3p; 486-3p; 25-3p; 149-5p; 181a-2-3p; 128-3p; 126-3p; 99a-5p; 1306-5p; 1-3p; 374a-3p; 130b-3p; 30c-5p; 98-5p; 15a-5p; 34a-5p; 330-5p; 7-5p; 15b-5p; 103a-3p; 151a-3p; 184; 409-3p; 320a-3p; 130a-3p; 27b-3p; 423-5p; 374b-5p; 181b-5p; 584-5p; 23a-3p; 92b-3p; 93-3p; 17-5p; 192-5p; 421; 497-5p; 154-3p; 26a-5p; 708-5p; 503-5p; 195-5p; 501-5p; 23b-3p; 133a-3p; 139-5p; 877-5p; 625-3p; 127-5p; 299-5p; 26b-5p; let-7b-5p; 206; 24-3p; 130b-5p; 3925-5p; 671-5p; 454-3p; 329-3p; 889-3p; 145-5p; 125a-5p; 140-5p; 30d-5p; 193a-5p; 491-5p; 34c-5p; 9-5p; 301a-3p; 129-5p; 1307-3p; 769-5p; 204-5p; 296-3p; 500a-5p; 378a-3p; 543 |
| Amyotrophic lateral sclerosis ALS | 8.13E-06 | 64 | 89 | 483-5p; 590-3p; 378a-5p; 30a-3p; let-7e-5p; 320c; 92a-3p; let-7d-5p; 542-3p; 374a-5p; 31-5p; let-7a-5p; 197-3p; 22-3p; 25-3p; 149-5p; 181a-2-3p; 128-3p; 126-3p; 205-5p; 1306-5p; 1-3p; 374a-3p; 30e-5p; 130b-3p; 574-3p; 30c-5p; 98-5p; let-7f-5p; 15a-5p; 34a-5p; 330-5p; 7-5p; 411-5p; 665; 576-5p; 15b-5p; 103a-3p; 184; 409-3p; 320a-3p; 382-5p; 130a-3p; 27b-3p; 374b-5p; 181b-5p; 23a-3p; 92b-3p; 93-3p; 17-5p; 484; 192-5p; 421; 497-5p; 26a-5p; 328-3p; 708-5p; 503-5p; 195-5p; 501-5p; 23b-3p; 133a-3p; 139-5p; 625-3p; 26b-5p; let-7b-5p; 206; 24-3p; 130b-5p; 3925-5p; 337-3p; 454-3p; 329-3p; 145-5p; 125a-5p; 377-3p; 30d-5p; 491-5p; 34c-5p; 9-5p; 301a-3p; 129-5p; 376c-3p; 1307-3p; 204-5p; 125b-1-3p; 500a-5p; 378a-3p; 1185-5p |
| Inositol phosphate metabolism | 9.81E-05 | 68 | 89 | 107; 483-5p; 590-3p; 378a-5p; let-7e-5p; 362-5p; 92a-3p; let-7d-5p; 542-3p; 374a-5p; let-7a-5p; 197-3p; 320b; 22-3p; 25-3p; 149-5p; 128-3p; 126-3p; 99a-5p; 205-5p; 1306-5p; 1-3p; 374a-3p; 30e-5p; 130b-3p; 30c-5p; 98-5p; let-7f-5p; 15a-5p; 34a-5p; 7-5p; 411-5p; 665; 15b-5p; 103a-3p; 191-5p; 127-3p; 184; 409-3p; 320a-3p; 382-5p; 130a-3p; 27b-3p; 423-5p; 374b-5p; 181b-5p; 23a-3p; 22-5p; 92b-3p; 93-3p; 17-5p; 484; 192-5p; 421; 497-5p; 26a-5p; 328-3p; 503-5p; 195-5p; 941; 501-5p; 23b-3p; 133a-3p; 139-5p; 877-5p; 127-5p; 664a-5p; 26b-5p; let-7b-5p; 24-3p; 130b-5p; 671-5p; 454-3p; 329-3p; 99b-5p; 145-5p; 125a-5p; 377-3p; 30d-5p; 9-5p; 301a-3p; 129-5p; 1307-3p; 204-5p; 1287-5p; 500a-5p; 378a-3p; 1185-5p; 543 |
| Glycolysis Gluconeogenesis | 3.89E-06 | 63 | 88 | 107; 483-5p; 378a-5p; 1185-1-3p; 30a-3p; let-7e-5p; 320c; 92a-3p; let-7d-5p; 542-3p; 374a-5p; let-7a-5p; 197-3p; 320b; 22-3p; 486-3p; 25-3p; 149-5p; 181a-2-3p; 128-3p; 99a-5p; 1306-5p; 1-3p; 374a-3p; 130b-3p; 30c-5p; 485-3p; 98-5p; let-7f-5p; 15a-5p; 34a-5p; 330-5p; 7-5p; 665; 576-5p; 15b-5p; 103a-3p; let-7a-3p; 191-5p; 184; 409-3p; 320a-3p; 130a-3p; 27b-3p; 423-5p; 374b-5p; 181b-5p; 23a-3p; 22-5p; 92b-3p; 136-3p; 93-3p; 17-5p; 484; 192-5p; 497-5p; 26a-5p; 328-3p; 195-5p; 501-5p; 23b-3p; 133a-3p; 139-5p; 625-5p; 26b-5p; let-7b-5p; 24-3p; 130b-5p; 671-5p; 190a-5p; 454-3p; 329-3p; 99b-5p; 145-5p; 125a-5p; 377-3p; 493-3p; 491-5p; 34c-5p; 9-5p; 301a-3p; 129-5p; 376c-3p; 1307-3p; 769-5p; 204-5p; 296-3p; 378a-3p |
| Legionellosis | 6.57E-05 | 66 | 88 | 107; 342-3p; 378a-5p; 30a-3p; let-7e-5p; 362-5p; 320c; 320d; 92a-3p; let-7d-5p; 542-3p; 100-3p; 374a-5p; 31-5p; let-7a-5p; 197-3p; 320b; 22-3p; 25-3p; 149-5p; 128-3p; 126-3p; 205-5p; 1306-5p; 1-3p; 30e-5p; 130b-3p; 30c-5p; 98-5p; let-7f-5p; 15a-5p; 34a-5p; 330-5p; 7-5p; 665; 15b-5p; 103a-3p; 320a-3p; 382-5p; 130a-3p; 27b-3p; 423-5p; 181b-5p; 23a-3p; 92b-3p; 93-3p; 17-5p; 484; 192-5p; 421; 497-5p; 26a-5p; 328-3p; 503-5p; 195-5p; 941; 501-5p; 23b-3p; 133a-3p; 139-5p; 877-5p; 625-3p; 127-5p; 6511a-3p; 26b-5p; let-7b-5p; 24-3p; 130b-5p; 3925-5p; 337-3p; 671-5p; 190a-5p; 454-3p; 329-3p; 145-5p; 125a-5p; 376b-3p; 30d-5p; 296-5p; 34c-5p; 9-5p; 301a-3p; 129-5p; 376c-3p; 1307-3p; 204-5p; 296-3p; 378a-3p |
| Epithelial cell signaling in Helicobacter pylori infection | 1.59E-04 | 67 | 88 | 27a-5p; 133a-5p; 107; 342-3p; 590-3p; 1185-1-3p; 30a-3p; let-7e-5p; 92a-3p; let-7d-5p; 542-3p; 100-3p; 374a-5p; 31-5p; let-7a-5p; 22-3p; 486-3p; 25-3p; 149-5p; 181a-2-3p; 128-3p; 126-3p; 323b-3p; 205-5p; 1-3p; 374a-3p; 30e-5p; 130b-3p; 574-3p; 30c-5p; 98-5p; let-7f-5p; 15a-5p; 34a-5p; 7-5p; 411-5p; 665; 576-5p; 15b-5p; 103a-3p; 151a-3p; 409-3p; 320a-3p; 382-5p; 130a-3p; 27b-3p; 423-5p; 374b-5p; 181b-5p; 23a-3p; 92b-3p; 93-3p; 17-5p; 484; 192-5p; 421; 497-5p; 26a-5p; 708-5p; 503-5p; 195-5p; 23b-3p; 133a-3p; 139-5p; 877-5p; 625-5p; 299-5p; 3613-5p; 26b-5p; let-7b-5p; 206; 24-3p; 130b-5p; 671-5p; 454-3p; 329-3p; 145-5p; 125a-5p; 377-3p; 140-5p; 30d-5p; 491-5p; 34c-5p; 9-5p; 301a-3p; 129-5p; 628-5p; 204-5p |
| Adipocytokine signaling pathway | 1.05E-03 | 70 | 88 | 27a-5p; 342-3p; 590-3p; 378a-5p; 1185-1-3p; let-7e-5p; 92a-3p; let-7d-5p; 542-3p; 374a-5p; let-7a-5p; 197-3p; 22-3p; 486-3p; 149-5p; 128-3p; 126-3p; 99a-5p; 205-5p; 1-3p; 323a-3p; 374a-3p; 30e-5p; 130b-3p; 574-3p; 30c-5p; 485-3p; 98-5p; let-7f-5p; 15a-5p; 34a-5p; 330-5p; 7-5p; 665; 15b-5p; 103a-3p; 191-5p; 151a-3p; 184; 409-3p; 320a-3p; 130a-3p; 27b-3p; 374b-5p; 181b-5p; 23a-3p; 136-3p; 93-3p; 17-5p; 484; 192-5p; 421; 497-5p; 154-3p; 26a-5p; 708-5p; 503-5p; 675-3p; 195-5p; 501-5p; 23b-3p; 139-5p; 877-5p; 625-3p; 127-5p; 3613-5p; 26b-5p; 432-5p; let-7b-5p; 206; 24-3p; 130b-5p; 337-3p; 454-3p; 329-3p; 99b-5p; 145-5p; 125a-5p; 30d-5p; 193a-5p; 9-5p; 301a-3p; 129-5p; 628-5p; 1307-3p; 204-5p; 500a-5p; 378a-3p |
| Gastric acid secretion | 2.79E-03 | 72 | 88 | 107; 590-3p; 378a-5p; 1185-1-3p; 320c; 320d; 92a-3p; let-7d-5p; 374a-5p; let-7a-5p; 197-3p; 320b; 22-3p; 486-3p; 25-3p; 149-5p; 181a-2-3p; 128-3p; 126-3p; 99a-5p; 205-5p; 1-3p; 30e-5p; 130b-3p; 574-3p; 30c-5p; 98-5p; let-7f-5p; 15a-5p; 34a-5p; 7-5p; 411-5p; 665; 15b-5p; 103a-3p; 184; 409-3p; 320a-3p; 382-5p; let-7i-3p; 130a-3p; 27b-3p; 423-5p; 374b-5p; 181b-5p; 23a-3p; 92b-3p; 136-3p; 93-3p; 17-5p; 484; 192-5p; 26a-5p; 328-3p; 195-5p; 941; 501-5p; 23b-3p; 133a-3p; 877-5p; 127-5p; 625-5p; 26b-5p; 432-5p; let-7b-5p; 206; 24-3p; 130b-5p; 598-3p; 3925-5p; 671-5p; 190a-5p; 454-3p; 329-3p; 145-5p; 99b-3p; 376b-3p; 493-3p; 30d-5p; 34c-5p; 9-5p; 301a-3p; 129-5p; 1307-3p; 204-5p; 296-3p; 500a-5p; 378a-3p |
| Oxidative phosphorylation | 7.87E-03 | 74 | 88 | 483-5p; 342-3p; 590-3p; 378a-5p; 30a-3p; let-7e-5p; 320c; 92a-3p; let-7d-5p; 542-3p; 374a-5p; 31-5p; let-7a-5p; 197-3p; 320b; 486-3p; 25-3p; 149-5p; 128-3p; 99a-5p; 323b-3p; 205-5p; 629-5p; 1-3p; 379-5p; 30e-5p; 130b-3p; 30c-5p; 98-5p; let-7f-5p; 15a-5p; 34a-5p; 7-5p; 411-5p; 665; 15b-5p; 103a-3p; 409-3p; 320a-3p; 130a-3p; 27b-3p; 423-5p; 374b-5p; 628-3p; 181b-5p; 23a-3p; 92b-3p; 17-5p; 484; 192-5p; 421; 497-5p; 26a-5p; 328-3p; 708-5p; 503-5p; 675-3p; 195-5p; 23b-3p; 877-5p; 299-5p; 26b-5p; let-7b-5p; 206; 24-3p; 130b-5p; 671-5p; 454-3p; 329-3p; 889-3p; 99b-5p; 145-5p; 125a-5p; 377-3p; 99b-3p; 376b-3p; 140-5p; 30d-5p; 491-5p; 34c-5p; 9-5p; 301a-3p; 129-5p; 769-5p; 1287-5p; 296-3p; 1185-5p; 543 |
| GABAergic synapse | 9.12E-04 | 69 | 87 | 107; 342-3p; 590-3p; 1185-1-3p; 30a-3p; let-7e-5p; 320c; 320d; 92a-3p; let-7d-5p; 542-3p; 100-3p; 374a-5p; 31-5p; let-7a-5p; 320b; 22-3p; 25-3p; 149-5p; 128-3p; 99a-5p; 205-5p; 1-3p; 374a-3p; 30e-5p; 130b-3p; 30c-5p; 98-5p; let-7f-5p; 15a-5p; 34a-5p; 7-5p; 411-5p; 665; 15b-5p; 103a-3p; let-7a-3p; 151a-3p; 184; 320a-3p; 130a-3p; 27b-3p; 423-5p; 374b-5p; 181b-5p; 584-5p; 23a-3p; 92b-3p; 136-3p; 17-5p; 484; 192-5p; 421; 497-5p; 26a-5p; 195-5p; 23b-3p; 133a-3p; 139-5p; 877-5p; 625-5p; 15b-3p; 6511a-3p; 26b-5p; let-7b-5p; 24-3p; 130b-5p; 3925-5p; 454-3p; 329-3p; 889-3p; 377-3p; 493-3p; 30d-5p; 296-5p; 491-5p; 34c-5p; 9-5p; 301a-3p; 129-5p; 1307-3p; 1271-5p; 204-5p; 1287-5p; 296-3p; 500a-5p; 378a-3p |
| Inflammatory bowel disease IBD | 1.10E-09 | 53 | 86 | 107; 483-5p; 342-3p; 378a-5p; 1185-1-3p; let-7e-5p; 320c; 92a-3p; let-7d-5p; 542-3p; 374a-5p; 31-5p; let-7a-5p; 197-3p; 320b; 486-3p; 149-5p; 128-3p; 205-5p; 1-3p; 323a-3p; 374a-3p; 30e-5p; 130b-3p; 574-3p; 30c-5p; 485-3p; 98-5p; let-7f-5p; 15a-5p; 34a-5p; 7-5p; 576-5p; 15b-5p; 103a-3p; let-7a-3p; 191-5p; 151a-3p; 409-3p; 320a-3p; 130a-3p; 27b-3p; 423-5p; 374b-5p; 181b-5p; 23a-3p; 22-5p; 92b-3p; 17-5p; 484; 192-5p; 497-5p; 154-3p; 26a-5p; 708-5p; 503-5p; 195-5p; 501-5p; 7704; 139-5p; 877-5p; 127-5p; 625-5p; 3613-5p; 6511a-3p; 26b-5p; let-7b-5p; 206; 24-3p; 130b-5p; 337-3p; 454-3p; 329-3p; 145-5p; 125a-5p; 377-3p; 140-5p; 30d-5p; 491-5p; 9-5p; 301a-3p; 129-5p; 769-5p; 204-5p; 500a-5p; 378a-3p |
| Arrhythmogenic right ventricular cardiomyopathy ARVC | 8.95E-05 | 65 | 86 | 107; 483-5p; 342-3p; 590-3p; 378a-5p; 1185-1-3p; 30a-3p; let-7e-5p; 92a-3p; let-7d-5p; 31-5p; let-7a-5p; 197-3p; 22-3p; 486-3p; 25-3p; 149-5p; 205-5p; 629-5p; 1-3p; 30e-5p; 130b-3p; 30c-5p; 485-3p; 98-5p; let-7f-5p; 15a-5p; 34a-5p; 330-5p; 7-5p; 411-5p; 665; 576-5p; 15b-5p; 103a-3p; let-7a-3p; 409-3p; 320a-3p; let-7i-3p; 130a-3p; 423-5p; 23a-3p; 92b-3p; 136-3p; 93-3p; 17-5p; 484; 192-5p; 421; 497-5p; 154-3p; 26a-5p; 195-5p; 501-5p; 23b-3p; 133a-3p; 877-5p; 127-5p; 625-5p; 26b-5p; let-7b-5p; 206; 24-3p; 130b-5p; 3925-5p; 671-5p; 454-3p; 329-3p; 99b-5p; 145-5p; 99b-3p; 376b-3p; 493-3p; 30d-5p; 34c-5p; 9-5p; 301a-3p; 129-5p; 1307-3p; 769-5p; 204-5p; 1287-5p; 296-3p; 125b-1-3p; 500a-5p; 378a-3p |
| Leishmaniasis | 2.13E-04 | 66 | 86 | 107; 483-5p; 342-3p; 590-3p; let-7e-5p; 92a-3p; let-7d-5p; 374a-5p; let-7a-5p; 197-3p; 320b; 25-3p; 149-5p; 181a-2-3p; 128-3p; 126-3p; 323b-3p; 205-5p; 1306-5p; 1-3p; 30e-5p; 130b-3p; 574-3p; 30c-5p; 98-5p; let-7f-5p; 15a-5p; 34a-5p; 330-5p; 7-5p; 665; 576-5p; 15b-5p; 103a-3p; 191-5p; 184; 409-3p; 320a-3p; 130a-3p; 27b-3p; 423-5p; 181b-5p; 584-5p; 22-5p; 92b-3p; 93-3p; 17-5p; 484; 192-5p; 421; 497-5p; 154-3p; 26a-5p; 328-3p; 708-5p; 503-5p; 195-5p; 501-5p; 23b-3p; 7704; 139-5p; 877-5p; 127-5p; 6511a-3p; 26b-5p; let-7b-5p; 24-3p; 130b-5p; 454-3p; 329-3p; 145-5p; 125a-5p; 140-5p; 30d-5p; 491-5p; 9-5p; 301a-3p; 129-5p; 628-5p; 1307-3p; 769-5p; 204-5p; 296-3p; 500a-5p; 378a-3p; 543 |
| RNA degradation | 6.25E-03 | 71 | 86 | 342-3p; 590-3p; 378a-5p; 1185-1-3p; 30a-3p; let-7e-5p; 320c; 92a-3p; 542-3p; 31-5p; let-7a-5p; 197-3p; 22-3p; 25-3p; 149-5p; 128-3p; 323b-3p; 629-5p; 1-3p; 374a-3p; 30e-5p; 130b-3p; 30c-5p; 98-5p; let-7f-5p; 15a-5p; 34a-5p; 330-5p; 7-5p; 411-5p; 665; 15b-5p; 103a-3p; let-7a-3p; 320a-3p; 382-5p; 130a-3p; 27b-3p; 423-5p; 181b-5p; 23a-3p; 92b-3p; 136-3p; 93-3p; 17-5p; 484; 192-5p; 421; 497-5p; 26a-5p; 328-3p; 675-3p; 195-5p; 501-5p; 23b-3p; 139-5p; 877-5p; 625-3p; 127-5p; 625-5p; 299-5p; 26b-5p; let-7b-5p; 206; 24-3p; 130b-5p; 3925-5p; 337-3p; 190a-5p; 454-3p; 329-3p; 889-3p; 99b-5p; 145-5p; 125a-5p; 377-3p; 30d-5p; 34c-5p; 9-5p; 301a-3p; 129-5p; 1307-3p; 769-5p; 1271-5p; 204-5p; 378a-3p |
| Type II diabetes mellitus | 1.56E-07 | 56 | 85 | 107; 483-5p; 342-3p; 590-3p; 1185-1-3p; 30a-3p; let-7e-5p; 92a-3p; let-7d-5p; 542-3p; 374a-5p; 31-5p; let-7a-5p; 197-3p; 320b; 486-3p; 25-3p; 128-3p; 126-3p; 99a-5p; 205-5p; 1-3p; 30e-5p; 130b-3p; 30c-5p; 98-5p; let-7f-5p; 15a-5p; 34a-5p; 330-5p; 7-5p; 576-5p; 15b-5p; 103a-3p; 191-5p; 151a-3p; 184; 409-3p; 320a-3p; 130a-3p; 27b-3p; 423-5p; 374b-5p; 181b-5p; 584-5p; 23a-3p; 92b-3p; 17-5p; 484; 192-5p; 421; 497-5p; 154-3p; 26a-5p; 328-3p; 503-5p; 195-5p; 23b-3p; 133a-3p; 139-5p; 877-5p; 625-5p; 15b-3p; 26b-5p; 432-5p; let-7b-5p; 24-3p; 130b-5p; 454-3p; 329-3p; 99b-5p; 145-5p; 125a-5p; 377-3p; 30d-5p; 193a-5p; 491-5p; 301a-3p; 129-5p; 628-5p; 1307-3p; 769-5p; 204-5p; 296-3p; 378a-3p |
| Notch signaling pathway | 4.94E-06 | 60 | 85 | 107; 483-5p; 342-3p; 590-3p; 1185-1-3p; 30a-3p; let-7e-5p; 320c; 320d; 92a-3p; let-7d-5p; 542-3p; 374a-5p; let-7a-5p; 197-3p; 320b; 25-3p; 149-5p; 128-3p; 205-5p; 1-3p; 30e-5p; 130b-3p; 574-3p; 30c-5p; 485-3p; 98-5p; let-7f-5p; 15a-5p; 34a-5p; 7-5p; 665; 15b-5p; 103a-3p; 191-5p; 320a-3p; 382-5p; 130a-3p; 27b-3p; 423-5p; 374b-5p; 628-3p; 181b-5p; 23a-3p; 92b-3p; 136-3p; 93-3p; 17-5p; 484; 192-5p; 497-5p; 26a-5p; 195-5p; 501-5p; 23b-3p; 133a-3p; 139-5p; 127-5p; 625-5p; 26b-5p; let-7b-5p; 206; 24-3p; 130b-5p; 598-3p; 671-5p; 454-3p; 329-3p; 145-5p; 125a-5p; 140-5p; 30d-5p; 296-5p; 491-5p; 34c-5p; 9-5p; 301a-3p; 129-5p; 1307-3p; 769-5p; 1271-5p; 204-5p; 296-3p; 378a-3p; 543 |
| Amoebiasis | 2.99E-03 | 68 | 84 | 107; 590-3p; 378a-5p; 30a-3p; let-7e-5p; 92a-3p; let-7d-5p; 542-3p; 100-3p; 374a-5p; 31-5p; let-7a-5p; 197-3p; 320b; 22-3p; 25-3p; 149-5p; 128-3p; 126-3p; 99a-5p; 205-5p; 1-3p; 379-5p; 30e-5p; 130b-3p; 574-3p; 30c-5p; 98-5p; let-7f-5p; 15a-5p; 34a-5p; 7-5p; 15b-5p; 103a-3p; 127-3p; 184; 409-3p; 320a-3p; 382-5p; 130a-3p; 423-5p; 23a-3p; 92b-3p; 93-3p; 17-5p; 484; 192-5p; 421; 497-5p; 26a-5p; 503-5p; 195-5p; 501-5p; 23b-3p; 133a-3p; 139-5p; 877-5p; 127-5p; 625-5p; 6511a-3p; 26b-5p; 432-5p; let-7b-5p; 206; 24-3p; 130b-5p; 454-3p; 329-3p; 145-5p; 125a-5p; 376b-3p; 140-5p; 30d-5p; 193a-5p; 34c-5p; 9-5p; 301a-3p; 129-5p; 769-5p; 204-5p; 296-3p; 500a-5p; 378a-3p; 543 |
| Basal cell carcinoma | 9.79E-03 | 70 | 84 | 27a-5p; 107; 590-3p; 378a-5p; 1185-1-3p; 30a-3p; let-7e-5p; 320c; 92a-3p; let-7d-5p; 542-3p; 374a-5p; 31-5p; let-7a-5p; 22-3p; 486-3p; 25-3p; 149-5p; 181a-2-3p; 128-3p; 1-3p; 374a-3p; 30e-5p; 130b-3p; 30c-5p; 485-3p; 98-5p; let-7f-5p; 15a-5p; 34a-5p; 7-5p; 665; 576-5p; 15b-5p; 103a-3p; let-7a-3p; 409-3p; 320a-3p; 130a-3p; 27b-3p; 423-5p; 374b-5p; 23a-3p; 92b-3p; 136-3p; 93-3p; 17-5p; 484; 192-5p; 421; 497-5p; 154-3p; 26a-5p; 708-5p; 503-5p; 195-5p; 501-5p; 7704; 133a-3p; 139-5p; 625-5p; 299-5p; 26b-5p; let-7b-5p; 24-3p; 130b-5p; 454-3p; 329-3p; 145-5p; 125a-5p; 377-3p; 99b-3p; 140-5p; 493-3p; 30d-5p; 491-5p; 9-5p; 301a-3p; 129-5p; 376c-3p; 769-5p; 204-5p; 125b-1-3p; 378a-3p |
| RIG-I-like receptor signaling pathway | 3.10E-05 | 60 | 83 | 107; 483-5p; 342-3p; 590-3p; 378a-5p; 1185-1-3p; let-7e-5p; 362-5p; 92a-3p; let-7d-5p; 100-3p; 374a-5p; let-7a-5p; 197-3p; 486-3p; 25-3p; 181a-2-3p; 128-3p; 126-3p; 99a-5p; 205-5p; 1306-5p; 1-3p; 323a-3p; 30e-5p; 130b-3p; 574-3p; 30c-5p; 98-5p; let-7f-5p; 15a-5p; 34a-5p; 7-5p; 665; 15b-5p; 103a-3p; 151a-3p; 320a-3p; 130a-3p; 27b-3p; 181b-5p; 23a-3p; 92b-3p; 17-5p; 484; 192-5p; 421; 497-5p; 26a-5p; 328-3p; 708-5p; 503-5p; 195-5p; 23b-3p; 139-5p; 877-5p; 625-5p; 664a-5p; 299-5p; 6511a-3p; 26b-5p; let-7b-5p; 24-3p; 130b-5p; 337-3p; 454-3p; 329-3p; 145-5p; 125a-5p; 376b-3p; 140-5p; 30d-5p; 296-5p; 193a-5p; 491-5p; 9-5p; 301a-3p; 129-5p; 1307-3p; 204-5p; 500a-5p; 1185-5p; 543 |
| Amphetamine addiction | 1.29E-04 | 61 | 82 | 342-3p; 378a-5p; 1185-1-3p; 30a-3p; 92a-3p; 100-3p; 374a-5p; let-7a-5p; 197-3p; 320b; 22-3p; 486-3p; 149-5p; 181a-2-3p; 128-3p; 126-3p; 99a-5p; 323b-3p; 205-5p; 1-3p; 323a-3p; 30e-5p; 130b-3p; 30c-5p; let-7f-5p; 15a-5p; 34a-5p; 7-5p; 411-5p; 576-5p; 15b-5p; 103a-3p; let-7a-3p; 184; 409-3p; 320a-3p; 382-5p; 130a-3p; 27b-3p; 423-5p; 374b-5p; 181b-5p; 92b-3p; 93-3p; 17-5p; 484; 192-5p; 26a-5p; 501-5p; 23b-3p; 7704; 133a-3p; 139-5p; 625-5p; 26b-5p; 432-5p; let-7b-5p; 206; 24-3p; 130b-5p; 598-3p; 3925-5p; 671-5p; 190a-5p; 454-3p; 329-3p; 99b-5p; 145-5p; 125a-5p; 376b-3p; 493-3p; 30d-5p; 491-5p; 9-5p; 301a-3p; 129-5p; 628-5p; 769-5p; 204-5p; 500a-5p; 1185-5p; 543 |
| Lysine degradation | 3.74E-04 | 63 | 82 | 107; 342-3p; 590-3p; 378a-5p; 30a-3p; let-7e-5p; 320c; 92a-3p; let-7d-5p; 542-3p; 374a-5p; 31-5p; let-7a-5p; 197-3p; 320b; 486-3p; 25-3p; 149-5p; 128-3p; 126-3p; 99a-5p; 1306-5p; 1-3p; 30e-5p; 130b-3p; 30c-5p; 98-5p; let-7f-5p; 15a-5p; 34a-5p; 7-5p; 665; 15b-5p; 103a-3p; 127-3p; 151a-3p; 320a-3p; 27b-3p; 423-5p; 181b-5p; 92b-3p; 93-3p; 17-5p; 484; 192-5p; 421; 497-5p; 26a-5p; 328-3p; 708-5p; 503-5p; 195-5p; 501-5p; 23b-3p; 7704; 877-5p; 127-5p; 625-5p; 664a-5p; 6511a-3p; 26b-5p; let-7b-5p; 24-3p; 130b-5p; 671-5p; 329-3p; 889-3p; 99b-5p; 145-5p; 125a-5p; 377-3p; 30d-5p; 296-5p; 491-5p; 34c-5p; 9-5p; 301a-3p; 129-5p; 628-5p; 1271-5p; 204-5p; 296-3p |
| Rheumatoid arthritis | 2.26E-05 | 58 | 81 | 107; 342-3p; 590-3p; 378a-5p; 30a-3p; let-7e-5p; 92a-3p; let-7d-5p; 542-3p; 100-3p; 374a-5p; 31-5p; let-7a-5p; 197-3p; 486-3p; 149-5p; 128-3p; 126-3p; 323b-3p; 205-5p; 1-3p; 379-5p; 30e-5p; 130b-3p; 574-3p; 30c-5p; 98-5p; let-7f-5p; 15a-5p; 34a-5p; 7-5p; 665; 576-5p; 15b-5p; 191-5p; 184; 409-3p; 320a-3p; 130a-3p; 27b-3p; 423-5p; 374b-5p; 181b-5p; 23a-3p; 22-5p; 92b-3p; 93-3p; 17-5p; 484; 192-5p; 421; 497-5p; 26a-5p; 708-5p; 503-5p; 195-5p; 7704; 139-5p; 299-5p; 26b-5p; let-7b-5p; 206; 24-3p; 130b-5p; 454-3p; 329-3p; 145-5p; 125a-5p; 377-3p; 140-5p; 30d-5p; 296-5p; 34c-5p; 9-5p; 301a-3p; 129-5p; 769-5p; 204-5p; 296-3p; 378a-3p; 543 |
| Apoptosis - multiple species | 7.25E-04 | 62 | 80 | 342-3p; 590-3p; 378a-5p; 1185-1-3p; 30a-3p; let-7e-5p; 362-5p; 320c; 92a-3p; let-7d-5p; 542-3p; 374a-5p; let-7a-5p; 197-3p; 22-3p; 25-3p; 149-5p; 181a-2-3p; 128-3p; 126-3p; 205-5p; 1306-5p; 1-3p; 323a-3p; 30e-5p; 130b-3p; 30c-5p; 98-5p; let-7f-5p; 15a-5p; 34a-5p; 7-5p; 665; 15b-5p; 103a-3p; 184; 382-5p; 130a-3p; 27b-3p; 423-5p; 181b-5p; 584-5p; 23a-3p; 22-5p; 92b-3p; 93-3p; 17-5p; 484; 192-5p; 421; 497-5p; 26a-5p; 708-5p; 503-5p; 195-5p; 23b-3p; 133a-3p; 139-5p; 877-5p; 625-5p; 15b-3p; 6511a-3p; 26b-5p; let-7b-5p; 206; 24-3p; 454-3p; 889-3p; 125a-5p; 376b-3p; 140-5p; 30d-5p; 296-5p; 491-5p; 34c-5p; 9-5p; 301a-3p; 129-5p; 376c-3p; 204-5p |
| Vasopressin-regulated water reabsorption | 3.15E-02 | 68 | 79 | 181a-3p; 590-3p; 1185-1-3p; let-7e-5p; 320c; 320d; 92a-3p; let-7d-5p; 374a-5p; 31-5p; 132-5p; let-7a-5p; 320b; 22-3p; 25-3p; 149-5p; 181a-2-3p; 128-3p; 99a-5p; 323b-3p; 205-5p; 1-3p; 30e-5p; 130b-3p; 30c-5p; 98-5p; let-7f-5p; 15a-5p; 34a-5p; 330-5p; 7-5p; 665; 576-5p; 15b-5p; 103a-3p; let-7a-3p; 184; 409-3p; 320a-3p; 382-5p; 130a-3p; 27b-3p; 423-5p; 181b-5p; 584-5p; 23a-3p; 92b-3p; 17-5p; 484; 192-5p; 497-5p; 26a-5p; 328-3p; 503-5p; 195-5p; 23b-3p; 133a-3p; 877-5p; 625-5p; 15b-3p; 299-5p; 26b-5p; 432-5p; let-7b-5p; 206; 24-3p; 130b-5p; 454-3p; 889-3p; 125a-5p; 377-3p; 376b-3p; 30d-5p; 34c-5p; 9-5p; 301a-3p; 129-5p; 204-5p; 378a-3p |
| Aldosterone-regulated sodium reabsorption | 5.64E-05 | 56 | 78 | 107; 483-5p; 1185-1-3p; 30a-3p; let-7e-5p; 92a-3p; 542-3p; 100-3p; 31-5p; let-7a-5p; 197-3p; 320b; 486-3p; 25-3p; 181a-2-3p; 128-3p; 126-3p; 99a-5p; 1-3p; 374a-3p; 130b-3p; 30c-5p; 98-5p; 15a-5p; 34a-5p; 330-5p; 7-5p; 411-5p; 665; 576-5p; 15b-5p; 103a-3p; 184; 320a-3p; 130a-3p; 423-5p; 584-5p; 23a-3p; 92b-3p; 93-3p; 17-5p; 484; 192-5p; 421; 497-5p; 154-3p; 26a-5p; 503-5p; 195-5p; 941; 23b-3p; 133a-3p; 139-5p; 877-5p; 625-5p; 26b-5p; 432-5p; let-7b-5p; 206; 24-3p; 130b-5p; 671-5p; 190a-5p; 454-3p; 329-3p; 145-5p; 125a-5p; 30d-5p; 193a-5p; 301a-3p; 129-5p; 628-5p; 376c-3p; 1307-3p; 769-5p; 296-3p; 378a-3p; 543 |
| Vibrio cholerae infection | 6.93E-04 | 60 | 78 | 107; 342-3p; 30a-3p; let-7e-5p; 320c; 320d; 92a-3p; let-7d-5p; 542-3p; 374a-5p; 31-5p; let-7a-5p; 197-3p; 320b; 22-3p; 486-3p; 25-3p; 149-5p; 181a-2-3p; 128-3p; 99a-5p; 323b-3p; 205-5p; 1-3p; 30e-5p; 130b-3p; 30c-5p; 98-5p; let-7f-5p; 15a-5p; 34a-5p; 7-5p; 665; 15b-5p; 103a-3p; 320a-3p; let-7i-3p; 130a-3p; 423-5p; 374b-5p; 181b-5p; 92b-3p; 93-3p; 17-5p; 484; 192-5p; 421; 497-5p; 26a-5p; 503-5p; 195-5p; 23b-3p; 133a-3p; 877-5p; 127-5p; 664a-5p; 299-5p; 26b-5p; 432-5p; let-7b-5p; 24-3p; 3925-5p; 454-3p; 329-3p; 99b-5p; 145-5p; 377-3p; 99b-3p; 376b-3p; 493-3p; 30d-5p; 34c-5p; 9-5p; 301a-3p; 1307-3p; 204-5p; 296-3p; 378a-3p |
| Pancreatic secretion | 1.67E-02 | 66 | 78 | 133a-5p; 483-5p; 590-3p; let-7e-5p; 320c; 92a-3p; let-7d-5p; 542-3p; 374a-5p; 31-5p; let-7a-5p; 197-3p; 320b; 486-3p; 25-3p; 181a-2-3p; 128-3p; 99a-5p; 205-5p; 1-3p; 374a-3p; 30e-5p; 130b-3p; 574-3p; 30c-5p; 98-5p; let-7f-5p; 15a-5p; 34a-5p; 7-5p; 411-5p; 665; 576-5p; 15b-5p; 184; 320a-3p; 130a-3p; 27b-3p; 423-5p; 374b-5p; 181b-5p; 92b-3p; 93-3p; 17-5p; 484; 192-5p; 26a-5p; 195-5p; 941; 501-5p; 23b-3p; 133a-3p; 139-5p; 877-5p; 127-5p; 625-5p; 26b-5p; 432-5p; let-7b-5p; 24-3p; 130b-5p; 337-3p; 671-5p; 454-3p; 329-3p; 145-5p; 99b-3p; 376b-3p; 30d-5p; 34c-5p; 9-5p; 301a-3p; 129-5p; 1307-3p; 204-5p; 296-3p; 500a-5p; 378a-3p |
| Glycerophospholipid metabolism | 2.05E-02 | 66 | 78 | 107; 483-5p; 342-3p; 590-3p; 378a-5p; 30a-3p; let-7e-5p; 92a-3p; let-7d-5p; 542-3p; 100-3p; 374a-5p; let-7a-5p; 197-3p; 149-5p; 128-3p; 205-5p; 1306-5p; 1-3p; 30e-5p; 130b-3p; 30c-5p; 98-5p; let-7f-5p; 15a-5p; 34a-5p; 7-5p; 411-5p; 665; 15b-5p; 103a-3p; 191-5p; 184; 320a-3p; 130a-3p; 27b-3p; 423-5p; 374b-5p; 181b-5p; 23a-3p; 92b-3p; 93-3p; 17-5p; 484; 192-5p; 421; 497-5p; 26a-5p; 328-3p; 503-5p; 195-5p; 23b-3p; 7704; 877-5p; 625-3p; 127-5p; 625-5p; 664a-5p; 15b-3p; 26b-5p; let-7b-5p; 206; 24-3p; 130b-5p; 454-3p; 329-3p; 889-3p; 99b-5p; 145-5p; 125a-5p; 30d-5p; 34c-5p; 9-5p; 301a-3p; 129-5p; 769-5p; 296-3p; 543 |
| Ribosome biogenesis in eukaryotes | 2.35E-02 | 66 | 78 | 107; 483-5p; 342-3p; 590-3p; let-7e-5p; 92a-3p; let-7d-5p; 542-3p; 374a-5p; 31-5p; let-7a-5p; 197-3p; 22-3p; 486-3p; 25-3p; 149-5p; 128-3p; 99a-5p; 205-5p; 1-3p; 323a-3p; 30e-5p; 130b-3p; 30c-5p; 98-5p; 15a-5p; 34a-5p; 330-5p; 7-5p; 411-5p; 665; 15b-5p; 103a-3p; 151a-3p; 7706; 184; 320a-3p; 382-5p; 130a-3p; 27b-3p; 423-5p; 374b-5p; 181b-5p; 22-5p; 92b-3p; 93-3p; 17-5p; 484; 192-5p; 497-5p; 195-5p; 941; 23b-3p; 625-3p; 625-5p; 664a-5p; 6511a-3p; 26b-5p; let-7b-5p; 206; 24-3p; 337-3p; 671-5p; 454-3p; 329-3p; 889-3p; 145-5p; 125a-5p; 30d-5p; 193a-5p; 491-5p; 9-5p; 301a-3p; 129-5p; 204-5p; 1287-5p; 296-3p; 1185-5p |
| Renin secretion | 3.12E-03 | 61 | 77 | 107; 590-3p; 1185-1-3p; let-7e-5p; 320c; 320d; 92a-3p; let-7d-5p; let-7a-5p; 197-3p; 320b; 22-3p; 486-3p; 25-3p; 149-5p; 181a-2-3p; 128-3p; 205-5p; 1-3p; 379-5p; 30e-5p; 130b-3p; 30c-5p; 98-5p; let-7f-5p; 15a-5p; 34a-5p; 330-5p; 7-5p; 411-5p; 665; 576-5p; 15b-5p; 103a-3p; let-7a-3p; 409-3p; 320a-3p; 382-5p; 130a-3p; 27b-3p; 423-5p; 181b-5p; 23a-3p; 22-5p; 92b-3p; 17-5p; 195-5p; 501-5p; 23b-3p; 133a-3p; 625-3p; 625-5p; 664a-5p; 26b-5p; 432-5p; let-7b-5p; 206; 24-3p; 130b-5p; 598-3p; 3925-5p; 190a-5p; 454-3p; 329-3p; 145-5p; 125a-5p; 377-3p; 376b-3p; 493-3p; 30d-5p; 34c-5p; 9-5p; 301a-3p; 129-5p; 1271-5p; 204-5p; 500a-5p |
| ECM-receptor interaction | 4.03E-03 | 62 | 77 | 107; 30a-3p; let-7e-5p; 320c; 92a-3p; let-7d-5p; 542-3p; 31-5p; let-7a-5p; 197-3p; 486-3p; 25-3p; 149-5p; 128-3p; 99a-5p; 323b-3p; 205-5p; 1-3p; 379-5p; 130b-3p; 30c-5p; 98-5p; let-7f-5p; 15a-5p; 34a-5p; 330-5p; 7-5p; 411-5p; 665; 576-5p; 15b-5p; 103a-3p; let-7a-3p; 320a-3p; 27b-3p; 423-5p; 181b-5p; 92b-3p; 136-3p; 93-3p; 17-5p; 484; 192-5p; 421; 497-5p; 154-3p; 26a-5p; 328-3p; 708-5p; 503-5p; 675-3p; 195-5p; 133a-3p; 625-3p; 127-5p; 625-5p; 664a-5p; 299-5p; 26b-5p; let-7b-5p; 24-3p; 130b-5p; 329-3p; 145-5p; 125a-5p; 376b-3p; 140-5p; 491-5p; 34c-5p; 9-5p; 129-5p; 1307-3p; 769-5p; 204-5p; 1287-5p; 125b-1-3p; 378a-3p |
| Insulin secretion | 1.15E-02 | 63 | 76 | 27a-5p; 342-3p; 590-3p; 378a-5p; 1185-1-3p; 30a-3p; let-7e-5p; 362-5p; 92a-3p; let-7d-5p; 374a-5p; let-7a-5p; 197-3p; 320b; 22-3p; 486-3p; 25-3p; 149-5p; 181a-2-3p; 128-3p; 99a-5p; 323b-3p; 205-5p; 1306-5p; 1-3p; 30c-5p; 98-5p; 15a-5p; 34a-5p; 7-5p; 411-5p; 576-5p; 103a-3p; let-7a-3p; 184; 409-3p; 320a-3p; 27b-3p; 423-5p; 374b-5p; 181b-5p; 92b-3p; 93-3p; 17-5p; 484; 192-5p; 421; 497-5p; 26a-5p; 941; 501-5p; 23b-3p; 133a-3p; 877-5p; 127-5p; 625-5p; 26b-5p; 432-5p; let-7b-5p; 206; 24-3p; 130b-5p; 671-5p; 190a-5p; 329-3p; 889-3p; 125a-5p; 377-3p; 376b-3p; 30d-5p; 9-5p; 129-5p; 204-5p; 296-3p; 500a-5p; 1185-5p |
| Cortisol synthesis and secretion | 4.45E-04 | 56 | 75 | 342-3p; 590-3p; 378a-5p; 1185-1-3p; 92a-3p; let-7d-5p; 542-3p; 374a-5p; 31-5p; let-7a-5p; 197-3p; 320b; 22-3p; 25-3p; 149-5p; 128-3p; 99a-5p; 323b-3p; 205-5p; 1-3p; 30e-5p; 130b-3p; 30c-5p; 98-5p; let-7f-5p; 15a-5p; 34a-5p; 7-5p; 576-5p; 15b-5p; 103a-3p; let-7a-3p; 320a-3p; 130a-3p; 27b-3p; 423-5p; 374b-5p; 181b-5p; 23a-3p; 92b-3p; 17-5p; 484; 192-5p; 195-5p; 501-5p; 23b-3p; 7704; 133a-3p; 877-5p; 625-5p; 299-5p; 26b-5p; 432-5p; let-7b-5p; 206; 24-3p; 130b-5p; 454-3p; 329-3p; 99b-5p; 145-5p; 125a-5p; 377-3p; 376b-3p; 140-5p; 30d-5p; 296-5p; 34c-5p; 9-5p; 301a-3p; 129-5p; 376c-3p; 1307-3p; 204-5p; 500a-5p |
| Ovarian steroidogenesis | 7.42E-04 | 57 | 75 | 342-3p; 590-3p; 1185-1-3p; let-7e-5p; 92a-3p; let-7d-5p; let-7a-5p; 320b; 22-3p; 149-5p; 181a-2-3p; 128-3p; 99a-5p; 205-5p; 1-3p; 30e-5p; 130b-3p; 30c-5p; 98-5p; let-7f-5p; 34a-5p; 7-5p; 665; 576-5p; 15b-5p; let-7a-3p; 320a-3p; 130a-3p; 27b-3p; 181b-5p; 92b-3p; 17-5p; 484; 192-5p; 421; 497-5p; 26a-5p; 503-5p; 675-3p; 195-5p; 501-5p; 23b-3p; 7704; 133a-3p; 139-5p; 877-5p; 625-5p; 15b-3p; 299-5p; 26b-5p; 432-5p; let-7b-5p; 206; 24-3p; 130b-5p; 190a-5p; 454-3p; 329-3p; 99b-5p; 145-5p; 125a-5p; 377-3p; 376b-3p; 140-5p; 30d-5p; 301a-3p; 129-5p; 376c-3p; 1307-3p; 1271-5p; 204-5p; 296-3p; 125b-1-3p; 500a-5p; 378a-3p |
| Hematopoietic cell lineage | 5.12E-03 | 59 | 74 | 107; 342-3p; 30a-3p; let-7e-5p; 92a-3p; let-7d-5p; 542-3p; 31-5p; let-7a-5p; 197-3p; 22-3p; 486-3p; 149-5p; 128-3p; 205-5p; 1-3p; 379-5p; 30e-5p; 130b-3p; 30c-5p; 485-3p; 98-5p; let-7f-5p; 15a-5p; 34a-5p; 330-5p; 7-5p; 665; 576-5p; 15b-5p; 103a-3p; let-7a-3p; 191-5p; 184; 320a-3p; 130a-3p; 27b-3p; 423-5p; 181b-5p; 6724-5p; 23a-3p; 22-5p; 92b-3p; 136-3p; 17-5p; 484; 192-5p; 497-5p; 26a-5p; 328-3p; 708-5p; 503-5p; 195-5p; 7704; 877-5p; 26b-5p; let-7b-5p; 24-3p; 130b-5p; 3925-5p; 190a-5p; 329-3p; 145-5p; 125a-5p; 377-3p; 376b-3p; 30d-5p; 34c-5p; 9-5p; 376c-3p; 1307-3p; 769-5p; 204-5p; 1287-5p |
| Cardiac muscle contraction | 4.01E-02 | 64 | 74 | 483-5p; 590-3p; 378a-5p; let-7e-5p; 320c; 92a-3p; let-7d-5p; 31-5p; let-7a-5p; 197-3p; 320b; 486-3p; 25-3p; 149-5p; 181a-2-3p; 128-3p; 99a-5p; 1-3p; 30e-5p; 130b-3p; 30c-5p; 98-5p; let-7f-5p; 15a-5p; 34a-5p; 7-5p; 411-5p; 665; 15b-5p; 103a-3p; 409-3p; 320a-3p; 130a-3p; 423-5p; 628-3p; 23a-3p; 92b-3p; 93-3p; 17-5p; 484; 192-5p; 421; 497-5p; 26a-5p; 708-5p; 195-5p; 941; 501-5p; 23b-3p; 133a-3p; 139-5p; 127-5p; 625-5p; 299-5p; 26b-5p; let-7b-5p; 24-3p; 130b-5p; 671-5p; 454-3p; 329-3p; 145-5p; 125a-5p; 99b-3p; 140-5p; 30d-5p; 301a-3p; 129-5p; 769-5p; 204-5p; 296-3p; 500a-5p; 378a-3p; 1185-5p |
| Olfactory transduction | 5.66E-04 | 55 | 73 | 483-5p; 342-3p; let-7d-3p; 590-3p; 30a-3p; let-7e-5p; 92a-3p; 542-3p; let-7a-5p; 22-3p; 486-3p; 25-3p; 149-5p; 128-3p; 99a-5p; 629-5p; 1-3p; 130b-3p; 30c-5p; 98-5p; let-7f-5p; 15a-5p; 330-5p; 7-5p; 411-5p; 665; 15b-5p; 103a-3p; 191-5p; 127-3p; 409-3p; 320a-3p; 382-5p; 130a-3p; 27b-3p; 423-5p; 181b-5p; 23a-3p; 92b-3p; 17-5p; 484; 192-5p; 421; 497-5p; 26a-5p; 195-5p; 501-5p; 23b-3p; 133a-3p; 877-5p; 625-5p; 664a-5p; 26b-5p; 432-5p; let-7b-5p; 206; 24-3p; 598-3p; 3925-5p; 671-5p; 190a-5p; 454-3p; 889-3p; 493-3p; 491-5p; 9-5p; 301a-3p; 129-5p; 769-5p; 1271-5p; 204-5p; 1287-5p; 500a-5p |
| Regulation of lipolysis in adipocytes | 1.52E-03 | 56 | 73 | 27a-5p; 107; 590-3p; 1185-1-3p; 30a-3p; let-7e-5p; 320c; 320d; 92a-3p; let-7d-5p; 542-3p; let-7a-5p; 320b; 22-3p; 25-3p; 149-5p; 128-3p; 126-3p; 99a-5p; 205-5p; 1-3p; 379-5p; 374a-3p; 30e-5p; 130b-3p; 30c-5p; 98-5p; 15a-5p; 34a-5p; 330-5p; 7-5p; 665; 15b-5p; 103a-3p; 191-5p; 184; 409-3p; 320a-3p; 374b-5p; 181b-5p; 23a-3p; 92b-3p; 17-5p; 484; 192-5p; 497-5p; 26a-5p; 708-5p; 503-5p; 195-5p; 501-5p; 23b-3p; 139-5p; 877-5p; 625-3p; 625-5p; 664a-5p; 26b-5p; 432-5p; let-7b-5p; 206; 130b-5p; 329-3p; 145-5p; 125a-5p; 376b-3p; 30d-5p; 193a-5p; 129-5p; 628-5p; 204-5p; 296-3p; 378a-3p |
| Cytosolic DNA-sensing pathway | 7.13E-06 | 47 | 71 | 107; 483-5p; 342-3p; 590-3p; 378a-5p; 1185-1-3p; let-7e-5p; 362-5p; 92a-3p; let-7d-5p; 374a-5p; let-7a-5p; 197-3p; 486-3p; 25-3p; 149-5p; 126-3p; 1306-5p; 1-3p; 374a-3p; 30e-5p; 130b-3p; 30c-5p; 98-5p; let-7f-5p; 15a-5p; 34a-5p; 7-5p; 411-5p; 665; 15b-5p; 151a-3p; 320a-3p; 130a-3p; 27b-3p; 423-5p; 584-5p; 23a-3p; 136-3p; 17-5p; 484; 497-5p; 26a-5p; 328-3p; 708-5p; 503-5p; 195-5p; 23b-3p; 7704; 139-5p; 877-5p; 625-5p; 664a-5p; 26b-5p; 432-5p; let-7b-5p; 24-3p; 130b-5p; 337-3p; 454-3p; 329-3p; 145-5p; 125a-5p; 30d-5p; 296-5p; 193a-5p; 9-5p; 301a-3p; 1307-3p; 204-5p; 500a-5p |
| Viral protein interaction with cytokine and cytokine receptor | 2.50E-03 | 55 | 71 | 107; 590-3p; 378a-5p; 30a-3p; let-7e-5p; 92a-3p; let-7d-5p; 542-3p; 100-3p; 374a-5p; 31-5p; let-7a-5p; 197-3p; 22-3p; 25-3p; 149-5p; 128-3p; 126-3p; 205-5p; 629-5p; 1-3p; 323a-3p; 30e-5p; 130b-3p; 30c-5p; 98-5p; let-7f-5p; 15a-5p; 34a-5p; 7-5p; 411-5p; 665; 184; 130a-3p; 27b-3p; 374b-5p; 628-3p; 181b-5p; 23a-3p; 92b-3p; 93-3p; 17-5p; 484; 192-5p; 26a-5p; 708-5p; 195-5p; 139-5p; 127-5p; 625-5p; 15b-3p; 26b-5p; let-7b-5p; 206; 24-3p; 130b-5p; 190a-5p; 454-3p; 329-3p; 125a-5p; 30d-5p; 34c-5p; 9-5p; 301a-3p; 129-5p; 376c-3p; 1307-3p; 769-5p; 1271-5p; 204-5p; 296-3p |
| N-Glycan biosynthesis | 1.23E-04 | 49 | 69 | 107; 483-5p; 590-3p; 378a-5p; let-7e-5p; 92a-3p; 542-3p; 31-5p; 197-3p; 22-3p; 25-3p; 149-5p; 128-3p; 323b-3p; 1306-5p; 1-3p; 30e-5p; 130b-3p; 30c-5p; 485-3p; 98-5p; 15a-5p; 34a-5p; 7-5p; 665; 15b-5p; 103a-3p; let-7a-3p; 409-3p; 320a-3p; 382-5p; 130a-3p; 27b-3p; 181b-5p; 6724-5p; 23a-3p; 22-5p; 92b-3p; 93-3p; 17-5p; 484; 192-5p; 26a-5p; 328-3p; 195-5p; 23b-3p; 127-5p; 664a-5p; 15b-3p; 6511a-3p; 26b-5p; 432-5p; let-7b-5p; 24-3p; 130b-5p; 598-3p; 3925-5p; 454-3p; 329-3p; 145-5p; 125a-5p; 377-3p; 99b-3p; 30d-5p; 296-5p; 301a-3p; 129-5p; 769-5p; 296-3p |
| Sphingolipid metabolism | 1.78E-03 | 53 | 69 | 27a-5p; 342-3p; 378a-5p; let-7e-5p; 362-5p; 92a-3p; let-7d-5p; 31-5p; let-7a-5p; 197-3p; 149-5p; 128-3p; 99a-5p; 205-5p; 1-3p; 130b-3p; 98-5p; let-7f-5p; 15a-5p; 34a-5p; 330-5p; 7-5p; 411-5p; 665; 576-5p; 15b-5p; let-7a-3p; 191-5p; 184; 130a-3p; 27b-3p; 374b-5p; 181b-5p; 92b-3p; 17-5p; 484; 192-5p; 497-5p; 328-3p; 503-5p; 195-5p; 133a-3p; 139-5p; 625-3p; 625-5p; 664a-5p; 299-5p; 26b-5p; let-7b-5p; 206; 24-3p; 130b-5p; 671-5p; 190a-5p; 454-3p; 329-3p; 145-5p; 125a-5p; 377-3p; 140-5p; 9-5p; 301a-3p; 129-5p; 1307-3p; 769-5p; 1271-5p; 204-5p; 296-3p; 125b-1-3p |
| Bile secretion | 1.92E-02 | 57 | 69 | 1185-1-3p; 92a-3p; let-7d-5p; 374a-5p; 132-5p; 197-3p; 320b; 22-3p; 486-3p; 149-5p; 181a-2-3p; 128-3p; 99a-5p; 205-5p; 1-3p; 30e-5p; 130b-3p; 574-3p; 30c-5p; 98-5p; 15a-5p; 34a-5p; 7-5p; 411-5p; 15b-5p; let-7a-3p; 320a-3p; 130a-3p; 27b-3p; 423-5p; 374b-5p; 181b-5p; 92b-3p; 93-3p; 17-5p; 484; 192-5p; 497-5p; 26a-5p; 328-3p; 503-5p; 195-5p; 941; 501-5p; 23b-3p; 7704; 877-5p; 625-5p; 299-5p; 26b-5p; 432-5p; let-7b-5p; 24-3p; 130b-5p; 671-5p; 454-3p; 329-3p; 889-3p; 145-5p; 377-3p; 99b-3p; 376b-3p; 140-5p; 30d-5p; 34c-5p; 301a-3p; 129-5p; 1307-3p; 204-5p |
| Prion diseases | 7.66E-06 | 45 | 68 | 107; 483-5p; 590-3p; 30a-3p; 92a-3p; let-7a-5p; 197-3p; 320b; 22-3p; 486-3p; 149-5p; 128-3p; 205-5p; 1-3p; 379-5p; 30e-5p; 130b-3p; 30c-5p; 98-5p; let-7f-5p; 15a-5p; 34a-5p; 7-5p; 15b-5p; 191-5p; 409-3p; 320a-3p; 382-5p; 130a-3p; 27b-3p; 181b-5p; 584-5p; 22-5p; 92b-3p; 93-3p; 17-5p; 484; 192-5p; 497-5p; 154-3p; 26a-5p; 195-5p; 23b-3p; 139-5p; 127-5p; 6511a-3p; 26b-5p; let-7b-5p; 206; 24-3p; 454-3p; 329-3p; 889-3p; 125a-5p; 377-3p; 140-5p; 30d-5p; 296-5p; 34c-5p; 9-5p; 301a-3p; 129-5p; 1307-3p; 769-5p; 204-5p; 296-3p; 125b-1-3p; 378a-3p |
| Endocrine and other factor-regulated calcium reabsorption | 6.61E-04 | 50 | 68 | 590-3p; let-7e-5p; 92a-3p; let-7d-5p; 31-5p; let-7a-5p; 197-3p; 320b; 22-3p; 25-3p; 181a-2-3p; 128-3p; 99a-5p; 205-5p; 1-3p; 130b-3p; 574-3p; 30c-5p; 485-3p; 98-5p; 15a-5p; 34a-5p; 330-5p; 7-5p; 411-5p; 665; 15b-5p; 103a-3p; 184; 320a-3p; 382-5p; 130a-3p; 27b-3p; 423-5p; 181b-5p; 92b-3p; 93-3p; 17-5p; 484; 192-5p; 497-5p; 26a-5p; 503-5p; 195-5p; 941; 501-5p; 23b-3p; 7704; 625-5p; 26b-5p; 432-5p; let-7b-5p; 206; 24-3p; 130b-5p; 671-5p; 454-3p; 329-3p; 145-5p; 377-3p; 376b-3p; 30d-5p; 9-5p; 301a-3p; 129-5p; 204-5p; 500a-5p; 543 |
| Carbohydrate digestion and absorption | 8.47E-07 | 42 | 67 | 27a-5p; 107; 92a-3p; 542-3p; let-7a-5p; 197-3p; 22-3p; 25-3p; 149-5p; 181a-2-3p; 128-3p; 126-3p; 99a-5p; 1-3p; 374a-3p; 130b-3p; 30c-5p; 485-3p; 98-5p; 15a-5p; 34a-5p; 330-5p; 7-5p; 411-5p; 665; 15b-5p; 103a-3p; 184; 409-3p; 320a-3p; 130a-3p; 423-5p; 374b-5p; 181b-5p; 23a-3p; 92b-3p; 136-3p; 93-3p; 17-5p; 484; 192-5p; 497-5p; 26a-5p; 708-5p; 503-5p; 195-5p; 941; 23b-3p; 139-5p; 625-3p; 26b-5p; let-7b-5p; 206; 24-3p; 130b-5p; 671-5p; 454-3p; 329-3p; 99b-5p; 125a-5p; 30d-5p; 193a-5p; 9-5p; 301a-3p; 1307-3p; 296-3p; 378a-3p |
| Salivary secretion | 2.28E-02 | 56 | 67 | 590-3p; let-7e-5p; 92a-3p; let-7d-5p; let-7a-5p; 197-3p; 320b; 22-3p; 486-3p; 25-3p; 181a-2-3p; 128-3p; 99a-5p; 205-5p; 1-3p; 130b-3p; 30c-5p; 98-5p; let-7f-5p; 15a-5p; 34a-5p; 7-5p; 411-5p; 15b-5p; 184; 320a-3p; 382-5p; 130a-3p; 27b-3p; 423-5p; 181b-5p; 92b-3p; 93-3p; 17-5p; 484; 192-5p; 497-5p; 26a-5p; 195-5p; 941; 501-5p; 23b-3p; 877-5p; 625-3p; 625-5p; 26b-5p; 432-5p; let-7b-5p; 206; 24-3p; 130b-5p; 598-3p; 3925-5p; 671-5p; 190a-5p; 454-3p; 329-3p; 376b-3p; 493-3p; 30d-5p; 34c-5p; 9-5p; 301a-3p; 129-5p; 204-5p; 296-3p; 500a-5p |
| Synaptic vesicle cycle | 2.28E-02 | 56 | 67 | 27a-5p; 342-3p; 30a-3p; let-7e-5p; 92a-3p; let-7d-5p; 542-3p; 374a-5p; 31-5p; let-7a-5p; 486-3p; 25-3p; 128-3p; 99a-5p; 323b-3p; 205-5p; 1306-5p; 1-3p; 130b-3p; 574-3p; 30c-5p; 485-3p; 98-5p; let-7f-5p; 15a-5p; 34a-5p; 330-5p; 7-5p; 665; 15b-5p; 103a-3p; 409-3p; 320a-3p; 382-5p; 130a-3p; 423-5p; 374b-5p; 181b-5p; 92b-3p; 17-5p; 484; 192-5p; 421; 497-5p; 503-5p; 195-5p; 501-5p; 23b-3p; 625-5p; 15b-3p; 299-5p; 26b-5p; 432-5p; let-7b-5p; 206; 24-3p; 454-3p; 329-3p; 377-3p; 491-5p; 34c-5p; 9-5p; 301a-3p; 204-5p; 500a-5p; 378a-3p; 543 |
| Other types of O-glycan biosynthesis | 3.09E-04 | 47 | 66 | 107; 342-3p; 590-3p; 378a-5p; 30a-3p; 320c; 320d; 92a-3p; 542-3p; 100-3p; 374a-5p; let-7a-5p; 320b; 486-3p; 25-3p; 128-3p; 99a-5p; 1306-5p; 1-3p; 323a-3p; 30e-5p; 130b-3p; 30c-5p; 98-5p; 15a-5p; 34a-5p; 330-5p; 7-5p; 665; 15b-5p; 103a-3p; 320a-3p; 130a-3p; 27b-3p; 374b-5p; 6724-5p; 92b-3p; 17-5p; 484; 192-5p; 497-5p; 26a-5p; 195-5p; 501-5p; 15b-3p; 26b-5p; 432-5p; let-7b-5p; 130b-5p; 598-3p; 3925-5p; 671-5p; 454-3p; 329-3p; 125a-5p; 99b-3p; 140-5p; 30d-5p; 296-5p; 34c-5p; 9-5p; 301a-3p; 129-5p; 204-5p; 296-3p; 378a-3p |
| Circadian rhythm | 1.43E-02 | 53 | 65 | 107; 590-3p; 1185-1-3p; let-7e-5p; 92a-3p; let-7d-5p; 374a-5p; let-7a-5p; 197-3p; 25-3p; 149-5p; 128-3p; 205-5p; 629-5p; 1-3p; 30e-5p; 130b-3p; 30c-5p; 485-3p; 98-5p; let-7f-5p; 15a-5p; 34a-5p; 411-5p; 665; 576-5p; 15b-5p; 103a-3p; let-7a-3p; 320a-3p; 130a-3p; 27b-3p; 423-5p; 374b-5p; 181b-5p; 92b-3p; 17-5p; 484; 192-5p; 421; 497-5p; 154-3p; 26a-5p; 675-3p; 195-5p; 941; 133a-3p; 127-5p; 3613-5p; 26b-5p; 432-5p; let-7b-5p; 24-3p; 130b-5p; 454-3p; 329-3p; 377-3p; 493-3p; 30d-5p; 9-5p; 301a-3p; 129-5p; 1271-5p; 204-5p; 500a-5p |
| Drug metabolism - other enzymes | 7.04E-05 | 44 | 64 | 27a-5p; 107; 342-3p; 590-3p; 378a-5p; 30a-3p; let-7e-5p; 92a-3p; let-7d-5p; let-7a-5p; 197-3p; 25-3p; 149-5p; 181a-2-3p; 1-3p; 379-5p; 30e-5p; 130b-3p; 30c-5p; 98-5p; let-7f-5p; 15a-5p; 34a-5p; 665; 576-5p; 15b-5p; 103a-3p; 409-3p; 130a-3p; 27b-3p; 423-5p; 374b-5p; 23a-3p; 92b-3p; 17-5p; 484; 192-5p; 497-5p; 26a-5p; 708-5p; 503-5p; 675-3p; 195-5p; 23b-3p; 133a-3p; 127-5p; 664a-5p; 26b-5p; let-7b-5p; 24-3p; 130b-5p; 454-3p; 329-3p; 125a-5p; 99b-3p; 30d-5p; 9-5p; 301a-3p; 129-5p; 376c-3p; 1307-3p; 204-5p; 1287-5p; 378a-3p |
| Cocaine addiction | 3.05E-04 | 45 | 64 | 107; 342-3p; 1185-1-3p; 320c; 320d; 92a-3p; 374a-5p; let-7a-5p; 197-3p; 320b; 22-3p; 25-3p; 149-5p; 128-3p; 126-3p; 323b-3p; 205-5p; 1-3p; 30e-5p; 30c-5p; 15a-5p; 34a-5p; 7-5p; 411-5p; 665; 576-5p; 103a-3p; let-7a-3p; 151a-3p; 320a-3p; 382-5p; 27b-3p; 423-5p; 374b-5p; 181b-5p; 23a-3p; 92b-3p; 93-3p; 17-5p; 501-5p; 23b-3p; 133a-3p; 139-5p; 877-5p; 625-5p; 26b-5p; 432-5p; let-7b-5p; 206; 130b-5p; 3925-5p; 671-5p; 329-3p; 145-5p; 125a-5p; 376b-3p; 30d-5p; 491-5p; 9-5p; 129-5p; 628-5p; 204-5p; 500a-5p; 1185-5p |
| Ferroptosis | 7.13E-03 | 50 | 64 | 342-3p; 1185-1-3p; let-7e-5p; 320c; 92a-3p; let-7d-5p; 374a-5p; let-7a-5p; 22-3p; 25-3p; 128-3p; 205-5p; 1-3p; 30e-5p; 130b-3p; 30c-5p; 485-3p; 98-5p; let-7f-5p; 15a-5p; 34a-5p; 7-5p; 665; 15b-5p; 191-5p; 409-3p; 320a-3p; 382-5p; 130a-3p; 423-5p; 374b-5p; 181b-5p; 92b-3p; 93-3p; 17-5p; 484; 192-5p; 26a-5p; 328-3p; 941; 133a-3p; 625-5p; 664a-5p; 299-5p; 26b-5p; let-7b-5p; 24-3p; 130b-5p; 454-3p; 329-3p; 145-5p; 125a-5p; 377-3p; 30d-5p; 491-5p; 9-5p; 301a-3p; 129-5p; 1307-3p; 769-5p; 204-5p; 296-3p; 125b-1-3p; 378a-3p |
| Glycerolipid metabolism | 1.56E-02 | 52 | 64 | 342-3p; 590-3p; 378a-5p; 30a-3p; let-7e-5p; 92a-3p; let-7d-5p; 31-5p; let-7a-5p; 197-3p; 149-5p; 128-3p; 205-5p; 1306-5p; 1-3p; 30e-5p; 130b-3p; 30c-5p; 98-5p; let-7f-5p; 15a-5p; 34a-5p; 7-5p; 411-5p; 665; 15b-5p; 184; 130a-3p; 27b-3p; 423-5p; 628-3p; 181b-5p; 93-3p; 17-5p; 484; 192-5p; 421; 497-5p; 26a-5p; 328-3p; 195-5p; 877-5p; 625-3p; 127-5p; 664a-5p; 26b-5p; 432-5p; let-7b-5p; 24-3p; 130b-5p; 454-3p; 889-3p; 99b-5p; 145-5p; 125a-5p; 377-3p; 30d-5p; 9-5p; 301a-3p; 129-5p; 1307-3p; 769-5p; 204-5p; 378a-3p |
| Pyrimidine metabolism | 4.69E-03 | 49 | 63 | 27a-5p; 107; 342-3p; 590-3p; 378a-5p; 30a-3p; let-7e-5p; 92a-3p; let-7d-5p; 542-3p; 132-5p; let-7a-5p; 197-3p; 486-3p; 25-3p; 149-5p; 181a-2-3p; 128-3p; 99a-5p; 1-3p; 379-5p; 374a-3p; 30e-5p; 130b-3p; 30c-5p; 98-5p; let-7f-5p; 15a-5p; 34a-5p; 7-5p; 665; 15b-5p; 103a-3p; 320a-3p; 27b-3p; 423-5p; 374b-5p; 92b-3p; 17-5p; 484; 192-5p; 497-5p; 26a-5p; 708-5p; 675-3p; 195-5p; 133a-3p; 127-5p; 664a-5p; 26b-5p; let-7b-5p; 206; 24-3p; 130b-5p; 671-5p; 125a-5p; 30d-5p; 9-5p; 301a-3p; 628-5p; 204-5p; 1287-5p; 378a-3p |
| Base excision repair | 7.26E-04 | 44 | 61 | 27a-5p; 107; 378a-5p; let-7e-5p; 92a-3p; let-7d-5p; 542-3p; 31-5p; let-7a-5p; 22-3p; 486-3p; 149-5p; 128-3p; 99a-5p; 205-5p; 1-3p; 379-5p; 130b-3p; 98-5p; let-7f-5p; 15a-5p; 34a-5p; 7-5p; 665; 15b-5p; 103a-3p; 320a-3p; 130a-3p; 27b-3p; 423-5p; 181b-5p; 92b-3p; 93-3p; 17-5p; 484; 192-5p; 497-5p; 26a-5p; 328-3p; 708-5p; 195-5p; 501-5p; 877-5p; 625-5p; 299-5p; 26b-5p; let-7b-5p; 24-3p; 130b-5p; 3925-5p; 671-5p; 454-3p; 125a-5p; 140-5p; 34c-5p; 301a-3p; 129-5p; 628-5p; 1307-3p; 1271-5p; 500a-5p |
| Various types of N-glycan biosynthesis | 5.45E-05 | 40 | 60 | 378a-5p; let-7e-5p; 92a-3p; let-7a-5p; 197-3p; 22-3p; 486-3p; 25-3p; 149-5p; 128-3p; 323b-3p; 1306-5p; 1-3p; 30e-5p; 130b-3p; 30c-5p; 98-5p; 15a-5p; 34a-5p; 7-5p; 665; 15b-5p; 103a-3p; let-7a-3p; 409-3p; 320a-3p; 382-5p; 130a-3p; 27b-3p; 423-5p; 181b-5p; 6724-5p; 23a-3p; 22-5p; 92b-3p; 484; 192-5p; 26a-5p; 328-3p; 195-5p; 23b-3p; 664a-5p; 26b-5p; 432-5p; let-7b-5p; 24-3p; 130b-5p; 598-3p; 454-3p; 145-5p; 125a-5p; 377-3p; 30d-5p; 296-5p; 301a-3p; 129-5p; 769-5p; 204-5p; 296-3p; 378a-3p |
| SNARE interactions in vesicular transport | 1.55E-04 | 41 | 60 | 27a-5p; 107; 483-5p; 342-3p; 590-3p; 378a-5p; 1185-1-3p; let-7e-5p; 92a-3p; let-7d-5p; 100-3p; 374a-5p; let-7a-5p; 22-3p; 205-5p; 1-3p; 30e-5p; 130b-3p; 30c-5p; 98-5p; let-7f-5p; 15a-5p; 34a-5p; 330-5p; 7-5p; 411-5p; 665; 576-5p; 15b-5p; 103a-3p; 320a-3p; 382-5p; 130a-3p; 374b-5p; 181b-5p; 6724-5p; 136-3p; 17-5p; 192-5p; 421; 497-5p; 708-5p; 195-5p; 877-5p; 15b-3p; 299-5p; 26b-5p; let-7b-5p; 206; 24-3p; 130b-5p; 454-3p; 377-3p; 30d-5p; 296-5p; 491-5p; 9-5p; 301a-3p; 129-5p; 543 |
| Pyruvate metabolism | 7.92E-04 | 42 | 59 | 590-3p; 1185-1-3p; 30a-3p; let-7e-5p; 320c; 92a-3p; let-7d-5p; 374a-5p; let-7a-5p; 197-3p; 22-3p; 25-3p; 128-3p; 99a-5p; 205-5p; 1-3p; 30c-5p; 98-5p; let-7f-5p; 34a-5p; 330-5p; 7-5p; 576-5p; 15b-5p; 191-5p; 184; 320a-3p; 27b-3p; 423-5p; 181b-5p; 23a-3p; 92b-3p; 17-5p; 484; 192-5p; 497-5p; 26a-5p; 328-3p; 23b-3p; 133a-3p; 625-5p; 664a-5p; 15b-3p; 26b-5p; let-7b-5p; 24-3p; 130b-5p; 190a-5p; 99b-5p; 125a-5p; 377-3p; 30d-5p; 491-5p; 34c-5p; 9-5p; 129-5p; 769-5p; 296-3p; 378a-3p |
| Ether lipid metabolism | 1.12E-02 | 46 | 59 | 107; 342-3p; 590-3p; 378a-5p; 30a-3p; let-7e-5p; 92a-3p; let-7d-5p; 542-3p; 100-3p; 374a-5p; let-7a-5p; 128-3p; 205-5p; 1-3p; 323a-3p; 379-5p; 30e-5p; 130b-3p; 30c-5p; 98-5p; let-7f-5p; 15a-5p; 34a-5p; 7-5p; 665; 15b-5p; 103a-3p; 184; 320a-3p; 130a-3p; 27b-3p; 181b-5p; 23a-3p; 92b-3p; 17-5p; 484; 192-5p; 497-5p; 26a-5p; 195-5p; 23b-3p; 877-5p; 625-3p; 625-5p; 26b-5p; let-7b-5p; 24-3p; 454-3p; 329-3p; 99b-5p; 145-5p; 125a-5p; 377-3p; 30d-5p; 34c-5p; 9-5p; 301a-3p; 129-5p |
| Nucleotide excision repair | 3.26E-05 | 37 | 58 | 133a-5p; 107; 483-5p; 342-3p; 378a-5p; let-7e-5p; 92a-3p; let-7d-5p; 542-3p; 31-5p; let-7a-5p; 197-3p; 25-3p; 149-5p; 99a-5p; 1306-5p; 1-3p; 30e-5p; 130b-3p; 30c-5p; 98-5p; let-7f-5p; 15a-5p; 34a-5p; 7-5p; 411-5p; 665; 15b-5p; 103a-3p; 320a-3p; 130a-3p; 27b-3p; 17-5p; 484; 192-5p; 497-5p; 26a-5p; 195-5p; 941; 501-5p; 23b-3p; 877-5p; 127-5p; 625-5p; 299-5p; 26b-5p; let-7b-5p; 24-3p; 130b-5p; 454-3p; 329-3p; 145-5p; 493-3p; 30d-5p; 9-5p; 301a-3p; 129-5p; 296-3p |
| Aminoacyl-tRNA biosynthesis | 1.46E-04 | 39 | 58 | 483-5p; 342-3p; 590-3p; 378a-5p; let-7e-5p; 92a-3p; let-7d-5p; let-7a-5p; 197-3p; 486-3p; 149-5p; 181a-2-3p; 128-3p; 99a-5p; 323b-3p; 1306-5p; 130b-3p; 98-5p; let-7f-5p; 7-5p; 411-5p; 665; 15b-5p; 191-5p; 320a-3p; 130a-3p; 27b-3p; 423-5p; 6724-5p; 584-5p; 92b-3p; 93-3p; 17-5p; 484; 192-5p; 421; 26a-5p; 503-5p; 23b-3p; 664a-5p; 26b-5p; let-7b-5p; 24-3p; 130b-5p; 454-3p; 377-3p; 493-3p; 30d-5p; 296-5p; 193a-5p; 9-5p; 301a-3p; 129-5p; 1307-3p; 769-5p; 204-5p; 296-3p; 543 |
| Complement and coagulation cascades | 1.22E-02 | 46 | 58 | 342-3p; 181a-3p; 378a-5p; 30a-3p; let-7e-5p; 92a-3p; let-7d-5p; 542-3p; let-7a-5p; 197-3p; 149-5p; 128-3p; 205-5p; 1306-5p; 1-3p; 323a-3p; 379-5p; 30e-5p; 130b-3p; 30c-5p; 98-5p; let-7f-5p; 15a-5p; 330-5p; 7-5p; 665; 576-5p; 15b-5p; 409-3p; 22-5p; 17-5p; 484; 192-5p; 421; 497-5p; 26a-5p; 195-5p; 23b-3p; 127-5p; 664a-5p; 6511a-3p; 26b-5p; 432-5p; let-7b-5p; 206; 130b-5p; 3925-5p; 329-3p; 145-5p; 125a-5p; 30d-5p; 9-5p; 301a-3p; 129-5p; 376c-3p; 1307-3p; 769-5p; 204-5p |
| Arginine and proline metabolism | 4.07E-04 | 38 | 55 | 107; 483-5p; 342-3p; 30a-3p; let-7e-5p; 92a-3p; let-7d-5p; 542-3p; let-7a-5p; 22-3p; 25-3p; 149-5p; 128-3p; 205-5p; 1306-5p; 1-3p; 379-5p; 30e-5p; 98-5p; let-7f-5p; 15a-5p; 34a-5p; 7-5p; 665; 576-5p; 103a-3p; 151a-3p; 320a-3p; 423-5p; 181b-5p; 92b-3p; 93-3p; 17-5p; 484; 192-5p; 497-5p; 26a-5p; 328-3p; 941; 26b-5p; let-7b-5p; 24-3p; 130b-5p; 671-5p; 99b-5p; 145-5p; 377-3p; 493-3p; 30d-5p; 9-5p; 129-5p; 628-5p; 769-5p; 500a-5p; 543 |
| Malaria | 1.28E-03 | 39 | 55 | 107; 378a-5p; 30a-3p; let-7e-5p; 320c; 92a-3p; let-7d-5p; 100-3p; 31-5p; let-7a-5p; 197-3p; 149-5p; 126-3p; 205-5p; 1-3p; 574-3p; 98-5p; let-7f-5p; 15a-5p; 34a-5p; 7-5p; 665; 15b-5p; 409-3p; 320a-3p; 130a-3p; 27b-3p; 423-5p; 181b-5p; 23a-3p; 22-5p; 92b-3p; 17-5p; 26a-5p; 503-5p; 675-3p; 23b-3p; 133a-3p; 139-5p; 625-5p; 26b-5p; let-7b-5p; 206; 24-3p; 130b-5p; 329-3p; 145-5p; 125a-5p; 491-5p; 34c-5p; 9-5p; 1307-3p; 204-5p; 296-3p; 378a-3p |
| DNA replication | 1.40E-04 | 35 | 54 | 133a-5p; 107; 378a-5p; 1185-1-3p; let-7e-5p; 92a-3p; let-7d-5p; 542-3p; 31-5p; let-7a-5p; 197-3p; 25-3p; 149-5p; 1-3p; 30e-5p; 130b-3p; 30c-5p; 98-5p; let-7f-5p; 15a-5p; 34a-5p; 7-5p; 665; 15b-5p; 103a-3p; let-7a-3p; 320a-3p; 130a-3p; 423-5p; 93-3p; 17-5p; 484; 192-5p; 328-3p; 503-5p; 675-3p; 195-5p; 501-5p; 877-5p; 625-5p; 299-5p; 26b-5p; let-7b-5p; 24-3p; 130b-5p; 454-3p; 99b-5p; 145-5p; 140-5p; 30d-5p; 301a-3p; 129-5p; 628-5p; 500a-5p |
| Autophagy - other | 1.13E-03 | 38 | 54 | 107; 342-3p; 30a-3p; let-7e-5p; 320c; 92a-3p; let-7d-5p; 374a-5p; let-7a-5p; 197-3p; 149-5p; 128-3p; 99a-5p; 30e-5p; 30c-5p; 98-5p; let-7f-5p; 15a-5p; 34a-5p; 7-5p; 15b-5p; 103a-3p; 382-5p; 130a-3p; 27b-3p; 423-5p; 374b-5p; 181b-5p; 584-5p; 17-5p; 192-5p; 421; 497-5p; 26a-5p; 708-5p; 503-5p; 195-5p; 23b-3p; 139-5p; 299-5p; 26b-5p; let-7b-5p; 24-3p; 130b-5p; 671-5p; 329-3p; 889-3p; 99b-5p; 125a-5p; 376b-3p; 30d-5p; 193a-5p; 9-5p; 129-5p |
| Homologous recombination | 1.13E-03 | 38 | 54 | 133a-5p; 107; 590-3p; 378a-5p; let-7e-5p; 92a-3p; 374a-5p; 31-5p; 197-3p; 25-3p; 149-5p; 128-3p; 99a-5p; 1306-5p; 1-3p; 30e-5p; 130b-3p; 30c-5p; 485-3p; 15a-5p; 34a-5p; 7-5p; 665; 103a-3p; 127-3p; 320a-3p; 27b-3p; 181b-5p; 23a-3p; 92b-3p; 17-5p; 484; 192-5p; 421; 26a-5p; 328-3p; 501-5p; 23b-3p; 625-5p; 26b-5p; 432-5p; let-7b-5p; 24-3p; 130b-5p; 3925-5p; 145-5p; 377-3p; 99b-3p; 493-3p; 30d-5p; 491-5p; 376c-3p; 769-5p; 543 |
| Fatty acid degradation | 1.40E-03 | 38 | 54 | 342-3p; 30a-3p; let-7e-5p; 92a-3p; let-7d-5p; 374a-5p; let-7a-5p; 197-3p; 128-3p; 99a-5p; 205-5p; 1-3p; 130b-3p; 98-5p; let-7f-5p; 15a-5p; 34a-5p; 7-5p; 665; 15b-5p; let-7a-3p; 191-5p; 151a-3p; 130a-3p; 374b-5p; 181b-5p; 92b-3p; 93-3p; 17-5p; 484; 192-5p; 421; 497-5p; 26a-5p; 328-3p; 195-5p; 23b-3p; 877-5p; 26b-5p; let-7b-5p; 24-3p; 130b-5p; 454-3p; 99b-5p; 377-3p; 491-5p; 9-5p; 301a-3p; 129-5p; 628-5p; 376c-3p; 1307-3p; 204-5p; 378a-3p |
| Proteasome | 3.21E-03 | 40 | 54 | 107; 378a-5p; let-7e-5p; 92a-3p; let-7a-5p; 25-3p; 149-5p; 99a-5p; 1-3p; 30e-5p; 130b-3p; 30c-5p; 98-5p; 15a-5p; 7-5p; 665; 15b-5p; 191-5p; 151a-3p; 409-3p; 320a-3p; 130a-3p; 423-5p; 6724-5p; 23a-3p; 92b-3p; 93-3p; 484; 192-5p; 421; 26a-5p; 195-5p; 23b-3p; 133a-3p; 139-5p; 877-5p; 625-5p; 26b-5p; let-7b-5p; 206; 24-3p; 130b-5p; 671-5p; 454-3p; 125a-5p; 493-3p; 30d-5p; 296-5p; 301a-3p; 129-5p; 1307-3p; 769-5p; 1287-5p; 378a-3p |
| Graft-versus-host disease | 1.04E-06 | 30 | 53 | 107; 30a-3p; let-7e-5p; 92a-3p; let-7a-5p; 25-3p; 149-5p; 128-3p; 1306-5p; 1-3p; 30e-5p; 130b-3p; 30c-5p; 485-3p; 98-5p; let-7f-5p; 15a-5p; 34a-5p; 330-5p; 576-5p; 15b-5p; 191-5p; 151a-3p; 409-3p; 320a-3p; 130a-3p; 27b-3p; 423-5p; 181b-5p; 23a-3p; 22-5p; 92b-3p; 17-5p; 484; 26a-5p; 501-5p; 7704; 26b-5p; 24-3p; 130b-5p; 454-3p; 329-3p; 145-5p; 125a-5p; 99b-3p; 30d-5p; 491-5p; 9-5p; 301a-3p; 628-5p; 204-5p; 296-3p; 500a-5p |
| Valine, leucine and isoleucine degradation | 2.11E-03 | 38 | 53 | 378a-5p; 30a-3p; let-7e-5p; 92a-3p; 374a-5p; let-7a-5p; 197-3p; 25-3p; 149-5p; 128-3p; 99a-5p; 1-3p; 130b-3p; 98-5p; let-7f-5p; 34a-5p; 7-5p; 665; 15b-5p; 191-5p; 151a-3p; 320a-3p; 130a-3p; 181b-5p; 23a-3p; 92b-3p; 17-5p; 484; 192-5p; 421; 497-5p; 26a-5p; 328-3p; 503-5p; 23b-3p; 877-5p; 664a-5p; 15b-3p; 26b-5p; let-7b-5p; 24-3p; 130b-5p; 454-3p; 329-3p; 99b-5p; 125a-5p; 377-3p; 9-5p; 301a-3p; 628-5p; 769-5p; 204-5p; 378a-3p |
| RNA polymerase | 1.35E-02 | 41 | 53 | 483-5p; 342-3p; 590-3p; 378a-5p; let-7e-5p; 362-5p; 92a-3p; let-7d-5p; 374a-5p; let-7a-5p; 197-3p; 486-3p; 149-5p; 128-3p; 1306-5p; 1-3p; 374a-3p; 30e-5p; 130b-3p; 30c-5p; 98-5p; let-7f-5p; 15a-5p; 34a-5p; 7-5p; 665; 15b-5p; 320a-3p; 130a-3p; 423-5p; 374b-5p; 584-5p; 136-3p; 17-5p; 484; 421; 497-5p; 26a-5p; 328-3p; 195-5p; 501-5p; 7704; 26b-5p; let-7b-5p; 24-3p; 130b-5p; 454-3p; 30d-5p; 296-5p; 9-5p; 301a-3p; 129-5p; 500a-5p |
| African trypanosomiasis | 4.70E-06 | 30 | 52 | 107; 590-3p; let-7e-5p; 92a-3p; 31-5p; let-7a-5p; 197-3p; 25-3p; 149-5p; 128-3p; 126-3p; 1306-5p; 1-3p; 130b-3p; 30c-5p; 485-3p; 98-5p; let-7f-5p; 15a-5p; 34a-5p; 665; 15b-5p; 184; 409-3p; 320a-3p; 130a-3p; 423-5p; 181b-5p; 23a-3p; 22-5p; 92b-3p; 93-3p; 17-5p; 484; 192-5p; 26a-5p; 501-5p; 23b-3p; 6511a-3p; 26b-5p; let-7b-5p; 24-3p; 130b-5p; 454-3p; 329-3p; 125a-5p; 9-5p; 301a-3p; 129-5p; 204-5p; 296-3p; 500a-5p |
| Glycine, serine and threonine metabolism | 2.47E-04 | 34 | 52 | 107; 483-5p; 342-3p; 590-3p; let-7e-5p; 92a-3p; let-7d-5p; 542-3p; let-7a-5p; 197-3p; 22-3p; 486-3p; 25-3p; 149-5p; 128-3p; 99a-5p; 323b-3p; 205-5p; 1-3p; 98-5p; let-7f-5p; 15a-5p; 34a-5p; 7-5p; 665; 15b-5p; 423-5p; 92b-3p; 93-3p; 17-5p; 484; 192-5p; 421; 497-5p; 26a-5p; 503-5p; 195-5p; 139-5p; 625-5p; 26b-5p; let-7b-5p; 671-5p; 190a-5p; 145-5p; 377-3p; 193a-5p; 34c-5p; 129-5p; 628-5p; 769-5p; 296-3p; 500a-5p |
| Basal transcription factors | 2.30E-03 | 37 | 52 | 107; 342-3p; 378a-5p; let-7e-5p; 92a-3p; 542-3p; 100-3p; 31-5p; let-7a-5p; 197-3p; 25-3p; 99a-5p; 205-5p; 1-3p; 30e-5p; 30c-5p; 98-5p; let-7f-5p; 15a-5p; 7-5p; 411-5p; 665; 15b-5p; 103a-3p; 320a-3p; 423-5p; 374b-5p; 181b-5p; 92b-3p; 136-3p; 17-5p; 484; 192-5p; 421; 497-5p; 26a-5p; 195-5p; 501-5p; 23b-3p; 139-5p; 877-5p; 15b-3p; 26b-5p; let-7b-5p; 24-3p; 671-5p; 329-3p; 30d-5p; 9-5p; 129-5p; 204-5p; 500a-5p |
| ABC transporters | 3.82E-03 | 38 | 52 | 320c; 320d; 92a-3p; 100-3p; 374a-5p; 31-5p; 197-3p; 320b; 181a-2-3p; 128-3p; 1-3p; 130b-3p; 98-5p; 15a-5p; 330-5p; 7-5p; 411-5p; 665; 576-5p; 15b-5p; let-7a-3p; 320a-3p; 130a-3p; 27b-3p; 423-5p; 374b-5p; 23a-3p; 92b-3p; 17-5p; 484; 192-5p; 497-5p; 26a-5p; 328-3p; 195-5p; 23b-3p; 625-5p; 15b-3p; 26b-5p; let-7b-5p; 24-3p; 130b-5p; 454-3p; 329-3p; 889-3p; 145-5p; 491-5p; 9-5p; 301a-3p; 129-5p; 1307-3p; 204-5p |
| Intestinal immune network for IgA production | 3.76E-03 | 37 | 51 | 107; 30a-3p; let-7e-5p; 92a-3p; let-7d-5p; 31-5p; let-7a-5p; 25-3p; 149-5p; 181a-2-3p; 128-3p; 126-3p; 1-3p; 574-3p; 30c-5p; 98-5p; let-7f-5p; 34a-5p; 7-5p; 665; 576-5p; 15b-5p; 184; 130a-3p; 27b-3p; 423-5p; 23a-3p; 22-5p; 92b-3p; 17-5p; 484; 192-5p; 26a-5p; 503-5p; 7704; 133a-3p; 139-5p; 625-5p; 3613-5p; 26b-5p; let-7b-5p; 24-3p; 130b-5p; 671-5p; 454-3p; 329-3p; 145-5p; 491-5p; 9-5p; 129-5p; 204-5p |
| One carbon pool by folate | 1.73E-05 | 30 | 50 | 133a-5p; 342-3p; 378a-5p; 30a-3p; 92a-3p; let-7a-5p; 197-3p; 22-3p; 486-3p; 25-3p; 149-5p; 181a-2-3p; 99a-5p; 1306-5p; 629-5p; 1-3p; 30e-5p; 30c-5p; 15a-5p; 34a-5p; 330-5p; 665; 576-5p; 15b-5p; 103a-3p; 320a-3p; 382-5p; 423-5p; 6724-5p; 93-3p; 484; 192-5p; 421; 497-5p; 503-5p; 195-5p; 501-5p; 877-5p; 625-5p; 26b-5p; let-7b-5p; 24-3p; 130b-5p; 99b-5p; 30d-5p; 296-5p; 34c-5p; 9-5p; 769-5p; 500a-5p |
| Type I diabetes mellitus | 3.30E-04 | 33 | 50 | 342-3p; 30a-3p; let-7e-5p; 92a-3p; 197-3p; 25-3p; 149-5p; 128-3p; 1306-5p; 1-3p; 30e-5p; 30c-5p; 485-3p; 98-5p; 15a-5p; 34a-5p; 330-5p; 665; 576-5p; 15b-5p; 191-5p; 409-3p; 320a-3p; 130a-3p; 27b-3p; 423-5p; 181b-5p; 23a-3p; 22-5p; 92b-3p; 17-5p; 484; 26a-5p; 7704; 6511a-3p; 26b-5p; 432-5p; 24-3p; 130b-5p; 329-3p; 145-5p; 125a-5p; 30d-5p; 491-5p; 9-5p; 129-5p; 628-5p; 1271-5p; 204-5p; 296-3p |
| Hippo signaling pathway - multiple species | 2.02E-02 | 39 | 50 | 107; 590-3p; 92a-3p; 542-3p; 374a-5p; 31-5p; let-7a-5p; 197-3p; 22-3p; 25-3p; 149-5p; 181a-2-3p; 128-3p; 629-5p; 1-3p; 374a-3p; 130b-3p; 30c-5p; 98-5p; 15a-5p; 330-5p; 7-5p; 411-5p; 15b-5p; 103a-3p; 320a-3p; 130a-3p; 423-5p; 374b-5p; 181b-5p; 92b-3p; 17-5p; 484; 192-5p; 497-5p; 708-5p; 195-5p; 877-5p; 26b-5p; let-7b-5p; 24-3p; 130b-5p; 454-3p; 329-3p; 125a-5p; 377-3p; 9-5p; 301a-3p; 129-5p; 769-5p |
| Porphyrin and chlorophyll metabolism | 3.63E-03 | 35 | 49 | 342-3p; 378a-5p; 30a-3p; let-7e-5p; 362-5p; 92a-3p; let-7d-5p; 374a-5p; let-7a-5p; 25-3p; 181a-2-3p; 128-3p; 1-3p; 130b-3p; 30c-5p; 98-5p; let-7f-5p; 15a-5p; 411-5p; 665; 15b-5p; 409-3p; 320a-3p; 130a-3p; 374b-5p; 181b-5p; 92b-3p; 17-5p; 484; 192-5p; 497-5p; 503-5p; 195-5p; 501-5p; 133a-3p; 127-5p; 26b-5p; let-7b-5p; 24-3p; 130b-5p; 454-3p; 329-3p; 145-5p; 377-3p; 301a-3p; 129-5p; 376c-3p; 1307-3p; 204-5p |
| Fructose and mannose metabolism | 4.02E-03 | 35 | 49 | 378a-5p; 1185-1-3p; 92a-3p; 542-3p; 197-3p; 320b; 149-5p; 128-3p; 1306-5p; 98-5p; 15a-5p; 34a-5p; 330-5p; 665; 15b-5p; 320a-3p; 382-5p; 27b-3p; 423-5p; 628-3p; 181b-5p; 22-5p; 92b-3p; 93-3p; 17-5p; 484; 192-5p; 497-5p; 328-3p; 195-5p; 23b-3p; 133a-3p; 877-5p; 664a-5p; 26b-5p; let-7b-5p; 24-3p; 99b-5p; 145-5p; 125a-5p; 377-3p; 493-3p; 491-5p; 9-5p; 129-5p; 1307-3p; 1271-5p; 204-5p; 378a-3p |
| Glutathione metabolism | 6.55E-03 | 36 | 49 | 342-3p; 590-3p; 30a-3p; let-7e-5p; 92a-3p; let-7d-5p; let-7a-5p; 197-3p; 149-5p; 1306-5p; 1-3p; 379-5p; 30e-5p; 30c-5p; 98-5p; let-7f-5p; 15a-5p; 34a-5p; 7-5p; 665; 15b-5p; 320a-3p; 423-5p; 92b-3p; 17-5p; 484; 192-5p; 497-5p; 26a-5p; 503-5p; 195-5p; 501-5p; 133a-3p; 127-5p; 625-5p; 664a-5p; 6511a-3p; 26b-5p; let-7b-5p; 206; 24-3p; 329-3p; 125a-5p; 377-3p; 99b-3p; 30d-5p; 491-5p; 1307-3p; 500a-5p |
| Amino sugar and nucleotide sugar metabolism | 7.19E-03 | 36 | 49 | 483-5p; 342-3p; 590-3p; 378a-5p; 92a-3p; 374a-5p; 197-3p; 320b; 25-3p; 149-5p; 128-3p; 1-3p; 30e-5p; 30c-5p; 98-5p; 15a-5p; 34a-5p; 411-5p; 665; 576-5p; 409-3p; 320a-3p; 27b-3p; 181b-5p; 92b-3p; 93-3p; 17-5p; 484; 192-5p; 421; 328-3p; 23b-3p; 877-5p; 664a-5p; 6511a-3p; 26b-5p; let-7b-5p; 24-3p; 671-5p; 329-3p; 99b-5p; 125a-5p; 30d-5p; 9-5p; 129-5p; 1307-3p; 769-5p; 296-3p; 378a-3p |
| Galactose metabolism | 9.83E-07 | 26 | 48 | 92a-3p; 197-3p; 149-5p; 128-3p; 1306-5p; 1-3p; 30e-5p; 130b-3p; 30c-5p; 485-3p; 98-5p; 15a-5p; 34a-5p; 330-5p; 665; 576-5p; 15b-5p; 409-3p; 320a-3p; 130a-3p; 628-3p; 181b-5p; 6724-5p; 23a-3p; 92b-3p; 136-3p; 17-5p; 484; 497-5p; 195-5p; 23b-3p; 26b-5p; let-7b-5p; 598-3p; 671-5p; 454-3p; 329-3p; 99b-5p; 145-5p; 125a-5p; 30d-5p; 296-5p; 9-5p; 301a-3p; 129-5p; 1307-3p; 769-5p; 296-3p |
| Mucin type O-glycan biosynthesis | 2.56E-04 | 31 | 48 | 342-3p; 590-3p; 378a-5p; 30a-3p; 92a-3p; 374a-5p; let-7a-5p; 25-3p; 205-5p; 323a-3p; 30e-5p; 130b-3p; 30c-5p; 98-5p; 15a-5p; 34a-5p; 7-5p; 665; 15b-5p; 130a-3p; 27b-3p; 374b-5p; 92b-3p; 484; 192-5p; 497-5p; 26a-5p; 503-5p; 195-5p; 501-5p; 26b-5p; 432-5p; let-7b-5p; 130b-5p; 454-3p; 329-3p; 125a-5p; 99b-3p; 140-5p; 30d-5p; 491-5p; 34c-5p; 9-5p; 301a-3p; 129-5p; 1307-3p; 204-5p; 378a-3p |
| Collecting duct acid secretion | 4.98E-04 | 32 | 48 | 342-3p; 378a-5p; 30a-3p; let-7e-5p; let-7d-5p; 542-3p; 374a-5p; let-7a-5p; 197-3p; 486-3p; 128-3p; 323b-3p; 1306-5p; 1-3p; 130b-3p; 30c-5p; 98-5p; let-7f-5p; 34a-5p; 665; 576-5p; 15b-5p; 320a-3p; 130a-3p; 423-5p; 374b-5p; 6724-5p; 17-5p; 484; 192-5p; 421; 26a-5p; 503-5p; 23b-3p; 299-5p; 26b-5p; let-7b-5p; 24-3p; 130b-5p; 454-3p; 329-3p; 377-3p; 99b-3p; 296-5p; 34c-5p; 9-5p; 301a-3p; 129-5p |
| Retinol metabolism | 3.18E-02 | 38 | 48 | 107; 590-3p; let-7e-5p; 92a-3p; let-7d-5p; 542-3p; let-7a-5p; 197-3p; 25-3p; 128-3p; 130b-3p; 98-5p; let-7f-5p; 15a-5p; 34a-5p; 7-5p; 411-5p; 665; 576-5p; 15b-5p; 103a-3p; let-7a-3p; 409-3p; 130a-3p; 27b-3p; 628-3p; 17-5p; 484; 497-5p; 503-5p; 195-5p; 133a-3p; 26b-5p; let-7b-5p; 24-3p; 130b-5p; 337-3p; 454-3p; 329-3p; 376b-3p; 140-5p; 34c-5p; 301a-3p; 129-5p; 376c-3p; 204-5p; 296-3p; 378a-3p |
| Starch and sucrose metabolism | 1.83E-06 | 26 | 47 | 133a-5p; 378a-5p; let-7e-5p; 92a-3p; let-7d-5p; 100-3p; 374a-5p; 31-5p; let-7a-5p; 149-5p; 128-3p; 1-3p; 323a-3p; 130b-3p; 485-3p; 98-5p; let-7f-5p; 15a-5p; 34a-5p; 330-5p; 665; 576-5p; 191-5p; 409-3p; 130a-3p; 27b-3p; 181b-5p; 23a-3p; 92b-3p; 136-3p; 17-5p; 484; 192-5p; 23b-3p; 664a-5p; 26b-5p; let-7b-5p; 24-3p; 130b-5p; 671-5p; 454-3p; 329-3p; 99b-5p; 125a-5p; 301a-3p; 1307-3p; 296-3p |
| Chemical carcinogenesis | 1.43E-03 | 32 | 47 | 107; 1185-1-3p; 30a-3p; let-7e-5p; 92a-3p; let-7d-5p; 374a-5p; let-7a-5p; 197-3p; 25-3p; 181a-2-3p; 128-3p; 1-3p; 323a-3p; 130b-3p; 98-5p; let-7f-5p; 15a-5p; 7-5p; 665; 15b-5p; 103a-3p; let-7a-3p; 409-3p; 27b-3p; 374b-5p; 92b-3p; 93-3p; 484; 497-5p; 26a-5p; 503-5p; 195-5p; 133a-3p; 26b-5p; let-7b-5p; 24-3p; 130b-5p; 329-3p; 145-5p; 99b-3p; 9-5p; 129-5p; 376c-3p; 1307-3p; 204-5p; 296-3p |
| Tryptophan metabolism | 1.95E-03 | 32 | 46 | 378a-5p; 30a-3p; 92a-3p; 374a-5p; let-7a-5p; 22-3p; 181a-2-3p; 128-3p; 99a-5p; 205-5p; 1-3p; 379-5p; let-7f-5p; 34a-5p; 7-5p; 15b-5p; 103a-3p; 151a-3p; 27b-3p; 374b-5p; 181b-5p; 92b-3p; 17-5p; 484; 192-5p; 421; 497-5p; 328-3p; 503-5p; 23b-3p; 877-5p; 26b-5p; let-7b-5p; 24-3p; 130b-5p; 671-5p; 329-3p; 99b-5p; 145-5p; 125a-5p; 377-3p; 9-5p; 129-5p; 628-5p; 296-3p; 500a-5p |
| Metabolism of xenobiotics by cytochrome P450 | 9.14E-03 | 34 | 46 | 107; 1185-1-3p; 30a-3p; let-7e-5p; 92a-3p; let-7d-5p; let-7a-5p; 197-3p; 25-3p; 181a-2-3p; 128-3p; 1-3p; 323a-3p; 130b-3p; 98-5p; let-7f-5p; 15a-5p; 34a-5p; 7-5p; 15b-5p; 103a-3p; let-7a-3p; 409-3p; 27b-3p; 92b-3p; 93-3p; 17-5p; 484; 497-5p; 503-5p; 195-5p; 7704; 133a-3p; 26b-5p; let-7b-5p; 24-3p; 130b-5p; 329-3p; 145-5p; 99b-3p; 9-5p; 129-5p; 376c-3p; 1307-3p; 204-5p; 296-3p |
| Drug metabolism - cytochrome P450 | 2.05E-04 | 28 | 45 | 107; 1185-1-3p; 30a-3p; let-7e-5p; 92a-3p; let-7d-5p; let-7a-5p; 197-3p; 22-3p; 25-3p; 128-3p; 205-5p; 1-3p; 130b-3p; 98-5p; let-7f-5p; 15a-5p; 7-5p; 665; 15b-5p; 103a-3p; let-7a-3p; 409-3p; 320a-3p; 27b-3p; 92b-3p; 497-5p; 503-5p; 195-5p; 133a-3p; 26b-5p; let-7b-5p; 24-3p; 130b-5p; 671-5p; 329-3p; 145-5p; 99b-3p; 9-5p; 129-5p; 628-5p; 376c-3p; 1307-3p; 204-5p; 500a-5p |
| Mismatch repair | 1.59E-05 | 25 | 44 | 133a-5p; 107; 590-3p; 1185-1-3p; let-7e-5p; 92a-3p; let-7d-5p; 542-3p; 31-5p; let-7a-5p; 149-5p; 1-3p; 30e-5p; 130b-3p; 30c-5p; 98-5p; let-7f-5p; 15a-5p; 34a-5p; 7-5p; 665; 103a-3p; let-7a-3p; 151a-3p; 409-3p; 130a-3p; 92b-3p; 17-5p; 484; 192-5p; 26a-5p; 501-5p; 23b-3p; 877-5p; 625-5p; 26b-5p; let-7b-5p; 24-3p; 130b-5p; 454-3p; 145-5p; 30d-5p; 301a-3p; 296-3p |
| Protein export | 3.53E-03 | 31 | 44 | let-7e-5p; 92a-3p; 320b; 25-3p; 181a-2-3p; 128-3p; 323b-3p; 1-3p; 379-5p; 30e-5p; 30c-5p; 98-5p; 15a-5p; 34a-5p; 665; 15b-5p; 409-3p; 320a-3p; 423-5p; 584-5p; 92b-3p; 93-3p; 17-5p; 484; 192-5p; 421; 497-5p; 503-5p; 195-5p; 133a-3p; 664a-5p; 299-5p; 26b-5p; let-7b-5p; 24-3p; 671-5p; 329-3p; 145-5p; 140-5p; 30d-5p; 34c-5p; 129-5p; 1307-3p; 296-3p |
| Glycosylphosphatidylinositol GPI-anchor biosynthesis | 5.81E-03 | 31 | 43 | 590-3p; 378a-5p; let-7e-5p; 92a-3p; 542-3p; 374a-5p; 31-5p; let-7a-5p; 22-3p; 149-5p; 1-3p; 130b-3p; 98-5p; 34a-5p; 7-5p; 411-5p; 665; 320a-3p; 130a-3p; 27b-3p; 374b-5p; 181b-5p; 584-5p; 23a-3p; 92b-3p; 17-5p; 192-5p; 26a-5p; 941; 23b-3p; 26b-5p; let-7b-5p; 130b-5p; 337-3p; 190a-5p; 454-3p; 145-5p; 99b-3p; 376b-3p; 9-5p; 301a-3p; 129-5p; 769-5p |
| Nicotinate and nicotinamide metabolism | 5.64E-03 | 30 | 42 | 107; 590-3p; 92a-3p; 542-3p; 374a-5p; 22-3p; 128-3p; 126-3p; 1-3p; 98-5p; 15a-5p; 34a-5p; 7-5p; 15b-5p; 103a-3p; 320a-3p; 374b-5p; 181b-5p; 484; 192-5p; 421; 497-5p; 26a-5p; 503-5p; 195-5p; 501-5p; 23b-3p; 7704; 133a-3p; 664a-5p; 15b-3p; 26b-5p; 206; 24-3p; 130b-5p; 329-3p; 125a-5p; 377-3p; 9-5p; 204-5p; 500a-5p; 543 |
| Fatty acid elongation | 6.93E-03 | 30 | 42 | 590-3p; 378a-5p; 30a-3p; 92a-3p; 542-3p; 197-3p; 486-3p; 128-3p; 99a-5p; 1-3p; 30e-5p; 130b-3p; 30c-5p; 98-5p; 7-5p; 411-5p; 665; 15b-5p; 191-5p; 151a-3p; 320a-3p; 130a-3p; 17-5p; 484; 192-5p; 421; 26a-5p; 501-5p; 877-5p; 625-5p; 26b-5p; let-7b-5p; 24-3p; 190a-5p; 454-3p; 329-3p; 30d-5p; 9-5p; 301a-3p; 129-5p; 1307-3p; 204-5p |
| Staphylococcus aureus infection | 2.11E-02 | 32 | 42 | 342-3p; 378a-5p; let-7e-5p; 92a-3p; let-7d-5p; let-7a-5p; 197-3p; 149-5p; 128-3p; 1306-5p; 98-5p; let-7f-5p; 15a-5p; 34a-5p; 330-5p; 7-5p; 665; 576-5p; 15b-5p; 27b-3p; 423-5p; 22-5p; 92b-3p; 17-5p; 497-5p; 26a-5p; 195-5p; 7704; 127-5p; 26b-5p; let-7b-5p; 24-3p; 130b-5p; 3925-5p; 337-3p; 329-3p; 125a-5p; 129-5p; 1307-3p; 769-5p; 204-5p; 296-3p |
| Histidine metabolism | 7.07E-05 | 24 | 41 | 107; 1185-1-3p; 30a-3p; let-7e-5p; 92a-3p; let-7d-5p; 100-3p; let-7a-5p; 22-3p; 149-5p; 128-3p; 205-5p; 1-3p; let-7f-5p; 15a-5p; 34a-5p; 7-5p; 665; 15b-5p; 103a-3p; let-7a-3p; 181b-5p; 17-5p; 192-5p; 497-5p; 328-3p; 195-5p; 127-5p; let-7b-5p; 24-3p; 130b-5p; 671-5p; 329-3p; 99b-5p; 145-5p; 377-3p; 493-3p; 9-5p; 129-5p; 628-5p; 500a-5p |
| Tyrosine metabolism | 1.19E-03 | 27 | 41 | 107; 483-5p; 342-3p; 1185-1-3p; let-7e-5p; 92a-3p; let-7d-5p; let-7a-5p; 22-3p; 205-5p; 1-3p; 98-5p; let-7f-5p; 15a-5p; 34a-5p; 330-5p; 7-5p; 665; 15b-5p; 103a-3p; let-7a-3p; 320a-3p; 181b-5p; 93-3p; 17-5p; 484; 497-5p; 195-5p; 664a-5p; 26b-5p; let-7b-5p; 24-3p; 671-5p; 329-3p; 889-3p; 145-5p; 9-5p; 628-5p; 376c-3p; 769-5p; 500a-5p |
| Pentose phosphate pathway | 4.23E-03 | 28 | 40 | 378a-5p; let-7e-5p; 92a-3p; 542-3p; 197-3p; 25-3p; 149-5p; 128-3p; 1-3p; 30e-5p; 30c-5p; 15a-5p; 34a-5p; 330-5p; 665; 15b-5p; 191-5p; 320a-3p; 27b-3p; 423-5p; 92b-3p; 17-5p; 484; 192-5p; 26a-5p; 195-5p; 23b-3p; 133a-3p; 26b-5p; let-7b-5p; 206; 24-3p; 671-5p; 125a-5p; 493-3p; 30d-5p; 129-5p; 376c-3p; 296-3p; 378a-3p |
| beta-Alanine metabolism | 8.02E-03 | 29 | 40 | 107; 342-3p; 1185-1-3p; 30a-3p; 92a-3p; let-7a-5p; 149-5p; 128-3p; 1-3p; let-7f-5p; 15a-5p; 34a-5p; 7-5p; 15b-5p; 103a-3p; let-7a-3p; 27b-3p; 181b-5p; 17-5p; 484; 192-5p; 421; 497-5p; 26a-5p; 328-3p; 195-5p; 664a-5p; 26b-5p; let-7b-5p; 24-3p; 130b-5p; 329-3p; 99b-5p; 145-5p; 377-3p; 493-3p; 491-5p; 9-5p; 129-5p; 204-5p |
| Glycosaminoglycan biosynthesis - heparan sulfate heparin | 1.44E-02 | 29 | 40 | 30a-3p; let-7e-5p; 92a-3p; 486-3p; 25-3p; 128-3p; 99a-5p; 1306-5p; 1-3p; 30e-5p; 30c-5p; 485-3p; 15a-5p; 34a-5p; 665; 576-5p; 15b-5p; 191-5p; 320a-3p; 382-5p; 23a-3p; 92b-3p; 17-5p; 484; 192-5p; 497-5p; 503-5p; 195-5p; 941; 23b-3p; 26b-5p; let-7b-5p; 24-3p; 329-3p; 493-3p; 30d-5p; 1307-3p; 296-3p; 500a-5p; 378a-3p |
| Steroid hormone biosynthesis | 4.25E-02 | 31 | 40 | 30a-3p; 92a-3p; 542-3p; 181a-2-3p; 205-5p; 1-3p; 98-5p; let-7f-5p; 15a-5p; 665; 15b-5p; 191-5p; 409-3p; 27b-3p; 23a-3p; 22-5p; 17-5p; 192-5p; 497-5p; 708-5p; 503-5p; 195-5p; 501-5p; 23b-3p; 133a-3p; 127-5p; 664a-5p; 26b-5p; let-7b-5p; 24-3p; 130b-5p; 3925-5p; 329-3p; 129-5p; 376c-3p; 1307-3p; 204-5p; 296-3p; 500a-5p; 378a-3p |
| Folate biosynthesis | 3.14E-06 | 20 | 39 | 590-3p; 378a-5p; let-7e-5p; 92a-3p; let-7d-5p; let-7a-5p; 486-3p; 25-3p; 149-5p; 629-5p; 1-3p; 323a-3p; 130b-3p; 98-5p; let-7f-5p; 15a-5p; 34a-5p; 665; 15b-5p; 320a-3p; 130a-3p; 628-3p; 92b-3p; 93-3p; 484; 192-5p; 497-5p; 503-5p; 195-5p; 26b-5p; let-7b-5p; 206; 24-3p; 454-3p; 99b-5p; 145-5p; 9-5p; 301a-3p; 204-5p |
| Allograft rejection | 5.06E-03 | 27 | 39 | 30a-3p; let-7e-5p; 92a-3p; 25-3p; 149-5p; 128-3p; 1306-5p; 485-3p; 98-5p; 15a-5p; 34a-5p; 330-5p; 576-5p; 15b-5p; 409-3p; 320a-3p; 130a-3p; 27b-3p; 423-5p; 23a-3p; 22-5p; 92b-3p; 17-5p; 484; 503-5p; 7704; 625-5p; 6511a-3p; 26b-5p; 24-3p; 130b-5p; 329-3p; 145-5p; 125a-5p; 491-5p; 9-5p; 628-5p; 204-5p; 296-3p |
| Citrate cycle TCA cycle | 3.96E-04 | 23 | 38 | 107; 342-3p; 1185-1-3p; 92a-3p; 374a-5p; let-7a-5p; 197-3p; 320b; 22-3p; 149-5p; 99a-5p; 205-5p; 1-3p; 30c-5p; 98-5p; 34a-5p; 7-5p; 15b-5p; 103a-3p; let-7a-3p; 191-5p; 320a-3p; 27b-3p; 374b-5p; 23a-3p; 92b-3p; 93-3p; 17-5p; 484; 26a-5p; 503-5p; 23b-3p; 26b-5p; let-7b-5p; 24-3p; 130b-5p; 125a-5p; 129-5p |
| Pantothenate and CoA biosynthesis | 3.60E-02 | 29 | 38 | 27a-5p; 1185-1-3p; 92a-3p; 374a-5p; let-7a-5p; 25-3p; 128-3p; 205-5p; 485-3p; 98-5p; 15a-5p; 34a-5p; 7-5p; 15b-5p; let-7a-3p; 409-3p; 320a-3p; 27b-3p; 374b-5p; 92b-3p; 17-5p; 484; 192-5p; 497-5p; 154-3p; 26a-5p; 195-5p; 23b-3p; 664a-5p; 26b-5p; let-7b-5p; 337-3p; 145-5p; 125a-5p; 377-3p; 140-5p; 34c-5p; 769-5p |
| Ascorbate and aldarate metabolism | 2.47E-05 | 20 | 37 | 483-5p; 342-3p; 30a-3p; 92a-3p; let-7a-5p; 197-3p; 25-3p; 149-5p; 128-3p; 1-3p; let-7f-5p; 15a-5p; 34a-5p; 7-5p; 411-5p; 15b-5p; 409-3p; 181b-5p; 92b-3p; 17-5p; 192-5p; 497-5p; 328-3p; 503-5p; 195-5p; 133a-3p; 26b-5p; let-7b-5p; 24-3p; 130b-5p; 329-3p; 99b-5p; 377-3p; 9-5p; 129-5p; 376c-3p; 204-5p |
| Nitrogen metabolism | 1.31E-03 | 24 | 37 | let-7e-5p; 92a-3p; 542-3p; 374a-5p; let-7a-5p; 320b; 149-5p; 99a-5p; 1-3p; 98-5p; let-7f-5p; 15a-5p; 665; 15b-5p; 103a-3p; 320a-3p; 423-5p; 374b-5p; 93-3p; 17-5p; 484; 192-5p; 497-5p; 26a-5p; 503-5p; 195-5p; 23b-3p; 877-5p; 625-5p; 26b-5p; let-7b-5p; 24-3p; 329-3p; 99b-3p; 9-5p; 129-5p; 296-3p |
| Terpenoid backbone biosynthesis | 1.72E-03 | 24 | 37 | 107; 590-3p; let-7e-5p; 92a-3p; 374a-5p; let-7a-5p; 197-3p; 128-3p; 30e-5p; 130b-3p; 30c-5p; 98-5p; 103a-3p; 320a-3p; 130a-3p; 27b-3p; 423-5p; 374b-5p; 23a-3p; 92b-3p; 17-5p; 484; 192-5p; 26a-5p; 708-5p; 501-5p; 23b-3p; 26b-5p; let-7b-5p; 24-3p; 454-3p; 329-3p; 377-3p; 30d-5p; 9-5p; 301a-3p; 769-5p |
| Fatty acid biosynthesis | 5.17E-03 | 25 | 37 | 92a-3p; let-7d-5p; 374a-5p; 197-3p; 486-3p; 128-3p; 205-5p; 1-3p; 130b-3p; 30c-5p; 98-5p; 15a-5p; 34a-5p; 665; 15b-5p; 191-5p; 130a-3p; 27b-3p; 374b-5p; 92b-3p; 93-3p; 17-5p; 484; 192-5p; 497-5p; 26a-5p; 195-5p; 15b-3p; 26b-5p; let-7b-5p; 24-3p; 130b-5p; 454-3p; 301a-3p; 129-5p; 1307-3p; 378a-3p |
| Glyoxylate and dicarboxylate metabolism | 1.21E-02 | 27 | 37 | let-7e-5p; 92a-3p; 374a-5p; let-7a-5p; 320b; 486-3p; 25-3p; 149-5p; 99a-5p; 205-5p; 1-3p; let-7f-5p; 34a-5p; 665; 15b-5p; let-7a-3p; 191-5p; 320a-3p; 27b-3p; 423-5p; 181b-5p; 92b-3p; 484; 421; 23b-3p; 877-5p; 625-5p; 15b-3p; 26b-5p; let-7b-5p; 24-3p; 130b-5p; 34c-5p; 9-5p; 129-5p; 296-3p; 378a-3p |
| Phototransduction | 1.21E-02 | 27 | 37 | 107; 483-5p; 378a-5p; 486-3p; 1-3p; 130b-3p; let-7f-5p; 15a-5p; 7-5p; 411-5p; 15b-5p; 103a-3p; 320a-3p; 382-5p; 130a-3p; 27b-3p; 423-5p; 17-5p; 497-5p; 503-5p; 195-5p; 501-5p; 625-5p; let-7b-5p; 206; 24-3p; 598-3p; 3925-5p; 190a-5p; 454-3p; 889-3p; 376b-3p; 493-3p; 9-5p; 301a-3p; 129-5p; 1287-5p |
| Steroid biosynthesis | 3.02E-02 | 28 | 37 | 107; let-7e-5p; 92a-3p; let-7d-5p; 100-3p; 31-5p; let-7a-5p; 486-3p; 149-5p; 128-3p; 99a-5p; 205-5p; 130b-3p; 98-5p; 34a-5p; 7-5p; 665; 15b-5p; 103a-3p; let-7a-3p; 191-5p; 130a-3p; 27b-3p; 181b-5p; 93-3p; 17-5p; 484; 192-5p; 421; 23b-3p; 26b-5p; let-7b-5p; 24-3p; 130b-5p; 454-3p; 301a-3p; 769-5p |
| Mannose type O-glycan biosynthesis | 3.43E-03 | 24 | 36 | 92a-3p; 542-3p; 128-3p; 1-3p; 30e-5p; 130b-3p; 30c-5p; 98-5p; let-7f-5p; 15a-5p; 34a-5p; 330-5p; 15b-5p; 103a-3p; 130a-3p; 6724-5p; 23a-3p; 93-3p; 484; 26a-5p; 195-5p; 23b-3p; 139-5p; 877-5p; 625-3p; 625-5p; 26b-5p; 598-3p; 454-3p; 125a-5p; 30d-5p; 296-5p; 301a-3p; 129-5p; 769-5p; 296-3p |
| Vitamin digestion and absorption | 2.09E-02 | 27 | 36 | 590-3p; 30a-3p; let-7e-5p; 92a-3p; let-7d-5p; let-7a-5p; 1-3p; 379-5p; 130b-3p; 98-5p; let-7f-5p; 34a-5p; 7-5p; 411-5p; 665; 130a-3p; 27b-3p; 423-5p; 628-3p; 181b-5p; 484; 192-5p; 501-5p; 23b-3p; 26b-5p; let-7b-5p; 24-3p; 130b-5p; 454-3p; 145-5p; 140-5p; 491-5p; 9-5p; 301a-3p; 129-5p; 1185-5p |
| Glycosphingolipid biosynthesis - lacto and neolacto series | 2.49E-03 | 23 | 35 | 107; 590-3p; 378a-5p; 92a-3p; 128-3p; 1-3p; 30e-5p; 30c-5p; 98-5p; 15a-5p; 34a-5p; 330-5p; 665; 15b-5p; 103a-3p; 6724-5p; 23a-3p; 22-5p; 484; 192-5p; 497-5p; 26a-5p; 195-5p; 23b-3p; 26b-5p; 24-3p; 130b-5p; 598-3p; 329-3p; 125a-5p; 376b-3p; 30d-5p; 296-5p; 296-3p; 500a-5p |
| Non-homologous end-joining | 1.26E-02 | 25 | 35 | 27a-5p; 378a-5p; let-7e-5p; let-7d-5p; 31-5p; let-7a-5p; 486-3p; 128-3p; 1-3p; 379-5p; 485-3p; 98-5p; let-7f-5p; 34a-5p; 7-5p; 665; 423-5p; 181b-5p; 584-5p; 17-5p; 484; 192-5p; 941; 26b-5p; let-7b-5p; 24-3p; 130b-5p; 3925-5p; 671-5p; 99b-3p; 140-5p; 491-5p; 34c-5p; 129-5p; 628-5p |
| Autoimmune thyroid disease | 3.19E-02 | 26 | 35 | 30a-3p; let-7e-5p; 92a-3p; 25-3p; 149-5p; 128-3p; 1306-5p; 1-3p; 485-3p; 98-5p; let-7f-5p; 34a-5p; 330-5p; 576-5p; 320a-3p; 27b-3p; 423-5p; 23a-3p; 22-5p; 92b-3p; 17-5p; 484; 503-5p; 7704; 625-5p; 26b-5p; 24-3p; 130b-5p; 329-3p; 145-5p; 491-5p; 9-5p; 628-5p; 204-5p; 296-3p |
| Maturity onset diabetes of the young | 1.94E-06 | 15 | 33 | 133a-5p; 378a-5p; 1185-1-3p; 92a-3p; 197-3p; 629-5p; 1-3p; 130b-3p; 30c-5p; 34a-5p; 7-5p; 576-5p; 15b-5p; 409-3p; 320a-3p; 27b-3p; 23a-3p; 93-3p; 192-5p; 139-5p; 299-5p; 26b-5p; 432-5p; let-7b-5p; 24-3p; 329-3p; 140-5p; 493-3p; 34c-5p; 9-5p; 129-5p; 376c-3p; 296-3p |
| Arginine biosynthesis | 2.06E-02 | 23 | 32 | 342-3p; let-7e-5p; 92a-3p; let-7a-5p; 320b; 25-3p; 149-5p; 99a-5p; 205-5p; 1-3p; let-7f-5p; 7-5p; 665; 320a-3p; 423-5p; 181b-5p; 23a-3p; 93-3p; 17-5p; 484; 26a-5p; 877-5p; 625-5p; 26b-5p; 24-3p; 671-5p; 329-3p; 129-5p; 769-5p; 1271-5p; 296-3p; 543 |
| Glycosaminoglycan biosynthesis - keratan sulfate | 2.30E-03 | 19 | 31 | 107; 590-3p; 378a-5p; 92a-3p; 25-3p; 181a-2-3p; 128-3p; 1-3p; 30e-5p; 30c-5p; 98-5p; 15a-5p; 34a-5p; 15b-5p; 103a-3p; 6724-5p; 92b-3p; 484; 497-5p; 26a-5p; 195-5p; 625-5p; 26b-5p; 24-3p; 598-3p; 125a-5p; 30d-5p; 296-5p; 1307-3p; 204-5p; 296-3p |
| Phenylalanine metabolism | 1.37E-05 | 14 | 30 | 107; 342-3p; 590-3p; 1185-1-3p; 92a-3p; let-7a-5p; 22-3p; 205-5p; 1-3p; 15a-5p; 7-5p; 665; 15b-5p; 103a-3p; let-7a-3p; 320a-3p; 181b-5p; 93-3p; 484; 497-5p; 195-5p; 671-5p; 329-3p; 145-5p; 9-5p; 628-5p; 376c-3p; 1307-3p; 769-5p; 500a-5p |
| Glycosaminoglycan biosynthesis - chondroitin sulfate dermatan sulfate | 1.48E-02 | 20 | 29 | 92a-3p; 374a-5p; 197-3p; 486-3p; 25-3p; 149-5p; 1-3p; 30e-5p; 30c-5p; 98-5p; 34a-5p; 411-5p; 665; 15b-5p; 382-5p; 423-5p; 374b-5p; 92b-3p; 17-5p; 484; 192-5p; 26b-5p; let-7b-5p; 671-5p; 329-3p; 377-3p; 30d-5p; 34c-5p; 9-5p |
| Renin-angiotensin system | 1.57E-02 | 19 | 28 | 133a-5p; 378a-5p; let-7e-5p; 374a-5p; 486-3p; 128-3p; 1-3p; 379-5p; 130b-3p; 30c-5p; 485-3p; 34a-5p; 330-5p; 576-5p; 320a-3p; 130a-3p; 27b-3p; 374b-5p; 17-5p; 484; 192-5p; 503-5p; 941; 877-5p; 26b-5p; 24-3p; 329-3p; 193a-5p |
| Thiamine metabolism | 1.95E-03 | 14 | 25 | 342-3p; let-7e-5p; 92a-3p; let-7d-5p; let-7a-5p; 486-3p; 25-3p; 205-5p; 1-3p; 98-5p; let-7f-5p; 665; let-7a-3p; 151a-3p; 320a-3p; 423-5p; 17-5p; 26b-5p; let-7b-5p; 206; 24-3p; 145-5p; 129-5p; 769-5p; 204-5p |
| Ubiquinone and other terpenoid-quinone biosynthesis | 1.41E-02 | 15 | 23 | 342-3p; 590-3p; 92a-3p; 25-3p; 99a-5p; 1-3p; 7-5p; 411-5p; 665; 320a-3p; 423-5p; 92b-3p; 484; 192-5p; 133a-3p; 664a-5p; 26b-5p; 206; 24-3p; 3925-5p; 337-3p; 376c-3p; 769-5p |
| Glycosaminoglycan degradation | 3.59E-02 | 16 | 23 | 107; 342-3p; 30a-3p; 542-3p; 25-3p; 485-3p; 34a-5p; 665; 103a-3p; 181b-5p; 17-5p; 484; 26a-5p; 941; 23b-3p; 26b-5p; let-7b-5p; 24-3p; 491-5p; 34c-5p; 9-5p; 1307-3p; 769-5p |
| Glycosphingolipid biosynthesis - ganglio series | 2.24E-02 | 14 | 21 | 92a-3p; 374a-5p; 25-3p; 30e-5p; 30c-5p; let-7f-5p; 7-5p; 665; 92b-3p; 484; 503-5p; 501-5p; 26b-5p; let-7b-5p; 24-3p; 3925-5p; 377-3p; 30d-5p; 1307-3p; 204-5p; 500a-5p |
| Asthma | 3.79E-02 | 14 | 20 | let-7d-5p; 128-3p; 98-5p; let-7f-5p; 34a-5p; 576-5p; 130a-3p; 27b-3p; 423-5p; 22-5p; 17-5p; 503-5p; 7704; 625-5p; 26b-5p; 24-3p; 130b-5p; 145-5p; 9-5p; 204-5p |
| Vitamin B6 metabolism | 5.33E-03 | 11 | 19 | 342-3p; 92a-3p; 25-3p; 1-3p; 15a-5p; 34a-5p; 15b-5p; 382-5p; 484; 421; 497-5p; 26a-5p; 503-5p; 195-5p; 26b-5p; 24-3p; 145-5p; 34c-5p; 769-5p |
| Riboflavin metabolism | 1.23E-03 | 9 | 18 | 25-3p; 205-5p; 1-3p; 98-5p; 15a-5p; 15b-5p; 103a-3p; let-7a-3p; 151a-3p; 320a-3p; 17-5p; 497-5p; 503-5p; 195-5p; 127-5p; 664a-5p; 26b-5p; let-7b-5p |
| Taurine and hypotaurine metabolism | 1.42E-02 | 8 | 14 | 378a-5p; 197-3p; 128-3p; 99a-5p; 30e-5p; 30c-5p; 34a-5p; 7-5p; 382-5p; 501-5p; 625-5p; 6511a-3p; 30d-5p; 500a-5p |
| Phenylalanine, tyrosine and tryptophan biosynthesis | 7.36E-03 | 5 | 11 | 342-3p; 590-3p; 92a-3p; 205-5p; 1-3p; 665; 181b-5p; 93-3p; 484; 376c-3p; 769-5p |
| Neomycin, kanamycin and gentamicin biosynthesis | 1.06E-02 | 5 | 10 | 98-5p; 576-5p; 409-3p; 181b-5p; 484; 23b-3p; let-7b-5p; 329-3p; 99b-5p; 125a-5p |
| Valine, leucine and isoleucine biosynthesis | 1.67E-02 | 4 | 8 | 92a-3p; let-7a-5p; 25-3p; 7-5p; 92b-3p; 484; let-7b-5p; 769-5p |
