# Supplementary material for: Differential microRNA profiles of intramuscular and secreted extracellular vesicles in human tissue-engineered muscle
Source: Front Physiol. 2022 Aug 25;13:937899. doi: 10.3389/fphys.2022.937899 (PMC9452896; doi:10.3389/fphys.2022.937899)
Supplement: Supplementary file 3 [file Image1.pdf]

Supplementary Figure 1: EV Characterization

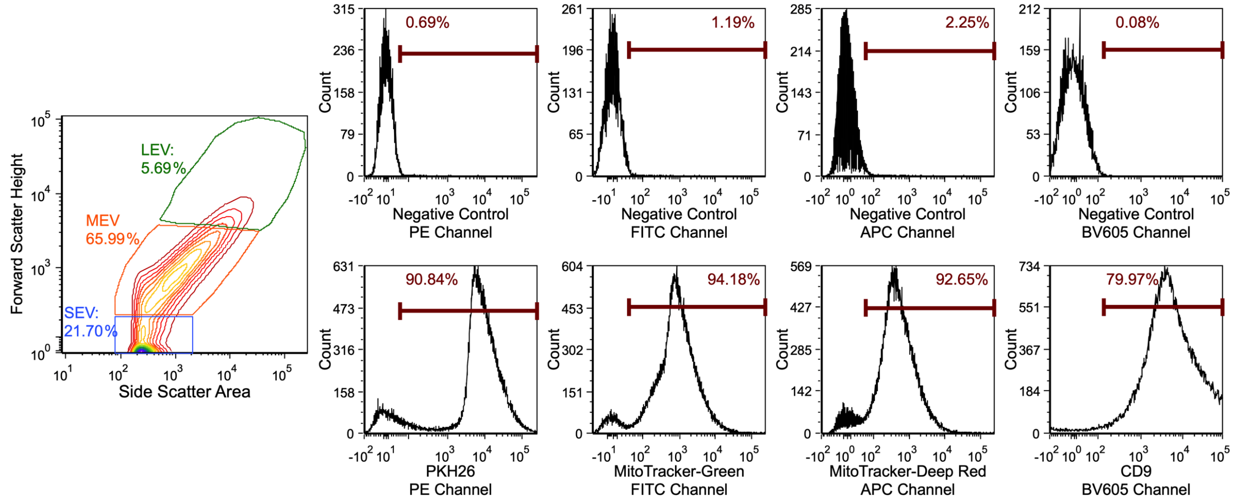

Isolated EVs were characterized by high resolution flow cytometry. The contour plot displays forward scatter (for relative size) and side scatter (for internal complexity and granularity), and the histograms display corresponding negative controls and staining of PKH26 (for bilayer structure), MitoTracker Green (for total mitochondria), MitoTracker Deep Red (for functional respiring mitochondria) and surface marker CD9.

Supplementary Figure 2: Heatmap of correlated miRs

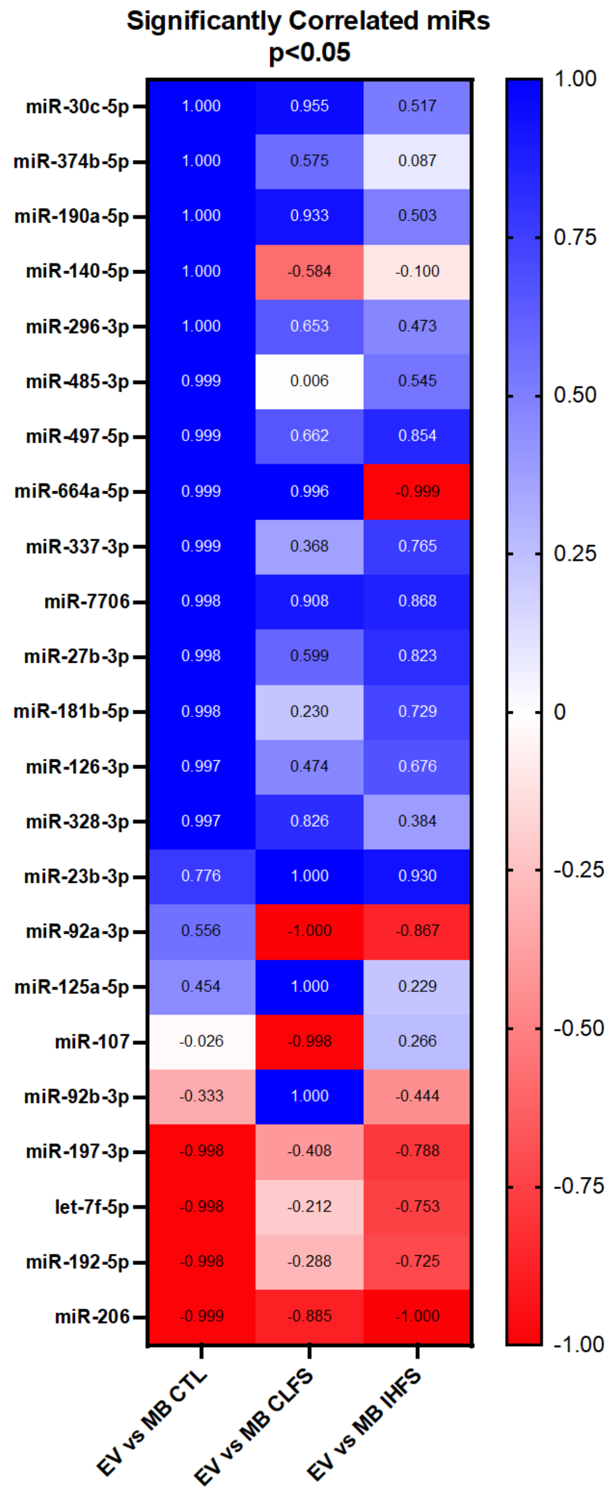

Abbreviations: EV, extracellular vesicles; MB, myobundle; CTL, control; CLFS, chronic low-frequency stimulation; IHFS, intermittent high-frequency stimulation. Legend: Data presented are Pearson correlation R values. Blue represents a positive correlation whereas red represents a negative correlation between EV and myobundle populations for each treatment.

Supplementary Figure 3: Myobundle Enriched miRs

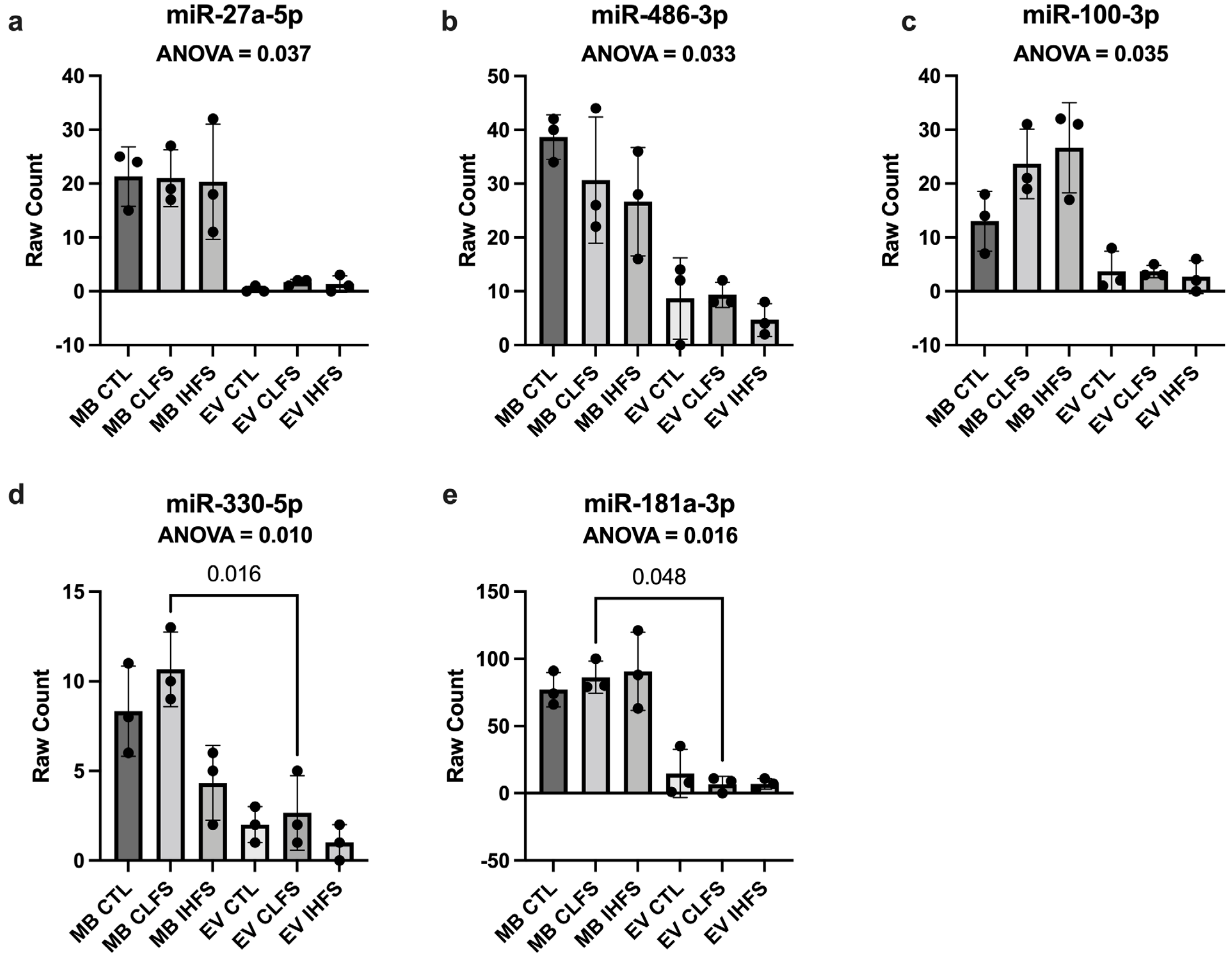

Abbreviations: MB, myobundle; EV, extracellular vesicle. Data are presented as mean±SD for miR-27a-5p (panel a), miR-486-3p (panel b), miR-100-3p (panel c), miR-330-5p (panel d), and miR-181a-3p (panel e). Raw count was found to be higher in the myobundle population subjected to CLFS for miRs-330-5p and -181a-3p.

Supplementary Figure 4: Extracellular Vesicle Enriched miRs

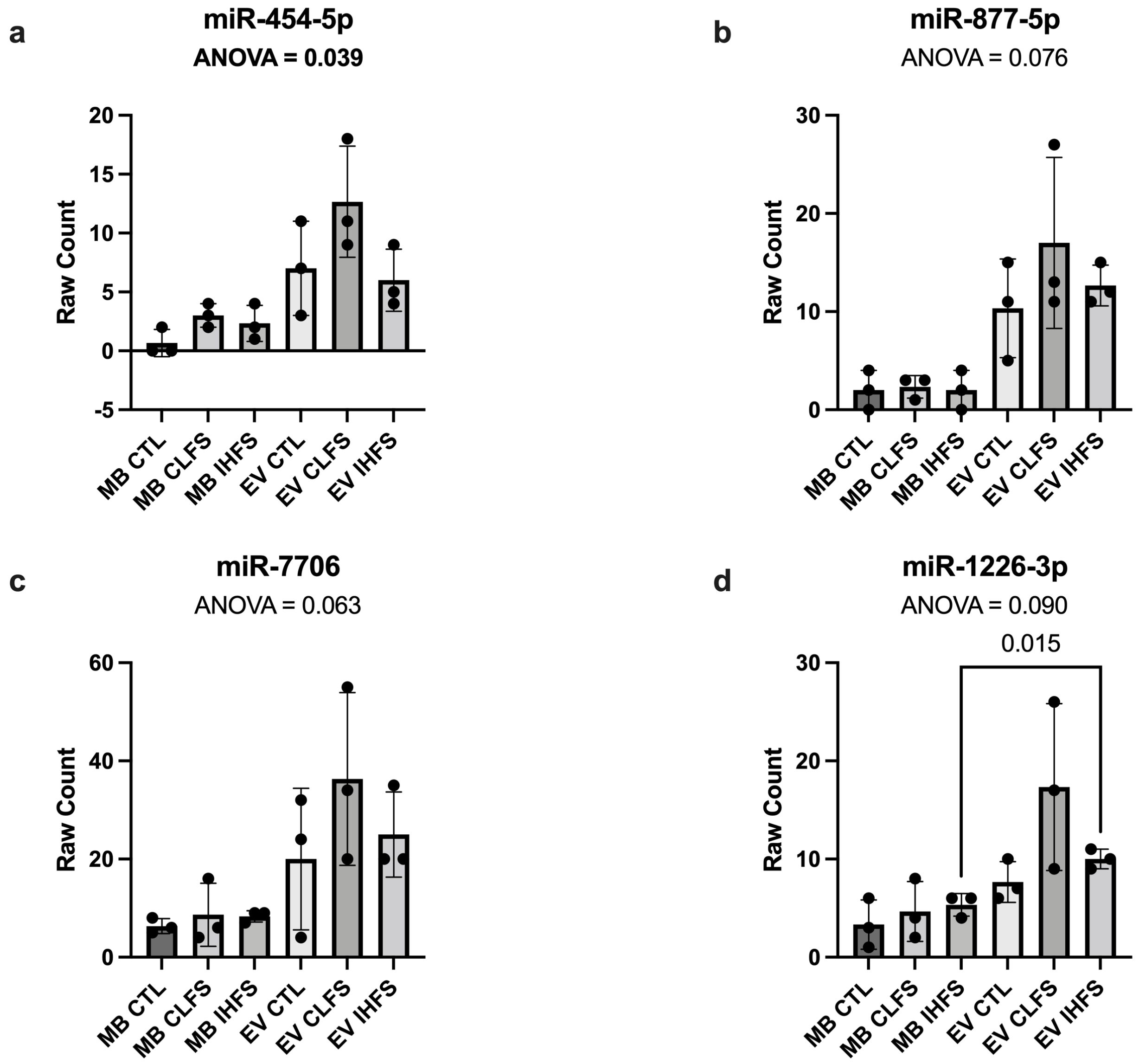

Abbreviations: MB, myobundle; EV, extracellular vesicle. Data are presented as mean±SD for miR-454-5p (panel a), miR-877-5p (panel b), miR-7706 (panel c), and miR 1226-3p (panel d). miR-1226-3p was identified as trending toward differential expression with raw counts being higher in the EV population subjected to IHFS.

Supplementary Figure 5: Overlap of Normalization Methods

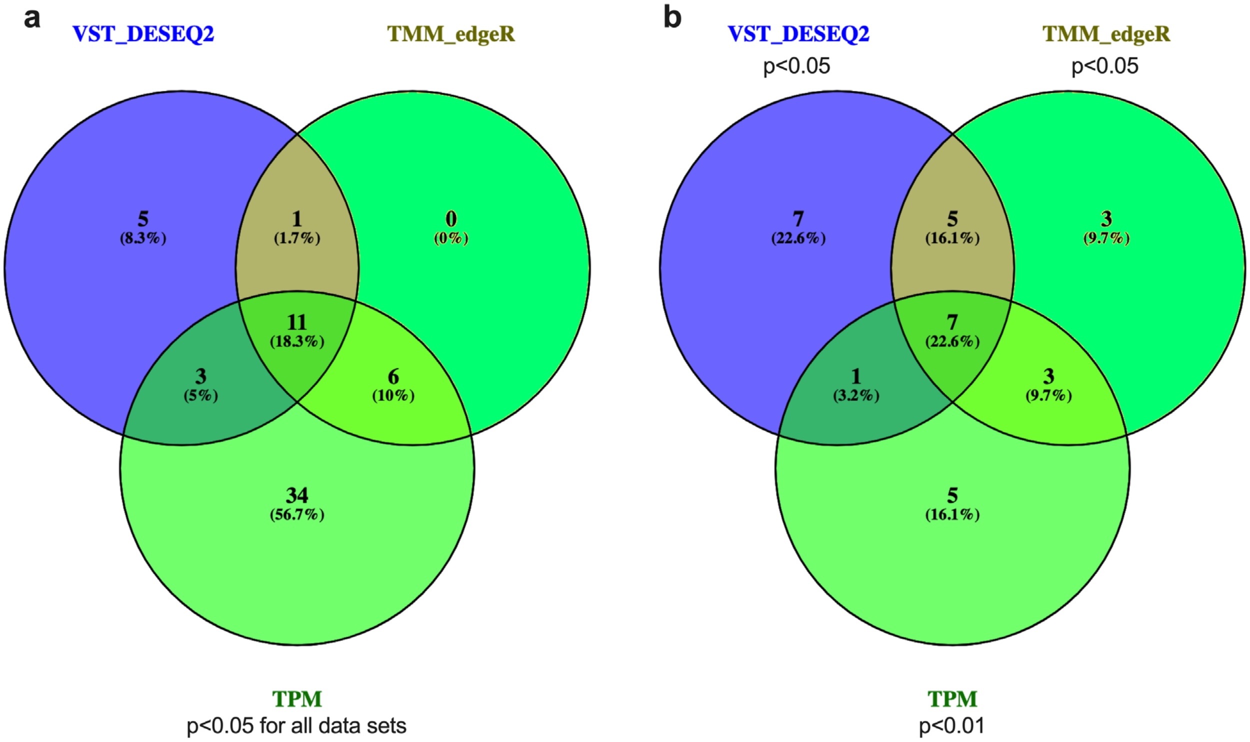

Abbreviations: VST, variance stabilizing technique; TMM, trimmed mean of M-values; TPM, transcripts per million. These data represent the number of differentially expressed miRs identified with $p<0.05$ for all normalization methods (panel a), and DESEQ2 and edgeR cutoff threshold at $p<0.05$ with TPM cutoff threshold set at $p<0.01$ (panel b).
